# dbCPG: A web resource for cancer predisposition genes

**DOI:** 10.18632/oncotarget.9334

**Published:** 2016-05-12

**Authors:** Ran Wei, Yao Yao, Wu Yang, Chun-Hou Zheng, Min Zhao, Junfeng Xia

**Affiliations:** ^1^ Institute of Health Sciences, School of Computer Science and Technology, Anhui University, Hefei, Anhui, 230601, China; ^2^ College of Electrical Engineering and Automation, Anhui University, Hefei, Anhui, 230601, China; ^3^ Co-Innovation Center for Information Support and Assurance Technology, Anhui University, Hefei, Anhui, 230601, China; ^4^ School of Engineering, Faculty of Science, Health, Education and Engineering, University of Sunshine Coast, Maroochydore DC, Queensland, 4558, Australia

**Keywords:** cancer predisposition gene, database, functional annotation, gene prioritization, network module

## Abstract

Cancer predisposition genes (CPGs) are genes in which inherited mutations confer highly or moderately increased risks of developing cancer. Identification of these genes and understanding the biological mechanisms that underlie them is crucial for the prevention, early diagnosis, and optimized management of cancer. Over the past decades, great efforts have been made to identify CPGs through multiple strategies. However, information on these CPGs and their molecular functions is scattered. To address this issue and provide a comprehensive resource for researchers, we developed the Cancer Predisposition Gene Database (dbCPG, Database URL: http://bioinfo.ahu.edu.cn:8080/dbCPG/index.jsp), the first literature-based gene resource for exploring human CPGs. It contains 827 human (724 protein-coding, 23 non-coding, and 80 unknown type genes), 637 rats, and 658 mouse CPGs. Furthermore, data mining was performed to gain insights into the understanding of the CPGs data, including functional annotation, gene prioritization, network analysis of prioritized genes and overlap analysis across multiple cancer types. A user-friendly web interface with multiple browse, search, and upload functions was also developed to facilitate access to the latest information on CPGs. Taken together, the dbCPG database provides a comprehensive data resource for further studies of cancer predisposition genes.

## INTRODUCTION

Cancer, as the second leading cause of death, is a major public health problem in the world. For instance, it is estimated that there are 1,658,370 new cancer cases and 589,430 cancer deaths in the United States in 2015 [1]. At least 3% of all cancers are hereditary, meaning a germline pathogenic mutation can contribute to cancer development [2]. Genes in which germline mutations increase the risks of developing cancer are called cancer predisposition genes (CPGs) [2]. It has long been acknowledged that the most of CPGs play significant role in fundamental biological processes such as DNA repair and cell cycle regulation [3]. Most CPGs act as tumor suppressors with mutations that abolish their function and contribute to the development of a cancer, only a few CPGs predisposed to cancer is the result of gain-of-function mutations [2]. Besides, the contribution of CPG mutations across cancer types is highly variable. For example, it was estimated that around 5–10% of breast cancers are due to germline mutations in CPGs such as *BRCA1* and *BRCA2* [4], while lung cancers are thought to be more strongly related to environment components.

The identification of CPG has a substantial impact on cancer detection and prevention [5]. As a result, many small-scale studies such as candidate gene approaches and high-throughput strategies like genome-wide mutation analyses (including exome and genome sequencing) have been applied onto the studies of CPG over the past decades. This has resulted in generation of enormous data and revelation of hundreds of disease-associated genomic markers in cancer patients, thus providing researchers important resources to potentially explore the molecular mechanisms and identify CPGs.

In the past few years, a larger number of database have emerged which mainly focused on a particular class of cancer genes as exemplified by tumor suppressor gene database [6], candidate cancer gene database [7], and cancer-related immunological gene database [8]. However, to the best of our knowledge, there is no database that focuses on CPGs. To fill this gap, we developed a comprehensive literature based database called dbCPG (Cancer Predisposition Gene Database). Aiming to efficiently integrate and analyze all or most of the published CPGs, we firstly performed a collection and review of peer-reviewed literature from databases such as Rahman's data [2], PubMed abstract (http://www.ncbi.nlm.nih.gov/pubmed), GeneReview [9], Online Mendelian Inheritance in Man (OMIM) [10] and Gene Reference Into Function (GeneRIF) [11]. Then we manually checked and obtained a total of 827 human (724 protein-coding, 23 non-coding and 80 unknown type genes (the type of gene is labelled as ‘unknown type’ in NCBI)), 637 rat and 658 mouse CPGs. To provide a comprehensive data source for cancer predisposition genes, the dbCPG integrates multitudinous annotation information for each CPG, including general information from NCBI, gene expressions from Expression Atlas [12], methylation sites from DiseaseMeth database [13], post-translational modification (PTM) information from dbPTM [14], germline mutation data from ClinVar [15], interacting partners from PINA [16], pathway information from MSigDB [17], and drug information from DGIdb [18]. As the first database for CPGs, dbCPG provides not only a comprehensive resource of CPGs for the cancer research community but also provides useful information for clinical application, such as diagnosis, optimized management and prevention of cancer.

## RESULTS AND DISCUSSION

### Representative entry in dbCPG

**Figure 1:**
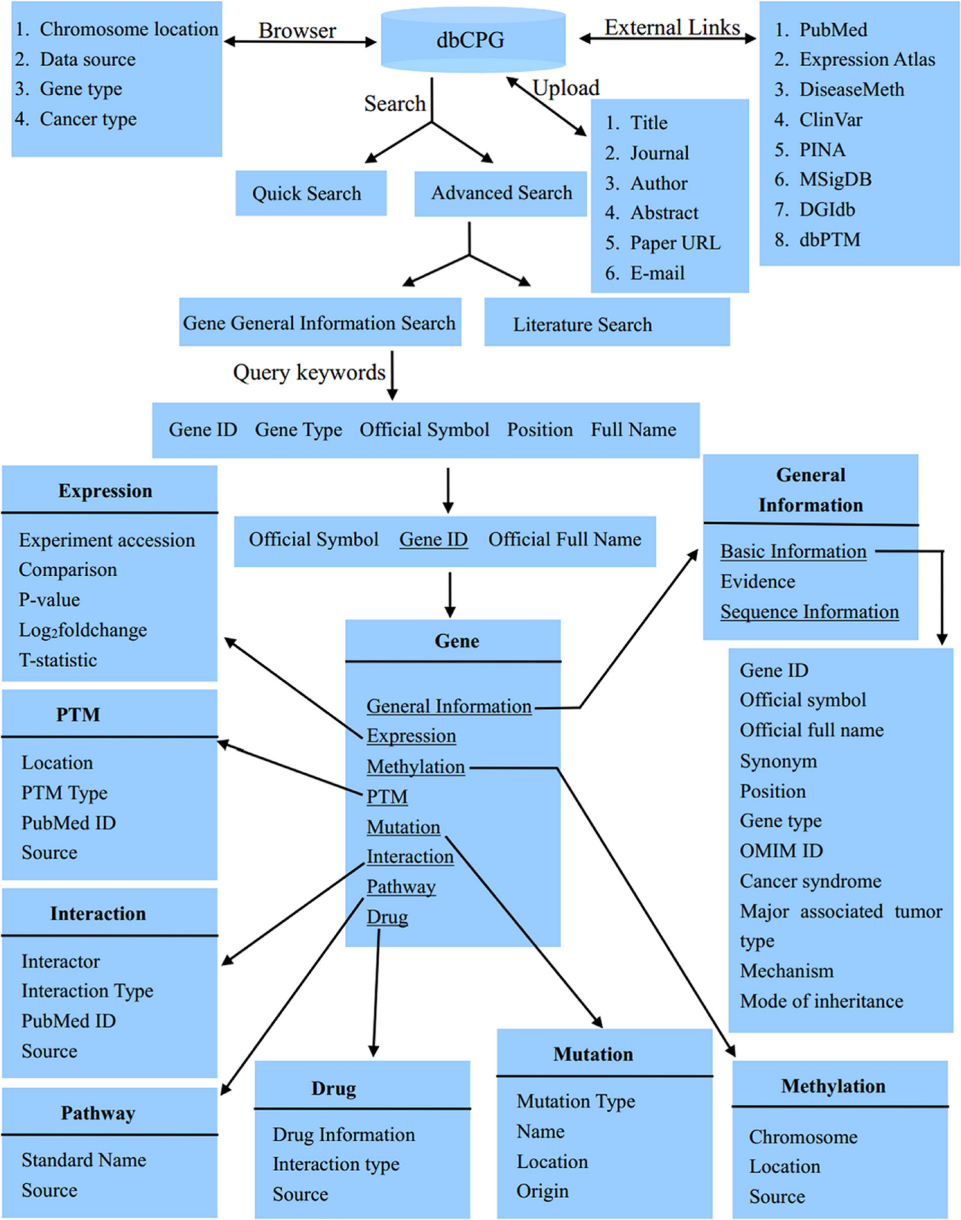
The database structure of dbCPG

The gene entries in dbCPG can be easily accessed in a variety of ways. The main page for each gene displays 8 annotation categories, including ‘General Information’, ‘Expression’, ‘Methylation’, ‘PTM’, ‘Mutation’, ‘Interaction’, ‘Pathway’ and ‘Drug’ category (Figure 1).

In the ‘General Information’ category, basic gene information, nucleotide sequence and protein sequence are displayed in a tabular view. Summaries of literature evidence and data sources are also provided. The ‘Expression’ category provides a tabular data which exhibits the *P*-value, log2 fold change and T-statistic with different comparisons, like ‘high grade serous ovarian carcinoma’ vs ‘normal’, which were collected from Expression Atlas [12] with the keywords ‘cancer’ AND ‘Homo sapiens’. In the ‘Methylation’ category, we used the textual interfaces to depict the methylation information, which were obtained from DiseaseMeth database [13]. The chromosome, methylation location and their respective sources are displayed. In the ‘PTM’ category, we displayed the PTM type with different amino acid modified location. We also showed the PubMed ID and data sources. All of these data were obtained from dbPTM [14]. The ‘Mutation’ category presents different types of germline mutations that were extracted from ClinVar [15]. In the ‘Interaction’ category, users can view the interaction partners and types, which were derived from PINA database [16]. For each CPG, we offered its involved pathways in the ‘Pathway’ category, which were obtained from MSigDB [17]. In the ‘Drug’ category, users can explore drug related information such as drug name, interaction type and data source, which were extracted from DGIdb database [18].

### Database statistics

**Figure 2:**
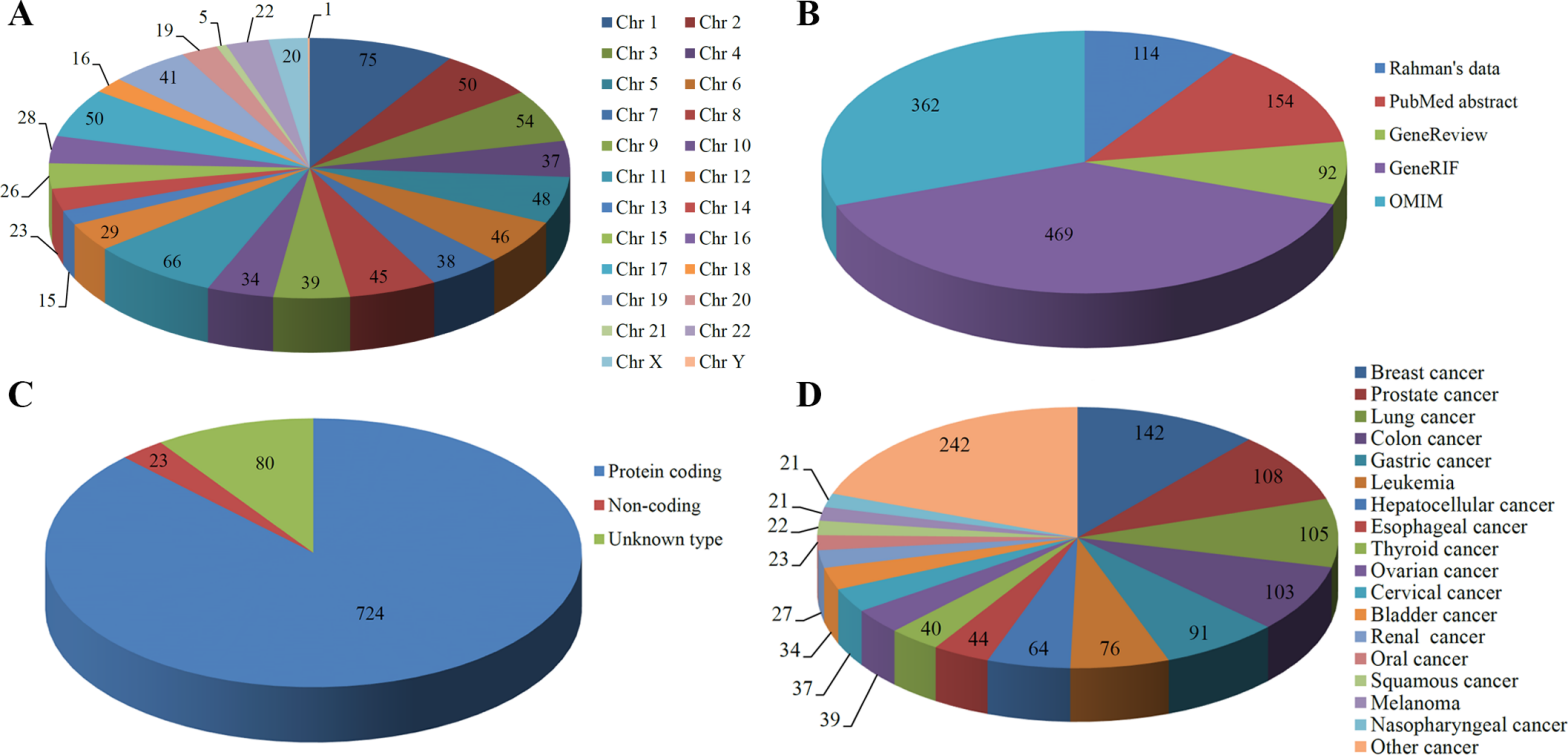
Data statistics based on (A) Chromosome location, (B) Data source, (C) Gene type, and (D) Cancer type in human CPGs

The current version of dbCPG contains 827 human, 637 rat and 658 mouse CPGs. Descriptions on the data sources and functional information are summarized in Table 1. For humans, each chromosome carries at least one CPG (Figure 2A), and most of CPGs is located on chromosome 1 (75 CPGs) and 11 (66 CPGs). In our database, the human CPGs were retrieved from five data sources (Figure 2B). Supplementary Figure S1 is a Venn diagram illustrating the overlapping CPGs among these five data sources. Since most of GeneRIFs were extracted from the title or abstract of the corresponding scientific paper [19], and OMIM is an authoritative catalog of human genes and traits, it is not surprising to see that a large proportion of human CPGs in dbCPG were obtained from GeneRIF (56.71%) or OMIM (43.77%).

**Table 1:** Annotation entry statistics for 827 CPGs

Data category	Related entries	Annotated CPGs	Content/sources
Human CPGs	827	827	Gene ID, official symbol, official full name, synonym, position, gene type, OMIM ID from Entrez gene database; cancer syndrome, major associated tumor type, mechanism of action of CPG mutations, mode of inheritance from PubMed
Rat CPGs	637	637	Rat CPGs mapped from HomoloGene
Mouse CPGs	658	658	Mouse CPGs mapped from MGI Human Mouse Orthologs
Literature	2097	805	Literature evidence for CPGs
OMIM	22	22	Disorder description for CPGs
Expression	8873	654	Expression Atlas database
Methylation	5292	695	DiseaseMeth database
PTM	11701	366	dbPTM
Germline mutation	29816	420	ClinVar
Interaction	20004	610	PINA database
Pathway	8640	580	MsigDB database
Drug	1651	133	DGIdb database

*CPG is short for cancer predisposition gene, MGI is short for mouse genome informatics, PTM is short for post-translational modification.

Among the 827 human CPGs, the majority of them (724) are protein-coding genes (Figure 2C). Only 23 belong to non-coding genes. In addition, 80 CPGs are labelled as ‘unknown type’ in dbCPG based on the annotation in NCBI. As CPGs have been reported in different types of cancer, this information were also included in dbCPG. In total, there are 90 types of cancer with reported CPGs in our database, of which the top 17 major cancer types contain at least 21 CPGs (Figure 2D). And breast cancer is the most frequent tumor with the number of CPGs.

**Figure 3:**
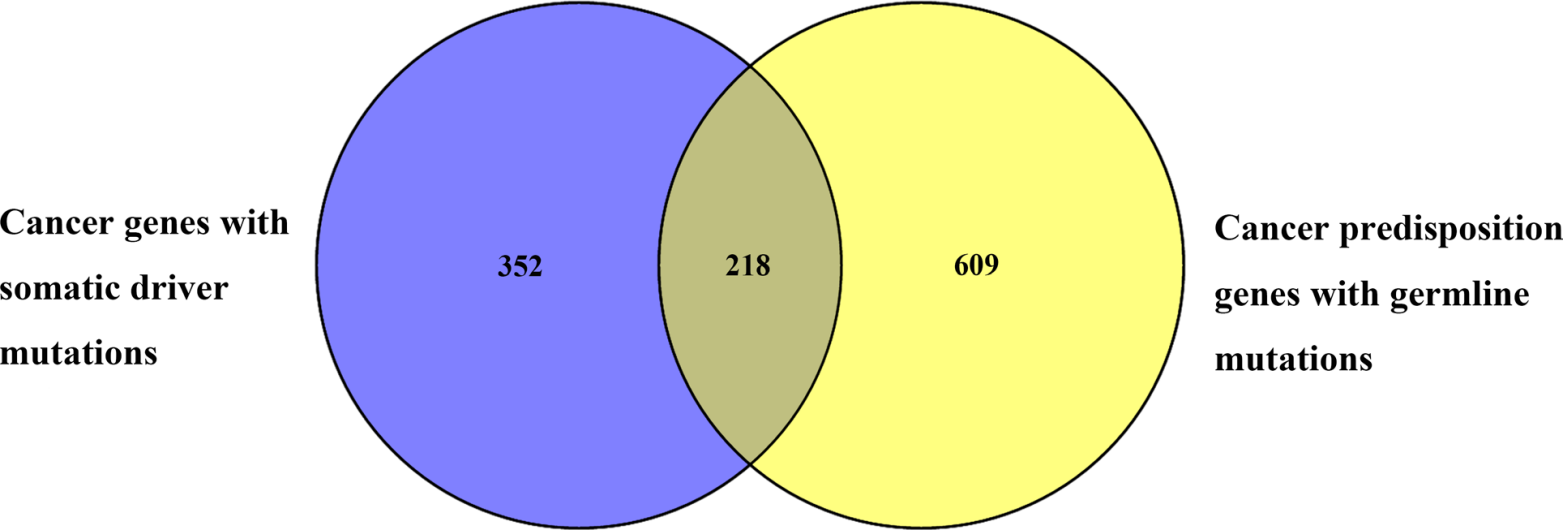
Overlap between cancer genes with somatic mutations and CPGs 570 cancer genes with somatic mutations are from COSMIC of which 218 are also included within the 827 human CPGs in dbCPG.

We also investigated the overlap between human CPGs and the known cancer genes with somatic mutations (Figure 3). 570 somatically mutated cancer genes were obtained from the COSMIC (Catalogue of Somatic Mutations in Cancer) database [20]. Of these, 218 are also known to be CPGs. These data reveal that 38% of somatically mutated cancer genes can cause predisposition to cancer when they have germline mutations. Conversely, we also see that about 26% of CPGs can contribute to carcinogenesis when they have somatic mutations. Considering the fact that cancer is a genetic disease driven by a combination of germline mutations coupled with the acquisition of somatic mutations, the integrated analysis of germline and somatic data can facilitate identification of likely pathogenic mutations and new cancer genes that are not readily identified by studying each data in isolation [21, 22].

### Biological features of protein-coding genes in dbCPG

As the majority of CPGs in our database are protein-coding genes (724 human protein-coding CPGs in Supplementary Table S1), we performed the pathway enrichment and disease association analyses on the 724 protein coding genes to better understand the biological function using the online tool KOBAS [23]. We collected the pathways and disease with an adjusted *P*-value < 0.05 as calculated by hypergeometric test followed by the Benjamini-Hochberg correction. As shown in Supplementary Table S3, a lot of the enriched pathways, such as ‘pathways in cancer’, ‘constitutive PI3K/AKT signaling in cancer’, ‘p53 signaling pathway’, and ‘PI3K/AKT signaling in cancer’ are cancer relevant. Among the top 20 enriched disease (Supplementary Table S4), there are 18 various types of cancer on the enriched list, including breast cancer, colorectal cancer, prostate cancer, lung cancer and ovarian cancer (Table 2).

**Table 2:** Top 20 enriched diseases of the 724 protein-coding CPGs

Disease name	Raw *P*-value	Benjamini-Hochberg adjusted *P*-value
Cancer	4.98E-30	5.67E-26
Breast cancer	3.77E-29	2.15E-25
Colorectal cancer	1.35E-27	5.13E-24
Lung cancer	5.18E-23	1.47E-19
Prostate cancer	4.04E-20	9.19E-17
Stomach cancer	3.54E-17	6.70E-14
Bladder cancer	8.49E-13	8.05E-10
Esophageal cancer	4.68E-12	2.32E-09
Ovarian cancer	2.72E-09	4.76E-07
Endometrial cancer	2.77E-08	2.76E-06
Endometriosis	3.59E-08	3.44E-06
Head and neck cancer	3.84E-08	3.55E-06
Oral cancer	1.11E-07	8.32E-06
Diabetes, type 1	1.24E-07	8.94E-06
Melanoma	1.28E-07	9.08E-06
Stomach neoplasms	4.64E-07	2.59E-05
Sarcoidosis	6.33E-07	3.32E-05
Infection	9.24E-07	4.45E-05
Neoplasms	9.81E-07	4.62E-05
Leukemia	1.09E-06	5.02E-05

To obtain comprehensive biological features, we also conducted enrichment tests on 724 human protein-coding CPGs by using DAVID server [24]. Statistically significant gene ontology (GO) terms and over-represented InterPro domain [25] were selected by an adjusted *P*-value < 0.05 calculated by hypergeometric test followed by the Benjamini-Hochberg correction. Using the complete human genes as background, the 724 protein-coding CPGs were over-represented in regulation of biological process, regulation of cellular process, regulation of cell proliferation and cell death according to GO Biological Processes terms (Supplementary Table S5). As shown in Supplementary Table S6, the most commonly represented InterPro domains were mainly related to kinase activities such as ‘tyrosine protein kinase, active site’, ‘tyrosine protein kinase’, ‘protein kinase, ATP binding site’ and ‘Protein kinase, core’, which highlight important roles of kinase activity in CPGs.

### The common CPGs across multiple cancer types

**Figure 4:**
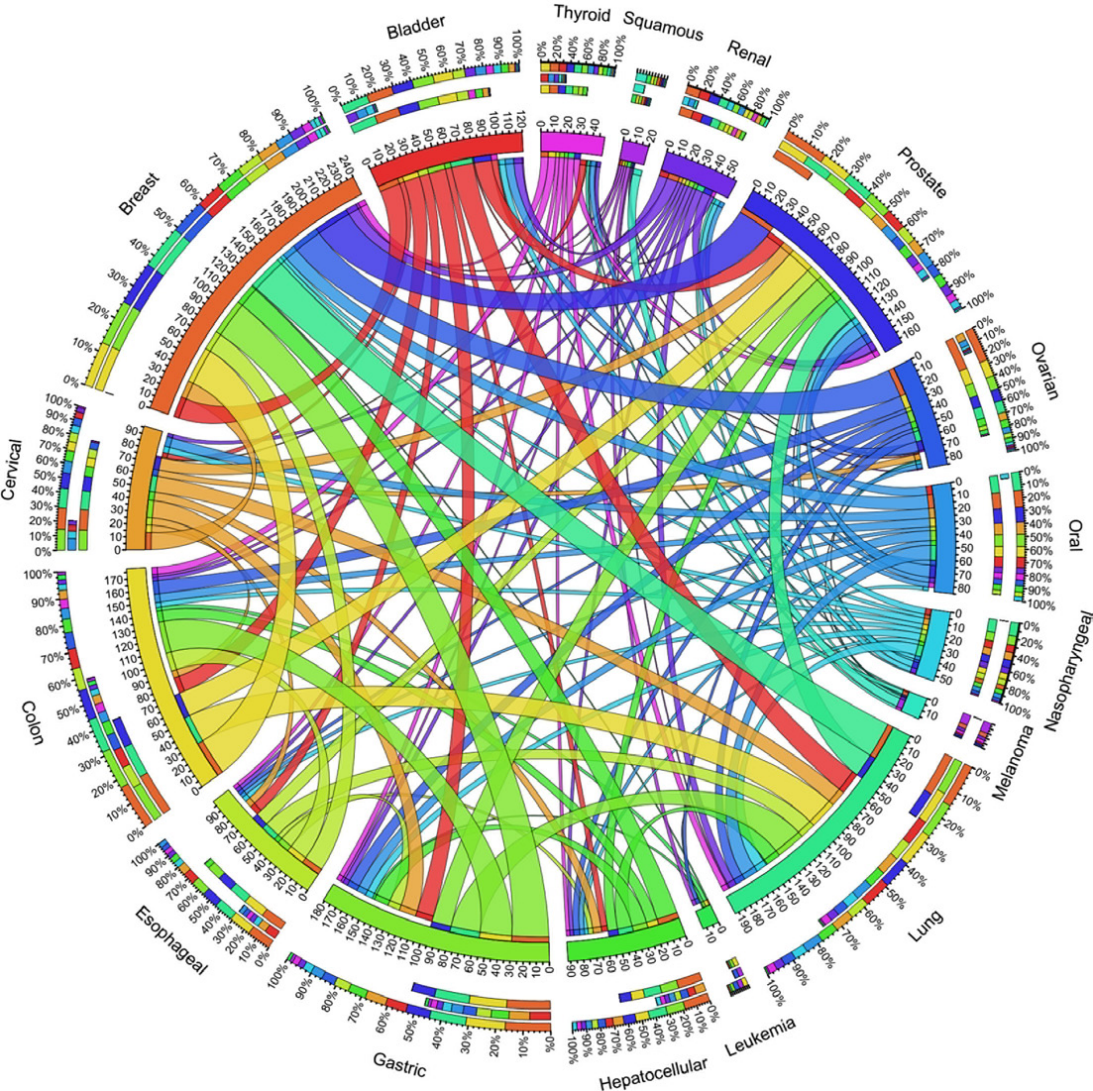
The shared CPGs across 17 cancer types The length of circularly arranged segment is proportional to the total CPGs in each cancer type. The ribbons connecting different segments represent the number of shared CPGs between cancer types. The three outer rings are stacked bar plots that represent relative contribution of other cancer types to the cancer type's totals, where the innermost, middle, and outermost ring represents the number of CPGs that other cancers share with a specific cancer, the number of CPGs that a specific cancer share with the other cancers, and the sum number of CPGs among different cancer types, respectively.

Based on the literature review, we provided all the CPGs in dbCPG with cancer type information. We grouped all the CPGs into 90 cancer types. The number of CPGs detected per cancer type varies considerably (range 1–144), with four types having more than 100 CPGs (cancers of breast, prostate, lung, and colon) and 31 types having only 1 CPG. To investigate the common mechanism of CPGs in different cancer types, we focused on the top 17 cancer types associated with more than 20 genes (Supplementary Table S7). Based on the common genes in the 17 cancer types, the overlapping relationships were plotted in Figure 4. The plot includes three outer rings, which represent relative contribution of other cancer types to the cancer types totals. It revealed that the multiple cancer types shared potential predisposition mechanisms. For example, we found 221 CPGs shared in two or more cancer types (Supplementary Table S8). Strikingly, there are three common CPGs (*GSTM1*, *MSH6*, and *TP53*) involved in at least 10 cancer types, in which germline mutations of these genes have been reported to increase individual susceptibility to a variety of human cancers [26–28].

### Prioritization of protein-coding CPGs and its enriched dense network module

Although the 724 protein-coding genes in dbCPG have literature evidence based on different data sources, we didn't investigate the importance of each CPG systematically. Hence, we performed gene prioritization analysis using ToppGene web server [29]. To prioritize genes, ToppGene uses a fuzzy-based similarity measure to calculate the similarity between two types of gene set (training set and test set) based on functional annotations. In this study, we compiled a training gene lists that included 57 well-established CPGs (Supplementary Table S9), which have at least 10 literature evidences. The remaining CPGs in dbCPG were used as the test set. Based on the gene ranking results of ToppGene (Supplementary Table S10), the top ranked genes tend to have multiple evidences. Besides 57 well-studied CPGs in the training set, *CTNNB1* was top ranked CPG in remaining 667 CPGs from the test set. Furthermore, functional analyses on the 100 CPGs (57 training genes and top 43 test genes) show similar distribution with the total 724 protein-coding CPGs (Supplementary Tables S11–S14).

**Figure 5:**
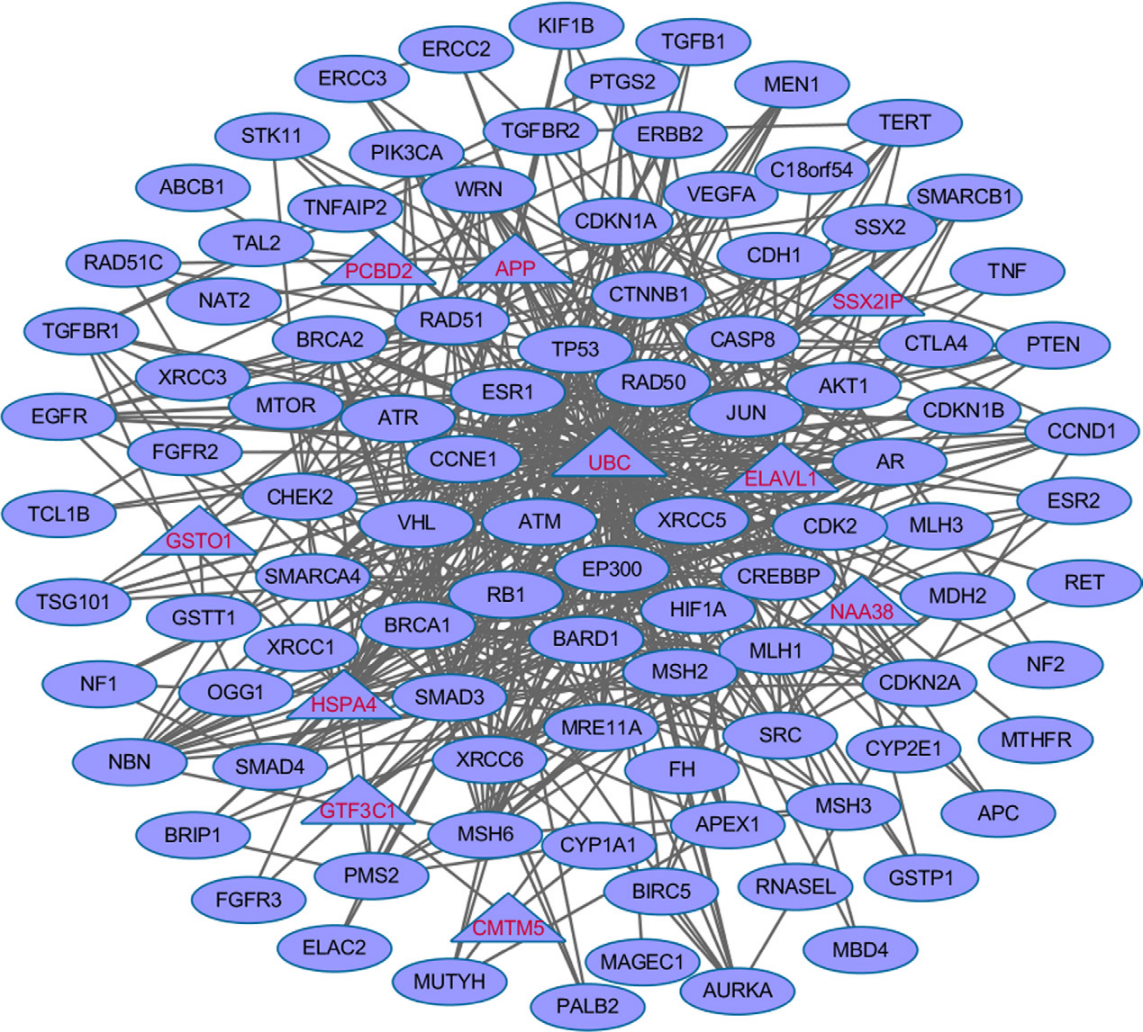
The enriched dense network module using the 100 CPG (57 training genes and top 43 test genes) based on protein-protein interaction data The 97 genes in diamond are terminal genes from the 100 CPGs. The remaining 10 genes in triangle are linker genes bridged the 92 genes.

We further explored the dense modules enriched with the 100 CPGs (57 training genes and top 43 test genes) through their protein-protein interactions by using Klein-Ravi algorithm in GeneRev [30]. We identified one module which contained 107 genes (Figure 5). Of the 107 nodes, 97 of them are from our dbCPG. The remaining 10 are the novel genes that may potentially bridge the cancer predisposition gene to fully implement their cellular function. In conclusion, the majority of the 100 CPGs connect each other and form a dense network, which also support the accuracy of our data curation.

## CONCLUSIONS

This study presents a unique resource, dbCPG, for the systematic annotation of susceptibility genes in cancer predisposition. Our aim is to collect a complete and up-to-date CPG resource and make it freely accessible to users. For each CPG in our database, we provided a wide range of information, including gene expression, methylation, PTM, germline mutation, protein-protein interaction, pathway, and drug information. We will update this database on a regular basis by adding new data from literature as well as other valuable resources. It is anticipated that dbCPG would serve as a valuable resource to the cancer research community.

## MATERIALS AND METHODS

### Data collection and literature curation

The goal of dbCPG database is to provide a comprehensive resource for investigation of CPGs and their molecular mechanisms in cancer, which can freely assist cancer research community to design the experiment, understand tumorigenic mechanisms and develop useful information for clinical application. Thus, we firstly collected 114 CPGs from Rahman's Nature paper [2], where the CPGs were identified based on literature review and database evaluations. Secondly, we performed a comprehensive literature search of PubMed on 8 April 2015 using the query expression: (‘cancer’ [Title/Abstract] OR ‘tumor’ [Title/Abstract]) AND (‘predisposition’ [Title/Abstract] OR ‘susceptibility’ [Title/Abstract]) AND (‘gene’ [Title/Abstract] OR ‘syndrome’ [Title/Abstract]), with the purpose of obtaining a precise and detailed list of publications for CPGs. As a result, we obtained 1319 PubMed abstracts. Then we extracted CPGs related sentences from the abstracts of these articles manually. We also read the full text to find the key sentences if necessary. Overall, 154 CPGs were collected from 624 related PubMed abstracts. Thirdly, 92 CPGs were identified from GeneReview [9], which is an online database mainly focused on specific heritable disease, on 20 May 2015 using the search terms: (‘neoplasms’ [All Fields] OR ‘neoplasms’ [All Fields] OR ‘cancer’ [All Fields]) AND (‘disease susceptibility’ [All Fields] OR (‘disease’ [All Fields] AND ‘susceptibility’ [All Fields]) OR ‘disease susceptibility’ [All Fields] OR ‘predisposition’ [All Fields]) AND (‘genes’ [All Fields] OR ‘genes’ [All Fields] OR ‘gene’ [All Fields]). Fourthly, we extracted 469 CPGs with the words: (‘cancer’ or ‘tumor’ or ‘carinoma’ or ‘neoplasm’) and (‘susceptibility’ or ‘predisposition’) from GeneRIF [11] on 23 May 2015, which is a clustering of short statements about gene function. Finally, we searched 362 CPGs derived from OMIM [10], a comprehensive database of human gene and genetic disorders, on 28 May 2015. After combing the gene sets obtained from these five data sources, we consolidated 827 human CPGs and retrieved their orthologs in rat and mouse using orthology data downloaded from HomoloGene (http://www.ncbi.nlm.nih.gov/homologene) and Mouse Genome Informatics (MGI) (http://www.informatics.jax.org).

### Data mining of human CPGs

To better understand the function of these CPGs in our dbCPG database, we retrieved comprehensive functional information from different public resources (Table 1). The basic gene information is included, such as gene ID, official symbol, official full name, synonym, position, gene type and OMIM ID from Entrez gene database and cancer syndrome, major associated type, mechanism of action of CPG mutations, and mode of inheritance from PubMed abstracts. Literature evidences were also provided. In addition, we provided functional information, including gene expression, methylation, post transcriptional modification, germline mutation, protein-protein interaction, pathway, and drug information (Figure 1). Details of these databases can be found through the cited references as well as from dbCPG.

To assess the function of 724 protein-coding CPGs, we explored the functional enrichment analysis by using two online tools, KOBAS [23] and DAVID [24]. KOBAS was used to analyze the pathway and disease, while DAVID was used to identify enriched biological themes (GO terms) and protein functional domains (InterPro terms) [25]. Then, we obtained those enriched functional terms with adjusted *P*-value less than 0.05. Furthermore, to investigate the importance of each protein coding CPG, we performed gene prioritization using ToppGene [29]. According to the number of literature evidences, 724 protein-coding CPGs were divided into two categories, 57 genes with at least 10 literature evidences acting as training gene set, the remaining 667 genes as test gene set, and finally, Klein-Ravi algorithm in GeneRev [30] was used to search the enriched dense modules.

### Database construction

We stored all the dbCPGs, annotations and related data by using MySQL (version 5.1.73), which is a popular open source and freely available database. A user-friendly web interface for browsing and searching was created using Java Server Pages technology. The database structure was shown in Figure 1, which is a systematical and detailed presentation of dbCPG.

dbCPG supports text query. In the home page, users can find a quick search box on the left to search by gene official symbol and gene ID. An advanced search option in search page is provided to search CPG related information, including the gene ID, gene official symbol, full name, gene type and genomic location. In addition, a search interface to access CPGs related literature provided a window for users to find more comprehensive CPG descriptions from original literature sources.

In Browser page, users can browse CPGs using genomic location, data source, gene type, species or cancer type (Figure 1). Using the chromosome browser, users can obtain a summary of the CPG lists. Clicking on the hyperlinks of the gene ID, users can access corresponding gene evidence and annotation pages. In each browser page, users can click on the hyperlinks of the specific data source, gene type, species or cancer type to obtain the corresponding CPG lists.

Aside from data retrieval from dbCPG, users are encouraged to upload additional publication information to the websites. Users may first search the dbCPG database to check if their publication has already been deposited into the database. If not, users may upload the related publication information, which will be stored in dbCPG. The new record will be forwarded to the dbCPG developer via email and will become available after a manual check and confirmation.

## SUPPLEMENTARY MATERIALS FIGURE AND TABLES




