# Supplementary material for: dbCPG: A web resource for cancer predisposition genes
Source: Oncotarget. 2016 May 12;7(25):37803–11. doi: 10.18632/oncotarget.9334 (PMC5122350; doi:10.18632/oncotarget.9334)
Supplement: Supplementary file 1 [file oncotarget-07-37803-s001.pdf]

dbCPG: A web resource for cancer predisposition genes

Supplementary Materials

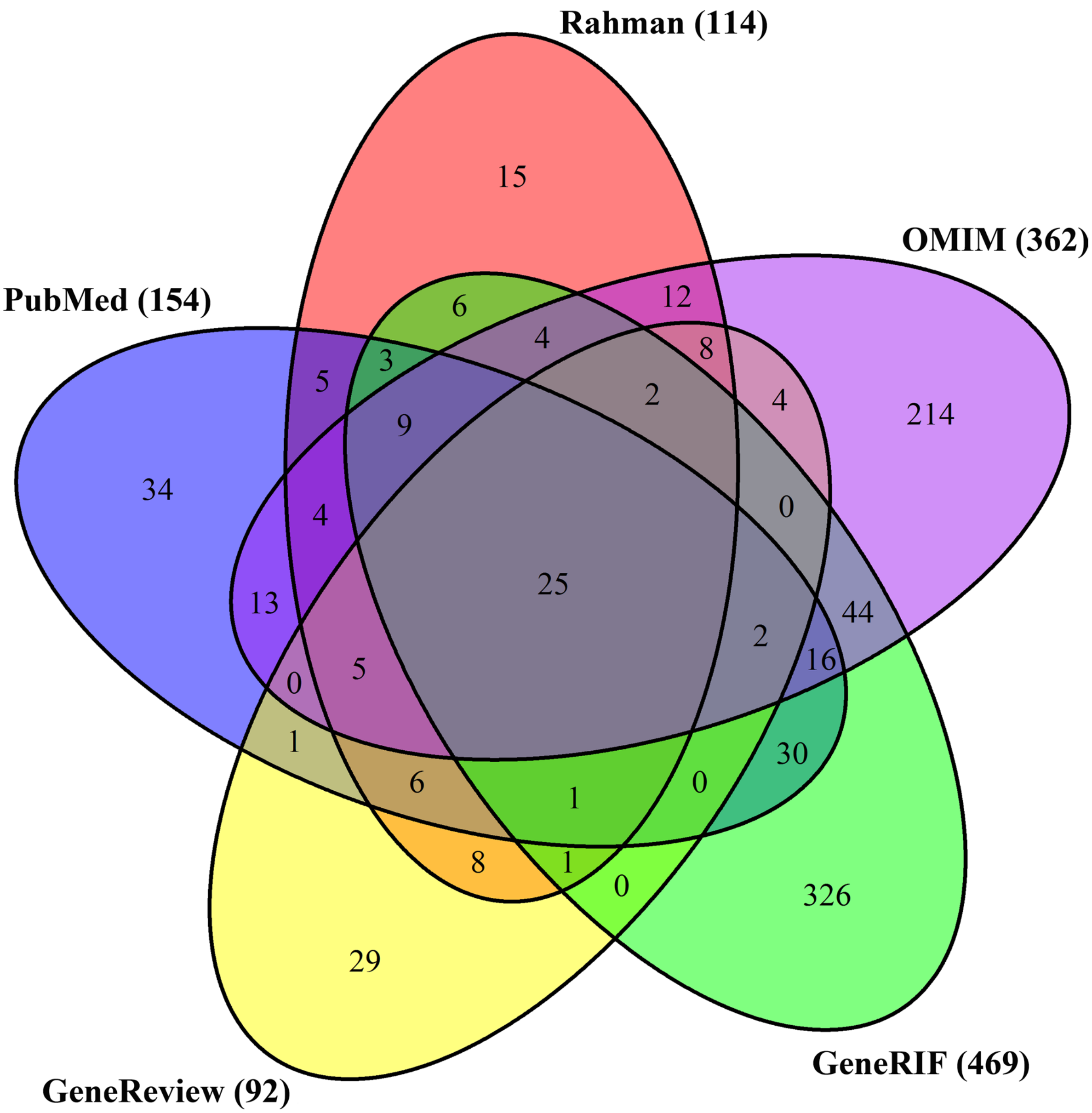

Supplementary Figure S1: Overlap of cancer predisposition genes in five data sources.

Supplementarey Table S1: The information of 724 human protein-coding CPGs

Gene ID	Official symbol	Official full name	Gene type
9	NAT1	N-acetyltransferase 1 (arylamine N-acetyltransferase)	protein-coding
10	NAT2	N-acetyltransferase 2 (arylamine N-acetyltransferase)	protein-coding
25	ABL1	ABL proto-oncogene 1, non-receptor tyrosine kinase	protein-coding
27	ABL2	ABL proto-oncogene 2, non-receptor tyrosine kinase	protein-coding
31	ACACA	acetyl-CoA carboxylase alpha	protein-coding
59	ACTA2	actin, alpha 2, smooth muscle, aorta	protein-coding
91	ACVR1B	activin A receptor, type IB	protein-coding
118	ADD1	adducin 1 (alpha)	protein-coding
125	ADH1B	alcohol dehydrogenase 1B (class I), beta polypeptide	protein-coding
126	ADH1C	alcohol dehydrogenase 1C (class I), gamma polypeptide	protein-coding
131	ADH7	alcohol dehydrogenase 7 (class IV), mu or sigma polypeptide	protein-coding
142	PARP1	poly (ADP-ribose) polymerase 1	protein-coding
155	ADRB3	adrenoceptor beta 3	protein-coding
177	AGER	advanced glycosylation end product-specific receptor	protein-coding
207	AKT1	v-akt murine thymoma viral oncogene homolog 1	protein-coding
217	ALDH2	aldehyde dehydrogenase 2 family (mitochondrial)	protein-coding
238	ALK	anaplastic lymphoma receptor tyrosine kinase	protein-coding
239	ALOX12	arachidonate 12-lipoxygenase	protein-coding
324	APC	adenomatous polyposis coli	protein-coding
328	APEX1	APEX nuclease (multifunctional DNA repair enzyme) 1	protein-coding
331	XIAP	X-linked inhibitor of apoptosis, E3 ubiquitin protein ligase	protein-coding
332	BIRC5	baculoviral IAP repeat containing 5	protein-coding
355	FAS	fas cell surface death receptor	protein-coding
356	FASLG	fas ligand (TNF superfamily, member 6)	protein-coding
367	AR	androgen receptor	protein-coding
405	ARNT	aryl hydrocarbon receptor nuclear translocator	protein-coding
463	ZFHX3	zinc finger homeobox 3	protein-coding
472	ATM	ATM serine/threonine kinase	protein-coding
488	ATP2A2	ATPase, Ca++ transporting, cardiac muscle, slow twitch 2	protein-coding
489	ATP2A3	ATPase, Ca++ transporting, ubiquitous	protein-coding
545	ATR	ATR serine/threonine kinase	protein-coding
571	BACH1	BTB and CNC homology 1, basic leucine zipper transcription factor 1	protein-coding
573	BAG1	BCL2-associated athanogene	protein-coding
580	BARD1	BRCA1 associated RING domain 1	protein-coding
581	BAX	BCL2-associated X protein	protein-coding
595	CCND1	cyclin D1	protein-coding
596	BCL2	B-cell CLL/lymphoma 2	protein-coding
602	BCL3	B-cell CLL/lymphoma 3	protein-coding
604	BCL6	B-cell CLL/lymphoma 6	protein-coding
605	BCL7A	B-cell CLL/lymphoma 7A	protein-coding
613	BCR	breakpoint cluster region	protein-coding
641	BLM	bloom syndrome, RecQ helicase-like	protein-coding
650	BMP2	bone morphogenetic protein 2	protein-coding
652	BMP4	bone morphogenetic protein 4	protein-coding
657	BMPR1A	bone morphogenetic protein receptor, type IA	protein-coding
658	BMPR1B	bone morphogenetic protein receptor, type IB	protein-coding

672	BRCA1	breast cancer 1, early onset	protein-coding
673	BRAF	B-Raf proto-oncogene, serine/threonine kinase	protein-coding
675	BRCA2	breast cancer 2, early onset	protein-coding
699	BUB1	BUB1 mitotic checkpoint serine/threonine kinase	protein-coding
701	BUB1B	BUB1 mitotic checkpoint serine/threonine kinase B	protein-coding
811	CALR	calreticulin	protein-coding
836	CASP3	caspase 3, apoptosis-related cysteine peptidase	protein-coding
838	CASP5	caspase 5, apoptosis-related cysteine peptidase	protein-coding
840	CASP7	caspase 7, apoptosis-related cysteine peptidase	protein-coding
841	CASP8	caspase 8, apoptosis-related cysteine peptidase	protein-coding
842	CASP9	caspase 9, apoptosis-related cysteine peptidase	protein-coding
843	CASP10	caspase 10, apoptosis-related cysteine peptidase	protein-coding
846	CASR	calcium-sensing receptor	protein-coding
857	CAV1	caveolin 1, caveolae protein, 22kDa	protein-coding
861	RUNX1	runt-related transcription factor 1	protein-coding
864	RUNX3	runt-related transcription factor 3	protein-coding
865	CBFB	core-binding factor, beta subunit	protein-coding
867	CBL	Cbl proto-oncogene, E3 ubiquitin protein ligase	protein-coding
898	CCNE1	cyclin E1	protein-coding
929	CD14	CD14 molecule	protein-coding
940	CD28	CD28 molecule	protein-coding
941	CD80	CD80 molecule	protein-coding
942	CD86	CD86 molecule	protein-coding
944	TNFSF8	tumor necrosis factor (ligand) superfamily, member 8	protein-coding
960	CD44	CD44 molecule (Indian blood group)	protein-coding
999	CDH1	cadherin 1, type 1, E-cadherin (epithelial)	protein-coding
1012	CDH13	cadherin 13	protein-coding
1015	CDH17	cadherin 17, LI cadherin (liver-intestine)	protein-coding
1017	CDK2	cyclin-dependent kinase 2	protein-coding
1019	CDK4	cyclin-dependent kinase 4	protein-coding
1020	CDK5	cyclin-dependent kinase 5	protein-coding
1026	CDKN1A	cyclin-dependent kinase inhibitor 1A (p21, Cip1)	protein-coding
1027	CDKN1B	cyclin-dependent kinase inhibitor 1B (p27, Kip1)	protein-coding
1028	CDKN1C	cyclin-dependent kinase inhibitor 1C (p57, Kip2)	protein-coding
1029	CDKN2A	cyclin-dependent kinase inhibitor 2A	protein-coding
1030	CDKN2B	cyclin-dependent kinase inhibitor 2B (p15, inhibits CDK4)	protein-coding
1050	CEBPA	CCAAT/enhancer binding protein (C/EBP), alpha	protein-coding
1080	CFTR	cystic fibrosis transmembrane conductance regulator (ATP-binding cassette sub-family C, member 7)	protein-coding
1136	CHRNA3	cholinergic receptor, nicotinic, alpha 3 (neuronal)	protein-coding
1138	CHRNA5	cholinergic receptor, nicotinic, alpha 5 (neuronal)	protein-coding
1142	CHRNB3	cholinergic receptor, nicotinic, beta 3 (neuronal)	protein-coding
1143	CHRNB4	cholinergic receptor, nicotinic, beta 4 (neuronal)	protein-coding
1200	TPP1	tripeptidyl peptidase I	protein-coding
1234	CCR5	chemokine (C-C motif) receptor 5 (gene/pseudogene)	protein-coding
1294	COL7A1	collagen, type VII, alpha 1	protein-coding
1312	COMT	catechol-O-methyltransferase	protein-coding
1316	KLF6	kruppel-like factor 6	protein-coding

1326	MAP3K8	mitogen-activated protein kinase kinase kinase 8	protein-coding
1356	CP	ceruloplasmin (ferroxidase)	protein-coding
1378	CR1	complement component (3b/4b) receptor 1 (Knops blood group)	protein-coding
1380	CR2	complement component (3d/Epstein Barr virus) receptor 2	protein-coding
1385	CREB1	cAMP responsive element binding protein 1	protein-coding
1386	ATF2	activating transcription factor 2	protein-coding
1387	CREBBP	CREB binding protein	protein-coding
1401	CRP	C-reactive protein, pentraxin-related	protein-coding
1434	CSE1L	CSE1 chromosome segregation 1-like (yeast)	protein-coding
1487	CTBP1	C-terminal binding protein 1	protein-coding
1493	CTLA4	cytotoxic T-lymphocyte-associated protein 4	protein-coding
1499	CTNNB1	catenin (cadherin-associated protein), beta 1, 88kDa	protein-coding
1540	CYLD	cylindromatosis (turban tumor syndrome)	protein-coding
1543	CYP1A1	cytochrome P450, family 1, subfamily A, polypeptide 1	protein-coding
1544	CYP1A2	cytochrome P450, family 1, subfamily A, polypeptide 2	protein-coding
1545	CYP1B1	cytochrome P450, family 1, subfamily B, polypeptide 1	protein-coding
1548	CYP2A6	cytochrome P450, family 2, subfamily A, polypeptide 6	protein-coding
1557	CYP2C19	cytochrome P450, family 2, subfamily C, polypeptide 19	protein-coding
1565	CYP2D6	cytochrome P450, family 2, subfamily D, polypeptide 6	protein-coding
1571	CYP2E1	cytochrome P450, family 2, subfamily E, polypeptide 1	protein-coding
1581	CYP7A1	cytochrome P450, family 7, subfamily A, polypeptide 1	protein-coding
1583	CYP11A1	cytochrome P450, family 11, subfamily A, polypeptide 1	protein-coding
1586	CYP17A1	cytochrome P450, family 17, subfamily A, polypeptide 1	protein-coding
1588	CYP19A1	cytochrome P450, family 19, subfamily A, polypeptide 1	protein-coding
1630	DCC	DCC netrin 1 receptor	protein-coding
1636	ACE	angiotensin I converting enzyme	protein-coding
1643	DDB2	damage-specific DNA binding protein 2, 48kDa	protein-coding
1647	GADD45A	growth arrest and DNA-damage-inducible, alpha	protein-coding
1649	DDIT3	DNA-damage-inducible transcript 3	protein-coding
1673	DEFB4A	defensin, beta 4A	protein-coding
1674	DES	desmin	protein-coding
1728	NQO1	NAD(P)H dehydrogenase, quinone 1	protein-coding
1736	DKC1	dyskeratosis congenita 1, dyskerin	protein-coding
1761	DMRT1	doublesex and mab-3 related transcription factor 1	protein-coding
1788	DNMT3A	DNA (cytosine-5-)-methyltransferase 3 alpha	protein-coding
1789	DNMT3B	DNA (cytosine-5-)-methyltransferase 3 beta	protein-coding
1829	DSG2	desmoglein 2	protein-coding
1942	EFNA1	ephrin-A1	protein-coding
1950	EGF	epidermal growth factor	protein-coding
1956	EGFR	epidermal growth factor receptor	protein-coding
1991	ELANE	elastase, neutrophil expressed	protein-coding
2023	ENO1	enolase 1, (alpha)	protein-coding
2033	EP300	E1A binding protein p300	protein-coding
2047	EPHB1	EPH receptor B1	protein-coding
2048	EPHB2	EPH receptor B2	protein-coding
2052	EPHX1	epoxide hydrolase 1, microsomal (xenobiotic)	protein-coding
2064	ERBB2	erb-b2 receptor tyrosine kinase 2	protein-coding

2067	ERCC1	excision repair cross-complementation group 1	protein-coding
2068	ERCC2	excision repair cross-complementation group 2	protein-coding
2071	ERCC3	excision repair cross-complementation group 3	protein-coding
2072	ERCC4	excision repair cross-complementation group 4	protein-coding
2073	ERCC5	excision repair cross-complementation group 5	protein-coding
2074	ERCC6	excision repair cross-complementation group 6	protein-coding
2099	ESR1	estrogen receptor 1	protein-coding
2100	ESR2	estrogen receptor 2 (ER beta)	protein-coding
2104	ESRRG	estrogen-related receptor gamma	protein-coding
2120	ETV6	ets variant 6	protein-coding
2130	EWSR1	EWS RNA-binding protein 1	protein-coding
2131	EXT1	exostosin glycosyltransferase 1	protein-coding
2132	EXT2	exostosin glycosyltransferase 2	protein-coding
2146	EZH2	enhancer of zeste 2 polycomb repressive complex 2 subunit	protein-coding
2175	FANCA	fanconi anemia, complementation group A	protein-coding
2176	FANCC	fanconi anemia, complementation group C	protein-coding
2177	FANCD2	fanconi anemia, complementation group D2	protein-coding
2184	FAH	fumarylacetoacetate hydrolase (fumarylacetoacetase)	protein-coding
2189	FANCG	fanconi anemia, complementation group G	protein-coding
2237	FEN1	flap structure-specific endonuclease 1	protein-coding
2239	GPC4	glypican 4	protein-coding
2261	FGFR3	fibroblast growth factor receptor 3	protein-coding
2262	GPC5	glypican 5	protein-coding
2263	FGFR2	fibroblast growth factor receptor 2	protein-coding
2264	FGFR4	fibroblast growth factor receptor 4	protein-coding
2271	FH	fumarate hydratase	protein-coding
2294	FOXF1	forkhead box F1	protein-coding
2304	FOXE1	forkhead box E1	protein-coding
2308	FOXO1	forkhead box O1	protein-coding
2321	FLT1	fms-related tyrosine kinase 1	protein-coding
2322	FLT3	fms-related tyrosine kinase 3	protein-coding
2324	FLT4	fms-related tyrosine kinase 4	protein-coding
2348	FOLR1	folate receptor 1 (adult)	protein-coding
2475	MTOR	mechanistic target of rapamycin (serine/threonine kinase)	protein-coding
2531	KDSR	3-ketodihydrosphingosine reductase	protein-coding
2547	XRCC6	X-ray repair complementing defective repair in Chinese hamster cells 6	protein-coding
2549	GAB1	GRB2-associated binding protein 1	protein-coding
2623	GATA1	GATA binding protein 1 (globin transcription factor 1)	protein-coding
2624	GATA2	GATA binding protein 2	protein-coding
2629	GBA	glucosidase, beta, acid	protein-coding
2650	GCNT1	glucosaminyl (N-acetyl) transferase 1, core 2	protein-coding
2668	GDNF	glial cell derived neurotrophic factor	protein-coding
2706	GJB2	gap junction protein, beta 2, 26kDa	protein-coding
2719	GPC3	glypican 3	protein-coding
2771	GNAI2	guanine nucleotide binding protein (G protein), alpha inhibiting activity polypeptide 2	protein-coding
2776	GNAQ	guanine nucleotide binding protein (G protein), q polypeptide	protein-coding
2784	GNB3	guanine nucleotide binding protein (G protein), beta polypeptide 3	protein-coding

2876	GPX1	glutathione peroxidase 1	protein-coding
2935	GSPT1	G1 to S phase transition 1	protein-coding
2938	GSTA1	glutathione S-transferase alpha 1	protein-coding
2944	GSTM1	glutathione S-transferase mu 1	protein-coding
2947	GSTM3	glutathione S-transferase mu 3 (brain)	protein-coding
2950	GSTP1	glutathione S-transferase pi 1	protein-coding
2952	GSTT1	glutathione S-transferase theta 1	protein-coding
2956	MSH6	mutS homolog 6	protein-coding
2965	GTF2H1	general transcription factor IIH, polypeptide 1, 62kDa	protein-coding
3077	HFE	hemochromatosis	protein-coding
3091	HIF1A	hypoxia inducible factor 1, alpha subunit (basic helix-loop-helix transcription factor)	protein-coding
3092	HIP1	huntingtin interacting protein 1	protein-coding
3105	HLA-A	major histocompatibility complex, class I, A	protein-coding
3106	HLA-B	major histocompatibility complex, class I, B	protein-coding
3115	HLA-DPB1	major histocompatibility complex, class II, DP beta 1	protein-coding
3117	HLA-DQA1	major histocompatibility complex, class II, DQ alpha 1	protein-coding
3119	HLA-DQB1	major histocompatibility complex, class II, DQ beta 1	protein-coding
3123	HLA-DRB1	major histocompatibility complex, class II, DR beta 1	protein-coding
3135	HLA-G	major histocompatibility complex, class I, G	protein-coding
3145	HMBS	hydroxymethylbilane synthase	protein-coding
3161	HMMR	hyaluronan-mediated motility receptor (RHAMM)	protein-coding
3162	HMOX1	heme oxygenase 1	protein-coding
3249	HPN	hepsin	protein-coding
3265	HRAS	harvey rat sarcoma viral oncogene homolog	protein-coding
3266	ERAS	ES cell expressed Ras	protein-coding
3284	HSD3B2	hydroxy-delta-5-steroid dehydrogenase, 3 beta- and steroid delta-isomerase 2	protein-coding
3294	HSD17B2	hydroxysteroid (17-beta) dehydrogenase 2	protein-coding
3304	HSPA1B	heat shock 70kDa protein 1B	protein-coding
3383	ICAM1	intercellular adhesion molecule 1	protein-coding
3417	IDH1	isocitrate dehydrogenase 1 (NADP+), soluble	protein-coding
3451	IFNA17	interferon, alpha 17	protein-coding
3458	IFNG	interferon, gamma	protein-coding
3479	IGF1	insulin-like growth factor 1 (somatomedin C)	protein-coding
3481	IGF2	insulin-like growth factor 2	protein-coding
3482	IGF2R	insulin-like growth factor 2 receptor	protein-coding
3486	IGFBP3	insulin-like growth factor binding protein 3	protein-coding
3488	IGFBP5	insulin-like growth factor binding protein 5	protein-coding
3491	CYR61	cysteine-rich, angiogenic inducer, 61	protein-coding
3553	IL1B	interleukin 1, beta	protein-coding
3557	IL1RN	interleukin 1 receptor antagonist	protein-coding
3558	IL2	interleukin 2	protein-coding
3565	IL4	interleukin 4	protein-coding
3569	IL6	interleukin 6	protein-coding
3574	IL7	interleukin 7	protein-coding
3576	CXCL8	chemokine (C-X-C motif) ligand 8	protein-coding
3586	IL10	interleukin 10	protein-coding

3592	IL12A	interleukin 12A	protein-coding
3593	IL12B	interleukin 12B	protein-coding
3594	IL12RB1	interleukin 12 receptor, beta 1	protein-coding
3596	IL13	interleukin 13	protein-coding
3601	IL15RA	interleukin 15 receptor, alpha	protein-coding
3603	IL16	interleukin 16	protein-coding
3605	IL17A	interleukin 17A	protein-coding
3606	IL18	interleukin 18	protein-coding
3621	ING1	inhibitor of growth family, member 1	protein-coding
3659	IRF1	interferon regulatory factor 1	protein-coding
3662	IRF4	interferon regulatory factor 4	protein-coding
3667	IRS1	insulin receptor substrate 1	protein-coding
3673	ITGA2	integrin, alpha 2 (CD49B, alpha 2 subunit of VLA-2 receptor)	protein-coding
3680	ITGA9	integrin, alpha 9	protein-coding
3702	ITK	IL2-inducible T-cell kinase	protein-coding
3717	JAK2	janus kinase 2	protein-coding
3725	JUN	jun proto-oncogene	protein-coding
3732	CD82	CD82 molecule	protein-coding
3784	KCNQ1	potassium channel, voltage gated KQT-like subfamily Q, member 1	protein-coding
3791	KDR	kinase insert domain receptor	protein-coding
3815	KIT	v-kit Hardy-Zuckerman 4 feline sarcoma viral oncogene homolog	protein-coding
3817	KLK2	kallikrein-related peptidase 2	protein-coding
3845	KRAS	kirsten rat sarcoma viral oncogene homolog	protein-coding
3872	KRT17	keratin 17, type I	protein-coding
3897	L1CAM	L1 cell adhesion molecule	protein-coding
3921	RPSA	ribosomal protein SA	protein-coding
3929	LBP	lipopolysaccharide binding protein	protein-coding
3953	LEPR	leptin receptor	protein-coding
3958	LGALS3	lectin, galactoside-binding, soluble, 3	protein-coding
3972	LHB	luteinizing hormone beta polypeptide	protein-coding
3973	LHCGR	luteinizing hormone/choriogonadotropin receptor	protein-coding
3981	LIG4	ligase IV, DNA, ATP-dependent	protein-coding
4004	LMO1	LIM domain only 1 (rhombotin 1)	protein-coding
4005	LMO2	LIM domain only 2 (rhombotin-like 1)	protein-coding
4015	LOX	lysyl oxidase	protein-coding
4026	LPP	LIM domain containing preferred translocation partner in lipoma	protein-coding
4041	LRP5	low density lipoprotein receptor-related protein 5	protein-coding
4049	LTA	lymphotoxin alpha	protein-coding
4066	LYL1	lymphoblastic leukemia associated hematopoiesis regulator 1	protein-coding
4068	SH2D1A	SH2 domain containing 1A	protein-coding
4072	EPCAM	epithelial cell adhesion molecule	protein-coding
4085	MAD2L1	MAD2 mitotic arrest deficient-like 1 (yeast)	protein-coding
4088	SMAD3	SMAD family member 3	protein-coding
4089	SMAD4	SMAD family member 4	protein-coding
4092	SMAD7	SMAD family member 7	protein-coding
4149	MAX	MYC associated factor X	protein-coding
4153	MBL2	mannose-binding lectin (protein C) 2, soluble	protein-coding

4157	MC1R	melanocortin 1 receptor (alpha melanocyte stimulating hormone receptor)	protein-coding
4163	MCC	mutated in colorectal cancers	protein-coding
4191	MDH2	malate dehydrogenase 2, NAD (mitochondrial)	protein-coding
4193	MDM2	MDM2 proto-oncogene, E3 ubiquitin protein ligase	protein-coding
4210	MEFV	mediterranean fever	protein-coding
4214	MAP3K1	mitogen-activated protein kinase kinase kinase 1, E3 ubiquitin protein ligase	protein-coding
4221	MEN1	multiple endocrine neoplasia I	protein-coding
4233	MET	MET proto-oncogene, receptor tyrosine kinase	protein-coding
4254	KITLG	KIT ligand	protein-coding
4255	MGMT	O-6-methylguanine-DNA methyltransferase	protein-coding
4282	MIF	macrophage migration inhibitory factor (glycosylation-inhibiting factor)	protein-coding
4286	MITF	microphthalmia-associated transcription factor	protein-coding
4291	MLF1	myeloid leukemia factor 1	protein-coding
4292	MLH1	mutL homolog 1	protein-coding
4297	KMT2A	lysine (K)-specific methyltransferase 2A	protein-coding
4312	MMP1	matrix metallopeptidase 1	protein-coding
4313	MMP2	matrix metallopeptidase 2	protein-coding
4314	MMP3	matrix metallopeptidase 3	protein-coding
4316	MMP7	matrix metallopeptidase 7	protein-coding
4318	MMP9	matrix metallopeptidase 9	protein-coding
4323	MMP14	matrix metallopeptidase 14 (membrane-inserted)	protein-coding
4330	MN1	meningioma (disrupted in balanced translocation) 1	protein-coding
4353	MPO	myeloperoxidase	protein-coding
4361	MRE11A	MRE11 meiotic recombination 11 homolog A (S. cerevisiae)	protein-coding
4363	ABCC1	ATP-binding cassette, sub-family C (CFTR/MRP), member 1	protein-coding
4436	MSH2	mutS homolog 2	protein-coding
4437	MSH3	mutS homolog 3	protein-coding
4477	MSMB	microseminoprotein, beta-	protein-coding
4481	MSR1	macrophage scavenger receptor 1	protein-coding
4507	MTAP	methylthioadenosine phosphorylase	protein-coding
4524	MTHFR	methylenetetrahydrofolate reductase (NAD(P)H)	protein-coding
4543	MTNR1A	melatonin receptor 1A	protein-coding
4544	MTNR1B	melatonin receptor 1B	protein-coding
4548	MTR	5-methyltetrahydrofolate-homocysteine methyltransferase	protein-coding
4552	MTRR	5-methyltetrahydrofolate-homocysteine methyltransferase reductase	protein-coding
4582	MUC1	mucin 1, cell surface associated	protein-coding
4586	MUC5AC	mucin 5AC, oligomeric mucus/gel-forming	protein-coding
4591	TRIM37	tripartite motif containing 37	protein-coding
4595	MUTYH	mutY homolog	protein-coding
4601	MXI1	MAX interactor 1, dimerization protein	protein-coding
4602	MYB	v-myb avian myeloblastosis viral oncogene homolog	protein-coding
4609	MYC	v-myc avian myelocytomatosis viral oncogene homolog	protein-coding
4610	MYCL	v-myc avian myelocytomatosis viral oncogene lung carcinoma derived homolog	protein-coding
4629	MYH11	myosin, heavy chain 11, smooth muscle	protein-coding

4638	MYLK	myosin light chain kinase	protein-coding
4683	NBN	nibrin	protein-coding
4751	NEK2	NIMA-related kinase 2	protein-coding
4763	NF1	neurofibromin 1	protein-coding
4771	NF2	neurofibromin 2 (merlin)	protein-coding
4790	NFKB1	nuclear factor of kappa light polypeptide gene enhancer in B-cells 1	protein-coding
4824	NKX3-1	NK3 homeobox 1	protein-coding
4830	NME1	NME/NM23 nucleoside diphosphate kinase 1	protein-coding
4835	NQO2	NAD(P)H dehydrogenase, quinone 2	protein-coding
4843	NOS2	nitric oxide synthase 2, inducible	protein-coding
4846	NOS3	nitric oxide synthase 3 (endothelial cell)	protein-coding
4864	NPC1	niemann-pick disease, type C1	protein-coding
4869	NPM1	nucleophosmin (nucleolar phosphoprotein B23, numatrin)	protein-coding
4893	NRAS	neuroblastoma RAS viral (v-ras) oncogene homolog	protein-coding
4904	YBX1	Y box binding protein 1	protein-coding
4914	NTRK1	neurotrophic tyrosine kinase, receptor, type 1	protein-coding
4926	NUMA1	nuclear mitotic apparatus protein 1	protein-coding
4953	ODC1	ornithine decarboxylase 1	protein-coding
4968	OGG1	8-oxoguanine DNA glycosylase	protein-coding
4978	OPCML	opioid binding protein/cell adhesion molecule-like	protein-coding
4982	TNFRSF11B	tumor necrosis factor receptor superfamily, member 11b	protein-coding
5002	SLC22A18	solute carrier family 22, member 18	protein-coding
5054	SERPINE1	serpin peptidase inhibitor, clade E (nexin, plasminogen activator inhibitor type 1), member 1	protein-coding
5071	PARK2	parkin RBR E3 ubiquitin protein ligase	protein-coding
5077	PAX3	paired box 3	protein-coding
5079	PAX5	paired box 5	protein-coding
5081	PAX7	paired box 7	protein-coding
5087	PBX1	pre-B-cell leukemia homeobox 1	protein-coding
5106	PCK2	phosphoenolpyruvate carboxykinase 2 (mitochondrial)	protein-coding
5108	PCM1	pericentriolar material 1	protein-coding
5133	PDCD1	programmed cell death 1	protein-coding
5155	PDGFB	platelet-derived growth factor beta polypeptide	protein-coding
5156	PDGFRA	platelet-derived growth factor receptor, alpha polypeptide	protein-coding
5157	PDGFRL	platelet-derived growth factor receptor-like	protein-coding
5225	PGC	progastricsin (pepsinogen C)	protein-coding
5241	PGR	progesterone receptor	protein-coding
5243	ABCB1	ATP-binding cassette, sub-family B (MDR/TAP), member 1	protein-coding
5245	PHB	prohibitin	protein-coding
5265	SERPINA1	serpin peptidase inhibitor, clade A (alpha-1 antiproteinase, antitrypsin), member 1	protein-coding
5290	PIK3CA	phosphatidylinositol-4,5-bisphosphate 3-kinase, catalytic subunit alpha	protein-coding
5293	PIK3CD	phosphatidylinositol-4,5-bisphosphate 3-kinase, catalytic subunit delta	protein-coding
5300	PIN1	peptidylprolyl cis/trans isomerase, NIMA-interacting 1	protein-coding
5320	PLA2G2A	phospholipase A2, group IIA (platelets, synovial fluid)	protein-coding
5338	PLD2	phospholipase D2	protein-coding
5371	PML	promyelocytic leukemia	protein-coding
5395	PMS2	PMS2 postmeiotic segregation increased 2 (S. cerevisiae)	protein-coding

5423	POLB	polymerase (DNA directed), beta	protein-coding
5424	POLD1	polymerase (DNA directed), delta 1, catalytic subunit	protein-coding
5426	POLE	polymerase (DNA directed), epsilon, catalytic subunit	protein-coding
5429	POLH	polymerase (DNA directed), eta	protein-coding
5444	PON1	paraoxonase 1	protein-coding
5460	POU5F1	POU class 5 homeobox 1	protein-coding
5462	POU5F1B	POU class 5 homeobox 1B	protein-coding
5468	PPARG	peroxisome proliferator-activated receptor gamma	protein-coding
5519	PPP2R1B	protein phosphatase 2, regulatory subunit A, beta	protein-coding
5520	PPP2R2A	protein phosphatase 2, regulatory subunit B, alpha	protein-coding
5526	PPP2R5B	protein phosphatase 2, regulatory subunit B', beta	protein-coding
5546	PRCC	papillary renal cell carcinoma (translocation-associated)	protein-coding
5551	PRF1	perforin 1 (pore forming protein)	protein-coding
5562	PRKAA1	protein kinase, AMP-activated, alpha 1 catalytic subunit	protein-coding
5573	PRKAR1A	protein kinase, cAMP-dependent, regulatory, type I, alpha	protein-coding
5578	PRKCA	protein kinase C, alpha	protein-coding
5591	PRKDC	protein kinase, DNA-activated, catalytic polypeptide	protein-coding
5644	PRSS1	protease, serine, 1 (trypsin 1)	protein-coding
5727	PTCH1	patched 1	protein-coding
5728	PTEN	phosphatase and tensin homolog	protein-coding
5734	PTGER4	prostaglandin E receptor 4 (subtype EP4)	protein-coding
5740	PTGIS	prostaglandin I2 (prostacyclin) synthase	protein-coding
5743	PTGS2	prostaglandin-endoperoxide synthase 2 (prostaglandin G/H synthase and cyclooxygenase)	protein-coding
5744	PTHLH	parathyroid hormone-like hormone	protein-coding
5781	PTPN11	protein tyrosine phosphatase, non-receptor type 11	protein-coding
5782	PTPN12	protein tyrosine phosphatase, non-receptor type 12	protein-coding
5783	PTPN13	protein tyrosine phosphatase, non-receptor type 13 (APO-1/CD95 (Fas)-associated phosphatase)	protein-coding
5790	PTPRCAP	protein tyrosine phosphatase, receptor type, C-associated protein	protein-coding
5795	PTPRJ	protein tyrosine phosphatase, receptor type, J	protein-coding
5888	RAD51	RAD51 recombinase	protein-coding
5889	RAD51C	RAD51 paralog C	protein-coding
5892	RAD51D	RAD51 paralog D	protein-coding
5910	RAP1GDS1	RAP1, GTP-GDP dissociation stimulator 1	protein-coding
5914	RARA	retinoic acid receptor, alpha	protein-coding
5921	RASA1	RAS p21 protein activator (GTPase activating protein) 1	protein-coding
5925	RB1	retinoblastoma 1	protein-coding
5932	RBBP8	retinoblastoma binding protein 8	protein-coding
5979	RET	ret proto-oncogene	protein-coding
5980	REV3L	REV3-like, polymerase (DNA directed), zeta, catalytic subunit	protein-coding
6041	RNASEL	ribonuclease L (2',5'-oligoisoadenylate synthetase-dependent)	protein-coding
6049	RNF6	ring finger protein (C3H2C3 type) 6	protein-coding
6224	RPS20	ribosomal protein S20	protein-coding
6288	SAA1	serum amyloid A1	protein-coding
6318	SERPINB4	serpin peptidase inhibitor, clade B (ovalbumin), member 4	protein-coding
6347	CCL2	chemokine (C-C motif) ligand 2	protein-coding
6352	CCL5	chemokine (C-C motif) ligand 5	protein-coding

6387	CXCL12	chemokine (C-X-C motif) ligand 12	protein-coding
6389	SDHA	succinate dehydrogenase complex, subunit A, flavoprotein (Fp)	protein-coding
6390	SDHB	succinate dehydrogenase complex, subunit B, iron sulfur (Ip)	protein-coding
6391	SDHC	succinate dehydrogenase complex, subunit C, integral membrane protein, 15kDa	protein-coding
6392	SDHD	succinate dehydrogenase complex, subunit D, integral membrane protein	protein-coding
6401	SELE	selectin E	protein-coding
6455	SH3GL1	SH3-domain GRB2-like 1	protein-coding
6462	SHBG	sex hormone-binding globulin	protein-coding
6556	SLC11A1	solute carrier family 11 (proton-coupled divalent metal ion transporter), member 1	protein-coding
6563	SLC14A1	solute carrier family 14 (urea transporter), member 1 (Kidd blood group)	protein-coding
6595	SMARCA2	SWI/SNF related, matrix associated, actin dependent regulator of chromatin, subfamily a, member 2	protein-coding
6597	SMARCA4	SWI/SNF related, matrix associated, actin dependent regulator of chromatin, subfamily a, member 4	protein-coding
6598	SMARCB1	SWI/SNF related, matrix associated, actin dependent regulator of chromatin, subfamily b, member 1	protein-coding
6605	SMARCE1	SWI/SNF related, matrix associated, actin dependent regulator of chromatin, subfamily e, member 1	protein-coding
6608	SMO	smoothened, frizzled class receptor	protein-coding
6648	SOD2	superoxide dismutase 2, mitochondrial	protein-coding
6654	SOS1	son of sevenless homolog 1 (Drosophila)	protein-coding
6678	SPARC	secreted protein, acidic, cysteine-rich (osteonectin)	protein-coding
6690	SPINK1	serine peptidase inhibitor, Kazal type 1	protein-coding
6696	SPP1	secreted phosphoprotein 1	protein-coding
6714	SRC	SRC proto-oncogene, non-receptor tyrosine kinase	protein-coding
6736	SRY	sex determining region Y	protein-coding
6756	SSX1	synovial sarcoma, X breakpoint 1	protein-coding
6757	SSX2	synovial sarcoma, X breakpoint 2	protein-coding
6772	STAT1	signal transducer and activator of transcription 1, 91kDa	protein-coding
6774	STAT3	signal transducer and activator of transcription 3 (acute-phase response factor)	protein-coding
6775	STAT4	signal transducer and activator of transcription 4	protein-coding
6777	STAT5B	signal transducer and activator of transcription 5B	protein-coding
6782	HSPA13	heat shock protein 70kDa family, member 13	protein-coding
6790	AURKA	aurora kinase A	protein-coding
6794	STK11	serine/threonine kinase 11	protein-coding
6804	STX1A	syntaxin 1A (brain)	protein-coding
6813	STXBP2	syntaxin binding protein 2	protein-coding
6817	SULT1A1	sulfotransferase family, cytosolic, 1A, phenol-preferring, member 1	protein-coding
6886	TAL1	T-cell acute lymphocytic leukemia 1	protein-coding
6887	TAL2	T-cell acute lymphocytic leukemia 2	protein-coding
6927	HNF1A	HNF1 homeobox A	protein-coding
6928	HNF1B	HNF1 homeobox B	protein-coding
6929	TCF3	transcription factor 3	protein-coding
6948	TCN2	transcobalamin II	protein-coding

7001	PRDX2	peroxiredoxin 2	protein-coding
7015	TERT	telomerase reverse transcriptase	protein-coding
7030	TFE3	transcription factor binding to IGHM enhancer 3	protein-coding
7036	TFR2	transferrin receptor 2	protein-coding
7040	TGFB1	transforming growth factor, beta 1	protein-coding
7046	TGFBR1	transforming growth factor, beta receptor 1	protein-coding
7048	TGFBR2	transforming growth factor, beta receptor II (70/80kDa)	protein-coding
7049	TGFBR3	transforming growth factor, beta receptor III	protein-coding
7053	TGM3	transglutaminase 3	protein-coding
7057	THBS1	thrombospondin 1	protein-coding
7077	TIMP2	TIMP metallopeptidase inhibitor 2	protein-coding
7078	TIMP3	TIMP metallopeptidase inhibitor 3	protein-coding
7079	TIMP4	TIMP metallopeptidase inhibitor 4	protein-coding
7080	NKX2-1	NK2 homeobox 1	protein-coding
7087	ICAM5	intercellular adhesion molecule 5, telencephalin	protein-coding
7097	TLR2	toll-like receptor 2	protein-coding
7098	TLR3	toll-like receptor 3	protein-coding
7099	TLR4	toll-like receptor 4	protein-coding
7124	TNF	tumor necrosis factor	protein-coding
7127	TNFAIP2	tumor necrosis factor, alpha-induced protein 2	protein-coding
7157	TP53	tumor protein p53	protein-coding
7159	TP53BP2	tumor protein p53 binding protein 2	protein-coding
7161	TP73	tumor protein p73	protein-coding
7248	TSC1	tuberous sclerosis 1	protein-coding
7249	TSC2	tuberous sclerosis 2	protein-coding
7251	TSG101	tumor susceptibility 101	protein-coding
7253	TSHR	thyroid stimulating hormone receptor	protein-coding
7291	TWIST1	twist family bHLH transcription factor 1	protein-coding
7298	TYMS	thymidylate synthetase	protein-coding
7299	TYR	tyrosinase	protein-coding
7367	UGT2B17	UDP glucuronosyltransferase 2 family, polypeptide B17	protein-coding
7376	NR1H2	nuclear receptor subfamily 1, group H, member 2	protein-coding
7389	UROD	uroporphyrinogen decarboxylase	protein-coding
7421	VDR	vitamin D (1,25- dihydroxyvitamin D3) receptor	protein-coding
7422	VEGFA	vascular endothelial growth factor A	protein-coding
7424	VEGFC	vascular endothelial growth factor C	protein-coding
7428	VHL	von Hippel-Lindau tumor suppressor, E3 ubiquitin protein ligase	protein-coding
7454	WAS	wiskott-aldrich syndrome	protein-coding
7486	WRN	werner syndrome, RecQ helicase-like	protein-coding
7490	WT1	wilms tumor 1	protein-coding
7507	XPA	xeroderma pigmentosum, complementation group A	protein-coding
7508	XPC	xeroderma pigmentosum, complementation group C	protein-coding
7515	XRCC1	X-ray repair complementing defective repair in Chinese hamster cells 1	protein-coding
7516	XRCC2	X-ray repair complementing defective repair in Chinese hamster cells 2	protein-coding
7517	XRCC3	X-ray repair complementing defective repair in Chinese hamster cells 3	protein-coding
7518	XRCC4	X-ray repair complementing defective repair in Chinese hamster cells 4	protein-coding
7520	XRCC5	X-ray repair complementing defective repair in Chinese hamster cells 5 (double-strand-break rejoining)	protein-coding

7704	ZBTB16	zinc finger and BTB domain containing 16	protein-coding
7799	PRDM2	PR domain containing 2, with ZNF domain	protein-coding
7849	PAX8	paired box 8	protein-coding
7852	CXCR4	chemokine (C-X-C motif) receptor 4	protein-coding
7913	DEK	DEK proto-oncogene	protein-coding
7916	PRRC2A	proline-rich coiled-coil 2A	protein-coding
8000	PSCA	prostate stem cell antigen	protein-coding
8013	NR4A3	nuclear receptor subfamily 4, group A, member 3	protein-coding
8021	NUP214	nucleoporin 214kDa	protein-coding
8028	MLLT10	myeloid/lymphoid or mixed-lineage leukemia (trithorax homolog, Drosophila); translocated to, 10	protein-coding
8030	CCDC6	coiled-coil domain containing 6	protein-coding
8031	NCOA4	nuclear receptor coactivator 4	protein-coding
8074	FGF23	fibroblast growth factor 23	protein-coding
8091	HMGA2	high mobility group AT-hook 2	protein-coding
8115	TCL1A	T-cell leukemia/lymphoma 1A	protein-coding
8148	TAF15	TAF15 RNA polymerase II, TATA box binding protein (TBP)-associated factor, 68kDa	protein-coding
8195	MKKS	mcKusick-kaufman syndrome	protein-coding
8202	NCOA3	nuclear receptor coactivator 3	protein-coding
8289	ARID1A	AT rich interactive domain 1A (SWI-like)	protein-coding
8301	PICALM	phosphatidylinositol binding clathrin assembly protein	protein-coding
8312	AXIN1	axin 1	protein-coding
8313	AXIN2	axin 2	protein-coding
8314	BAP1	BRCA1 associated protein-1 (ubiquitin carboxy-terminal hydrolase)	protein-coding
8379	MAD1L1	MAD1 mitotic arrest deficient-like 1 (yeast)	protein-coding
8434	RECK	reversion-inducing-cysteine-rich protein with kazal motifs	protein-coding
8438	RAD54L	RAD54-like (S. cerevisiae)	protein-coding
8493	PPM1D	protein phosphatase, Mg2+/Mn2+ dependent, 1D	protein-coding
8518	IKBKAP	inhibitor of kappa light polypeptide gene enhancer in B-cells, kinase complex-associated protein	protein-coding
8626	TP63	tumor protein p63	protein-coding
8643	PTCH2	patched 2	protein-coding
8647	ABCB11	ATP-binding cassette, sub-family B (MDR/TAP), member 11	protein-coding
8676	STX11	syntaxin 11	protein-coding
8771	TNFRSF6B	tumor necrosis factor receptor superfamily, member 6b, decoy	protein-coding
8795	TNFRSF10B	tumor necrosis factor receptor superfamily, member 10b	protein-coding
8797	TNFRSF10A	tumor necrosis factor receptor superfamily, member 10a	protein-coding
8805	TRIM24	tripartite motif containing 24	protein-coding
8826	IQGAP1	IQ motif containing GTPase activating protein 1	protein-coding
8833	GMPS	guanine monphosphate synthase	protein-coding
8864	PER2	period circadian clock 2	protein-coding
8895	CPNE3	copine III	protein-coding
8915	BCL10	B-cell CLL/lymphoma 10	protein-coding
8929	PHOX2B	paired-like homeobox 2b	protein-coding
8930	MBD4	methyl-CpG binding domain protein 4	protein-coding
8932	MBD2	methyl-CpG binding domain protein 2	protein-coding
8973	CHRNA6	cholinergic receptor, nicotinic, alpha 6 (neuronal)	protein-coding

9049	AIP	aryl hydrocarbon receptor interacting protein	protein-coding
9075	CLDN2	claudin 2	protein-coding
9156	EXO1	exonuclease 1	protein-coding
9235	IL32	interleukin 32	protein-coding
9258	MFHAS1	malignant fibrous histiocytoma amplified sequence 1	protein-coding
9262	STK17B	serine/threonine kinase 17b	protein-coding
9308	CD83	CD83 molecule	protein-coding
9370	ADIPOQ	adiponectin, C1Q and collagen domain containing	protein-coding
9401	RECQL4	RecQ protein-like 4	protein-coding
9429	ABCG2	ATP-binding cassette, sub-family G (WHITE), member 2 (Junior blood group)	protein-coding
9473	THEMIS2	thymocyte selection associated family member 2	protein-coding
9474	ATG5	autophagy related 5	protein-coding
9518	GDF15	growth differentiation factor 15	protein-coding
9562	MINPP1	multiple inositol-polyphosphate phosphatase 1	protein-coding
9575	CLOCK	clock circadian regulator	protein-coding
9582	APOBEC3B	apolipoprotein B mRNA editing enzyme, catalytic polypeptide-like 3B	protein-coding
9622	KLK4	kallikrein-related peptidase 4	protein-coding
9623	TCL1B	T-cell leukemia/lymphoma 1B	protein-coding
9734	HDAC9	histone deacetylase 9	protein-coding
9821	RB1CC1	RB1-inducible coiled-coil 1	protein-coding
9940	DLEC1	deleted in lung and esophageal cancer 1	protein-coding
9947	MAGEC1	melanoma antigen family C1	protein-coding
9950	GOLGA5	golgin A5	protein-coding
10015	PDCD6IP	programmed cell death 6 interacting protein	protein-coding
10016	PDCD6	programmed cell death 6	protein-coding
10111	RAD50	RAD50 homolog (S. cerevisiae)	protein-coding
10165	SLC25A13	solute carrier family 25 (aspartate/glutamate carrier), member 13	protein-coding
10320	IKZF1	IKAROS family zinc finger 1 (Ikaros)	protein-coding
10395	DLC1	DLC1 Rho GTPase activating protein	protein-coding
10481	HOXB13	homeobox B13	protein-coding
10550	ARL6IP5	ADP-ribosylation factor-like 6 interacting protein 5	protein-coding
10553	HTATIP2	HIV-1 Tat interactive protein 2, 30kDa	protein-coding
10587	TXNRD2	thioredoxin reductase 2	protein-coding
10666	CD226	CD226 molecule	protein-coding
10801	SEPT9	septin 9	protein-coding
10804	GJB6	gap junction protein, beta 6, 30kDa	protein-coding
10891	PPARGC1A	peroxisome proliferator-activated receptor gamma, coactivator 1 alpha	protein-coding
10933	MORF4L1	mortality factor 4 like 1	protein-coding
10962	MLLT11	myeloid/lymphoid or mixed-lineage leukemia (trithorax homolog, Drosophila); translocated to, 11	protein-coding
11073	TOPBP1	topoisomerase (DNA) II binding protein 1	protein-coding
11168	PSIP1	PC4 and SFRS1 interacting protein 1	protein-coding
11178	LZTS1	leucine zipper, putative tumor suppressor 1	protein-coding
11186	RASSF1	Ras association (RalGDS/AF-6) domain family member 1	protein-coding
11200	CHEK2	checkpoint kinase 2	protein-coding
11201	POLI	polymerase (DNA directed) iota	protein-coding
11214	AKAP13	A kinase (PRKA) anchor protein 13	protein-coding

11236	RNF139	ring finger protein 139	protein-coding
11281	POU6F2	POU class 6 homeobox 2	protein-coding
11322	TMC6	transmembrane channel-like 6	protein-coding
11330	CTRC	chymotrypsin C (caldecrin)	protein-coding
22800	RRAS2	related RAS viral (r-ras) oncogene homolog 2	protein-coding
22803	XRN2	5'-3' exoribonuclease 2	protein-coding
22909	FAN1	FANCD2/FANCI-associated nuclease 1	protein-coding
23022	PALLD	palladin, cytoskeletal associated protein	protein-coding
23076	RRP1B	ribosomal RNA processing 1B	protein-coding
23089	PEG10	paternally expressed 10	protein-coding
23092	ARHGAP26	Rho GTPase activating protein 26	protein-coding
23095	KIF1B	kinesin family member 1B	protein-coding
23301	EHBP1	EH domain binding protein 1	protein-coding
23305	ACSL6	acyl-CoA synthetase long-chain family member 6	protein-coding
23365	ARHGEF12	Rho guanine nucleotide exchange factor (GEF) 12	protein-coding
23373	CRTC1	CREB regulated transcription coactivator 1	protein-coding
23405	DICER1	dicer 1, ribonuclease type III	protein-coding
25788	RAD54B	RAD54 homolog B (S. cerevisiae)	protein-coding
25913	POT1	protection of telomeres 1	protein-coding
26137	ZBTB20	zinc finger and BTB domain containing 20	protein-coding
26166	RGS22	regulator of G-protein signaling 22	protein-coding
26267	FBXO10	F-box protein 10	protein-coding
26277	TINF2	TERF1 (TRF1)-interacting nuclear factor 2	protein-coding
26511	CHIC2	cysteine-rich hydrophobic domain 2	protein-coding
26575	RGS17	regulator of G-protein signaling 17	protein-coding
26585	GREM1	gremlin 1, DAN family BMP antagonist	protein-coding
26762	HAVCR1	hepatitis A virus cellular receptor 1	protein-coding
27030	MLH3	mutL homolog 3	protein-coding
27178	IL37	interleukin 37	protein-coding
27232	GNMT	glycine N-methyltransferase	protein-coding
27324	TOX3	TOX high mobility group box family member 3	protein-coding
29851	ICOS	inducible T-cell co-stimulator	protein-coding
30835	CD209	CD209 molecule	protein-coding
50514	DEC1	deleted in esophageal cancer 1	protein-coding
50805	IRX4	iroquois homeobox 4	protein-coding
50940	PDE11A	phosphodiesterase 11A	protein-coding
51008	ASCC1	activating signal cointegrator 1 complex subunit 1	protein-coding
51079	NDUFA13	NADH dehydrogenase (ubiquinone) 1 alpha subcomplex, 13	protein-coding
51119	SBDS	shwachman-bodian-diamond syndrome	protein-coding
51176	LEF1	lymphoid enhancer-binding factor 1	protein-coding
51196	PLCE1	phospholipase C, epsilon 1	protein-coding
51592	TRIM33	tripartite motif containing 33	protein-coding
51684	SUFU	suppressor of fused homolog (Drosophila)	protein-coding
51741	WWOX	WW domain containing oxidoreductase	protein-coding
51750	RTEL1	regulator of telomere elongation helicase 1	protein-coding
54106	TLR9	toll-like receptor 9	protein-coding
54575	UGT1A10	UDP glucuronosyltransferase 1 family, polypeptide A10	protein-coding

54577	UGT1A7	UDP glucuronosyltransferase 1 family, polypeptide A7	protein-coding
54583	EGLN1	egl-9 family hypoxia-inducible factor 1	protein-coding
54658	UGT1A1	UDP glucuronosyltransferase 1 family, polypeptide A1	protein-coding
54790	TET2	tet methylcytosine dioxygenase 2	protein-coding
54904	WHSC1L1	wolf-hirschhorn syndrome candidate 1-like 1	protein-coding
54949	SDHAF2	succinate dehydrogenase complex assembly factor 2	protein-coding
55135	WRAP53	WD repeat containing, antisense to TP53	protein-coding
55215	FANCI	fanconi anemia, complementation group I	protein-coding
55294	FBXW7	F-box and WD repeat domain containing 7, E3 ubiquitin protein ligase	protein-coding
55353	LAPTM4B	lysosomal protein transmembrane 4 beta	protein-coding
55505	NOP10	NOP10 ribonucleoprotein	protein-coding
55651	NHP2	NHP2 ribonucleoprotein	protein-coding
55654	TMEM127	transmembrane protein 127	protein-coding
55729	ATF7IP	activating transcription factor 7 interacting protein	protein-coding
55743	CHFR	checkpoint with forkhead and ring finger domains, E3 ubiquitin protein ligase	protein-coding
56244	BTNL2	butyrophilin-like 2	protein-coding
57492	ARID1B	AT rich interactive domain 1B (SWI1-like)	protein-coding
57522	SRGAP1	SLIT-ROBO Rho GTPase activating protein 1	protein-coding
57591	MKL1	megakaryoblastic leukemia (translocation) 1	protein-coding
57697	FANCM	fanconi anemia, complementation group M	protein-coding
59348	ZNF350	zinc finger protein 350	protein-coding
60528	ELAC2	elaC ribonuclease Z 2	protein-coding
60561	RINT1	RAD50 interactor 1	protein-coding
64127	NOD2	nucleotide-binding oligomerization domain containing 2	protein-coding
64241	ABCG8	ATP-binding cassette, sub-family G (WHITE), member 8	protein-coding
64318	NOC3L	nucleolar complex associated 3 homolog (S. cerevisiae)	protein-coding
64324	NSD1	nuclear receptor binding SET domain protein 1	protein-coding
64754	SMYD3	SET and MYND domain containing 3	protein-coding
64783	RBM15	RNA binding motif protein 15	protein-coding
64848	YTHDC2	YTH domain containing 2	protein-coding
79058	ASPSCR1	alveolar soft part sarcoma chromosome region, candidate 1	protein-coding
79577	CDC73	cell division cycle 73	protein-coding
79633	FAT4	FAT atypical cadherin 4	protein-coding
79648	MCPH1	microcephalin 1	protein-coding
79651	RHBDF2	rhomboid 5 homolog 2 (Drosophila)	protein-coding
79695	GALNT12	polypeptide N-acetylgalactosaminyltransferase 12	protein-coding
79723	SUV39H2	suppressor of variegation 3-9 homolog 2 (Drosophila)	protein-coding
79728	PALB2	partner and localizer of BRCA2	protein-coding
80169	CTC1	CTS telomere maintenance complex component 1	protein-coding
81037	CLPTM1L	CLPTM1-like	protein-coding
81704	DOCK8	dedicator of cytokinesis 8	protein-coding
81848	SPRY4	sprouty homolog 4 (Drosophila)	protein-coding
83550	GPR101	G protein-coupled receptor 101	protein-coding
83734	ATG10	autophagy related 10	protein-coding
83990	BRIP1	BRCA1 interacting protein C-terminal helicase 1	protein-coding
84142	FAM175A	family with sequence similarity 175, member A	protein-coding
84168	ANTXR1	anthrax toxin receptor 1	protein-coding

84295	PHF6	PHD finger protein 6	protein-coding
84441	MAML2	mastermind-like 2 (Drosophila)	protein-coding
84445	LZTS2	leucine zipper, putative tumor suppressor 2	protein-coding
84651	SPINK7	serine peptidase inhibitor, Kazal type 7 (putative)	protein-coding
84868	HAVCR2	hepatitis A virus cellular receptor 2	protein-coding
84925	DIRC2	disrupted in renal carcinoma 2	protein-coding
92335	STRADA	STE20-related kinase adaptor alpha	protein-coding
112398	EGLN2	egl-9 family hypoxia-inducible factor 2	protein-coding
114799	ESCO1	establishment of sister chromatid cohesion N-acetyltransferase 1	protein-coding
115761	ARL11	ADP-ribosylation factor-like 11	protein-coding
115908	CTHRC1	collagen triple helix repeat containing 1	protein-coding
116840	CNTROB	centrobin, centrosomal BRCA2 interacting protein	protein-coding
120376	COLCA2	colorectal cancer associated 2	protein-coding
129563	DIS3L2	DIS3 like 3’-5’ exoribonuclease 2	protein-coding
133522	PPARGC1B	peroxisome proliferator-activated receptor gamma, coactivator 1 beta	protein-coding
147138	TMC8	transmembrane channel-like 8	protein-coding
149233	IL23R	interleukin 23 receptor	protein-coding
153090	DAB2IP	DAB2 interacting protein	protein-coding
157570	ESCO2	establishment of sister chromatid cohesion N-acetyltransferase 2	protein-coding
162681	C18orf54	chromosome 18 open reading frame 54	protein-coding
166968	MIER3	mesoderm induction early response 1, family member 3	protein-coding
200315	APOBEC3A	apolipoprotein B mRNA editing enzyme, catalytic polypeptide-like 3A	protein-coding
200942	KLHDC8B	kelch domain containing 8B	protein-coding
201163	FLCN	folliculin	protein-coding
201294	UNC13D	unc-13 homolog D (C. elegans)	protein-coding
222546	RFX6	regulatory factor X, 6	protein-coding
246778	IL27	interleukin 27	protein-coding
282617	IFNL3	interferon, lambda 3	protein-coding
284654	RSPO1	R-spondin 1	protein-coding
342184	FMN1	formin 1	protein-coding
345611	IRGM	immunity-related GTPase family, M	protein-coding
389421	LIN28B	lin-28 homolog B (C. elegans)	protein-coding
399948	COLCA1	colorectal cancer associated 1	protein-coding
729230	CCR2	chemokine (C-C motif) receptor 2	protein-coding
100144748	KLLN	killin, p53-regulated DNA replication inhibitor	protein-coding
100507436	MICA	MHC class I polypeptide-related sequence A	protein-coding

Supplementarey Table S2: The information of 23 non-coding CPGs

Gene ID	Official symbol	Official full name	Gene type
6023	RMRP	RNA component of mitochondrial RNA processing endoribonuclease	ncRNA
7012	TERC	telomerase RNA component	ncRNA
10866	HCP5	HLA complex P5	ncRNA
10984	KCNQ1OT1	KCNQ1 opposite strand/antisense transcript 1	ncRNA
283120	H19	H19, imprinted maternally expressed transcript	ncRNA
406938	MIR146A	microRNA 146a	ncRNA
406941	MIR149	microRNA 149	ncRNA
406960	MIR184	microRNA 184	ncRNA
406972	MIR196A1	microRNA 196a-1	ncRNA
406973	MIR196A2	microRNA 196a-2	ncRNA
406985	MIR200C	microRNA 200c	ncRNA
406995	MIR181A1	microRNA 181a-1	ncRNA
407018	MIR27A	microRNA 27a	ncRNA
407041	MIR34B	microRNA 34b	ncRNA
407042	MIR34C	microRNA 34c	ncRNA
442893	MIR151A	microRNA 151a	ncRNA
494327	MIR378A	microRNA 378a	ncRNA
574501	MIR499A	microRNA 499a	ncRNA
693155	MIR570	microRNA 570	ncRNA
100126327	MIR938	microRNA 938	ncRNA
100126334	MIR885	microRNA 885	ncRNA
100886964	PTCSC3	papillary thyroid carcinoma susceptibility candidate 3	ncRNA
344967	LOC344967	acyl-CoA thioesterase 7 pseudogene	pseudo

Supplementarey Table S3: The significantly enriched pathway annotations in the 724 human protein-coding CPGs

Annotation source	Functional term	Raw *P*-value	Benjamini-Hochberg adjusted *P*-value
Reactome	DNA Repair	1.24E-15	1.76E-12
KEGG PATHWAY	Pathways in cancer	1.54E-11	6.26E-09
Reactome	Signaling by SCF-KIT	7.13E-09	9.48E-07
Reactome	Signaling by FGFR in disease	1.52E-08	1.76E-06
Reactome	Constitutive PI3K/AKT Signaling in Cancer	1.00E-07	7.72E-06
Reactome	Signaling by ERBB4	1.02E-07	7.79E-06
Reactome	Downstream signal transduction	1.54E-07	1.04E-05
KEGG PATHWAY	Bladder cancer	2.15E-07	1.35E-05
PANTHER	p53 pathway	2.28E-07	1.42E-05
KEGG PATHWAY	Hepatitis B	4.08E-07	2.36E-05
KEGG PATHWAY	Prostate cancer	5.85E-07	3.11E-05
Reactome	PI3K/AKT activation	6.70E-07	3.46E-05
Reactome	Double-Strand Break Repair	7.33E-07	3.74E-05
Reactome	Signaling by FGFR	8.35E-07	4.18E-05
Reactome	Downstream signaling of activated FGFR	1.10E-06	5.02E-05
Reactome	PI-3K cascade	1.12E-06	5.02E-05

Reactome	PIP3 activates AKT signaling	1.12E-06	5.02E-05
Reactome	PI3K events in ERBB2 signaling	1.12E-06	5.02E-05
Reactome	PI3K events in ERBB4 signaling	1.12E-06	5.02E-05
Reactome	PI3K/AKT Signaling in Cancer	1.12E-06	5.02E-05
Reactome	Signaling by PDGF	1.14E-06	5.08E-05
KEGG PATHWAY	Colorectal cancer	1.51E-06	6.21E-05
Reactome	Role of LAT2/NTAL/LAB on calcium mobilization	1.63E-06	6.64E-05
Reactome	GAB1 signalosome	1.92E-06	7.56E-05
Reactome	Signaling by ERBB2	3.41E-06	0.00012442
KEGG PATHWAY	Transcriptional misregulation in cancer	3.87E-06	0.000138792
Reactome	Fc epsilon receptor (FCERI) signaling	5.16E-06	0.000174694
KEGG PATHWAY	Chronic myeloid leukemia	7.87E-06	0.000253754
Reactome	DAP12 signaling	7.94E-06	0.000255182
KEGG PATHWAY	Inflammatory bowel disease (IBD)	1.22E-05	0.000368006
KEGG PATHWAY	p53 signaling pathway	1.51E-05	0.000440671
PANTHER	p53 pathway feedback loops 2	1.67E-05	0.000474942
Reactome	Signaling by Overexpressed Wild-Type EGFR in Cancer	2.02E-05	0.000547256
Reactome	Signaling by EGFR	2.02E-05	0.000547256
KEGG PATHWAY	Allograft rejection	2.24E-05	0.000589157
Reactome	Signaling by EGFR in Cancer	2.51E-05	0.000640583
Reactome	Signaling by EGFRvIII in Cancer	2.51E-05	0.000640583
Reactome	Signaling by Ligand-Responsive EGFR Variants in Cancer	2.51E-05	0.000640583
Reactome	Signaling by FGFR mutants	3.01E-05	0.000738616
Reactome	NGF signalling via TRKA from the plasma membrane	3.53E-05	0.000839106
KEGG PATHWAY	Endometrial cancer	3.55E-05	0.000840489
Reactome	DAP12 interactions	3.92E-05	0.000904633
Reactome	Constitutive Signaling by EGFRvIII	5.31E-05	0.001165444
KEGG PATHWAY	Thyroid cancer	7.57E-05	0.001563371
PID	BARD1 signaling events	7.86E-05	0.001607189
Reactome	Fanconi Anemia pathway	8.37E-05	0.001698084
Reactome	IRS-related events triggered by IGF1R	8.92E-05	0.001790419
KEGG PATHWAY	Chagas disease (American trypanosomiasis)	9.53E-05	0.001875289
Reactome	EGFR Transactivation by Gastrin	9.77E-05	0.001905959
KEGG PATHWAY	Type I diabetes mellitus	0.000106592	0.002062232
KEGG PATHWAY	Fanconi anemia pathway	0.000109046	0.002102563
KEGG PATHWAY	Pancreatic cancer	0.000124269	0.002341271
Reactome	Signaling by Type 1 Insulin-like Growth Factor 1 Receptor (IGF1R)	0.000137704	0.002551328
Reactome	IGF1R signaling cascade	0.000137704	0.002551328
PANTHER	Interleukin signaling pathway	0.000154737	0.002776476
Reactome	Homologous Recombination Repair	0.000161175	0.002855953
Reactome	Homologous recombination repair of replication-independent double-strand breaks	0.000161175	0.002855953
Reactome	Downstream signaling events of B Cell Receptor (BCR)	0.000161653	0.002859968
Reactome	Constitutive Signaling by Ligand-Responsive EGFR Cancer Variants	0.000223935	0.003774059
KEGG PATHWAY	Proteoglycans in cancer	0.000236011	0.003930987
KEGG PATHWAY	Acute myeloid leukemia	0.000250097	0.004117372

KEGG PATHWAY	Renal cell carcinoma	0.000275339	0.004461911
Reactome	GRB2 events in EGFR signaling	0.000305701	0.004863856
Reactome	IRS-mediated signalling	0.000322473	0.005052959
Reactome	Global Genomic NER (GG-NER)	0.000362073	0.005513972
Reactome	SHC1 events in EGFR signaling	0.000410649	0.006162979
KEGG PATHWAY	Graft-versus-host disease	0.000412212	0.006178295
KEGG PATHWAY	FoxO signaling pathway	0.000427244	0.006302994
Reactome	Signaling by the B Cell Receptor (BCR)	0.000442629	0.006390037
Reactome	IRS-related events	0.000483801	0.006871064
KEGG PATHWAY	Glioma	0.000499189	0.007036901
Reactome	Mismatch Repair	0.000501064	0.007045858
Reactome	Dual incision reaction in GG-NER	0.000543517	0.0075037
Reactome	Formation of incision complex in GG-NER	0.000543517	0.0075037
Reactome	Early Phase of HIV Life Cycle	0.000567478	0.007796649
Reactome	Death Receptor Signalling	0.000567478	0.007796649
KEGG PATHWAY	Melanoma	0.000650579	0.008625855
Reactome	Extrinsic Pathway	0.000679601	0.008937729
Reactome	GRB2 events in ERBB2 signaling	0.000679601	0.008937729
KEGG PATHWAY	African trypanosomiasis	0.000798826	0.010119649
Reactome	Insulin receptor signalling cascade	0.000804353	0.010178325
Reactome	SHC1 events in ERBB2 signaling	0.000854048	0.010729532
KEGG PATHWAY	HIF-1 signaling pathway	0.000908724	0.011187922
Reactome	SHC-related events triggered by IGF1R	0.000914881	0.011239404
Reactome	Signaling by Leptin	0.000914881	0.011239404
KEGG PATHWAY	Non-small cell lung cancer	0.001028703	0.012396739
Reactome	Costimulation by the CD28 family	0.001084984	0.01300857
PANTHER	Hypoxia response via HIF activation	0.001116057	0.013280614
Reactome	Nonhomologous End-joining (NHEJ)	0.001203827	0.014077172
Reactome	Resolution of Abasic Sites (AP sites)	0.001206509	0.014077172
Reactome	SOS-mediated signalling	0.001206509	0.014077172
Reactome	Base Excision Repair	0.001206509	0.014077172
KEGG PATHWAY	Homologous recombination	0.00122145	0.014222326
KEGG PATHWAY	HTLV-I infection	0.001355076	0.015508392
KEGG PATHWAY	Malaria	0.001391624	0.015831117
KEGG PATHWAY	Central carbon metabolism in cancer	0.001454407	0.016430326
Reactome	Mismatch repair (MMR) directed by MSH2:MSH6 (MutSalpha)	0.001537274	0.017128331
Reactome	Mismatch repair (MMR) directed by MSH2:MSH3 (MutSbeta)	0.001537274	0.017128331
Reactome	Biological oxidations	0.001558635	0.017305898
KEGG PATHWAY	Autoimmune thyroid disease	0.001563956	0.017336336
KEGG PATHWAY	Rheumatoid arthritis	0.001580672	0.01744256
Reactome	Activation of Matrix Metalloproteinases	0.001608852	0.017649275
Reactome	Signalling by NGF	0.001736788	0.018590035
Reactome	Innate Immune System	0.001908869	0.019904032
Reactome	2-LTR circle formation	0.001922459	0.019946594
Reactome	Signalling to p38 via RIT and RIN	0.002007282	0.020505144
Reactome	Nucleotide Excision Repair	0.002111978	0.021299522

KEGG PATHWAY	Measles	0.002298728	0.022918785
KEGG PATHWAY	Chemical carcinogenesis	0.002476022	0.024282096
Reactome	ARMS-mediated activation	0.002539561	0.024755821
Reactome	Resolution of AP sites via the single-nucleotide replacement pathway	0.002565231	0.024963277
Reactome	Removal of DNA patch containing abasic residue	0.002690932	0.02585477
Reactome	Resolution of AP sites via the multiple-nucleotide patch replacement pathway	0.002690932	0.02585477
Reactome	VEGF ligand-receptor interactions	0.002910683	0.027373881
Reactome	Recruitment of repair and signaling proteins to double-strand breaks	0.002910683	0.027373881
Reactome	VEGF binds to VEGFR leading to receptor dimerization	0.002910683	0.027373881
PANTHER	PI3 kinase pathway	0.002930347	0.027459335
KEGG PATHWAY	Non-homologous end-joining	0.002958352	0.027698945
BioCyc	melatonin degradation I	0.003038648	0.02826464
BioCarta	p53 signaling pathway	0.003404389	0.031232522
PID	IL27-mediated signaling events	0.003473013	0.031708664
Reactome	AKT phosphorylates targets in the cytosol	0.003487271	0.031813311
Reactome	Frs2-mediated activation	0.003929786	0.035075961
PID	Regulation of Telomerase	0.003931246	0.035075961
PANTHER	Insulin/IGF pathway-protein kinase B signaling cascade	0.003938056	0.035109187
BioCyc	TCA cycle	0.004046146	0.035876041
BioCyc	superpathway of melatonin degradation	0.004075039	0.036104081
KEGG PATHWAY	Viral myocarditis	0.004114146	0.036309177
Reactome	Integration of provirus	0.004219303	0.037064705
Reactome	Highly calcium permeable nicotinic acetylcholine receptors	0.004219303	0.037064705
Reactome	Regulation of gene expression by Hypoxia-inducible Factor	0.004219303	0.037064705
Reactome	Cellular responses to stress	0.004300243	0.037368964
Reactome	SMAC-mediated dissociation of IAP:caspase complexes	0.004300398	0.037368964
Reactome	SMAC binds to IAPs	0.004300398	0.037368964
Reactome	FasL/ CD95L signaling	0.004300398	0.037368964
Reactome	SMAC-mediated apoptotic response	0.004300398	0.037368964
PANTHER	Apoptosis signaling pathway	0.004303212	0.037368964
BioCarta	cell cycle: g1/s check point	0.004509353	0.038512313
Reactome	Defective CYP27A1 causes Cerebrotendinous xanthomatosis (CTX)	0.004620934	0.0386812
Reactome	Defective CYP11B1 causes Adrenal hyperplasia 4 (AH4)	0.004620934	0.0386812
Reactome	Defective CYP17A1 causes Adrenal hyperplasia 5 (AH5)	0.004620934	0.0386812
Reactome	Defective FMO3 causes Trimethylaminuria (TMAU)	0.004620934	0.0386812
Reactome	Defective MAOA causes Brunner syndrome (BRUNS)	0.004620934	0.0386812
Reactome	Defective TBXAS1 causes Ghosal hematodiaphyseal dysplasia (GHDD)	0.004620934	0.0386812
Reactome	Defective CYP21A2 causes Adrenal hyperplasia 3 (AH3)	0.004620934	0.0386812
Reactome	Defective CYP11A1 causes Adrenal insufficiency, congenital, with 46,XY sex reversal (AICSR)	0.004620934	0.0386812
Reactome	Defective CYP7B1 causes Spastic paraplegia 5A, autosomal recessive (SPG5A) and Congenital bile acid synthesis defect 3 (CBAS3)	0.004620934	0.0386812

Reactome	Defective CYP27B1 causes Rickets vitamin D-dependent 1A (VDDR1A)	0.004620934	0.0386812
Reactome	Defective CYP11B2 causes Corticosterone methyloxidase 1 deficiency (CMO-1 deficiency)	0.004620934	0.0386812
Reactome	Defective CYP4F22 causes Ichthyosis, congenital, autosomal recessive 5 (ARCI5)	0.004620934	0.0386812
Reactome	Defective CYP2U1 causes Spastic paraplegia 56, autosomal recessive (SPG56)	0.004620934	0.0386812
Reactome	Defective CYP26C1 causes Focal facial dermal dysplasia 4 (FFDD4)	0.004620934	0.0386812
Reactome	Defective CYP26B1 causes Radiohumeral fusions with other skeletal and craniofacial anomalies (RHFCA)	0.004620934	0.0386812
Reactome	Defective CYP2R1 causes Rickets vitamin D-dependent 1B (VDDR1B)	0.004620934	0.0386812
Reactome	Phase 1 - Functionalization of compounds	0.004620934	0.0386812
Reactome	Defective CYP1B1 causes Glaucoma	0.004620934	0.0386812
Reactome	Defective CYP19A1 causes Aromatase excess syndrome (AEXS)	0.004620934	0.0386812
Reactome	Defective CYP24A1 causes Hypercalcemia, infantile (HCAI)	0.004620934	0.0386812
Reactome	Regulation of Hypoxia-inducible Factor (HIF) by oxygen	0.004814267	0.039888642
Reactome	Cellular response to hypoxia	0.004814267	0.039888642
Reactome	VEGFR2 mediated cell proliferation	0.004863953	0.040124967
BioCarta	atm signaling pathway	0.005384145	0.04359433
Reactome	SHC-mediated signalling	0.005535902	0.044632474
Reactome	Prolonged ERK activation events	0.005843452	0.046551201
Reactome	Interleukin-2 signaling	0.005886436	0.046860806
Reactome	Interleukin-6 signaling	0.005898875	0.0468942
Reactome	Base-free sugar-phosphate removal via the single-nucleotide replacement pathway	0.005898875	0.0468942
Reactome	Signaling by Interleukins	0.006028772	0.047685042
BioCyc	superpathway of steroid hormone biosynthesis	0.006188975	0.048290641
BioCarta	role of brca1 brca2 and atr in cancer susceptibility	0.006231484	0.048352547
KEGG PATHWAY	Intestinal immune network for IgA production	0.00637655	0.04921278
PID	AP-1 transcription factor network	0.006398248	0.049346757

Supplementary Table S4: The significantly enriched disease annotations in the 724 human protein-coding CPGs

Annotation source	Functional term	Raw *P*-value	Benjamini-Hochberg adjusted *P*-value
GAD	Cancer	4.98E-30	5.67E-26
GAD	Breast cancer	3.77E-29	2.15E-25
GAD	Colorectal cancer	1.35E-27	5.13E-24
GAD	Lung cancer	5.18E-23	1.47E-19
GAD	Prostate cancer	4.04E-20	9.19E-17
GAD	Stomach cancer	3.54E-17	6.70E-14
GAD	Bladder cancer	8.49E-13	8.05E-10
GAD	Esophageal cancer	4.68E-12	2.32E-09
GAD	Ovarian cancer	2.72E-09	4.76E-07
GAD	Endometrial cancer	2.77E-08	2.76E-06
GAD	Endometriosis	3.59E-08	3.44E-06
GAD	Head and neck cancer	3.84E-08	3.55E-06
GAD	Oral cancer	1.11E-07	8.32E-06
GAD	Diabetes, type 1	1.24E-07	8.94E-06
GAD	Melanoma	1.28E-07	9.08E-06
GAD	Stomach neoplasms	4.64E-07	2.59E-05
GAD	Sarcoidosis	6.33E-07	3.32E-05
GAD	Infection	9.24E-07	4.45E-05
GAD	Neoplasms	9.81E-07	4.62E-05
GAD	Leukemia	1.09E-06	5.02E-05
GAD	Leukemia, myeloid	1.27E-06	5.50E-05
GAD	Overall effect	1.65E-06	6.67E-05
GAD	Rheumatoid arthritis	1.67E-06	6.70E-05
GAD	Crohn's disease	1.88E-06	7.44E-05
GAD	Diabetes, type 2	2.36E-06	9.05E-05
GAD	Cervical cancer	2.46E-06	9.35E-05
GAD	Hepatitis b	3.90E-06	0.000139505
GAD	Reproduction	1.37E-05	0.000407534
GAD	Liver cancer	1.42E-05	0.000416113
GAD	Tuberculosis	1.77E-05	0.000497023
GAD	Gastric cancer	1.79E-05	0.000502555
GAD	Ulcerative colitis	1.96E-05	0.000540602
GAD	Inflammatory bowel disease	2.15E-05	0.000567824
GAD	HIV	2.57E-05	0.000651103
GAD	Lupus	3.17E-05	0.000766429
GAD	Periodontitis	6.51E-05	0.00138004
GAD	Pregnancy loss, recurrent	7.33E-05	0.00152106
GAD	Atopy	7.93E-05	0.001618829
GAD	Preterm delivery	0.000116346	0.002231965
GAD	Cardiovascular disease	0.00011949	0.002284564
GAD	Kidney cancer	0.000121036	0.002298666
GAD	Nasopharyngeal cancer	0.000121036	0.002298666
FunDO	Oral cancer	0.000124308	0.002341271
GAD	Chronic obstructive pulmonary disease/ copd	0.000127977	0.002398462

NHGRI GWAS Catalog	Bladder cancer	0.000143826	0.002634728
GAD	Brain cancer	0.000180657	0.003128093
GAD	Liver disease	0.000190623	0.003270783
GAD	Sclerosis, systemic	0.000239919	0.0039844
GAD	Polycystic ovary syndrome	0.00026605	0.004342308
GAD	Pancreatic cancer	0.000290942	0.00467479
GAD	Cytogenetic studies	0.000292745	0.004690524
GAD	Benzene toxicity	0.000292745	0.004690524
GAD	Myocardial infarct	0.000337897	0.005251259
GAD	Kidney failure, chronic	0.000339858	0.005262275
NHGRI GWAS Catalog	Melanoma	0.000342205	0.005267826
NHGRI GWAS Catalog	Testicular germ cell cancer	0.000399802	0.006032027
GAD	Kidney transplant	0.000431789	0.006302994
GAD	Cirrhosis, biliary primary	0.000442028	0.006390037
GAD	Carcinoma, squamous cell	0.000442028	0.006390037
GAD	Hepatitis b, chronic	0.000456673	0.006538124
GAD	Leukemia, myeloid, acute	0.000456673	0.006538124
FunDO	Behcet syndrome	0.000572653	0.007829928
NHGRI GWAS Catalog	Chronic lymphocytic leukemia	0.000685618	0.009006456
GAD	Allergic rhinitis	0.000689037	0.009040928
GAD	Atherosclerosis, coronary	0.000707474	0.009250833
FunDO	Endometriosis	0.000741124	0.009580716
GAD	Hepatitis c	0.000750071	0.009609013
GAD	Preeclampsia	0.000750071	0.009609013
GAD	Parkinson's disease	0.000861039	0.010763933
GAD	Lung neoplasms	0.000884074	0.010979508
GAD	Tobacco use disorder	0.001017293	0.012324523
GAD	Prostatic hyperplasia	0.001087702	0.013024947
GAD	Behcet's disease	0.001095472	0.013090434
GAD	Pancreatitis	0.001095472	0.013090434
NHGRI GWAS Catalog	Glioma	0.001099977	0.013130474
GAD	Polycystic ovarian syndrome	0.001124934	0.013302751
GAD	Bone mineral density	0.001124934	0.013302751
GAD	Sepsis	0.001124934	0.013302751
NHGRI GWAS Catalog	Lung cancer	0.001135106	0.013409107
GAD	Thrombophilia	0.001440548	0.016289939
NHGRI GWAS Catalog	Basal cell carcinoma	0.001580317	0.01744256
GAD	Radiotherapy response	0.001618989	0.017709254
GAD	Adenocarcinoma	0.001618989	0.017709254
GAD	Sjogren's syndrome	0.001630624	0.017802279
GAD	Graves' disease	0.001666338	0.018026647
GAD	Nephropathy	0.001666338	0.018026647
NHGRI GWAS Catalog	Lung adenocarcinoma	0.001687228	0.018227831
GAD	Rhinitis	0.001729763	0.01856193
GAD	Thyroid cancer	0.001729763	0.01856193
GAD	Kawasaki disease	0.001729763	0.01856193
GAD	Puberty, delayed	0.001787786	0.018936553

GAD	Puberty, precocious	0.001787786	0.018936553
GAD	Pof - premature ovarian failure	0.001787786	0.018936553
GAD	Primary ovarian insufficiency	0.001787786	0.018936553
GAD	Systemic lupus erythematosus	0.001815174	0.019137556
FunDO	Periodontitis	0.001831945	0.01927864
GAD	Infertility, male	0.002007976	0.020505144
GAD	Natural menopause	0.002007976	0.020505144
GAD	Coronary heart disease	0.002321607	0.023105516
GAD	Nephropathy in other diseases	0.00232355	0.023105516
GAD	Retinopathy, diabetic	0.002353055	0.023358074
GAD	Alzheimer's disease	0.002505331	0.024464723
GAD	Pneumonia	0.002695759	0.025879282
GAD	Pharmacogenomic	0.003066392	0.028476141
GAD	Esophageal neoplasms	0.003303922	0.030458201
NHGRI GWAS Catalog	Upper aerodigestive tract cancers	0.003398239	0.031201265
GAD	Graft-versus-host disease	0.003853562	0.034436857
GAD	Lung function	0.003853562	0.034436857
NHGRI GWAS Catalog	Inflammatory bowel disease	0.003954685	0.035202264
GAD	Urinary bladder cancer	0.004088871	0.036170289
GAD	Upper aerodigestive tract cancer	0.004088871	0.036170289
GAD	Cholangitis, sclerosing	0.004231076	0.037082219
GAD	Giant cell arteritis	0.004231076	0.037082219
GAD	Limb deficiency anomalies	0.004231076	0.037082219
GAD	Crohn's disease ulcerative colitis	0.004527643	0.038639511
GAD	Lupus erythematosus	0.00466408	0.03892779
GAD	Hepatitis c, chronic	0.004732306	0.039468266
GAD	Multiple myeloma	0.005462447	0.044102769
GAD	Lymphoma	0.005462447	0.044102769
GAD	Acute coronary syndrome	0.005462447	0.044102769
GAD	Hepatocellular carcinoma	0.006326658	0.048894067
GAD	Urinary bladder neoplasms	0.006326658	0.048894067
GAD	Primary biliary cirrhosis	0.006326658	0.048894067
FunDO	Pancreatitis	0.006439424	0.049563525

Supplementary Table S5: The significantly enriched Gene Ontology (GO) annotations in the 724 human protein-coding CPGs

Annotation source	Functional term	Proportion of annotated genes to all 724 coding CPGs	Raw *P*-value	Benjamini-Hochberg adjusted *P*-value
GOTERM_BP_ALL	GO:0048518~positive regulation of biological process	40.31117397	5.66E-75	2.36E-71
GOTERM_BP_ALL	GO:0048522~positive regulation of cellular process	38.18953324	3.28E-74	6.83E-71
GOTERM_BP_ALL	GO:0048519~negative regulation of biological process	34.37057992	9.35E-58	9.74E-55
GOTERM_BP_ALL	GO:0042127~regulation of cell proliferation	22.20650636	8.15E-58	1.13E-54
GOTERM_BP_ALL	GO:0048523~negative regulation of cellular process	32.53182461	1.05E-56	8.77E-54
GOTERM_BP_ALL	GO:0010941~regulation of cell death	22.06506365	7.02E-55	4.88E-52
GOTERM_BP_ALL	GO:0042221~response to chemical stimulus	27.86421499	1.20E-54	7.15E-52
GOTERM_BP_ALL	GO:0043067~regulation of programmed cell death	21.92362093	2.48E-54	1.29E-51
GOTERM_BP_ALL	GO:0042981~regulation of apoptosis	21.78217822	3.71E-54	1.72E-51
GOTERM_BP_ALL	GO:0006950~response to stress	31.82461103	2.44E-52	1.02E-49
GOTERM_BP_ALL	GO:0050896~response to stimulus	47.94908062	3.40E-50	1.29E-47
GOTERM_BP_ALL	GO:0009893~positive regulation of metabolic process	22.4893918	9.51E-50	3.30E-47
GOTERM_BP_ALL	GO:0048513~organ development	31.68316832	2.45E-49	7.86E-47
GOTERM_BP_ALL	GO:0048731~system development	37.19943423	3.63E-48	1.08E-45
GOTERM_BP_ALL	GO:0051716~cellular response to stimulus	20.79207921	9.17E-48	2.55E-45
GOTERM_BP_ALL	GO:0051239~regulation of multicellular organismal process	22.06506365	1.27E-46	3.32E-44
GOTERM_BP_ALL	GO:0048856~anatomical structure development	38.47241867	4.39E-46	1.08E-43
GOTERM_BP_ALL	GO:0010604~positive regulation of macromolecule metabolic process	20.93352192	4.89E-46	1.13E-43
GOTERM_BP_ALL	GO:0031325~positive regulation of cellular metabolic process	21.21640736	5.57E-46	1.22E-43
GOTERM_BP_ALL	GO:0032502~developmental process	43.56435644	6.92E-45	1.44E-42
GOTERM_BP_ALL	GO:0050793~regulation of developmental process	17.82178218	1.66E-42	3.29E-40
GOTERM_BP_ALL	GO:0009891~positive regulation of biosynthetic process	17.96322489	9.14E-42	1.73E-39
GOTERM_BP_ALL	GO:0031328~positive regulation of cellular biosynthetic process	17.82178218	9.88E-42	1.79E-39
GOTERM_BP_ALL	GO:0010033~response to organic substance	18.24611033	1.94E-41	3.38E-39
GOTERM_BP_ALL	GO:0045595~regulation of cell differentiation	14.85148515	1.66E-40	2.76E-38
GOTERM_BP_ALL	GO:0006974~response to DNA damage stimulus	12.87128713	7.06E-40	1.13E-37
GOTERM_BP_ALL	GO:0007275~multicellular organismal development	39.6039604	1.99E-39	3.07E-37

GOTERM_BP_ALL	GO:0033554~cellular response to stress	15.55869873	1.73E-38	2.58E-36
GOTERM_BP_ALL	GO:0010557~positive regulation of macromolecule biosynthetic process	16.69024045	4.35E-38	6.25E-36
GOTERM_BP_ALL	GO:0009628~response to abiotic stimulus	12.44695898	8.41E-38	1.17E-35
GOTERM_BP_ALL	GO:0002376~immune system process	20.79207921	2.85E-37	3.83E-35
GOTERM_BP_ALL	GO:0051173~positive regulation of nitrogen compound metabolic process	16.26591231	1.25E-36	1.63E-34
GOTERM_BP_ALL	GO:0065009~regulation of molecular function	20.22630835	2.84E-36	3.58E-34
GOTERM_BP_ALL	GO:0065008~regulation of biological quality	25.60113154	6.55E-36	8.03E-34
GOTERM_BP_ALL	GO:0006281~DNA repair	10.60820368	3.20E-35	3.81E-33
GOTERM_BP_ALL	GO:0008285~negative regulation of cell proliferation	11.88118812	4.15E-35	4.81E-33
GOTERM_BP_ALL	GO:0009314~response to radiation	8.910891089	2.28E-34	2.57E-32
GOTERM_BP_ALL	GO:0030154~cell differentiation	26.87411598	2.61E-34	2.87E-32
GOTERM_BP_ALL	GO:0043069~negative regulation of programmed cell death	11.59830269	1.22E-33	1.30E-31
GOTERM_BP_ALL	GO:0060548~negative regulation of cell death	11.59830269	1.50E-33	1.57E-31
GOTERM_BP_ALL	GO:0010942~positive regulation of cell death	12.72984441	1.85E-33	1.88E-31
GOTERM_BP_ALL	GO:0043066~negative regulation of apoptosis	11.45685997	2.79E-33	2.77E-31
GOTERM_BP_ALL	GO:0043065~positive regulation of apoptosis	12.5884017	4.31E-33	4.08E-31
GOTERM_BP_ALL	GO:0019222~regulation of metabolic process	43.84724187	4.23E-33	4.10E-31
GOTERM_BP_ALL	GO:0045935~positive regulation of nucleobase, nucleoside, nucleotide and nucleic acid metabolic process	15.2758133	4.58E-33	4.25E-31
GOTERM_BP_ALL	GO:0065007~biological regulation	70.43847242	6.42E-33	5.82E-31
GOTERM_BP_ALL	GO:0048869~cellular developmental process	27.15700141	7.03E-33	6.24E-31
GOTERM_BP_ALL	GO:0043068~positive regulation of programmed cell death	12.5884017	7.50E-33	6.51E-31
GOTERM_BP_ALL	GO:0002520~immune system development	10.04243281	1.55E-32	1.32E-30
GOTERM_BP_ALL	GO:0032879~regulation of localization	14.85148515	6.65E-32	5.54E-30
GOTERM_BP_ALL	GO:0006259~DNA metabolic process	13.43705799	7.90E-32	6.46E-30
GOTERM_BP_ALL	GO:0050789~regulation of biological process	67.75106082	8.94E-32	7.17E-30
GOTERM_BP_ALL	GO:0031323~regulation of cellular metabolic process	42.14992928	9.54E-32	7.50E-30
GOTERM_BP_ALL	GO:0050794~regulation of cellular process	65.91230552	1.17E-31	9.02E-30
GOTERM_BP_ALL	GO:0044093~positive regulation of molecular function	14.427157	2.23E-31	1.69E-29
GOTERM_BP_ALL	GO:0002682~regulation of immune system process	11.59830269	2.33E-31	1.73E-29

GOTERM_BP_ALL	GO:0010628~positive regulation of gene expression	14.14427157	2.47E-30	1.80E-28
GOTERM_BP_ALL	GO:0008284~positive regulation of cell proliferation	11.7397454	8.47E-30	6.09E-28
GOTERM_BP_ALL	GO:0019220~regulation of phosphate metabolic process	12.72984441	1.01E-29	7.13E-28
GOTERM_BP_ALL	GO:0051174~regulation of phosphorus metabolic process	12.72984441	1.01E-29	7.13E-28
GOTERM_BP_ALL	GO:0048583~regulation of response to stimulus	12.30551627	5.57E-29	3.87E-27
GOTERM_BP_ALL	GO:0042325~regulation of phosphorylation	12.30551627	6.55E-29	4.47E-27
GOTERM_BP_ALL	GO:0051726~regulation of cell cycle	10.32531825	6.98E-29	4.70E-27
GOTERM_BP_ALL	GO:0009411~response to UV	4.809052334	8.21E-29	5.44E-27
GOTERM_BP_ALL	GO:0045941~positive regulation of transcription	13.57850071	1.06E-28	6.88E-27
GOTERM_BP_ALL	GO:0009719~response to endogenous stimulus	11.31541726	2.97E-28	1.90E-26
GOTERM_BP_ALL	GO:0009725~response to hormone stimulus	10.74964639	3.32E-28	2.10E-26
GOTERM_BP_ALL	GO:0048534~hemopoietic or lymphoid organ development	9.052333805	3.87E-28	2.41E-26
GOTERM_BP_ALL	GO:0060255~regulation of macromolecule metabolic process	39.17963225	6.73E-28	4.12E-26
GOTERM_BP_ALL	GO:0051254~positive regulation of RNA metabolic process	12.30551627	6.90E-28	4.17E-26
GOTERM_BP_ALL	GO:0010646~regulation of cell communication	18.9533239	7.51E-28	4.47E-26
GOTERM_BP_ALL	GO:0048545~response to steroid hormone stimulus	7.779349364	1.09E-27	6.41E-26
GOTERM_BP_ALL	GO:0009605~response to external stimulus	17.53889675	1.16E-27	6.74E-26
GOTERM_BP_ALL	GO:0045893~positive regulation of transcription, DNA-dependent	12.16407355	1.84E-27	1.05E-25
GOTERM_BP_ALL	GO:0032501~multicellular organismal process	46.8175389	3.41E-27	1.92E-25
GOTERM_BP_ALL	GO:0030097~hemopoiesis	8.486562942	3.54E-27	1.97E-25
GOTERM_BP_ALL	GO:0080090~regulation of primary metabolic process	38.89674682	2.12E-26	1.16E-24
GOTERM_BP_ALL	GO:0050790~regulation of catalytic activity	16.40735502	3.33E-26	1.81E-24
GOTERM_BP_ALL	GO:0042592~homeostatic process	15.2758133	4.83E-26	2.58E-24
GOTERM_BP_ALL	GO:0009966~regulation of signal transduction	16.54879774	2.39E-25	1.26E-23
GOTERM_BP_ALL	GO:0051093~negative regulation of developmental process	8.486562942	6.61E-25	3.45E-23
GOTERM_BP_ALL	GO:0051094~positive regulation of developmental process	8.769448373	8.51E-25	4.38E-23
GOTERM_BP_ALL	GO:0010647~positive regulation of cell communication	9.476661952	2.01E-24	1.02E-22
GOTERM_BP_ALL	GO:0009967~positive regulation of signal transduction	8.910891089	3.99E-24	2.01E-22

GOTERM_BP_ALL	GO:0009653~anatomical structure morphogenesis	19.51909477	7.26E-24	3.60E-22
GOTERM_BP_ALL	GO:0014070~response to organic cyclic substance	5.799151344	1.03E-23	5.03E-22
GOTERM_BP_ALL	GO:0009892~negative regulation of metabolic process	14.99292786	1.45E-23	7.02E-22
GOTERM_BP_ALL	GO:0045944~positive regulation of transcription from RNA polymerase II promoter	9.900990099	1.68E-23	7.95E-22
GOTERM_BP_ALL	GO:0043085~positive regulation of catalytic activity	11.88118812	1.67E-23	8.00E-22
GOTERM_BP_ALL	GO:0016043~cellular component organization	31.25884017	2.46E-23	1.15E-21
GOTERM_BP_ALL	GO:0007242~intracellular signaling cascade	19.94342291	2.70E-23	1.25E-21
GOTERM_BP_ALL	GO:0045596~negative regulation of cell differentiation	7.496463932	3.00E-23	1.38E-21
GOTERM_BP_ALL	GO:0051098~regulation of binding	6.364922207	3.43E-23	1.55E-21
GOTERM_BP_ALL	GO:0006357~regulation of transcription from RNA polymerase II promoter	14.28571429	3.72E-23	1.67E-21
GOTERM_BP_ALL	GO:0051704~multi-organism process	13.71994342	5.07E-23	2.25E-21
GOTERM_BP_ALL	GO:0031324~negative regulation of cellular metabolic process	14.14427157	6.50E-23	2.85E-21
GOTERM_BP_ALL	GO:0045597~positive regulation of cell differentiation	7.637906648	8.13E-23	3.53E-21
GOTERM_BP_ALL	GO:0051171~regulation of nitrogen compound metabolic process	33.80480905	1.46E-22	6.27E-21
GOTERM_BP_ALL	GO:0001775~cell activation	8.486562942	1.72E-22	7.33E-21
GOTERM_BP_ALL	GO:0040012~regulation of locomotion	6.930693069	2.67E-22	1.12E-20
GOTERM_BP_ALL	GO:0031399~regulation of protein modification process	8.486562942	7.36E-22	3.07E-20
GOTERM_BP_ALL	GO:0045321~leukocyte activation	7.637906648	1.24E-21	5.11E-20
GOTERM_BP_ALL	GO:0042493~response to drug	7.213578501	1.37E-21	5.59E-20
GOTERM_BP_ALL	GO:0051270~regulation of cell motion	6.789250354	2.41E-21	9.75E-20
GOTERM_BP_ALL	GO:0009416~response to light stimulus	5.799151344	2.48E-21	9.96E-20
GOTERM_BP_ALL	GO:0019219~regulation of nucleobase, nucleoside, nucleotide and nucleic acid metabolic process	33.09759547	2.87E-21	1.14E-19
GOTERM_BP_ALL	GO:0032268~regulation of cellular protein metabolic process	10.74964639	4.57E-21	1.80E-19
GOTERM_BP_ALL	GO:0051246~regulation of protein metabolic process	11.59830269	6.87E-21	2.68E-19
GOTERM_BP_ALL	GO:0046649~lymphocyte activation	6.789250354	9.47E-21	3.66E-19
GOTERM_BP_ALL	GO:0006917~induction of apoptosis	8.628005658	9.79E-21	3.74E-19
GOTERM_BP_ALL	GO:0051052~regulation of DNA metabolic process	5.233380481	1.04E-20	3.95E-19
GOTERM_BP_ALL	GO:0051338~regulation of transferase activity	9.335219236	1.11E-20	4.16E-19
GOTERM_BP_ALL	GO:0012502~induction of programmed cell death	8.628005658	1.15E-20	4.29E-19
GOTERM_BP_ALL	GO:0050865~regulation of cell activation	6.364922207	1.21E-20	4.45E-19

GOTERM_BP_ALL	GO:0010212~response to ionizing radiation	3.96039604	1.58E-20	5.77E-19
GOTERM_BP_ALL	GO:0007049~cell cycle	14.14427157	1.65E-20	5.97E-19
GOTERM_BP_ALL	GO:0006955~immune response	13.15417256	2.19E-20	7.86E-19
GOTERM_BP_ALL	GO:0010605~negative regulation of macromolecule metabolic process	13.57850071	3.95E-20	1.40E-18
GOTERM_BP_ALL	GO:0051049~regulation of transport	10.04243281	3.94E-20	1.40E-18
GOTERM_BP_ALL	GO:0070482~response to oxygen levels	5.657708628	4.99E-20	1.75E-18
GOTERM_BP_ALL	GO:0031326~regulation of cellular biosynthetic process	33.66336634	5.32E-20	1.85E-18
GOTERM_BP_ALL	GO:0001932~regulation of protein amino acid phosphorylation	6.223479491	5.38E-20	1.85E-18
GOTERM_BP_ALL	GO:0032583~regulation of gene-specific transcription	5.516265912	5.83E-20	1.99E-18
GOTERM_BP_ALL	GO:0000003~reproduction	13.86138614	7.81E-20	2.65E-18
GOTERM_BP_ALL	GO:0080134~regulation of response to stress	7.779349364	8.64E-20	2.91E-18
GOTERM_BP_ALL	GO:0045937~positive regulation of phosphate metabolic process	4.809052334	9.30E-20	3.10E-18
GOTERM_BP_ALL	GO:0010562~positive regulation of phosphorus metabolic process	4.809052334	9.30E-20	3.10E-18
GOTERM_BP_ALL	GO:0045859~regulation of protein kinase activity	8.769448373	9.85E-20	3.26E-18
GOTERM_BP_ALL	GO:0008219~cell death	13.29561528	1.07E-19	3.52E-18
GOTERM_BP_ALL	GO:0009889~regulation of biosynthetic process	33.66336634	1.24E-19	4.01E-18
GOTERM_BP_ALL	GO:0043549~regulation of kinase activity	8.910891089	1.24E-19	4.04E-18
GOTERM_BP_ALL	GO:0030334~regulation of cell migration	6.082036775	1.48E-19	4.74E-18
GOTERM_BP_ALL	GO:0022414~reproductive process	13.71994342	1.62E-19	5.15E-18
GOTERM_BP_ALL	GO:0012501~programmed cell death	12.02263083	1.69E-19	5.33E-18
GOTERM_BP_ALL	GO:0016265~death	13.29561528	1.72E-19	5.39E-18
GOTERM_BP_ALL	GO:0002521~leukocyte differentiation	5.374823197	2.10E-19	6.54E-18
GOTERM_BP_ALL	GO:0009888~tissue development	12.5884017	2.63E-19	8.11E-18
GOTERM_BP_ALL	GO:0051247~positive regulation of protein metabolic process	7.213578501	3.06E-19	9.37E-18
GOTERM_BP_ALL	GO:0042327~positive regulation of phosphorylation	4.667609618	3.41E-19	1.04E-17
GOTERM_BP_ALL	GO:0043627~response to estrogen stimulus	4.809052334	5.10E-19	1.54E-17
GOTERM_BP_ALL	GO:0048660~regulation of smooth muscle cell proliferation	3.394625177	5.53E-19	1.66E-17
GOTERM_BP_ALL	GO:0016477~cell migration	7.637906648	6.42E-19	1.91E-17
GOTERM_BP_ALL	GO:0035295~tube development	6.789250354	7.52E-19	2.22E-17
GOTERM_BP_ALL	GO:0051101~regulation of DNA binding	5.091937765	8.61E-19	2.53E-17
GOTERM_BP_ALL	GO:0048646~anatomical structure formation involved in morphogenesis	8.628005658	1.26E-18	3.68E-17
GOTERM_BP_ALL	GO:0010556~regulation of macromolecule biosynthetic process	32.10749646	1.58E-18	4.59E-17
GOTERM_BP_ALL	GO:0001817~regulation of cytokine production	6.082036775	2.28E-18	6.56E-17

GOTERM_BP_ALL	GO:0001568~blood vessel development	7.072135785	2.43E-18	6.94E-17
GOTERM_BP_ALL	GO:0006915~apoptosis	11.59830269	2.94E-18	8.33E-17
GOTERM_BP_ALL	GO:0009611~response to wounding	10.74964639	3.33E-18	9.37E-17
GOTERM_BP_ALL	GO:0002694~regulation of leukocyte activation	5.799151344	3.58E-18	1.00E-16
GOTERM_BP_ALL	GO:0001666~response to hypoxia	5.233380481	3.92E-18	1.09E-16
GOTERM_BP_ALL	GO:0007167~enzyme linked receptor protein signaling pathway	8.345120226	6.20E-18	1.71E-16
GOTERM_BP_ALL	GO:0001944~vasculature development	7.072135785	6.92E-18	1.90E-16
GOTERM_BP_ALL	GO:0032270~positive regulation of cellular protein metabolic process	6.789250354	8.52E-18	2.31E-16
GOTERM_BP_ALL	GO:0003006~reproductive developmental process	7.213578501	8.52E-18	2.32E-16
GOTERM_BP_ALL	GO:0006916~anti-apoptosis	6.364922207	1.01E-17	2.72E-16
GOTERM_BP_ALL	GO:0008361~regulation of cell size	6.364922207	1.01E-17	2.72E-16
GOTERM_BP_ALL	GO:0022402~cell cycle process	11.03253182	1.03E-17	2.73E-16
GOTERM_BP_ALL	GO:0031667~response to nutrient levels	6.223479491	1.02E-17	2.73E-16
GOTERM_BP_ALL	GO:0051128~regulation of cellular component organization	9.759547383	1.10E-17	2.90E-16
GOTERM_BP_ALL	GO:0009790~embryonic development	11.03253182	1.39E-17	3.65E-16
GOTERM_BP_ALL	GO:0043170~macromolecule metabolic process	52.75813296	3.00E-17	7.80E-16
GOTERM_BP_ALL	GO:0032101~regulation of response to external stimulus	5.516265912	3.36E-17	8.70E-16
GOTERM_BP_ALL	GO:0035239~tube morphogenesis	4.95049505	3.79E-17	9.75E-16
GOTERM_BP_ALL	GO:0000075~cell cycle checkpoint	4.243281471	4.66E-17	1.19E-15
GOTERM_BP_ALL	GO:0051172~negative regulation of nitrogen compound metabolic process	10.32531825	4.86E-17	1.24E-15
GOTERM_BP_ALL	GO:0048468~cell development	11.7397454	4.95E-17	1.25E-15
GOTERM_BP_ALL	GO:0051240~positive regulation of multicellular organismal process	6.789250354	5.75E-17	2.78E-15
GOTERM_BP_ALL	GO:0048870~cell motility	7.637906648	7.93E-17	2.78E-15
GOTERM_BP_ALL	GO:0051674~localization of cell	7.637906648	7.93E-17	2.78E-15
GOTERM_BP_ALL	GO:0007584~response to nutrient	5.091937765	1.34E-16	2.78E-15
GOTERM_BP_ALL	GO:0009991~response to extracellular stimulus	6.364922207	1.35E-16	2.78E-15
GOTERM_BP_ALL	GO:0006302~double-strand break repair	3.536067893	1.47E-16	2.78E-15
GOTERM_BP_ALL	GO:0001934~positive regulation of protein amino acid phosphorylation	4.101838755	2.37E-16	5.22E-15
GOTERM_BP_ALL	GO:0007568~aging	4.526166902	1.93E-16	5.33E-15
GOTERM_BP_ALL	GO:0010468~regulation of gene expression	31.5417256	2.06E-16	5.33E-15
GOTERM_BP_ALL	GO:0043687~post-translational protein modification	17.1145686	2.18E-16	5.33E-15
GOTERM_BP_ALL	GO:0006355~regulation of transcription, DNA-dependent	22.4893918	2.27E-16	5.33E-15
GOTERM_BP_ALL	GO:0051252~regulation of RNA metabolic process	22.91371994	1.86E-16	5.44E-15
GOTERM_BP_ALL	GO:0007346~regulation of mitotic cell cycle	5.233380481	3.13E-16	7.77E-15
GOTERM_BP_ALL	GO:0006928~cell motion	9.618104668	3.84E-16	7.77E-15

GOTERM_BP_ALL	GO:0008283~cell proliferation	9.193776521	2.97E-16	7.88E-15
GOTERM_BP_ALL	GO:0050678~regulation of epithelial cell proliferation	3.677510608	4.75E-16	1.02E-14
GOTERM_BP_ALL	GO:0008152~metabolic process	65.06364922	4.17E-16	1.03E-14
GOTERM_BP_ALL	GO:0006468~protein amino acid phosphorylation	11.7397454	6.09E-16	1.27E-14
GOTERM_BP_ALL	GO:0040008~regulation of growth	7.920792079	5.51E-16	1.28E-14
GOTERM_BP_ALL	GO:0060249~anatomical structure homeostasis	4.384724187	6.32E-16	1.51E-14
GOTERM_BP_ALL	GO:0008629~induction of apoptosis by intracellular signals	3.253182461	7.56E-16	1.75E-14
GOTERM_BP_ALL	GO:0007399~nervous system development	15.98302687	8.34E-16	1.99E-14
GOTERM_BP_ALL	GO:0032535~regulation of cellular component size	6.930693069	1.06E-15	2.48E-14
GOTERM_BP_ALL	GO:0007243~protein kinase cascade	8.203677511	1.36E-15	2.94E-14
GOTERM_BP_ALL	GO:0043193~positive regulation of gene-specific transcription	3.96039604	1.30E-15	2.95E-14
GOTERM_BP_ALL	GO:0045934~negative regulation of nucleobase, nucleoside, nucleotide and nucleic acid metabolic process	9.900990099	1.46E-15	3.16E-14
GOTERM_BP_ALL	GO:0022008~neurogenesis	10.89108911	1.55E-15	3.40E-14
GOTERM_BP_ALL	GO:0009987~cellular process	81.61244696	2.12E-15	4.55E-14
GOTERM_BP_ALL	GO:0030098~lymphocyte differentiation	4.243281471	2.09E-15	4.59E-14
GOTERM_BP_ALL	GO:0010332~response to gamma radiation	2.263083451	3.56E-15	7.64E-14
GOTERM_BP_ALL	GO:0006310~DNA recombination	4.243281471	3.70E-15	7.84E-14
GOTERM_BP_ALL	GO:0045637~regulation of myeloid cell differentiation	3.536067893	4.01E-15	8.50E-14
GOTERM_BP_ALL	GO:0040017~positive regulation of locomotion	4.101838755	4.22E-15	8.93E-14
GOTERM_BP_ALL	GO:0051272~positive regulation of cell motion	4.101838755	4.22E-15	8.93E-14
GOTERM_BP_ALL	GO:0031327~negative regulation of cellular biosynthetic process	10.32531825	4.38E-15	9.11E-14
GOTERM_BP_ALL	GO:0048514~blood vessel morphogenesis	5.940594059	5.23E-15	1.09E-13
GOTERM_BP_ALL	GO:0009887~organ morphogenesis	10.32531825	5.79E-15	1.20E-13
GOTERM_BP_ALL	GO:0051099~positive regulation of binding	3.677510608	6.54E-15	1.36E-13
GOTERM_BP_ALL	GO:0040011~locomotion	8.769448373	7.36E-15	1.51E-13
GOTERM_BP_ALL	GO:0051050~positive regulation of transport	6.082036775	7.53E-15	1.55E-13
GOTERM_BP_ALL	GO:0007169~transmembrane receptor protein tyrosine kinase signaling pathway	6.082036775	8.94E-15	1.84E-13
GOTERM_BP_ALL	GO:0002237~response to molecule of bacterial origin	3.818953324	9.16E-15	1.85E-13
GOTERM_BP_ALL	GO:0031401~positive regulation of protein modification process	5.516265912	1.17E-14	2.38E-13
GOTERM_BP_ALL	GO:0050776~regulation of immune response	6.082036775	1.23E-14	2.48E-13
GOTERM_BP_ALL	GO:0007548~sex differentiation	4.95049505	1.25E-14	2.49E-13

GOTERM_BP_ALL	GO:0042770~DNA damage response, signal transduction	3.677510608	1.26E-14	2.52E-13
GOTERM_BP_ALL	GO:0009890~negative regulation of biosynthetic process	10.32531825	1.32E-14	2.62E-13
GOTERM_BP_ALL	GO:0042110~T cell activation	4.526166902	1.38E-14	2.72E-13
GOTERM_BP_ALL	GO:0060341~regulation of cellular localization	6.364922207	1.63E-14	3.19E-13
GOTERM_BP_ALL	GO:0051090~regulation of transcription factor activity	4.101838755	1.73E-14	3.39E-13
GOTERM_BP_ALL	GO:0030155~regulation of cell adhesion	4.667609618	2.52E-14	4.91E-13
GOTERM_BP_ALL	GO:0043523~regulation of neuron apoptosis	3.818953324	3.07E-14	5.94E-13
GOTERM_BP_ALL	GO:0045449~regulation of transcription	28.42998586	4.15E-14	7.96E-13
GOTERM_BP_ALL	GO:0051249~regulation of lymphocyte activation	4.809052334	4.14E-14	7.97E-13
GOTERM_BP_ALL	GO:0044238~primary metabolic process	59.40594059	4.24E-14	8.11E-13
GOTERM_BP_ALL	GO:0043388~positive regulation of DNA binding	3.394625177	4.33E-14	8.24E-13
GOTERM_BP_ALL	GO:0002460~adaptive immune response based on somatic recombination of immune receptors built from immunoglobulin superfamily domains	3.536067893	4.61E-14	8.73E-13
GOTERM_BP_ALL	GO:0002250~adaptive immune response	3.536067893	4.61E-14	8.73E-13
GOTERM_BP_ALL	GO:0048661~positive regulation of smooth muscle cell proliferation	2.404526167	7.31E-14	1.38E-12
GOTERM_BP_ALL	GO:0048754~branching morphogenesis of a tube	3.253182461	7.73E-14	1.45E-12
GOTERM_BP_ALL	GO:0002684~positive regulation of immune system process	6.082036775	7.90E-14	1.48E-12
GOTERM_BP_ALL	GO:0048608~reproductive structure development	4.384724187	9.51E-14	1.77E-12
GOTERM_BP_ALL	GO:0048729~tissue morphogenesis	5.233380481	9.90E-14	1.83E-12
GOTERM_BP_ALL	GO:0051347~positive regulation of transferase activity	6.082036775	1.06E-13	1.96E-12
GOTERM_BP_ALL	GO:0043412~biopolymer modification	19.23620934	1.07E-13	1.96E-12
GOTERM_BP_ALL	GO:0048699~generation of neurons	9.900990099	1.19E-13	2.17E-12
GOTERM_BP_ALL	GO:0010558~negative regulation of macromolecule biosynthetic process	9.759547383	1.29E-13	2.34E-12
GOTERM_BP_ALL	GO:0050867~positive regulation of cell activation	4.101838755	1.36E-13	2.45E-12
GOTERM_BP_ALL	GO:0007050~cell cycle arrest	3.96039604	1.35E-13	2.45E-12
GOTERM_BP_ALL	GO:0006952~defense response	10.46676096	1.61E-13	2.89E-12
GOTERM_BP_ALL	GO:0006979~response to oxidative stress	4.95049505	1.63E-13	2.91E-12
GOTERM_BP_ALL	GO:0001763~morphogenesis of a branching structure	3.394625177	1.67E-13	2.98E-12
GOTERM_BP_ALL	GO:0009617~response to bacterium	5.374823197	1.78E-13	3.15E-12
GOTERM_BP_ALL	GO:0044260~cellular macromolecule metabolic process	47.52475248	1.90E-13	3.36E-12
GOTERM_BP_ALL	GO:0030335~positive regulation of cell migration	3.677510608	1.96E-13	3.45E-12
GOTERM_BP_ALL	GO:0006996~organelle organization	17.39745403	1.98E-13	3.48E-12

GOTERM_BP_ALL	GO:0001819~positive regulation of cytokine production	3.677510608	2.59E-13	4.52E-12
GOTERM_BP_ALL	GO:0006954~inflammatory response	7.072135785	3.39E-13	5.89E-12
GOTERM_BP_ALL	GO:0045792~negative regulation of cell size	3.818953324	3.67E-13	6.34E-12
GOTERM_BP_ALL	GO:0034641~cellular nitrogen compound metabolic process	36.20933522	3.82E-13	6.58E-12
GOTERM_BP_ALL	GO:0032496~response to lipopolysaccharide	3.394625177	4.31E-13	7.37E-12
GOTERM_BP_ALL	GO:0032200~telomere organization	2.263083451	4.30E-13	7.37E-12
GOTERM_BP_ALL	GO:0031347~regulation of defense response	4.526166902	5.52E-13	9.40E-12
GOTERM_BP_ALL	GO:0050863~regulation of T cell activation	4.101838755	5.59E-13	9.47E-12
GOTERM_BP_ALL	GO:0033674~positive regulation of kinase activity	5.799151344	5.94E-13	1.00E-11
GOTERM_BP_ALL	GO:0002697~regulation of immune effector process	3.818953324	6.10E-13	1.03E-11
GOTERM_BP_ALL	GO:0048732~gland development	4.384724187	6.62E-13	1.11E-11
GOTERM_BP_ALL	GO:0016310~phosphorylation	12.16407355	7.20E-13	1.20E-11
GOTERM_BP_ALL	GO:0010551~regulation of specific transcription from RNA polymerase II promoter	3.677510608	7.66E-13	1.27E-11
GOTERM_BP_ALL	GO:0045137~development of primary sexual characteristics	4.243281471	7.74E-13	1.28E-11
GOTERM_BP_ALL	GO:0045860~positive regulation of protein kinase activity	5.657708628	8.43E-13	1.39E-11
GOTERM_BP_ALL	GO:0040007~growth	5.091937765	8.72E-13	1.43E-11
GOTERM_BP_ALL	GO:0001558~regulation of cell growth	5.233380481	1.05E-12	1.72E-11
GOTERM_BP_ALL	GO:0006807~nitrogen compound metabolic process	36.77510608	1.10E-12	1.79E-11
GOTERM_BP_ALL	GO:0032880~regulation of protein localization	4.384724187	1.22E-12	1.97E-11
GOTERM_BP_ALL	GO:0048584~positive regulation of response to stimulus	5.799151344	1.22E-12	1.97E-11
GOTERM_BP_ALL	GO:0044237~cellular metabolic process	56.718529	1.24E-12	2.00E-11
GOTERM_BP_ALL	GO:0070201~regulation of establishment of protein localization	4.101838755	1.36E-12	2.18E-11
GOTERM_BP_ALL	GO:0007165~signal transduction	29.56152758	1.49E-12	2.38E-11
GOTERM_BP_ALL	GO:0033043~regulation of organelle organization	5.516265912	1.60E-12	2.54E-11
GOTERM_BP_ALL	GO:0010627~regulation of protein kinase cascade	5.940594059	1.68E-12	2.66E-11
GOTERM_BP_ALL	GO:0051223~regulation of protein transport	3.96039604	1.90E-12	3.00E-11
GOTERM_BP_ALL	GO:0002696~positive regulation of leukocyte activation	3.818953324	2.06E-12	3.24E-11
GOTERM_BP_ALL	GO:0001503~ossification	3.96039604	2.38E-12	3.73E-11
GOTERM_BP_ALL	GO:0001501~skeletal system development	6.789250354	2.45E-12	3.83E-11
GOTERM_BP_ALL	GO:0050670~regulation of lymphocyte proliferation	3.394625177	2.49E-12	3.87E-11

GOTERM_BP_ALL	GO:0051130~positive regulation of cellular component organization	4.95049505	3.16E-12	4.90E-11
GOTERM_BP_ALL	GO:0070663~regulation of leukocyte proliferation	3.394625177	3.28E-12	5.06E-11
GOTERM_BP_ALL	GO:0032944~regulation of mononuclear cell proliferation	3.394625177	3.28E-12	5.06E-11
GOTERM_BP_ALL	GO:0006464~protein modification process	17.96322489	3.54E-12	5.45E-11
GOTERM_BP_ALL	GO:0009792~embryonic development ending in birth or egg hatching	6.930693069	3.58E-12	5.49E-11
GOTERM_BP_ALL	GO:0051707~response to other organism	6.364922207	4.53E-12	6.92E-11
GOTERM_BP_ALL	GO:0051241~negative regulation of multicellular organismal process	4.667609618	4.80E-12	7.30E-11
GOTERM_BP_ALL	GO:0008630~DNA damage response, signal transduction resulting in induction of apoptosis	2.263083451	4.82E-12	7.30E-11
GOTERM_BP_ALL	GO:0000723~telomere maintenance	2.121640736	4.84E-12	7.31E-11
GOTERM_BP_ALL	GO:0045926~negative regulation of growth	3.818953324	5.16E-12	7.76E-11
GOTERM_BP_ALL	GO:0001655~urogenital system development	3.818953324	5.16E-12	7.76E-11
GOTERM_BP_ALL	GO:0060284~regulation of cell development	5.233380481	5.73E-12	8.59E-11
GOTERM_BP_ALL	GO:0044419~interspecies interaction between organisms	6.223479491	7.69E-12	1.15E-10
GOTERM_BP_ALL	GO:0008406~gonad development	3.818953324	8.02E-12	1.19E-10
GOTERM_BP_ALL	GO:0010629~negative regulation of gene expression	8.769448373	8.17E-12	1.21E-10
GOTERM_BP_ALL	GO:0006139~nucleobase, nucleoside, nucleotide and nucleic acid metabolic process	33.52192362	8.84E-12	1.31E-10
GOTERM_BP_ALL	GO:0043009~chordate embryonic development	6.789250354	9.35E-12	1.38E-10
GOTERM_BP_ALL	GO:0045786~negative regulation of cell cycle	3.253182461	1.16E-11	1.71E-10
GOTERM_BP_ALL	GO:0060348~bone development	3.96039604	1.29E-11	1.89E-10
GOTERM_BP_ALL	GO:0051222~positive regulation of protein transport	2.97029703	1.49E-11	2.17E-10
GOTERM_BP_ALL	GO:0050680~negative regulation of epithelial cell proliferation	1.98019802	1.49E-11	2.17E-10
GOTERM_BP_ALL	GO:0051091~positive regulation of transcription factor activity	2.828854314	1.51E-11	2.18E-10
GOTERM_BP_ALL	GO:0009607~response to biotic stimulus	7.355021216	1.54E-11	2.22E-10
GOTERM_BP_ALL	GO:0048878~chemical homeostasis	8.769448373	1.59E-11	2.28E-10
GOTERM_BP_ALL	GO:0030522~intracellular receptor-mediated signaling pathway	3.111739745	1.81E-11	2.59E-10
GOTERM_BP_ALL	GO:0006796~phosphate metabolic process	13.29561528	2.34E-11	3.33E-10
GOTERM_BP_ALL	GO:0006793~phosphorus metabolic process	13.29561528	2.34E-11	3.33E-10
GOTERM_BP_ALL	GO:0042108~positive regulation of cytokine biosynthetic process	2.545968883	2.33E-11	3.33E-10

GOTERM_BP_ALL	GO:0007507~heart development	5.233380481	2.40E-11	3.38E-10
GOTERM_BP_ALL	GO:0042113~B cell activation	3.111739745	2.39E-11	3.39E-10
GOTERM_BP_ALL	GO:0022403~cell cycle phase	7.637906648	2.48E-11	3.49E-10
GOTERM_BP_ALL	GO:0050920~regulation of chemotaxis	2.121640736	2.80E-11	3.94E-10
GOTERM_BP_ALL	GO:0000018~regulation of DNA recombination	1.98019802	2.85E-11	3.99E-10
GOTERM_BP_ALL	GO:0051329~interphase of mitotic cell cycle	3.536067893	4.72E-11	6.58E-10
GOTERM_BP_ALL	GO:0034097~response to cytokine stimulus	3.111739745	5.36E-11	7.44E-10
GOTERM_BP_ALL	GO:0051345~positive regulation of hydrolase activity	4.667609618	5.41E-11	7.49E-10
GOTERM_BP_ALL	GO:0010552~positive regulation of specific transcription from RNA polymerase II promoter	2.687411598	5.47E-11	7.56E-10
GOTERM_BP_ALL	GO:0046822~regulation of nucleocytoplasmic transport	2.687411598	7.59E-11	1.04E-09
GOTERM_BP_ALL	GO:0010564~regulation of cell cycle process	3.677510608	7.63E-11	1.05E-09
GOTERM_BP_ALL	GO:0032844~regulation of homeostatic process	3.677510608	7.63E-11	1.05E-09
GOTERM_BP_ALL	GO:0051251~positive regulation of lymphocyte activation	3.394625177	8.20E-11	1.12E-09
GOTERM_BP_ALL	GO:0051325~interphase	3.536067893	9.00E-11	1.23E-09
GOTERM_BP_ALL	GO:0033273~response to vitamin	2.828854314	9.77E-11	1.33E-09
GOTERM_BP_ALL	GO:0010648~negative regulation of cell communication	5.516265912	1.02E-10	1.37E-09
GOTERM_BP_ALL	GO:0002252~immune effector process	3.96039604	1.05E-10	1.42E-09
GOTERM_BP_ALL	GO:0010035~response to inorganic substance	4.95049505	1.13E-10	1.52E-09
GOTERM_BP_ALL	GO:0060429~epithelium development	5.233380481	1.18E-10	1.58E-09
GOTERM_BP_ALL	GO:0051051~negative regulation of transport	3.96039604	1.26E-10	1.68E-09
GOTERM_BP_ALL	GO:0016481~negative regulation of transcription	7.920792079	1.35E-10	1.80E-09
GOTERM_BP_ALL	GO:0033157~regulation of intracellular protein transport	2.545968883	1.42E-10	1.89E-09
GOTERM_BP_ALL	GO:0022603~regulation of anatomical structure morphogenesis	5.091937765	1.73E-10	2.29E-09
GOTERM_BP_ALL	GO:0050730~regulation of peptidyl-tyrosine phosphorylation	2.828854314	1.73E-10	2.29E-09
GOTERM_BP_ALL	GO:0030308~negative regulation of cell growth	3.253182461	1.77E-10	2.33E-09
GOTERM_BP_ALL	GO:0007417~central nervous system development	7.496463932	2.07E-10	2.71E-09
GOTERM_BP_ALL	GO:0002761~regulation of myeloid leukocyte differentiation	2.263083451	2.07E-10	2.71E-09
GOTERM_BP_ALL	GO:0051253~negative regulation of RNA metabolic process	6.789250354	2.16E-10	2.81E-09
GOTERM_BP_ALL	GO:0031960~response to corticosteroid stimulus	3.111739745	2.39E-10	3.11E-09

GOTERM_BP_ALL	GO:0042129~regulation of T cell proliferation	2.687411598	2.62E-10	3.39E-09
GOTERM_BP_ALL	GO:0051384~response to glucocorticoid stimulus	2.97029703	3.13E-10	4.04E-09
GOTERM_BP_ALL	GO:0043405~regulation of MAP kinase activity	3.96039604	3.54E-10	4.55E-09
GOTERM_BP_ALL	GO:0001822~kidney development	3.253182461	4.28E-10	5.50E-09
GOTERM_BP_ALL	GO:0032504~multicellular organism reproduction	8.062234795	4.46E-10	5.71E-09
GOTERM_BP_ALL	GO:0048609~reproductive process in a multicellular organism	8.062234795	4.46E-10	5.71E-09
GOTERM_BP_ALL	GO:0002440~production of molecular mediator of immune response	1.98019802	4.54E-10	5.79E-09
GOTERM_BP_ALL	GO:0008637~apoptotic mitochondrial changes	1.98019802	4.54E-10	5.79E-09
GOTERM_BP_ALL	GO:0032103~positive regulation of response to external stimulus	2.687411598	4.66E-10	5.93E-09
GOTERM_BP_ALL	GO:0030217~T cell differentiation	2.687411598	6.17E-10	7.82E-09
GOTERM_BP_ALL	GO:0042035~regulation of cytokine biosynthetic process	2.828854314	8.49E-10	1.07E-08
GOTERM_BP_ALL	GO:0009968~negative regulation of signal transduction	4.95049505	8.91E-10	1.12E-08
GOTERM_BP_ALL	GO:0001952~regulation of cell-matrix adhesion	1.838755304	9.49E-10	1.19E-08
GOTERM_BP_ALL	GO:0048872~homeostasis of number of cells	3.253182461	9.86E-10	1.23E-08
GOTERM_BP_ALL	GO:0010638~positive regulation of organelle organization	2.97029703	1.04E-09	1.29E-08
GOTERM_BP_ALL	GO:0042698~ovulation cycle	2.687411598	1.06E-09	1.32E-08
GOTERM_BP_ALL	GO:0051276~chromosome organization	7.920792079	1.07E-09	1.32E-08
GOTERM_BP_ALL	GO:0001525~angiogenesis	3.96039604	1.10E-09	1.36E-08
GOTERM_BP_ALL	GO:0051053~negative regulation of DNA metabolic process	2.121640736	1.12E-09	1.39E-08
GOTERM_BP_ALL	GO:0002009~morphogenesis of an epithelium	3.253182461	1.21E-09	1.48E-08
GOTERM_BP_ALL	GO:0045785~positive regulation of cell adhesion	2.545968883	1.25E-09	1.54E-08
GOTERM_BP_ALL	GO:0045892~negative regulation of transcription, DNA-dependent	6.506364922	1.26E-09	1.54E-08
GOTERM_BP_ALL	GO:0050795~regulation of behavior	2.263083451	1.34E-09	1.64E-08
GOTERM_BP_ALL	GO:0050727~regulation of inflammatory response	2.828854314	1.39E-09	1.69E-08
GOTERM_BP_ALL	GO:0030099~myeloid cell differentiation	3.111739745	1.43E-09	1.73E-08
GOTERM_BP_ALL	GO:0051960~regulation of nervous system development	4.526166902	1.49E-09	1.80E-08
GOTERM_BP_ALL	GO:0016447~somatic recombination of immunoglobulin gene segments	1.555869873	1.52E-09	1.83E-08
GOTERM_BP_ALL	GO:0044092~negative regulation of molecular function	6.223479491	1.69E-09	2.03E-08
GOTERM_BP_ALL	GO:0046545~development of primary female sexual characteristics	2.687411598	1.79E-09	2.14E-08
GOTERM_BP_ALL	GO:0046660~female sex differentiation	2.687411598	1.79E-09	2.14E-08

GOTERM_BP_ALL	GO:0002443~leukocyte mediated immunity	2.97029703	2.03E-09	2.42E-08
GOTERM_BP_ALL	GO:0051129~negative regulation of cellular component organization	3.818953324	2.06E-09	2.45E-08
GOTERM_BP_ALL	GO:0006275~regulation of DNA replication	2.545968883	2.19E-09	2.60E-08
GOTERM_BP_ALL	GO:0002449~lymphocyte mediated immunity	2.687411598	2.30E-09	2.73E-08
GOTERM_BP_ALL	GO:0050671~positive regulation of lymphocyte proliferation	2.404526167	2.51E-09	2.97E-08
GOTERM_BP_ALL	GO:0006289~nucleotide-excision repair	2.404526167	2.51E-09	2.97E-08
GOTERM_BP_ALL	GO:0050921~positive regulation of chemotaxis	1.838755304	2.60E-09	3.06E-08
GOTERM_BP_ALL	GO:0045619~regulation of lymphocyte differentiation	2.545968883	2.87E-09	3.38E-08
GOTERM_BP_ALL	GO:0032386~regulation of intracellular transport	2.687411598	2.95E-09	3.46E-08
GOTERM_BP_ALL	GO:0002562~somatic diversification of immune receptors via germline recombination within a single locus	1.697312588	3.22E-09	3.77E-08
GOTERM_BP_ALL	GO:0016444~somatic cell DNA recombination	1.697312588	3.22E-09	3.77E-08
GOTERM_BP_ALL	GO:0051054~positive regulation of DNA metabolic process	2.404526167	3.37E-09	3.92E-08
GOTERM_BP_ALL	GO:0070665~positive regulation of leukocyte proliferation	2.404526167	3.37E-09	3.92E-08
GOTERM_BP_ALL	GO:0032946~positive regulation of mononuclear cell proliferation	2.404526167	3.37E-09	3.92E-08
GOTERM_BP_ALL	GO:0010740~positive regulation of protein kinase cascade	4.101838755	3.93E-09	4.56E-08
GOTERM_BP_ALL	GO:0010165~response to X-ray	1.414427157	4.09E-09	4.74E-08
GOTERM_BP_ALL	GO:0002377~immunoglobulin production	1.838755304	4.14E-09	4.79E-08
GOTERM_BP_ALL	GO:0045787~positive regulation of cell cycle	2.404526167	4.48E-09	5.16E-08
GOTERM_BP_ALL	GO:0070271~protein complex biogenesis	7.920792079	4.61E-09	5.29E-08
GOTERM_BP_ALL	GO:0006461~protein complex assembly	7.920792079	4.61E-09	5.29E-08
GOTERM_BP_ALL	GO:0048511~rhythmic process	3.536067893	5.26E-09	6.02E-08
GOTERM_BP_ALL	GO:0051046~regulation of secretion	4.526166902	5.28E-09	6.03E-08
GOTERM_BP_ALL	GO:0019725~cellular homeostasis	7.496463932	5.48E-09	6.24E-08
GOTERM_BP_ALL	GO:0043408~regulation of MAPKKK cascade	3.253182461	5.50E-09	6.25E-08
GOTERM_BP_ALL	GO:0030518~steroid hormone receptor signaling pathway	2.404526167	5.92E-09	6.71E-08
GOTERM_BP_ALL	GO:0045580~regulation of T cell differentiation	2.263083451	6.72E-09	7.60E-08
GOTERM_BP_ALL	GO:0050864~regulation of B cell activation	2.263083451	6.72E-09	7.60E-08
GOTERM_BP_ALL	GO:0060562~epithelial tube morphogenesis	2.545968883	8.02E-09	9.04E-08
GOTERM_BP_ALL	GO:0043406~positive regulation of MAP kinase activity	3.111739745	8.42E-09	9.46E-08

GOTERM_BP_ALL	GO:0051353~positive regulation of oxidoreductase activity	1.697312588	9.16E-09	1.03E-07
GOTERM_BP_ALL	GO:0042326~negative regulation of phosphorylation	2.121640736	9.56E-09	1.07E-07
GOTERM_BP_ALL	GO:0050870~positive regulation of T cell activation	2.687411598	9.55E-09	1.07E-07
GOTERM_BP_ALL	GO:0000122~negative regulation of transcription from RNA polymerase II promoter	5.233380481	9.79E-09	1.09E-07
GOTERM_BP_ALL	GO:0048863~stem cell differentiation	1.838755304	9.92E-09	1.10E-07
GOTERM_BP_ALL	GO:0006308~DNA catabolic process	2.404526167	1.02E-08	1.12E-07
GOTERM_BP_ALL	GO:0048871~multicellular organismal homeostasis	2.828854314	1.04E-08	1.14E-07
GOTERM_BP_ALL	GO:0001836~release of cytochrome c from mitochondria	1.555869873	1.08E-08	1.18E-07
GOTERM_BP_ALL	GO:0052547~regulation of peptidase activity	2.828854314	1.27E-08	1.40E-07
GOTERM_BP_ALL	GO:0010810~regulation of cell-substrate adhesion	2.121640736	1.32E-08	1.44E-07
GOTERM_BP_ALL	GO:0048598~embryonic morphogenesis	5.657708628	1.43E-08	1.56E-07
GOTERM_BP_ALL	GO:0050767~regulation of neurogenesis	3.96039604	1.46E-08	1.59E-07
GOTERM_BP_ALL	GO:0002200~somatic diversification of immune receptors	1.697312588	1.48E-08	1.61E-07
GOTERM_BP_ALL	GO:0007569~cell aging	1.838755304	1.49E-08	1.62E-07
GOTERM_BP_ALL	GO:0051348~negative regulation of transferase activity	2.97029703	1.54E-08	1.66E-07
GOTERM_BP_ALL	GO:0032355~response to estradiol stimulus	2.263083451	1.60E-08	1.72E-07
GOTERM_BP_ALL	GO:0051341~regulation of oxidoreductase activity	1.98019802	1.79E-08	1.92E-07
GOTERM_BP_ALL	GO:0007610~behavior	7.355021216	1.82E-08	1.95E-07
GOTERM_BP_ALL	GO:0043281~regulation of caspase activity	2.687411598	1.84E-08	1.97E-07
GOTERM_BP_ALL	GO:0016445~somatic diversification of immunoglobulins	1.555869873	1.89E-08	2.02E-07
GOTERM_BP_ALL	GO:0051336~regulation of hydrolase activity	5.940594059	2.12E-08	2.26E-07
GOTERM_BP_ALL	GO:0032269~negative regulation of cellular protein metabolic process	4.101838755	2.16E-08	2.29E-07
GOTERM_BP_ALL	GO:0048520~positive regulation of behavior	1.838755304	2.21E-08	2.34E-07
GOTERM_BP_ALL	GO:0002381~immunoglobulin production during immune response	1.272984441	2.37E-08	2.50E-07
GOTERM_BP_ALL	GO:0030183~B cell differentiation	2.121640736	2.42E-08	2.55E-07
GOTERM_BP_ALL	GO:0045936~negative regulation of phosphate metabolic process	2.121640736	2.42E-08	2.55E-07
GOTERM_BP_ALL	GO:0010563~negative regulation of phosphorus metabolic process	2.121640736	2.42E-08	2.55E-07
GOTERM_BP_ALL	GO:0000077~DNA damage checkpoint	2.121640736	2.42E-08	2.55E-07
GOTERM_BP_ALL	GO:0009612~response to mechanical stimulus	2.263083451	2.75E-08	2.89E-07
GOTERM_BP_ALL	GO:0019724~B cell mediated immunity	2.263083451	2.75E-08	2.89E-07

GOTERM_BP_ALL	GO:0048771~tissue remodeling	2.263083451	2.75E-08	2.89E-07
GOTERM_BP_ALL	GO:0008585~female gonad development	2.404526167	2.78E-08	2.91E-07
GOTERM_BP_ALL	GO:0045165~cell fate commitment	3.536067893	2.83E-08	2.96E-07
GOTERM_BP_ALL	GO:0048585~negative regulation of response to stimulus	2.97029703	3.20E-08	3.34E-07
GOTERM_BP_ALL	GO:0031349~positive regulation of defense response	2.545968883	3.23E-08	3.36E-07
GOTERM_BP_ALL	GO:0052548~regulation of endopeptidase activity	2.687411598	3.42E-08	3.55E-07
GOTERM_BP_ALL	GO:0001649~osteoblast differentiation	1.98019802	3.48E-08	3.60E-07
GOTERM_BP_ALL	GO:0019216~regulation of lipid metabolic process	3.111739745	4.76E-08	4.91E-07
GOTERM_BP_ALL	GO:0019058~viral infectious cycle	1.98019802	4.78E-08	4.92E-07
GOTERM_BP_ALL	GO:0051248~negative regulation of protein metabolic process	4.101838755	5.02E-08	5.15E-07
GOTERM_BP_ALL	GO:0044057~regulation of system process	5.516265912	5.24E-08	5.37E-07
GOTERM_BP_ALL	GO:0042306~regulation of protein import into nucleus	1.98019802	6.49E-08	6.63E-07
GOTERM_BP_ALL	GO:0031570~DNA integrity checkpoint	2.121640736	7.47E-08	7.61E-07
GOTERM_BP_ALL	GO:0060627~regulation of vesicle-mediated transport	2.828854314	8.42E-08	8.54E-07
GOTERM_BP_ALL	GO:0032768~regulation of monooxygenase activity	1.555869873	8.41E-08	8.55E-07
GOTERM_BP_ALL	GO:0001818~negative regulation of cytokine production	1.838755304	9.17E-08	9.28E-07
GOTERM_BP_ALL	GO:0040013~negative regulation of locomotion	2.263083451	9.60E-08	9.69E-07
GOTERM_BP_ALL	GO:0002703~regulation of leukocyte mediated immunity	2.263083451	9.60E-08	9.69E-07
GOTERM_BP_ALL	GO:0009636~response to toxin	2.263083451	9.60E-08	9.69E-07
GOTERM_BP_ALL	GO:0001889~liver development	2.121640736	9.72E-08	9.78E-07
GOTERM_BP_ALL	GO:0006959~humoral immune response	2.545968883	1.12E-07	1.13E-06
GOTERM_BP_ALL	GO:0022415~viral reproductive process	2.263083451	1.21E-07	1.21E-06
GOTERM_BP_ALL	GO:0022602~ovulation cycle process	2.263083451	1.21E-07	1.21E-06
GOTERM_BP_ALL	GO:0016064~immunoglobulin mediated immune response	2.121640736	1.26E-07	1.26E-06
GOTERM_BP_ALL	GO:0000079~regulation of cyclin-dependent protein kinase activity	2.121640736	1.26E-07	1.26E-06
GOTERM_BP_ALL	GO:0000165~MAPKKK cascade	3.96039604	1.33E-07	1.33E-06
GOTERM_BP_ALL	GO:0009650~UV protection	1.131541726	1.37E-07	1.36E-06
GOTERM_BP_ALL	GO:0030324~lung development	2.828854314	1.41E-07	1.40E-06
GOTERM_BP_ALL	GO:0018193~peptidyl-amino acid modification	3.677510608	1.44E-07	1.43E-06
GOTERM_BP_ALL	GO:0051271~negative regulation of cell motion	2.263083451	1.52E-07	1.50E-06
GOTERM_BP_ALL	GO:0050731~positive regulation of peptidyl-tyrosine phosphorylation	1.98019802	1.54E-07	1.52E-06
GOTERM_BP_ALL	GO:0033673~negative regulation of kinase activity	2.687411598	1.56E-07	1.53E-06

GOTERM_BP_ALL	GO:0002822~regulation of adaptive immune response based on somatic recombination of immune receptors built from immunoglobulin superfamily domains	2.121640736	1.61E-07	1.58E-06
GOTERM_BP_ALL	GO:0045471~response to ethanol	2.263083451	1.90E-07	1.86E-06
GOTERM_BP_ALL	GO:0010608~posttranscriptional regulation of gene expression	4.243281471	1.94E-07	1.90E-06
GOTERM_BP_ALL	GO:0021700~developmental maturation	2.828854314	1.96E-07	1.91E-06
GOTERM_BP_ALL	GO:0001701~in utero embryonic development	3.818953324	1.98E-07	1.93E-06
GOTERM_BP_ALL	GO:0045428~regulation of nitric oxide biosynthetic process	1.555869873	1.99E-07	1.93E-06
GOTERM_BP_ALL	GO:0046661~male sex differentiation	2.404526167	2.02E-07	1.96E-06
GOTERM_BP_ALL	GO:0002819~regulation of adaptive immune response	2.121640736	2.06E-07	1.99E-06
GOTERM_BP_ALL	GO:0055082~cellular chemical homeostasis	6.082036775	2.18E-07	2.10E-06
GOTERM_BP_ALL	GO:0030323~respiratory tube development	2.828854314	2.31E-07	2.22E-06
GOTERM_BP_ALL	GO:0051092~positive regulation of NF-kappaB transcription factor activity	1.838755304	2.35E-07	2.25E-06
GOTERM_BP_ALL	GO:0050801~ion homeostasis	6.364922207	2.42E-07	2.32E-06
GOTERM_BP_ALL	GO:0050900~leukocyte migration	2.121640736	2.61E-07	2.49E-06
GOTERM_BP_ALL	GO:0030336~negative regulation of cell migration	2.121640736	2.61E-07	2.49E-06
GOTERM_BP_ALL	GO:0009896~positive regulation of catabolic process	1.98019802	2.64E-07	2.52E-06
GOTERM_BP_ALL	GO:0080135~regulation of cellular response to stress	2.828854314	2.71E-07	2.57E-06
GOTERM_BP_ALL	GO:0043525~positive regulation of neuron apoptosis	1.414427157	3.02E-07	2.86E-06
GOTERM_BP_ALL	GO:0002208~somatic diversification of immunoglobulins during immune response	1.131541726	3.15E-07	2.98E-06
GOTERM_BP_ALL	GO:0002204~somatic recombination of immunoglobulin genes during immune response	1.131541726	3.15E-07	2.98E-06
GOTERM_BP_ALL	GO:0045190~isotype switching	1.131541726	3.15E-07	2.98E-06
GOTERM_BP_ALL	GO:0048145~regulation of fibroblast proliferation	1.697312588	3.36E-07	3.16E-06
GOTERM_BP_ALL	GO:0006873~cellular ion homeostasis	5.940594059	3.77E-07	3.55E-06
GOTERM_BP_ALL	GO:0010952~positive regulation of peptidase activity	2.121640736	4.14E-07	3.88E-06
GOTERM_BP_ALL	GO:0043280~positive regulation of caspase activity	2.121640736	4.14E-07	3.88E-06
GOTERM_BP_ALL	GO:0007093~mitotic cell cycle checkpoint	1.838755304	4.18E-07	3.92E-06
GOTERM_BP_ALL	GO:0043524~negative regulation of neuron apoptosis	1.98019802	4.39E-07	4.11E-06
GOTERM_BP_ALL	GO:0006897~endocytosis	4.243281471	4.77E-07	4.45E-06
GOTERM_BP_ALL	GO:0010324~membrane invagination	4.243281471	4.77E-07	4.45E-06

GOTERM_BP_ALL	GO:0048589~developmental growth	2.545968883	4.88E-07	4.54E-06
GOTERM_BP_ALL	GO:0006469~negative regulation of protein kinase activity	2.545968883	4.88E-07	4.54E-06
GOTERM_BP_ALL	GO:0007154~cell communication	9.900990099	4.91E-07	4.56E-06
GOTERM_BP_ALL	GO:0031099~regeneration	2.263083451	5.44E-07	5.04E-06
GOTERM_BP_ALL	GO:0001656~metanephros development	1.838755304	5.51E-07	5.09E-06
GOTERM_BP_ALL	GO:0051259~protein oligomerization	3.677510608	5.81E-07	5.36E-06
GOTERM_BP_ALL	GO:0060541~respiratory system development	2.828854314	5.83E-07	5.36E-06
GOTERM_BP_ALL	GO:0032388~positive regulation of intracellular transport	1.555869873	6.24E-07	5.73E-06
GOTERM_BP_ALL	GO:0045638~negative regulation of myeloid cell differentiation	1.555869873	6.24E-07	5.73E-06
GOTERM_BP_ALL	GO:0031329~regulation of cellular catabolic process	2.121640736	6.42E-07	5.88E-06
GOTERM_BP_ALL	GO:0030100~regulation of endocytosis	2.121640736	6.42E-07	5.88E-06
GOTERM_BP_ALL	GO:0032770~positive regulation of monooxygenase activity	1.131541726	6.55E-07	5.98E-06
GOTERM_BP_ALL	GO:0007369~gastrulation	2.263083451	6.62E-07	6.04E-06
GOTERM_BP_ALL	GO:0016032~viral reproduction	2.263083451	8.04E-07	7.32E-06
GOTERM_BP_ALL	GO:0051321~meiotic cell cycle	2.687411598	8.12E-07	7.38E-06
GOTERM_BP_ALL	GO:0043434~response to peptide hormone stimulus	3.394625177	8.20E-07	7.43E-06
GOTERM_BP_ALL	GO:0043086~negative regulation of catalytic activity	4.809052334	8.31E-07	7.51E-06
GOTERM_BP_ALL	GO:0006919~activation of caspase activity	1.98019802	8.98E-07	8.10E-06
GOTERM_BP_ALL	GO:0018108~peptidyl-tyrosine phosphorylation	1.838755304	9.33E-07	8.40E-06
GOTERM_BP_ALL	GO:0030879~mammary gland development	2.121640736	9.77E-07	8.78E-06
GOTERM_BP_ALL	GO:0042060~wound healing	3.818953324	1.00E-06	8.98E-06
GOTERM_BP_ALL	GO:0050927~positive regulation of positive chemotaxis	1.272984441	1.08E-06	9.68E-06
GOTERM_BP_ALL	GO:0050926~regulation of positive chemotaxis	1.272984441	1.08E-06	9.68E-06
GOTERM_BP_ALL	GO:0006298~mismatch repair	1.414427157	1.09E-06	9.76E-06
GOTERM_BP_ALL	GO:0042102~positive regulation of T cell proliferation	1.697312588	1.13E-06	1.01E-05
GOTERM_BP_ALL	GO:0000726~non-recombinational repair	1.131541726	1.26E-06	1.12E-05
GOTERM_BP_ALL	GO:0033135~regulation of peptidyl-serine phosphorylation	1.131541726	1.26E-06	1.12E-05
GOTERM_BP_ALL	GO:0007178~transmembrane receptor protein serine/threonine kinase signaling pathway	2.687411598	1.27E-06	1.13E-05
GOTERM_BP_ALL	GO:0002683~negative regulation of immune system process	2.404526167	1.28E-06	1.13E-05
GOTERM_BP_ALL	GO:0000082~G1/S transition of mitotic cell cycle	1.98019802	1.40E-06	1.24E-05
GOTERM_BP_ALL	GO:0046546~development of primary male sexual characteristics	2.121640736	1.46E-06	1.29E-05

GOTERM_BP_ALL	GO:0032582~negative regulation of gene-specific transcription	1.838755304	1.53E-06	1.35E-05
GOTERM_BP_ALL	GO:0018212~peptidyl-tyrosine modification	1.838755304	1.53E-06	1.35E-05
GOTERM_BP_ALL	GO:0000302~response to reactive oxygen species	2.263083451	1.68E-06	1.48E-05
GOTERM_BP_ALL	GO:0050871~positive regulation of B cell activation	1.555869873	1.69E-06	1.48E-05
GOTERM_BP_ALL	GO:0010565~regulation of cellular ketone metabolic process	1.98019802	1.74E-06	1.52E-05
GOTERM_BP_ALL	GO:0001953~negative regulation of cell-matrix adhesion	0.99009901	1.87E-06	1.64E-05
GOTERM_BP_ALL	GO:0045073~regulation of chemokine biosynthetic process	0.99009901	1.87E-06	1.64E-05
GOTERM_BP_ALL	GO:0006303~double-strand break repair via nonhomologous end joining	0.99009901	1.87E-06	1.64E-05
GOTERM_BP_ALL	GO:0019217~regulation of fatty acid metabolic process	1.838755304	1.94E-06	1.69E-05
GOTERM_BP_ALL	GO:0002700~regulation of production of molecular mediator of immune response	1.697312588	1.96E-06	1.71E-05
GOTERM_BP_ALL	GO:0009894~regulation of catabolic process	2.545968883	2.07E-06	1.80E-05
GOTERM_BP_ALL	GO:0007420~brain development	4.809052334	2.15E-06	1.86E-05
GOTERM_BP_ALL	GO:0007398~ectoderm development	3.818953324	2.20E-06	1.90E-05
GOTERM_BP_ALL	GO:0032642~regulation of chemokine production	1.131541726	2.26E-06	1.95E-05
GOTERM_BP_ALL	GO:0000060~protein import into nucleus, translocation	1.555869873	2.30E-06	1.97E-05
GOTERM_BP_ALL	GO:0006284~base-excision repair	1.414427157	2.30E-06	1.98E-05
GOTERM_BP_ALL	GO:0010038~response to metal ion	2.97029703	2.35E-06	2.01E-05
GOTERM_BP_ALL	GO:0055066~di-, tri-valent inorganic cation homeostasis	4.243281471	2.65E-06	2.27E-05
GOTERM_BP_ALL	GO:0045429~positive regulation of nitric oxide biosynthetic process	1.272984441	2.68E-06	2.29E-05
GOTERM_BP_ALL	GO:0007126~meiosis	2.545968883	2.78E-06	2.37E-05
GOTERM_BP_ALL	GO:0051327~M phase of meiotic cell cycle	2.545968883	2.78E-06	2.37E-05
GOTERM_BP_ALL	GO:0051047~positive regulation of secretion	2.687411598	2.97E-06	2.52E-05
GOTERM_BP_ALL	GO:0007389~pattern specification process	4.526166902	3.08E-06	2.61E-05
GOTERM_BP_ALL	GO:0044085~cellular component biogenesis	11.31541726	3.08E-06	2.61E-05
GOTERM_BP_ALL	GO:0010720~positive regulation of cell development	2.121640736	3.11E-06	2.63E-05
GOTERM_BP_ALL	GO:0007179~transforming growth factor beta receptor signaling pathway	1.98019802	3.23E-06	2.72E-05
GOTERM_BP_ALL	GO:0030888~regulation of B cell proliferation	1.414427157	3.25E-06	2.73E-05
GOTERM_BP_ALL	GO:0043933~macromolecular complex subunit organization	8.769448373	3.25E-06	2.73E-05

GOTERM_BP_ALL	GO:0022607~cellular component assembly	10.32531825	3.27E-06	2.74E-05
GOTERM_BP_ALL	GO:0019221~cytokine-mediated signaling pathway	2.121640736	3.72E-06	3.11E-05
GOTERM_BP_ALL	GO:0008584~male gonad development	1.838755304	3.82E-06	3.18E-05
GOTERM_BP_ALL	GO:0050866~negative regulation of cell activation	1.98019802	3.93E-06	3.27E-05
GOTERM_BP_ALL	GO:0010812~negative regulation of cell-substrate adhesion	0.99009901	3.96E-06	3.29E-05
GOTERM_BP_ALL	GO:0002244~hemopoietic progenitor cell differentiation	0.99009901	3.96E-06	3.29E-05
GOTERM_BP_ALL	GO:0045670~regulation of osteoclast differentiation	1.272984441	4.04E-06	3.35E-05
GOTERM_BP_ALL	GO:0019059~initiation of viral infection	1.272984441	4.04E-06	3.35E-05
GOTERM_BP_ALL	GO:0000718~nucleotide-excision repair, DNA damage removal	1.272984441	4.04E-06	3.35E-05
GOTERM_BP_ALL	GO:0002263~cell activation during immune response	1.555869873	4.09E-06	3.38E-05
GOTERM_BP_ALL	GO:0002366~leukocyte activation during immune response	1.555869873	4.09E-06	3.38E-05
GOTERM_BP_ALL	GO:0031331~positive regulation of cellular catabolic process	1.555869873	4.09E-06	3.38E-05
GOTERM_BP_ALL	GO:0001776~leukocyte homeostasis	1.555869873	4.09E-06	3.38E-05
GOTERM_BP_ALL	GO:0033160~positive regulation of protein import into nucleus, translocation	0.848656294	4.37E-06	3.61E-05
GOTERM_BP_ALL	GO:0045910~negative regulation of DNA recombination	0.848656294	4.37E-06	3.61E-05
GOTERM_BP_ALL	GO:0002320~lymphoid progenitor cell differentiation	0.848656294	4.37E-06	3.61E-05
GOTERM_BP_ALL	GO:0030595~leukocyte chemotaxis	1.555869873	5.37E-06	4.42E-05
GOTERM_BP_ALL	GO:0010639~negative regulation of organelle organization	2.263083451	5.42E-06	4.45E-05
GOTERM_BP_ALL	GO:0001894~tissue homeostasis	1.98019802	5.74E-06	4.70E-05
GOTERM_BP_ALL	GO:0045765~regulation of angiogenesis	1.98019802	5.74E-06	4.70E-05
GOTERM_BP_ALL	GO:0032147~activation of protein kinase activity	2.687411598	5.73E-06	4.70E-05
GOTERM_BP_ALL	GO:0045088~regulation of innate immune response	1.838755304	5.82E-06	4.76E-05
GOTERM_BP_ALL	GO:0002706~regulation of lymphocyte mediated immunity	1.838755304	5.82E-06	4.76E-05
GOTERM_BP_ALL	GO:0033044~regulation of chromosome organization	1.414427157	6.20E-06	5.05E-05
GOTERM_BP_ALL	GO:0032649~regulation of interferon-gamma production	1.414427157	6.20E-06	5.05E-05
GOTERM_BP_ALL	GO:0060393~regulation of pathway-restricted SMAD protein phosphorylation	1.131541726	6.29E-06	5.12E-05
GOTERM_BP_ALL	GO:0045089~positive regulation of innate immune response	1.697312588	6.69E-06	5.44E-05
GOTERM_BP_ALL	GO:0045834~positive regulation of lipid metabolic process	1.697312588	6.69E-06	5.44E-05
GOTERM_BP_ALL	GO:0048610~reproductive cellular process	3.253182461	7.02E-06	5.69E-05

GOTERM_BP_ALL	GO:0007498~mesoderm development	2.121640736	7.38E-06	5.98E-05
GOTERM_BP_ALL	GO:0008354~germ cell migration	0.99009901	7.61E-06	6.15E-05
GOTERM_BP_ALL	GO:0014910~regulation of smooth muscle cell migration	0.99009901	7.61E-06	6.15E-05
GOTERM_BP_ALL	GO:0032680~regulation of tumor necrosis factor production	1.414427157	8.37E-06	6.75E-05
GOTERM_BP_ALL	GO:0001101~response to acid	1.272984441	8.54E-06	6.88E-05
GOTERM_BP_ALL	GO:0048469~cell maturation	2.121640736	8.69E-06	6.97E-05
GOTERM_BP_ALL	GO:0051783~regulation of nuclear division	1.838755304	8.69E-06	6.98E-05
GOTERM_BP_ALL	GO:0007088~regulation of mitosis	1.838755304	8.69E-06	6.98E-05
GOTERM_BP_ALL	GO:0060326~cell chemotaxis	1.555869873	9.00E-06	7.20E-05
GOTERM_BP_ALL	GO:0007259~JAK-STAT cascade	1.555869873	9.00E-06	7.20E-05
GOTERM_BP_ALL	GO:0006260~DNA replication	3.536067893	9.66E-06	7.71E-05
GOTERM_BP_ALL	GO:0043410~positive regulation of MAPKKK cascade	1.697312588	1.04E-05	8.29E-05
GOTERM_BP_ALL	GO:0031400~negative regulation of protein modification process	2.687411598	1.06E-05	8.44E-05
GOTERM_BP_ALL	GO:0001936~regulation of endothelial cell proliferation	1.414427157	1.12E-05	8.86E-05
GOTERM_BP_ALL	GO:0001933~negative regulation of protein amino acid phosphorylation	1.414427157	1.12E-05	8.86E-05
GOTERM_BP_ALL	GO:0051893~regulation of focal adhesion formation	0.848656294	1.12E-05	8.88E-05
GOTERM_BP_ALL	GO:0002902~regulation of B cell apoptosis	0.848656294	1.12E-05	8.88E-05
GOTERM_BP_ALL	GO:0055093~response to hyperoxia	0.848656294	1.12E-05	8.88E-05
GOTERM_BP_ALL	GO:0010224~response to UV-B	0.848656294	1.12E-05	8.88E-05
GOTERM_BP_ALL	GO:0006953~acute-phase response	1.555869873	1.15E-05	9.09E-05
GOTERM_BP_ALL	GO:0046637~regulation of alpha-beta T cell differentiation	1.272984441	1.21E-05	9.51E-05
GOTERM_BP_ALL	GO:0019827~stem cell maintenance	1.272984441	1.21E-05	9.51E-05
GOTERM_BP_ALL	GO:0030278~regulation of ossification	2.121640736	1.39E-05	1.10E-04
GOTERM_BP_ALL	GO:0019538~protein metabolic process	25.03536068	1.40E-05	1.10E-04
GOTERM_BP_ALL	GO:0050714~positive regulation of protein secretion	1.555869873	1.46E-05	1.14E-04
GOTERM_BP_ALL	GO:0045639~positive regulation of myeloid cell differentiation	1.414427157	1.47E-05	1.15E-04
GOTERM_BP_ALL	GO:0032755~positive regulation of interleukin-6 production	1.131541726	1.50E-05	1.17E-04
GOTERM_BP_ALL	GO:0000725~recombinational repair	1.131541726	1.50E-05	1.17E-04
GOTERM_BP_ALL	GO:0000724~double-strand break repair via homologous recombination	1.131541726	1.50E-05	1.17E-04
GOTERM_BP_ALL	GO:0065003~macromolecular complex assembly	8.062234795	1.51E-05	1.18E-04
GOTERM_BP_ALL	GO:0051100~negative regulation of binding	1.838755304	1.53E-05	1.19E-04
GOTERM_BP_ALL	GO:0032102~negative regulation of response to external stimulus	1.697312588	1.58E-05	1.23E-04
GOTERM_BP_ALL	GO:0002637~regulation of immunoglobulin production	1.272984441	1.67E-05	1.30E-04

GOTERM_BP_ALL	GO:0000737~DNA catabolic process, endonucleolytic	1.272984441	1.67E-05	1.30E-04
GOTERM_BP_ALL	GO:0048864~stem cell development	1.272984441	1.67E-05	1.30E-04
GOTERM_BP_ALL	GO:0008544~epidermis development	3.394625177	1.75E-05	1.36E-04
GOTERM_BP_ALL	GO:0007281~germ cell development	2.404526167	1.80E-05	1.39E-04
GOTERM_BP_ALL	GO:0043392~negative regulation of DNA binding	1.697312588	1.93E-05	1.49E-04
GOTERM_BP_ALL	GO:0014031~mesenchymal cell development	1.697312588	1.93E-05	1.49E-04
GOTERM_BP_ALL	GO:0048762~mesenchymal cell differentiation	1.697312588	1.93E-05	1.49E-04
GOTERM_BP_ALL	GO:0030182~neuron differentiation	5.940594059	2.01E-05	1.55E-04
GOTERM_BP_ALL	GO:0016049~cell growth	1.838755304	2.19E-05	1.68E-04
GOTERM_BP_ALL	GO:0010676~positive regulation of cellular carbohydrate metabolic process	1.131541726	2.21E-05	1.70E-04
GOTERM_BP_ALL	GO:0002685~regulation of leukocyte migration	1.131541726	2.21E-05	1.70E-04
GOTERM_BP_ALL	GO:0045913~positive regulation of carbohydrate metabolic process	1.131541726	2.21E-05	1.70E-04
GOTERM_BP_ALL	GO:0033077~T cell differentiation in the thymus	1.272984441	2.28E-05	1.74E-04
GOTERM_BP_ALL	GO:0045732~positive regulation of protein catabolic process	1.272984441	2.28E-05	1.74E-04
GOTERM_BP_ALL	GO:0048662~negative regulation of smooth muscle cell proliferation	0.99009901	2.28E-05	1.74E-04
GOTERM_BP_ALL	GO:0007162~negative regulation of cell adhesion	1.555869873	2.29E-05	1.75E-04
GOTERM_BP_ALL	GO:0045667~regulation of osteoblast differentiation	1.555869873	2.29E-05	1.75E-04
GOTERM_BP_ALL	GO:0006290~pyrimidine dimer repair	0.707213579	2.32E-05	1.77E-04
GOTERM_BP_ALL	GO:0060485~mesenchyme development	1.697312588	2.34E-05	1.78E-04
GOTERM_BP_ALL	GO:0043536~positive regulation of blood vessel endothelial cell migration	0.848656294	2.42E-05	1.84E-04
GOTERM_BP_ALL	GO:0033158~regulation of protein import into nucleus, translocation	0.848656294	2.42E-05	1.84E-04
GOTERM_BP_ALL	GO:0001657~ureteric bud development	1.414427157	2.48E-05	1.88E-04
GOTERM_BP_ALL	GO:0032943~mononuclear cell proliferation	1.555869873	2.84E-05	2.14E-04
GOTERM_BP_ALL	GO:0002699~positive regulation of immune effector process	1.555869873	2.84E-05	2.14E-04
GOTERM_BP_ALL	GO:0070661~leukocyte proliferation	1.555869873	2.84E-05	2.14E-04
GOTERM_BP_ALL	GO:0030162~regulation of proteolysis	1.697312588	2.83E-05	2.14E-04
GOTERM_BP_ALL	GO:0000278~mitotic cell cycle	5.233380481	2.84E-05	2.14E-04
GOTERM_BP_ALL	GO:0007267~cell-cell signaling	7.355021216	2.89E-05	2.17E-04
GOTERM_BP_ALL	GO:0045740~positive regulation of DNA replication	1.272984441	3.06E-05	2.29E-04
GOTERM_BP_ALL	GO:0046634~regulation of alpha-beta T cell activation	1.414427157	3.16E-05	2.37E-04
GOTERM_BP_ALL	GO:0032675~regulation of interleukin-6 production	1.414427157	3.16E-05	2.37E-04

GOTERM_BP_ALL	GO:0045884~regulation of survival gene product expression	1.131541726	3.19E-05	2.39E-04
GOTERM_BP_ALL	GO:0032845~negative regulation of homeostatic process	1.131541726	3.19E-05	2.39E-04
GOTERM_BP_ALL	GO:0031644~regulation of neurological system process	2.97029703	3.20E-05	2.39E-04
GOTERM_BP_ALL	GO:0055080~cation homeostasis	4.384724187	3.26E-05	2.43E-04
GOTERM_BP_ALL	GO:0043279~response to alkaloid	1.697312588	3.41E-05	2.54E-04
GOTERM_BP_ALL	GO:0044087~regulation of cellular component biogenesis	2.828854314	3.61E-05	2.68E-04
GOTERM_BP_ALL	GO:0001938~positive regulation of endothelial cell proliferation	0.99009901	3.65E-05	2.71E-04
GOTERM_BP_ALL	GO:0043370~regulation of CD4-positive, alpha beta T cell differentiation	0.99009901	3.65E-05	2.71E-04
GOTERM_BP_ALL	GO:0046777~protein amino acid autophosphorylation	2.121640736	3.82E-05	2.83E-04
GOTERM_BP_ALL	GO:0030856~regulation of epithelial cell differentiation	1.272984441	4.05E-05	2.99E-04
GOTERM_BP_ALL	GO:0018105~peptidyl-serine phosphorylation	1.272984441	4.05E-05	2.99E-04
GOTERM_BP_ALL	GO:0045930~negative regulation of mitotic cell cycle	1.131541726	4.49E-05	3.31E-04
GOTERM_BP_ALL	GO:0001658~branching involved in ureteric bud morphogenesis	1.131541726	4.49E-05	3.31E-04
GOTERM_BP_ALL	GO:0008633~activation of pro-apoptotic gene products	1.131541726	4.49E-05	3.31E-04
GOTERM_BP_ALL	GO:0022407~regulation of cell-cell adhesion	1.131541726	4.49E-05	3.31E-04
GOTERM_BP_ALL	GO:0060675~ureteric bud morphogenesis	1.131541726	4.49E-05	3.31E-04
GOTERM_BP_ALL	GO:0033138~positive regulation of peptidyl-serine phosphorylation	0.848656294	4.66E-05	3.43E-04
GOTERM_BP_ALL	GO:0045622~regulation of T-helper cell differentiation	0.848656294	4.66E-05	3.43E-04
GOTERM_BP_ALL	GO:0032870~cellular response to hormone stimulus	2.687411598	4.94E-05	3.63E-04
GOTERM_BP_ALL	GO:0045767~regulation of anti-apoptosis	1.414427157	5.04E-05	3.70E-04
GOTERM_BP_ALL	GO:0010675~regulation of cellular carbohydrate metabolic process	1.414427157	5.04E-05	3.70E-04
GOTERM_BP_ALL	GO:0050729~positive regulation of inflammatory response	1.272984441	5.29E-05	3.88E-04
GOTERM_BP_ALL	GO:0035108~limb morphogenesis	2.263083451	5.58E-05	4.08E-04
GOTERM_BP_ALL	GO:0035107~appendage morphogenesis	2.263083451	5.58E-05	4.08E-04
GOTERM_BP_ALL	GO:0043535~regulation of blood vessel endothelial cell migration	0.99009901	5.61E-05	4.09E-04
GOTERM_BP_ALL	GO:0050764~regulation of phagocytosis	0.99009901	5.61E-05	4.09E-04
GOTERM_BP_ALL	GO:0051170~nuclear import	2.121640736	5.67E-05	4.14E-04
GOTERM_BP_ALL	GO:0002695~negative regulation of leukocyte activation	1.697312588	5.78E-05	4.20E-04
GOTERM_BP_ALL	GO:0051969~regulation of transmission of nerve impulse	2.828854314	5.85E-05	4.25E-04

GOTERM_BP_ALL	GO:0035264~multicellular organism growth	1.131541726	6.19E-05	4.49E-04
GOTERM_BP_ALL	GO:0045727~positive regulation of translation	1.131541726	6.19E-05	4.49E-04
GOTERM_BP_ALL	GO:0006109~regulation of carbohydrate metabolic process	1.414427157	6.28E-05	4.54E-04
GOTERM_BP_ALL	GO:0017015~regulation of transforming growth factor beta receptor signaling pathway	1.414427157	6.28E-05	4.54E-04
GOTERM_BP_ALL	GO:0042475~odontogenesis of dentine-containing tooth	1.414427157	6.28E-05	4.54E-04
GOTERM_BP_ALL	GO:0030005~cellular di-, tri-valent inorganic cation homeostasis	3.677510608	6.64E-05	4.79E-04
GOTERM_BP_ALL	GO:0008624~induction of apoptosis by extracellular signals	2.404526167	6.63E-05	4.79E-04
GOTERM_BP_ALL	GO:0042742~defense response to bacterium	2.404526167	6.63E-05	4.79E-04
GOTERM_BP_ALL	GO:0060390~regulation of SMAD protein nuclear translocation	0.707213579	6.71E-05	4.83E-04
GOTERM_BP_ALL	GO:0033683~nucleotide-excision repair, DNA incision	0.707213579	6.71E-05	4.83E-04
GOTERM_BP_ALL	GO:0060391~positive regulation of SMAD protein nuclear translocation	0.707213579	6.71E-05	4.83E-04
GOTERM_BP_ALL	GO:0002544~chronic inflammatory response	0.707213579	6.71E-05	4.83E-04
GOTERM_BP_ALL	GO:0010225~response to UV-C	0.707213579	6.71E-05	4.83E-04
GOTERM_BP_ALL	GO:0042509~regulation of tyrosine phosphorylation of STAT protein	1.272984441	6.84E-05	4.91E-04
GOTERM_BP_ALL	GO:0051187~cofactor catabolic process	1.272984441	6.84E-05	4.91E-04
GOTERM_BP_ALL	GO:0050708~regulation of protein secretion	1.697312588	6.83E-05	4.91E-04
GOTERM_BP_ALL	GO:0055074~calcium ion homeostasis	3.253182461	7.31E-05	5.23E-04
GOTERM_BP_ALL	GO:0042593~glucose homeostasis	1.555869873	7.60E-05	5.44E-04
GOTERM_BP_ALL	GO:0033500~carbohydrate homeostasis	1.555869873	7.60E-05	5.44E-04
GOTERM_BP_ALL	GO:0001704~formation of primary germ layer	1.414427157	7.77E-05	5.54E-04
GOTERM_BP_ALL	GO:0035270~endocrine system development	1.838755304	7.85E-05	5.59E-04
GOTERM_BP_ALL	GO:0045087~innate immune response	2.687411598	8.07E-05	5.74E-04
GOTERM_BP_ALL	GO:0007005~mitochondrion organization	2.687411598	8.07E-05	5.74E-04
GOTERM_BP_ALL	GO:0045736~negative regulation of cyclin-dependent protein kinase activity	0.848656294	8.21E-05	5.83E-04
GOTERM_BP_ALL	GO:0030520~estrogen receptor signaling pathway	0.848656294	8.21E-05	5.83E-04
GOTERM_BP_ALL	GO:0002762~negative regulation of myeloid leukocyte differentiation	0.99009901	8.32E-05	5.90E-04
GOTERM_BP_ALL	GO:0035094~response to nicotine	0.99009901	8.32E-05	5.90E-04
GOTERM_BP_ALL	GO:0051896~regulation of protein kinase B signaling cascade	0.99009901	8.32E-05	5.90E-04
GOTERM_BP_ALL	GO:0007131~reciprocal meiotic recombination	1.131541726	8.39E-05	5.93E-04

GOTERM_BP_ALL	GO:0002712~regulation of B cell mediated immunity	1.131541726	8.39E-05	5.93E-04
GOTERM_BP_ALL	GO:0002889~regulation of immunoglobulin mediated immune response	1.131541726	8.39E-05	5.93E-04
GOTERM_BP_ALL	GO:0060021~palate development	1.272984441	8.75E-05	6.18E-04
GOTERM_BP_ALL	GO:0045582~positive regulation of T cell differentiation	1.272984441	8.75E-05	6.18E-04
GOTERM_BP_ALL	GO:0060173~limb development	2.263083451	8.90E-05	6.28E-04
GOTERM_BP_ALL	GO:0048736~appendage development	2.263083451	8.90E-05	6.28E-04
GOTERM_BP_ALL	GO:0001890~placenta development	1.697312588	9.44E-05	6.65E-04
GOTERM_BP_ALL	GO:0001541~ovarian follicle development	1.414427157	9.54E-05	6.71E-04
GOTERM_BP_ALL	GO:0001570~vasculogenesis	1.414427157	9.54E-05	6.71E-04
GOTERM_BP_ALL	GO:0010553~negative regulation of specific transcription from RNA polymerase II promoter	1.414427157	9.54E-05	6.71E-04
GOTERM_BP_ALL	GO:0055065~metal ion homeostasis	3.394625177	9.69E-05	6.80E-04
GOTERM_BP_ALL	GO:0030198~extracellular matrix organization	2.263083451	9.96E-05	6.97E-04
GOTERM_BP_ALL	GO:0009059~macromolecule biosynthetic process	24.46958982	1.02E-04	7.16E-04
GOTERM_BP_ALL	GO:0040014~regulation of multicellular organism growth	1.697312588	1.10E-04	7.71E-04
GOTERM_BP_ALL	GO:0010948~negative regulation of cell cycle process	1.131541726	1.12E-04	7.79E-04
GOTERM_BP_ALL	GO:0000186~activation of MAPKK activity	1.131541726	1.12E-04	7.79E-04
GOTERM_BP_ALL	GO:0019932~second-messenger-mediated signaling	3.677510608	1.16E-04	8.09E-04
GOTERM_BP_ALL	GO:0022405~hair cycle process	1.414427157	1.16E-04	8.09E-04
GOTERM_BP_ALL	GO:0001942~hair follicle development	1.414427157	1.16E-04	8.09E-04
GOTERM_BP_ALL	GO:0031647~regulation of protein stability	1.414427157	1.16E-04	8.09E-04
GOTERM_BP_ALL	GO:0007127~meiosis I	1.414427157	1.16E-04	8.09E-04
GOTERM_BP_ALL	GO:0022404~molting cycle process	1.414427157	1.16E-04	8.09E-04
GOTERM_BP_ALL	GO:0006351~transcription, DNA-dependent	4.243281471	1.17E-04	8.12E-04
GOTERM_BP_ALL	GO:0016044~membrane organization	5.091937765	1.17E-04	8.13E-04
GOTERM_BP_ALL	GO:0033993~response to lipid	0.99009901	1.20E-04	8.29E-04
GOTERM_BP_ALL	GO:0007492~endoderm development	0.99009901	1.20E-04	8.29E-04
GOTERM_BP_ALL	GO:0019882~antigen processing and presentation	1.98019802	1.22E-04	8.44E-04
GOTERM_BP_ALL	GO:0042176~regulation of protein catabolic process	1.555869873	1.29E-04	8.88E-04
GOTERM_BP_ALL	GO:0006913~nucleocytoplasmic transport	2.828854314	1.31E-04	9.03E-04
GOTERM_BP_ALL	GO:0046006~regulation of activated T cell proliferation	0.848656294	1.35E-04	9.29E-04
GOTERM_BP_ALL	GO:0010259~multicellular organismal aging	0.848656294	1.35E-04	9.29E-04

GOTERM_BP_ALL	GO:0070228~regulation of lymphocyte apoptosis	0.848656294	1.35E-04	9.29E-04
GOTERM_BP_ALL	GO:0060389~pathway-restricted SMAD protein phosphorylation	0.848656294	1.35E-04	9.29E-04
GOTERM_BP_ALL	GO:0010833~telomere maintenance via telomere lengthening	0.848656294	1.35E-04	9.29E-04
GOTERM_BP_ALL	GO:0002573~myeloid leukocyte differentiation	1.272984441	1.39E-04	9.53E-04
GOTERM_BP_ALL	GO:0042303~molting cycle	1.414427157	1.41E-04	9.66E-04
GOTERM_BP_ALL	GO:0001824~blastocyst development	1.414427157	1.41E-04	9.66E-04
GOTERM_BP_ALL	GO:0042633~hair cycle	1.414427157	1.41E-04	9.66E-04
GOTERM_BP_ALL	GO:0003002~regionalization	3.253182461	1.46E-04	9.98E-04
GOTERM_BP_ALL	GO:0031100~organ regeneration	1.131541726	1.47E-04	0.001001502
GOTERM_BP_ALL	GO:0010243~response to organic nitrogen	1.697312588	1.49E-04	0.001014904
GOTERM_BP_ALL	GO:0007249~I-kappaB kinase/NF-kappaB cascade	1.697312588	1.49E-04	0.001014904
GOTERM_BP_ALL	GO:0032774~RNA biosynthetic process	4.243281471	1.49E-04	0.001015283
GOTERM_BP_ALL	GO:0051095~regulation of helicase activity	0.707213579	1.51E-04	0.001022516
GOTERM_BP_ALL	GO:0045080~positive regulation of chemokine biosynthetic process	0.707213579	1.51E-04	0.001022516
GOTERM_BP_ALL	GO:0060558~regulation of calcidiol 1-monooxygenase activity	0.707213579	1.51E-04	0.001022516
GOTERM_BP_ALL	GO:0051169~nuclear transport	2.828854314	1.55E-04	0.001053529
GOTERM_BP_ALL	GO:0030003~cellular cation homeostasis	3.818953324	1.61E-04	0.001085766
GOTERM_BP_ALL	GO:0007098~centrosome cycle	0.99009901	1.68E-04	0.001136729
GOTERM_BP_ALL	GO:0010907~positive regulation of glucose metabolic process	0.99009901	1.68E-04	0.001136729
GOTERM_BP_ALL	GO:0051402~neuron apoptosis	0.99009901	1.68E-04	0.001136729
GOTERM_BP_ALL	GO:0031669~cellular response to nutrient levels	1.414427157	1.70E-04	0.001147798
GOTERM_BP_ALL	GO:0045621~positive regulation of lymphocyte differentiation	1.272984441	1.73E-04	0.001163774
GOTERM_BP_ALL	GO:0010906~regulation of glucose metabolic process	1.272984441	1.73E-04	0.001163774
GOTERM_BP_ALL	GO:0006606~protein import into nucleus	1.98019802	1.77E-04	0.001188131
GOTERM_BP_ALL	GO:0042391~regulation of membrane potential	2.545968883	1.78E-04	0.00119448
GOTERM_BP_ALL	GO:0042476~odontogenesis	1.555869873	1.79E-04	0.00120018
GOTERM_BP_ALL	GO:0051250~negative regulation of lymphocyte activation	1.555869873	1.79E-04	0.00120018
GOTERM_BP_ALL	GO:0000279~M phase	4.526166902	1.81E-04	0.001207905
GOTERM_BP_ALL	GO:0006935~chemotaxis	2.828854314	1.84E-04	0.001225838
GOTERM_BP_ALL	GO:0042330~taxis	2.828854314	1.84E-04	0.001225838
GOTERM_BP_ALL	GO:0002526~acute inflammatory response	2.121640736	1.87E-04	0.001245143
GOTERM_BP_ALL	GO:0007565~female pregnancy	2.263083451	1.89E-04	0.001258918
GOTERM_BP_ALL	GO:0007423~sensory organ development	3.536067893	1.99E-04	0.001320111
GOTERM_BP_ALL	GO:0046328~regulation of JNK cascade	1.697312588	1.99E-04	0.001322177
GOTERM_BP_ALL	GO:0042063~gliogenesis	1.697312588	1.99E-04	0.001322177

GOTERM_BP_ALL	GO:0006350~transcription	18.81188119	2.01E-04	0.001332582
GOTERM_BP_ALL	GO:0046890~regulation of lipid biosynthetic process	1.414427157	2.04E-04	0.001349468
GOTERM_BP_ALL	GO:0043433~negative regulation of transcription factor activity	1.414427157	2.04E-04	0.001349468
GOTERM_BP_ALL	GO:0034645~cellular macromolecule biosynthetic process	24.04526167	2.05E-04	0.001351132
GOTERM_BP_ALL	GO:0007611~learning or memory	2.263083451	2.09E-04	0.001380569
GOTERM_BP_ALL	GO:0008202~steroid metabolic process	3.253182461	2.10E-04	0.001382196
GOTERM_BP_ALL	GO:0010595~positive regulation of endothelial cell migration	0.848656294	2.11E-04	0.001389514
GOTERM_BP_ALL	GO:0045086~positive regulation of interleukin-2 biosynthetic process	0.848656294	2.11E-04	0.001389514
GOTERM_BP_ALL	GO:0045191~regulation of isotype switching	0.848656294	2.11E-04	0.001389514
GOTERM_BP_ALL	GO:0042522~regulation of tyrosine phosphorylation of Stat5 protein	0.848656294	2.11E-04	0.001389514
GOTERM_BP_ALL	GO:0050804~regulation of synaptic transmission	2.545968883	2.13E-04	0.001397506
GOTERM_BP_ALL	GO:0001707~mesoderm formation	1.272984441	2.13E-04	0.001399107
GOTERM_BP_ALL	GO:0046425~regulation of JAK-STAT cascade	1.272984441	2.13E-04	0.001399107
GOTERM_BP_ALL	GO:0030521~androgen receptor signaling pathway	1.272984441	2.13E-04	0.001399107
GOTERM_BP_ALL	GO:0010741~negative regulation of protein kinase cascade	1.272984441	2.13E-04	0.001399107
GOTERM_BP_ALL	GO:0009058~biosynthetic process	29.27864215	2.17E-04	0.001416867
GOTERM_BP_ALL	GO:0046883~regulation of hormone secretion	1.697312588	2.29E-04	0.001496536
GOTERM_BP_ALL	GO:0030890~positive regulation of B cell proliferation	0.99009901	2.31E-04	0.001505868
GOTERM_BP_ALL	GO:0051385~response to mineralocorticoid stimulus	0.99009901	2.31E-04	0.001505868
GOTERM_BP_ALL	GO:0032570~response to progesterone stimulus	0.99009901	2.31E-04	0.001505868
GOTERM_BP_ALL	GO:0040029~regulation of gene expression, epigenetic	1.838755304	2.32E-04	0.001508845
GOTERM_BP_ALL	GO:0032868~response to insulin stimulus	2.121640736	2.32E-04	0.001510327
GOTERM_BP_ALL	GO:0043062~extracellular structure organization	2.828854314	2.34E-04	0.0015189
GOTERM_BP_ALL	GO:0008156~negative regulation of DNA replication	1.131541726	2.44E-04	0.001577786
GOTERM_BP_ALL	GO:0031341~regulation of cell killing	1.131541726	2.44E-04	0.001577786
GOTERM_BP_ALL	GO:0045833~negative regulation of lipid metabolic process	1.131541726	2.44E-04	0.001577786
GOTERM_BP_ALL	GO:0046320~regulation of fatty acid oxidation	1.131541726	2.44E-04	0.001577786
GOTERM_BP_ALL	GO:0048002~antigen processing and presentation of peptide antigen	1.131541726	2.44E-04	0.001577786
GOTERM_BP_ALL	GO:0042542~response to hydrogen peroxide	1.555869873	2.45E-04	0.001584619

GOTERM_BP_ALL	GO:0001667~ameboidal cell migration	1.272984441	2.61E-04	0.001685741
GOTERM_BP_ALL	GO:0030900~forebrain development	2.687411598	2.78E-04	0.001795562
GOTERM_BP_ALL	GO:0019047~provirus integration	0.707213579	2.90E-04	0.001867906
GOTERM_BP_ALL	GO:0030069~lysogeny	0.707213579	2.90E-04	0.001867906
GOTERM_BP_ALL	GO:0045807~positive regulation of endocytosis	1.131541726	3.08E-04	0.001980635
GOTERM_BP_ALL	GO:0001569~patterning of blood vessels	0.99009901	3.11E-04	0.001993466
GOTERM_BP_ALL	GO:0043029~T cell homeostasis	0.99009901	3.11E-04	0.001993466
GOTERM_BP_ALL	GO:0032231~regulation of actin filament bundle formation	0.99009901	3.11E-04	0.001993466
GOTERM_BP_ALL	GO:0010862~positive regulation of pathway-restricted SMAD protein phosphorylation	0.848656294	3.16E-04	0.002026537
GOTERM_BP_ALL	GO:0048332~mesoderm morphogenesis	1.272984441	3.17E-04	0.002029339
GOTERM_BP_ALL	GO:0016331~morphogenesis of embryonic epithelium	1.555869873	3.30E-04	0.002107304
GOTERM_BP_ALL	GO:0045927~positive regulation of growth	1.838755304	3.35E-04	0.002135085
GOTERM_BP_ALL	GO:0051186~cofactor metabolic process	3.111739745	3.38E-04	0.002155444
GOTERM_BP_ALL	GO:0006909~phagocytosis	1.414427157	3.41E-04	0.002165429
GOTERM_BP_ALL	GO:0010721~negative regulation of cell development	1.414427157	3.41E-04	0.002165429
GOTERM_BP_ALL	GO:0070302~regulation of stress-activated protein kinase signaling pathway	1.697312588	3.43E-04	0.002174661
GOTERM_BP_ALL	GO:0044248~cellular catabolic process	10.32531825	3.46E-04	0.002191238
GOTERM_BP_ALL	GO:0006875~cellular metal ion homeostasis	3.111739745	3.63E-04	0.002296253
GOTERM_BP_ALL	GO:0050769~positive regulation of neurogenesis	1.555869873	3.81E-04	0.002406309
GOTERM_BP_ALL	GO:0018209~peptidyl-serine modification	1.272984441	3.83E-04	0.00241558
GOTERM_BP_ALL	GO:0006874~cellular calcium ion homeostasis	2.97029703	3.84E-04	0.002416366
GOTERM_BP_ALL	GO:0043467~regulation of generation of precursor metabolites and energy	1.131541726	3.86E-04	0.002426983
GOTERM_BP_ALL	GO:0045768~positive regulation of anti-apoptosis	1.131541726	3.86E-04	0.002426983
GOTERM_BP_ALL	GO:0000019~regulation of mitotic recombination	0.565770863	3.98E-04	0.002501183
GOTERM_BP_ALL	GO:0042092~T-helper 2 type immune response	0.565770863	3.98E-04	0.002501183
GOTERM_BP_ALL	GO:0048538~thymus development	0.99009901	4.10E-04	0.002572921
GOTERM_BP_ALL	GO:0000187~activation of MAPK activity	1.838755304	4.23E-04	0.002646276
GOTERM_BP_ALL	GO:0034504~protein localization in nucleus	1.98019802	4.34E-04	0.00271226
GOTERM_BP_ALL	GO:0007166~cell surface receptor linked signal transduction	16.69024045	4.37E-04	0.002725478
GOTERM_BP_ALL	GO:0007612~learning	1.555869873	4.38E-04	0.00273056
GOTERM_BP_ALL	GO:0016567~protein ubiquitination	2.263083451	4.51E-04	0.002806659

GOTERM_BP_ALL	GO:0002687~positive regulation of leukocyte migration	0.848656294	4.56E-04	0.002834689
GOTERM_BP_ALL	GO:0032760~positive regulation of tumor necrosis factor production	0.848656294	4.56E-04	0.002834689
GOTERM_BP_ALL	GO:0050778~positive regulation of immune response	2.545968883	4.58E-04	0.002844303
GOTERM_BP_ALL	GO:0050796~regulation of insulin secretion	1.272984441	4.59E-04	0.002844703
GOTERM_BP_ALL	GO:0032446~protein modification by small protein conjugation	2.404526167	4.60E-04	0.002846231
GOTERM_BP_ALL	GO:0016202~regulation of striated muscle tissue development	1.414427157	4.68E-04	0.002890374
GOTERM_BP_ALL	GO:0003013~circulatory system process	2.97029703	4.74E-04	0.002923963
GOTERM_BP_ALL	GO:0008015~blood circulation	2.97029703	4.74E-04	0.002923963
GOTERM_BP_ALL	GO:0044259~multicellular organismal macromolecule metabolic process	1.131541726	4.78E-04	0.002946328
GOTERM_BP_ALL	GO:0006084~acetyl-CoA metabolic process	1.131541726	4.78E-04	0.002946328
GOTERM_BP_ALL	GO:0050728~negative regulation of inflammatory response	1.131541726	4.78E-04	0.002946328
GOTERM_BP_ALL	GO:0007276~gamete generation	4.95049505	4.92E-04	0.003024558
GOTERM_BP_ALL	GO:0055114~oxidation reduction	7.072135785	4.97E-04	0.003049925
GOTERM_BP_ALL	GO:0052200~response to host defenses	0.707213579	5.03E-04	0.003083293
GOTERM_BP_ALL	GO:0010717~regulation of epithelial to mesenchymal transition	0.707213579	5.03E-04	0.003083293
GOTERM_BP_ALL	GO:0052173~response to defenses of other organism during symbiotic interaction	0.707213579	5.03E-04	0.003083293
GOTERM_BP_ALL	GO:0016446~somatic hypermutation of immunoglobulin genes	0.707213579	5.03E-04	0.003083293
GOTERM_BP_ALL	GO:0075136~response to host	0.707213579	5.03E-04	0.003083293
GOTERM_BP_ALL	GO:0042104~positive regulation of activated T cell proliferation	0.707213579	5.03E-04	0.003083293
GOTERM_BP_ALL	GO:0002566~somatic diversification of immune receptors via somatic mutation	0.707213579	5.03E-04	0.003083293
GOTERM_BP_ALL	GO:0035019~somatic stem cell maintenance	0.707213579	5.03E-04	0.003083293
GOTERM_BP_ALL	GO:0007626~locomotory behavior	3.818953324	5.26E-04	0.003218876
GOTERM_BP_ALL	GO:0042531~positive regulation of tyrosine phosphorylation of STAT protein	0.99009901	5.33E-04	0.003259589
GOTERM_BP_ALL	GO:0043271~negative regulation of ion transport	0.99009901	5.33E-04	0.003259589
GOTERM_BP_ALL	GO:0006099~tricarboxylic acid cycle	0.99009901	5.33E-04	0.003259589
GOTERM_BP_ALL	GO:0046356~acetyl-CoA catabolic process	0.99009901	5.33E-04	0.003259589
GOTERM_BP_ALL	GO:0032989~cellular component morphogenesis	4.95049505	5.35E-04	0.003267138
GOTERM_BP_ALL	GO:0048634~regulation of muscle development	1.414427157	5.45E-04	0.003321007
GOTERM_BP_ALL	GO:0050679~positive regulation of epithelial cell proliferation	1.272984441	5.47E-04	0.003330831

GOTERM_BP_ALL	GO:0044249~cellular biosynthetic process	28.14710042	5.67E-04	0.003448653
GOTERM_BP_ALL	GO:0009615~response to virus	2.121640736	5.71E-04	0.003465088
GOTERM_BP_ALL	GO:0006970~response to osmotic stress	1.131541726	5.88E-04	0.003561476
GOTERM_BP_ALL	GO:0051297~centrosome organization	1.131541726	5.88E-04	0.003561476
GOTERM_BP_ALL	GO:0051291~protein heterooligomerization	1.414427157	6.32E-04	0.003822866
GOTERM_BP_ALL	GO:0006725~cellular aromatic compound metabolic process	2.404526167	6.42E-04	0.003877645
GOTERM_BP_ALL	GO:0051493~regulation of cytoskeleton organization	2.404526167	6.42E-04	0.003877645
GOTERM_BP_ALL	GO:0046651~lymphocyte proliferation	1.272984441	6.49E-04	0.003912514
GOTERM_BP_ALL	GO:0033189~response to vitamin A	1.272984441	6.49E-04	0.003912514
GOTERM_BP_ALL	GO:0048666~neuron development	4.384724187	6.58E-04	0.00396501
GOTERM_BP_ALL	GO:0006366~transcription from RNA polymerase II promoter	3.394625177	6.67E-04	0.004012258
GOTERM_BP_ALL	GO:0031343~positive regulation of cell killing	0.99009901	6.83E-04	0.00410148
GOTERM_BP_ALL	GO:0030855~epithelial cell differentiation	2.404526167	6.96E-04	0.004173349
GOTERM_BP_ALL	GO:0000902~cell morphogenesis	4.526166902	7.05E-04	0.004219577
GOTERM_BP_ALL	GO:0043269~regulation of ion transport	1.98019802	7.19E-04	0.004298243
GOTERM_BP_ALL	GO:0030326~embryonic limb morphogenesis	1.838755304	7.31E-04	0.004356811
GOTERM_BP_ALL	GO:0035113~embryonic appendage morphogenesis	1.838755304	7.31E-04	0.004356811
GOTERM_BP_ALL	GO:0045444~fat cell differentiation	1.414427157	7.30E-04	0.004358378
GOTERM_BP_ALL	GO:0031668~cellular response to extracellular stimulus	1.555869873	7.43E-04	0.004425933
GOTERM_BP_ALL	GO:0034599~cellular response to oxidative stress	1.272984441	7.65E-04	0.004545031
GOTERM_BP_ALL	GO:0051899~membrane depolarization	1.272984441	7.65E-04	0.004545031
GOTERM_BP_ALL	GO:0001838~embryonic epithelial tube formation	1.272984441	7.65E-04	0.004545031
GOTERM_BP_ALL	GO:0050930~induction of positive chemotaxis	0.707213579	8.07E-04	0.004789696
GOTERM_BP_ALL	GO:0050829~defense response to Gram-negative bacterium	0.707213579	8.07E-04	0.004789696
GOTERM_BP_ALL	GO:0042088~T-helper 1 type immune response	0.707213579	8.07E-04	0.004789696
GOTERM_BP_ALL	GO:0007292~female gamete generation	1.555869873	8.42E-04	0.004992343
GOTERM_BP_ALL	GO:0051785~positive regulation of nuclear division	0.99009901	8.63E-04	0.005108779
GOTERM_BP_ALL	GO:0045862~positive regulation of proteolysis	0.99009901	8.63E-04	0.005108779
GOTERM_BP_ALL	GO:0010743~regulation of foam cell differentiation	0.99009901	8.63E-04	0.005108779
GOTERM_BP_ALL	GO:0045669~positive regulation of osteoblast differentiation	0.99009901	8.63E-04	0.005108779
GOTERM_BP_ALL	GO:0045840~positive regulation of mitosis	0.99009901	8.63E-04	0.005108779

GOTERM_BP_ALL	GO:0010594~regulation of endothelial cell migration	0.99009901	8.63E-04	0.005108779
GOTERM_BP_ALL	GO:0001910~regulation of leukocyte mediated cytotoxicity	0.99009901	8.63E-04	0.005108779
GOTERM_BP_ALL	GO:0010827~regulation of glucose transport	1.131541726	8.66E-04	0.00511624
GOTERM_BP_ALL	GO:0031575~G1/S transition checkpoint	0.848656294	8.69E-04	0.005128277
GOTERM_BP_ALL	GO:0043200~response to amino acid stimulus	0.848656294	8.69E-04	0.005128277
GOTERM_BP_ALL	GO:0043409~negative regulation of MAPKKK cascade	0.848656294	8.69E-04	0.005128277
GOTERM_BP_ALL	GO:0032655~regulation of interleukin-12 production	0.848656294	8.69E-04	0.005128277
GOTERM_BP_ALL	GO:0002053~positive regulation of mesenchymal cell proliferation	0.848656294	8.69E-04	0.005128277
GOTERM_BP_ALL	GO:0046330~positive regulation of JNK cascade	0.848656294	8.69E-04	0.005128277
GOTERM_BP_ALL	GO:0002474~antigen processing and presentation of peptide antigen via MHC class I	0.848656294	8.69E-04	0.005128277
GOTERM_BP_ALL	GO:0035148~tube lumen formation	1.272984441	8.97E-04	0.005283259
GOTERM_BP_ALL	GO:0044267~cellular protein metabolic process	20.08486563	9.51E-04	0.005596058
GOTERM_BP_ALL	GO:0002328~pro-B cell differentiation	0.565770863	9.61E-04	0.00564332
GOTERM_BP_ALL	GO:0010623~developmental programmed cell death	0.565770863	9.61E-04	0.00564332
GOTERM_BP_ALL	GO:0032202~telomere assembly	0.565770863	9.61E-04	0.00564332
GOTERM_BP_ALL	GO:0002326~B cell lineage commitment	0.565770863	9.61E-04	0.00564332
GOTERM_BP_ALL	GO:0051096~positive regulation of helicase activity	0.565770863	9.61E-04	0.00564332
GOTERM_BP_ALL	GO:0002360~T cell lineage commitment	0.565770863	9.61E-04	0.00564332
GOTERM_BP_ALL	GO:0034391~regulation of smooth muscle cell apoptosis	0.565770863	9.61E-04	0.00564332
GOTERM_BP_ALL	GO:0002347~response to tumor cell	0.565770863	9.61E-04	0.00564332
GOTERM_BP_ALL	GO:0045005~maintenance of fidelity during DNA-dependent DNA replication	0.565770863	9.61E-04	0.00564332
GOTERM_BP_ALL	GO:0002246~healing during inflammatory response	0.565770863	9.61E-04	0.00564332
GOTERM_BP_ALL	GO:0051048~negative regulation of secretion	1.414427157	9.63E-04	0.005651983
GOTERM_BP_ALL	GO:0031023~microtubule organizing center organization	1.131541726	0.001039029	0.00608558
GOTERM_BP_ALL	GO:0006800~oxygen and reactive oxygen species metabolic process	1.555869873	0.001072795	0.006274022
GOTERM_BP_ALL	GO:0048066~pigmentation during development	0.99009901	0.001078246	0.006296988
GOTERM_BP_ALL	GO:0009109~coenzyme catabolic process	0.99009901	0.001078246	0.006296988
GOTERM_BP_ALL	GO:0046427~positive regulation of JAK-STAT cascade	0.99009901	0.001078246	0.006296988
GOTERM_BP_ALL	GO:0035137~hindlimb morphogenesis	0.99009901	0.001078246	0.006296988

GOTERM_BP_ALL	GO:0048146~positive regulation of fibroblast proliferation	0.99009901	0.001078246	0.006296988
GOTERM_BP_ALL	GO:0045766~positive regulation of angiogenesis	0.99009901	0.001078246	0.006296988
GOTERM_BP_ALL	GO:0035303~regulation of dephosphorylation	0.99009901	0.001078246	0.006296988
GOTERM_BP_ALL	GO:0030330~DNA damage response, signal transduction by p53 class mediator	0.99009901	0.001078246	0.006296988
GOTERM_BP_ALL	GO:0046635~positive regulation of alpha-beta T cell activation	0.99009901	0.001078246	0.006296988
GOTERM_BP_ALL	GO:0001837~epithelial to mesenchymal transition	0.848656294	0.001157216	0.006747436
GOTERM_BP_ALL	GO:0010464~regulation of mesenchymal cell proliferation	0.848656294	0.001157216	0.006747436
GOTERM_BP_ALL	GO:0002285~lymphocyte activation during immune response	0.848656294	0.001157216	0.006747436
GOTERM_BP_ALL	GO:0018107~peptidyl-threonine phosphorylation	0.848656294	0.001157216	0.006747436
GOTERM_BP_ALL	GO:0030501~positive regulation of bone mineralization	0.848656294	0.001157216	0.006747436
GOTERM_BP_ALL	GO:0050869~negative regulation of B cell activation	0.848656294	0.001157216	0.006747436
GOTERM_BP_ALL	GO:0002763~positive regulation of myeloid leukocyte differentiation	0.848656294	0.001157216	0.006747436
GOTERM_BP_ALL	GO:0046631~alpha-beta T cell activation	0.848656294	0.001157216	0.006747436
GOTERM_BP_ALL	GO:0045646~regulation of erythrocyte differentiation	0.848656294	0.001157216	0.006747436
GOTERM_BP_ALL	GO:0000904~cell morphogenesis involved in differentiation	3.394625177	0.001192564	0.006943241
GOTERM_BP_ALL	GO:0021915~neural tube development	1.555869873	0.001205965	0.007011253
GOTERM_BP_ALL	GO:0030030~cell projection organization	4.526166902	0.001212204	0.007037613
GOTERM_BP_ALL	GO:0002274~myeloid leukocyte activation	1.272984441	0.001215507	0.007046926
GOTERM_BP_ALL	GO:0001816~cytokine production	1.272984441	0.001215507	0.007046926
GOTERM_BP_ALL	GO:0030111~regulation of Wnt receptor signaling pathway	1.272984441	0.001215507	0.007046926
GOTERM_BP_ALL	GO:0002791~regulation of peptide secretion	1.272984441	0.001215507	0.007046926
GOTERM_BP_ALL	GO:0032729~positive regulation of interferon-gamma production	0.707213579	0.001221185	0.007069917
GOTERM_BP_ALL	GO:0048010~vascular endothelial growth factor receptor signaling pathway	0.707213579	0.001221185	0.007069917
GOTERM_BP_ALL	GO:0007004~telomere maintenance via telomerase	0.707213579	0.001221185	0.007069917
GOTERM_BP_ALL	GO:0048070~regulation of pigmentation during development	0.707213579	0.001221185	0.007069917
GOTERM_BP_ALL	GO:0051897~positive regulation of protein kinase B signaling cascade	0.707213579	0.001221185	0.007069917
GOTERM_BP_ALL	GO:0031348~negative regulation of defense response	1.131541726	0.001238359	0.007159116
GOTERM_BP_ALL	GO:0042098~T cell proliferation	0.99009901	0.001331953	0.007687813
GOTERM_BP_ALL	GO:0001654~eye development	2.263083451	0.001338439	0.007714456

GOTERM_BP_ALL	GO:0051302~regulation of cell division	1.272984441	0.00140566	0.008089426
GOTERM_BP_ALL	GO:0007010~cytoskeleton organization	5.091937765	0.001409207	0.008098599
GOTERM_BP_ALL	GO:0044236~multicellular organismal metabolic process	1.131541726	0.001466411	0.008414612
GOTERM_BP_ALL	GO:0009895~negative regulation of catabolic process	1.131541726	0.001466411	0.008414612
GOTERM_BP_ALL	GO:0043010~camera-type eye development	1.98019802	0.001494509	0.008563493
GOTERM_BP_ALL	GO:0046626~regulation of insulin receptor signaling pathway	0.848656294	0.001510395	0.008642323
GOTERM_BP_ALL	GO:0002758~innate immune response-activating signal transduction	0.848656294	0.001510395	0.008642323
GOTERM_BP_ALL	GO:0048008~platelet-derived growth factor receptor signaling pathway	0.848656294	0.001510395	0.008642323
GOTERM_BP_ALL	GO:0045076~regulation of interleukin-2 biosynthetic process	0.848656294	0.001510395	0.008642323
GOTERM_BP_ALL	GO:0002218~activation of innate immune response	0.848656294	0.001510395	0.008642323
GOTERM_BP_ALL	GO:0070169~positive regulation of biomineral formation	0.848656294	0.001510395	0.008642323
GOTERM_BP_ALL	GO:0051492~regulation of stress fiber formation	0.848656294	0.001510395	0.008642323
GOTERM_BP_ALL	GO:0051301~cell division	3.818953324	0.001554721	0.008882838
GOTERM_BP_ALL	GO:0043473~pigmentation	1.414427157	0.001611628	0.009194146
GOTERM_BP_ALL	GO:0045793~positive regulation of cell size	1.272984441	0.001618686	0.009221649
GOTERM_BP_ALL	GO:0002260~lymphocyte homeostasis	0.99009901	0.001628801	0.009266397
GOTERM_BP_ALL	GO:0007173~epidermal growth factor receptor signaling pathway	0.99009901	0.001628801	0.009266397
GOTERM_BP_ALL	GO:0031577~spindle checkpoint	0.707213579	0.001764111	0.010019339
GOTERM_BP_ALL	GO:0051926~negative regulation of calcium ion transport	0.707213579	0.001764111	0.010019339
GOTERM_BP_ALL	GO:0045885~positive regulation of survival gene product expression	0.707213579	0.001764111	0.010019339
GOTERM_BP_ALL	GO:0032204~regulation of telomere maintenance	0.707213579	0.001764111	0.010019339
GOTERM_BP_ALL	GO:0009056~catabolic process	11.59830269	0.001830597	0.010381197
GOTERM_BP_ALL	GO:0034101~erythrocyte homeostasis	1.272984441	0.001856472	0.010498755
GOTERM_BP_ALL	GO:0014911~positive regulation of smooth muscle cell migration	0.565770863	0.001854192	0.010500149
GOTERM_BP_ALL	GO:0006283~transcription-coupled nucleotide-excision repair	0.565770863	0.001854192	0.010500149
GOTERM_BP_ALL	GO:0033483~gas homeostasis	0.565770863	0.001854192	0.010500149
GOTERM_BP_ALL	GO:0046638~positive regulation of alpha-beta T cell differentiation	0.848656294	0.001936874	0.010936568
GOTERM_BP_ALL	GO:0015074~DNA integration	0.848656294	0.001936874	0.010936568
GOTERM_BP_ALL	GO:0031330~negative regulation of cellular catabolic process	0.848656294	0.001936874	0.010936568
GOTERM_BP_ALL	GO:0002831~regulation of response to biotic stimulus	0.99009901	0.001973328	0.011126403

GOTERM_BP_ALL	GO:0033013~tetrapyrrole metabolic process	0.99009901	0.001973328	0.011126403
GOTERM_BP_ALL	GO:0006778~porphyrin metabolic process	0.99009901	0.001973328	0.011126403
GOTERM_BP_ALL	GO:0014075~response to amine stimulus	1.131541726	0.002019727	0.011371426
GOTERM_BP_ALL	GO:0051781~positive regulation of cell division	1.131541726	0.002019727	0.011371426
GOTERM_BP_ALL	GO:0006968~cellular defense response	1.414427157	0.002046586	0.011506407
GOTERM_BP_ALL	GO:0009791~post-embryonic development	1.555869873	0.002089059	0.011728242
GOTERM_BP_ALL	GO:0031175~neuron projection development	3.394625177	0.002214228	0.012410656
GOTERM_BP_ALL	GO:0001841~neural tube formation	1.131541726	0.002350772	0.013154157
GOTERM_BP_ALL	GO:0016569~covalent chromatin modification	2.121640736	0.002364002	0.013210073
GOTERM_BP_ALL	GO:0030500~regulation of bone mineralization	0.99009901	0.002370215	0.013226895
GOTERM_BP_ALL	GO:0032663~regulation of interleukin-2 production	0.99009901	0.002370215	0.013226895
GOTERM_BP_ALL	GO:0032368~regulation of lipid transport	0.99009901	0.002370215	0.013226895
GOTERM_BP_ALL	GO:0060606~tube closure	0.99009901	0.002370215	0.013226895
GOTERM_BP_ALL	GO:0042990~regulation of transcription factor import into nucleus	0.99009901	0.002370215	0.013226895
GOTERM_BP_ALL	GO:0001843~neural tube closure	0.99009901	0.002370215	0.013226895
GOTERM_BP_ALL	GO:0046849~bone remodeling	0.848656294	0.002445088	0.013624152
GOTERM_BP_ALL	GO:0070304~positive regulation of stress-activated protein kinase signaling pathway	0.848656294	0.002445088	0.013624152
GOTERM_BP_ALL	GO:0007140~male meiosis	0.848656294	0.002445088	0.013624152
GOTERM_BP_ALL	GO:0046889~positive regulation of lipid biosynthetic process	0.848656294	0.002445088	0.013624152
GOTERM_BP_ALL	GO:0043255~regulation of carbohydrate biosynthetic process	0.848656294	0.002445088	0.013624152
GOTERM_BP_ALL	GO:0001912~positive regulation of leukocyte mediated cytotoxicity	0.848656294	0.002445088	0.013624152
GOTERM_BP_ALL	GO:0000086~G2/M transition of mitotic cell cycle	0.848656294	0.002445088	0.013624152
GOTERM_BP_ALL	GO:0018210~peptidyl-threonine modification	0.848656294	0.002445088	0.013624152
GOTERM_BP_ALL	GO:0031572~G2/M transition DNA damage checkpoint	0.707213579	0.00245419	0.013656355
GOTERM_BP_ALL	GO:0031056~regulation of histone modification	0.707213579	0.00245419	0.013656355
GOTERM_BP_ALL	GO:0014066~regulation of phosphoinositide 3-kinase cascade	0.707213579	0.00245419	0.013656355
GOTERM_BP_ALL	GO:0034612~response to tumor necrosis factor	0.707213579	0.00245419	0.013656355
GOTERM_BP_ALL	GO:0002286~T cell activation during immune response	0.707213579	0.00245419	0.013656355
GOTERM_BP_ALL	GO:0051043~regulation of membrane protein ectodomain proteolysis	0.707213579	0.00245419	0.013656355
GOTERM_BP_ALL	GO:0006790~sulfur metabolic process	1.98019802	0.002864664	0.01590424

GOTERM_BP_ALL	GO:0001764~neuron migration	1.414427157	0.0028693	0.015908652
GOTERM_BP_ALL	GO:0021766~hippocampus development	0.848656294	0.003043574	0.016845809
GOTERM_BP_ALL	GO:0010001~glial cell differentiation	1.272984441	0.003095291	0.017107357
GOTERM_BP_ALL	GO:0010769~regulation of cell morphogenesis involved in differentiation	1.555869873	0.003119308	0.017216366
GOTERM_BP_ALL	GO:0010922~positive regulation of phosphatase activity	0.565770863	0.003131842	0.017262234
GOTERM_BP_ALL	GO:0046825~regulation of protein export from nucleus	0.565770863	0.003131842	0.017262234
GOTERM_BP_ALL	GO:0030949~positive regulation of vascular endothelial growth factor receptor signaling pathway	0.565770863	0.003131842	0.017262234
GOTERM_BP_ALL	GO:0045821~positive regulation of glycolysis	0.565770863	0.003131842	0.017262234
GOTERM_BP_ALL	GO:0031058~positive regulation of histone modification	0.565770863	0.003131842	0.017262234
GOTERM_BP_ALL	GO:0010149~senescence	0.565770863	0.003131842	0.017262234
GOTERM_BP_ALL	GO:0050868~negative regulation of T cell activation	1.131541726	0.003136782	0.017266479
GOTERM_BP_ALL	GO:0044403~symbiosis, encompassing mutualism through parasitism	1.131541726	0.003136782	0.017266479
GOTERM_BP_ALL	GO:0019953~sexual reproduction	5.091937765	0.003162954	0.017386588
GOTERM_BP_ALL	GO:0046632~alpha-beta T cell differentiation	0.707213579	0.003309372	0.018161508
GOTERM_BP_ALL	GO:0032945~negative regulation of mononuclear cell proliferation	0.99009901	0.003340383	0.018306347
GOTERM_BP_ALL	GO:0070167~regulation of biomineral formation	0.99009901	0.003340383	0.018306347
GOTERM_BP_ALL	GO:0050672~negative regulation of lymphocyte proliferation	0.99009901	0.003340383	0.018306347
GOTERM_BP_ALL	GO:0021761~limbic system development	0.99009901	0.003340383	0.018306347
GOTERM_BP_ALL	GO:0070664~negative regulation of leukocyte proliferation	0.99009901	0.003340383	0.018306347
GOTERM_BP_ALL	GO:0022604~regulation of cell morphogenesis	2.121640736	0.003389611	0.018549725
GOTERM_BP_ALL	GO:0017038~protein import	2.121640736	0.003389611	0.018549725
GOTERM_BP_ALL	GO:0070647~protein modification by small protein conjugation or removal	2.404526167	0.003532499	0.01930017
GOTERM_BP_ALL	GO:0030218~erythrocyte differentiation	1.131541726	0.003598042	0.019629698
GOTERM_BP_ALL	GO:0001755~neural crest cell migration	0.848656294	0.003740906	0.020375951
GOTERM_BP_ALL	GO:0009410~response to xenobiotic stimulus	0.848656294	0.003740906	0.020375951
GOTERM_BP_ALL	GO:0001974~blood vessel remodeling	0.848656294	0.003740906	0.020375951
GOTERM_BP_ALL	GO:0050715~positive regulation of cytokine secretion	0.848656294	0.003740906	0.020375951
GOTERM_BP_ALL	GO:0014020~primary neural tube formation	0.99009901	0.003923541	0.02133416
GOTERM_BP_ALL	GO:0046324~regulation of glucose import	0.99009901	0.003923541	0.02133416
GOTERM_BP_ALL	GO:0032869~cellular response to insulin stimulus	1.414427157	0.004349578	0.023536344

GOTERM_BP_ALL	GO:0009057~macromolecule catabolic process	7.637906648	0.004349206	0.023564871
GOTERM_BP_ALL	GO:0042771~DNA damage response, signal transduction by p53 class mediator resulting in induction of apoptosis	0.707213579	0.004346924	0.023583206
GOTERM_BP_ALL	GO:0042116~macrophage activation	0.707213579	0.004346924	0.023583206
GOTERM_BP_ALL	GO:0032677~regulation of interleukin-8 production	0.707213579	0.004346924	0.023583206
GOTERM_BP_ALL	GO:0032369~negative regulation of lipid transport	0.707213579	0.004346924	0.023583206
GOTERM_BP_ALL	GO:0006338~chromatin remodeling	1.272984441	0.004386452	0.023703226
GOTERM_BP_ALL	GO:0045778~positive regulation of ossification	0.848656294	0.004545633	0.024522989
GOTERM_BP_ALL	GO:0002708~positive regulation of lymphocyte mediated immunity	0.99009901	0.004578772	0.024668106
GOTERM_BP_ALL	GO:0002705~positive regulation of leukocyte mediated immunity	0.99009901	0.004578772	0.024668106
GOTERM_BP_ALL	GO:0050768~negative regulation of neurogenesis	1.131541726	0.004673294	0.025139659
GOTERM_BP_ALL	GO:0043414~biopolymer methylation	1.414427157	0.004799167	0.025776411
GOTERM_BP_ALL	GO:0032205~negative regulation of telomere maintenance	0.565770863	0.004836877	0.025943446
GOTERM_BP_ALL	GO:0051972~regulation of telomerase activity	0.565770863	0.004836877	0.025943446
GOTERM_BP_ALL	GO:0006266~DNA ligation	0.565770863	0.004836877	0.025943446
GOTERM_BP_ALL	GO:0010660~regulation of muscle cell apoptosis	0.565770863	0.004836877	0.025943446
GOTERM_BP_ALL	GO:0030857~negative regulation of epithelial cell differentiation	0.565770863	0.004836877	0.025943446
GOTERM_BP_ALL	GO:0048147~negative regulation of fibroblast proliferation	0.565770863	0.004836877	0.025943446
GOTERM_BP_ALL	GO:0045843~negative regulation of striated muscle development	0.565770863	0.004836877	0.025943446
GOTERM_BP_ALL	GO:0002281~macrophage activation during immune response	0.565770863	0.004836877	0.025943446
GOTERM_BP_ALL	GO:0031670~cellular response to nutrient	0.565770863	0.004836877	0.025943446
GOTERM_BP_ALL	GO:0051260~protein homooligomerization	1.697312588	0.004883379	0.02615661
GOTERM_BP_ALL	GO:0010817~regulation of hormone levels	2.263083451	0.004949351	0.026472193
GOTERM_BP_ALL	GO:0006417~regulation of translation	2.121640736	0.005072561	0.027089179
GOTERM_BP_ALL	GO:0016568~chromatin modification	3.394625177	0.005146494	0.027444484
GOTERM_BP_ALL	GO:0021543~pallium development	1.131541726	0.005293923	0.028185588
GOTERM_BP_ALL	GO:0009060~aerobic respiration	0.99009901	0.005311133	0.028240183
GOTERM_BP_ALL	GO:0003018~vascular process in circulatory system	1.272984441	0.005453693	0.02895234
GOTERM_BP_ALL	GO:0040018~positive regulation of multicellular organism growth	0.848656294	0.005466214	0.028981221
GOTERM_BP_ALL	GO:0043331~response to dsRNA	0.848656294	0.005466214	0.028981221
GOTERM_BP_ALL	GO:0048730~epidermis morphogenesis	0.848656294	0.005466214	0.028981221

GOTERM_BP_ALL	GO:0042743~hydrogen peroxide metabolic process	0.848656294	0.005466214	0.028981221
GOTERM_BP_ALL	GO:0033280~response to vitamin D	0.707213579	0.005583252	0.029556711
GOTERM_BP_ALL	GO:0045954~positive regulation of natural killer cell mediated cytotoxicity	0.707213579	0.005583252	0.029556711
GOTERM_BP_ALL	GO:0050999~regulation of nitric-oxide synthase activity	0.707213579	0.005583252	0.029556711
GOTERM_BP_ALL	GO:0051412~response to corticosterone stimulus	0.707213579	0.005583252	0.029556711
GOTERM_BP_ALL	GO:0002717~positive regulation of natural killer cell mediated immunity	0.707213579	0.005583252	0.029556711
GOTERM_BP_ALL	GO:0019079~viral genome replication	0.707213579	0.005583252	0.029556711
GOTERM_BP_ALL	GO:0031576~G2/M transition checkpoint	0.707213579	0.005583252	0.029556711
GOTERM_BP_ALL	GO:0017144~drug metabolic process	0.707213579	0.005583252	0.029556711
GOTERM_BP_ALL	GO:0042089~cytokine biosynthetic process	0.707213579	0.005583252	0.029556711
GOTERM_BP_ALL	GO:0007090~regulation of S phase of mitotic cell cycle	0.707213579	0.005583252	0.029556711
GOTERM_BP_ALL	GO:0022612~gland morphogenesis	0.707213579	0.005583252	0.029556711
GOTERM_BP_ALL	GO:0046579~positive regulation of Ras protein signal transduction	0.707213579	0.005583252	0.029556711
GOTERM_BP_ALL	GO:0043123~positive regulation of I-kappaB kinase/NF-kappaB cascade	1.697312588	0.005719328	0.030229835
GOTERM_BP_ALL	GO:0045333~cellular respiration	1.697312588	0.005719328	0.030229835
GOTERM_BP_ALL	GO:0060537~muscle tissue development	1.98019802	0.005866196	0.030957095
GOTERM_BP_ALL	GO:0034284~response to monosaccharide stimulus	1.131541726	0.005974369	0.031480823
GOTERM_BP_ALL	GO:0009746~response to hexose stimulus	1.131541726	0.005974369	0.031480823
GOTERM_BP_ALL	GO:0043407~negative regulation of MAP kinase activity	0.99009901	0.006125687	0.032226988
GOTERM_BP_ALL	GO:0048565~gut development	0.99009901	0.006125687	0.032226988
GOTERM_BP_ALL	GO:0055072~iron ion homeostasis	0.99009901	0.006125687	0.032226988
GOTERM_BP_ALL	GO:0009952~anterior/posterior pattern formation	2.121640736	0.006136776	0.032243988
GOTERM_BP_ALL	GO:0002318~myeloid progenitor cell differentiation	0.424328147	0.006402131	0.033576782
GOTERM_BP_ALL	GO:0033599~regulation of mammary gland epithelial cell proliferation	0.424328147	0.006402131	0.033576782
GOTERM_BP_ALL	GO:0070587~regulation of cell-cell adhesion involved in gastrulation	0.424328147	0.006402131	0.033576782
GOTERM_BP_ALL	GO:0034114~regulation of heterotypic cell-cell adhesion	0.424328147	0.006402131	0.033576782
GOTERM_BP_ALL	GO:0006295~nucleotide-excision repair, DNA incision, 3'-to lesion	0.424328147	0.006402131	0.033576782
GOTERM_BP_ALL	GO:0060218~hemopoietic stem cell differentiation	0.424328147	0.006402131	0.033576782
GOTERM_BP_ALL	GO:0050765~negative regulation of phagocytosis	0.424328147	0.006402131	0.033576782
GOTERM_BP_ALL	GO:0032203~telomere formation via telomerase	0.424328147	0.006402131	0.033576782

GOTERM_BP_ALL	GO:0042789~mRNA transcription from RNA polymerase II promoter	0.424328147	0.006402131	0.033576782
GOTERM_BP_ALL	GO:0043570~maintenance of DNA repeat elements	0.424328147	0.006402131	0.033576782
GOTERM_BP_ALL	GO:0070141~response to UV-A	0.424328147	0.006402131	0.033576782
GOTERM_BP_ALL	GO:0035234~germ cell programmed cell death	0.424328147	0.006402131	0.033576782
GOTERM_BP_ALL	GO:0034393~positive regulation of smooth muscle cell apoptosis	0.424328147	0.006402131	0.033576782
GOTERM_BP_ALL	GO:0010661~positive regulation of muscle cell apoptosis	0.424328147	0.006402131	0.033576782
GOTERM_BP_ALL	GO:0060559~positive regulation of calcidiol 1-monooxygenase activity	0.424328147	0.006402131	0.033576782
GOTERM_BP_ALL	GO:0006925~inflammatory cell apoptosis	0.424328147	0.006402131	0.033576782
GOTERM_BP_ALL	GO:0010470~regulation of gastrulation	0.424328147	0.006402131	0.033576782
GOTERM_BP_ALL	GO:0046628~positive regulation of insulin receptor signaling pathway	0.424328147	0.006402131	0.033576782
GOTERM_BP_ALL	GO:0050994~regulation of lipid catabolic process	0.848656294	0.006510969	0.034096838
GOTERM_BP_ALL	GO:0044243~multicellular organismal catabolic process	0.848656294	0.006510969	0.034096838
GOTERM_BP_ALL	GO:0009953~dorsal/ventral pattern formation	1.272984441	0.006708313	0.035071479
GOTERM_BP_ALL	GO:0010574~regulation of vascular endothelial growth factor production	0.565770863	0.007003905	0.036547941
GOTERM_BP_ALL	GO:0033003~regulation of mast cell activation	0.565770863	0.007003905	0.036547941
GOTERM_BP_ALL	GO:0043491~protein kinase B signaling cascade	0.565770863	0.007003905	0.036547941
GOTERM_BP_ALL	GO:0045911~positive regulation of DNA recombination	0.565770863	0.007003905	0.036547941
GOTERM_BP_ALL	GO:0002262~myeloid cell homeostasis	0.565770863	0.007003905	0.036547941
GOTERM_BP_ALL	GO:0051000~positive regulation of nitric-oxide synthase activity	0.565770863	0.007003905	0.036547941
GOTERM_BP_ALL	GO:0048635~negative regulation of muscle development	0.565770863	0.007003905	0.036547941
GOTERM_BP_ALL	GO:0000731~DNA synthesis during DNA repair	0.565770863	0.007003905	0.036547941
GOTERM_BP_ALL	GO:0022408~negative regulation of cell-cell adhesion	0.565770863	0.007003905	0.036547941
GOTERM_BP_ALL	GO:0035162~embryonic hemopoiesis	0.565770863	0.007003905	0.036547941
GOTERM_BP_ALL	GO:0034405~response to fluid shear stress	0.565770863	0.007003905	0.036547941
GOTERM_BP_ALL	GO:0045414~regulation of interleukin-8 biosynthetic process	0.565770863	0.007003905	0.036547941
GOTERM_BP_ALL	GO:0045671~negative regulation of osteoclast differentiation	0.565770863	0.007003905	0.036547941
GOTERM_BP_ALL	GO:0045830~positive regulation of isotype switching	0.565770863	0.007003905	0.036547941
GOTERM_BP_ALL	GO:0032673~regulation of interleukin-4 production	0.565770863	0.007003905	0.036547941

GOTERM_BP_ALL	GO:0030947~regulation of vascular endothelial growth factor receptor signaling pathway	0.565770863	0.007003905	0.036547941
GOTERM_BP_ALL	GO:0048873~homeostasis of number of cells within a tissue	0.707213579	0.007033769	0.036610111
GOTERM_BP_ALL	GO:0042269~regulation of natural killer cell mediated cytotoxicity	0.707213579	0.007033769	0.036610111
GOTERM_BP_ALL	GO:0042107~cytokine metabolic process	0.707213579	0.007033769	0.036610111
GOTERM_BP_ALL	GO:0006278~RNA-dependent DNA replication	0.707213579	0.007033769	0.036610111
GOTERM_BP_ALL	GO:0002715~regulation of natural killer cell mediated immunity	0.707213579	0.007033769	0.036610111
GOTERM_BP_ALL	GO:0002221~pattern recognition receptor signaling pathway	0.707213579	0.007033769	0.036610111
GOTERM_BP_ALL	GO:0032732~positive regulation of interleukin-1 production	0.707213579	0.007033769	0.036610111
GOTERM_BP_ALL	GO:0051057~positive regulation of small GTPase mediated signal transduction	0.707213579	0.007033769	0.036610111
GOTERM_BP_ALL	GO:0021536~diencephalon development	0.99009901	0.007027483	0.036623443
GOTERM_BP_ALL	GO:0048568~embryonic organ development	2.404526167	0.007090474	0.036854937
GOTERM_BP_ALL	GO:0008217~regulation of blood pressure	1.697312588	0.007180084	0.037267283
GOTERM_BP_ALL	GO:0031396~regulation of protein ubiquitination	1.697312588	0.007180084	0.037267283
GOTERM_BP_ALL	GO:0032886~regulation of microtubule-based process	1.131541726	0.00752828	0.038997194
GOTERM_BP_ALL	GO:0008542~visual learning	0.848656294	0.007688018	0.039761901
GOTERM_BP_ALL	GO:0042130~negative regulation of T cell proliferation	0.848656294	0.007688018	0.039761901
GOTERM_BP_ALL	GO:0015980~energy derivation by oxidation of organic compounds	2.121640736	0.00782617	0.0404146
GOTERM_BP_ALL	GO:0006940~regulation of smooth muscle contraction	0.99009901	0.008021526	0.041355242
GOTERM_BP_ALL	GO:0051701~interaction with host	0.99009901	0.008021526	0.041355242
GOTERM_BP_ALL	GO:0048015~phosphoinositide-mediated signaling	1.555869873	0.00811044	0.041754352
GOTERM_BP_ALL	GO:0030593~neutrophil chemotaxis	0.707213579	0.008712784	0.044743231
GOTERM_BP_ALL	GO:0002718~regulation of cytokine production during immune response	0.707213579	0.008712784	0.044743231
GOTERM_BP_ALL	GO:0010921~regulation of phosphatase activity	0.707213579	0.008712784	0.044743231
GOTERM_BP_ALL	GO:0002275~myeloid cell activation during immune response	0.707213579	0.008712784	0.044743231
GOTERM_BP_ALL	GO:0046823~negative regulation of nucleocytoplasmic transport	0.707213579	0.008712784	0.044743231
GOTERM_BP_ALL	GO:0031344~regulation of cell projection organization	1.555869873	0.008764882	0.044950706
GOTERM_BP_ALL	GO:0033365~protein localization in organelle	2.121640736	0.008799138	0.045067939
GOTERM_BP_ALL	GO:0032963~collagen metabolic process	0.848656294	0.009005239	0.046047243

GOTERM_BP_ALL	GO:0010811~positive regulation of cell-substrate adhesion	0.848656294	0.009005239	0.046047243
GOTERM_BP_ALL	GO:0007595~lactation	0.848656294	0.009005239	0.046047243
GOTERM_BP_ALL	GO:0032259~methylation	1.414427157	0.009035999	0.046145209
GOTERM_BP_ALL	GO:0008213~protein amino acid alkylation	0.99009901	0.009112762	0.04647307
GOTERM_BP_ALL	GO:0006479~protein amino acid methylation	0.99009901	0.009112762	0.04647307
GOTERM_BP_ALL	GO:0043623~cellular protein complex assembly	2.263083451	0.009333086	0.047516859
GOTERM_BP_ALL	GO:0032990~cell part morphogenesis	3.111739745	0.009388364	0.047734756
GOTERM_BP_ALL	GO:0033151~V(D)J recombination	0.565770863	0.009659732	0.049027184
GOTERM_BP_ALL	GO:0042523~positive regulation of tyrosine phosphorylation of Stat5 protein	0.565770863	0.009659732	0.049027184
GOTERM_BP_ALL	GO:0070227~lymphocyte apoptosis	0.565770863	0.009659732	0.049027184
GOTERM_BP_ALL	GO:0010829~negative regulation of glucose transport	0.565770863	0.009659732	0.049027184
GOTERM_BP_ALL	GO:0032891~negative regulation of organic acid transport	0.565770863	0.009659732	0.049027184
GOTERM_BP_ALL	GO:0006349~genetic imprinting	0.565770863	0.009659732	0.049027184
GOTERM_BP_ALL	GO:0030858~positive regulation of epithelial cell differentiation	0.565770863	0.009659732	0.049027184
GOTERM_BP_ALL	GO:0046685~response to arsenic	0.565770863	0.009659732	0.049027184
GOTERM_CC_ALL	GO:0044428~nuclear part	24.46958982	5.89E-25	2.96E-22
GOTERM_CC_ALL	GO:0005654~nucleoplasm	15.2758133	2.23E-23	5.60E-21
GOTERM_CC_ALL	GO:0005634~nucleus	47.38330976	1.20E-21	2.02E-19
GOTERM_CC_ALL	GO:0031981~nuclear lumen	20.08486563	2.09E-21	2.62E-19
GOTERM_CC_ALL	GO:0031974~membrane-enclosed lumen	22.91371994	1.68E-19	1.41E-17
GOTERM_CC_ALL	GO:0043233~organelle lumen	22.63083451	1.52E-19	1.52E-17
GOTERM_CC_ALL	GO:0005615~extracellular space	11.88118812	3.39E-18	2.44E-16
GOTERM_CC_ALL	GO:0070013~intracellular organelle lumen	21.49929279	2.21E-17	1.39E-15
GOTERM_CC_ALL	GO:0043227~membrane-bounded organelle	62.37623762	1.04E-14	5.77E-13
GOTERM_CC_ALL	GO:0043231~intracellular membrane-bounded organelle	62.23479491	1.61E-14	8.10E-13
GOTERM_CC_ALL	GO:0044421~extracellular region part	13.43705799	2.54E-14	1.16E-12
GOTERM_CC_ALL	GO:0044424~intracellular part	76.52050919	2.05E-13	8.60E-12
GOTERM_CC_ALL	GO:0044451~nucleoplasm part	9.052333805	1.33E-12	5.13E-11
GOTERM_CC_ALL	GO:0005622~intracellular	77.79349364	5.25E-12	1.76E-10
GOTERM_CC_ALL	GO:0005737~cytoplasm	56.85997171	5.17E-12	1.86E-10
GOTERM_CC_ALL	GO:0005829~cytosol	15.70014144	9.52E-12	2.99E-10
GOTERM_CC_ALL	GO:0009986~cell surface	6.506364922	3.39E-11	1.00E-09
GOTERM_CC_ALL	GO:0000228~nuclear chromosome	4.243281471	5.11E-11	1.43E-09
GOTERM_CC_ALL	GO:0043234~protein complex	25.03536068	5.62E-11	1.49E-09
GOTERM_CC_ALL	GO:0005667~transcription factor complex	4.809052334	9.08E-11	2.28E-09
GOTERM_CC_ALL	GO:0005694~chromosome	7.496463932	1.56E-10	3.73E-09
GOTERM_CC_ALL	GO:0009897~external side of plasma membrane	4.243281471	1.71E-10	3.90E-09

GOTERM_CC_ALL	GO:0043226~organelle	65.7708628	1.98E-10	4.33E-09
GOTERM_CC_ALL	GO:0044446~intracellular organelle part	36.0678925	2.71E-10	5.68E-09
GOTERM_CC_ALL	GO:0044422~organelle part	36.20933522	3.13E-10	6.29E-09
GOTERM_CC_ALL	GO:0043229~intracellular organelle	65.34653465	7.20E-10	1.39E-08
GOTERM_CC_ALL	GO:0045121~membrane raft	3.677510608	1.84E-09	3.43E-08
GOTERM_CC_ALL	GO:0032991~macromolecular complex	27.86421499	7.50E-09	1.35E-07
GOTERM_CC_ALL	GO:0044454~nuclear chromosome part	3.253182461	9.56E-09	1.66E-07
GOTERM_CC_ALL	GO:0044459~plasma membrane part	20.93352192	1.37E-08	2.30E-07
GOTERM_CC_ALL	GO:0005901~caveola	2.121640736	2.42E-08	3.92E-07
GOTERM_CC_ALL	GO:0000267~cell fraction	12.02263083	7.03E-08	1.11E-06
GOTERM_CC_ALL	GO:0044427~chromosomal part	5.940594059	8.33E-08	1.27E-06
GOTERM_CC_ALL	GO:0000781~chromosome, telomeric region	1.555869873	1.88E-07	2.78E-06
GOTERM_CC_ALL	GO:0043235~receptor complex	2.828854314	4.67E-07	6.71E-06
GOTERM_CC_ALL	GO:0000784~nuclear chromosome, telomeric region	1.272984441	1.36E-06	1.90E-05
GOTERM_CC_ALL	GO:0005887~integral to plasma membrane	12.16407355	1.77E-06	2.40E-05
GOTERM_CC_ALL	GO:0005730~nucleolus	8.203677511	2.15E-06	2.84E-05
GOTERM_CC_ALL	GO:0031226~intrinsic to plasma membrane	12.30551627	2.43E-06	3.13E-05
GOTERM_CC_ALL	GO:0044444~cytoplasmic part	37.62376238	2.69E-06	3.39E-05
GOTERM_CC_ALL	GO:0032300~mismatch repair complex	0.848656294	2.79E-06	3.42E-05
GOTERM_CC_ALL	GO:0016605~PML body	1.414427157	7.09E-06	8.49E-05
GOTERM_CC_ALL	GO:0005792~microsome	3.818953324	1.11E-05	1.30E-04
GOTERM_CC_ALL	GO:0042598~vesicular fraction	3.818953324	1.85E-05	2.11E-04
GOTERM_CC_ALL	GO:0043232~intracellular non-membrane-bounded organelle	21.6407355	2.09E-05	2.34E-04
GOTERM_CC_ALL	GO:0043228~non-membrane-bounded organelle	21.6407355	2.09E-05	2.34E-04
GOTERM_CC_ALL	GO:0070603~SWI/SNF-type complex	0.99009901	2.18E-05	2.38E-04
GOTERM_CC_ALL	GO:0016514~SWI/SNF complex	0.99009901	2.18E-05	2.38E-04
GOTERM_CC_ALL	GO:0005626~insoluble fraction	8.628005658	6.65E-05	7.11E-04
GOTERM_CC_ALL	GO:0005576~extracellular region	17.1145686	8.46E-05	8.87E-04
GOTERM_CC_ALL	GO:0016604~nuclear body	2.828854314	1.09E-04	0.001121065
GOTERM_CC_ALL	GO:0000793~condensed chromosome	2.404526167	1.28E-04	0.001283759
GOTERM_CC_ALL	GO:0016585~chromatin remodeling complex	1.697312588	2.03E-04	0.002003508
GOTERM_CC_ALL	GO:0005624~membrane fraction	8.062234795	2.66E-04	0.002570304
GOTERM_CC_ALL	GO:0048471~perinuclear region of cytoplasm	3.818953324	2.92E-04	0.002769073
GOTERM_CC_ALL	GO:0000783~nuclear telomere cap complex	0.707213579	3.55E-04	0.003302063
GOTERM_CC_ALL	GO:0000782~telomere cap complex	0.707213579	3.55E-04	0.003302063
GOTERM_CC_ALL	GO:0060205~cytoplasmic membrane-bounded vesicle lumen	1.272984441	4.94E-04	0.0045045
GOTERM_CC_ALL	GO:0000790~nuclear chromatin	1.414427157	5.03E-04	0.004510049
GOTERM_CC_ALL	GO:0016363~nuclear matrix	1.414427157	5.77E-04	0.005080747
GOTERM_CC_ALL	GO:0031983~vesicle lumen	1.272984441	6.74E-04	0.005828668

GOTERM_CC_ALL	GO:0070419~nonhomologous end joining complex	0.565770863	7.35E-04	0.006251088
GOTERM_CC_ALL	GO:0005958~DNA-dependent protein kinase-DNA ligase 4 complex	0.565770863	7.35E-04	0.006251088
GOTERM_CC_ALL	GO:0000307~cyclin-dependent protein kinase holoenzyme complex	0.707213579	8.68E-04	0.007253119
GOTERM_CC_ALL	GO:0005625~soluble fraction	3.818953324	0.001022017	0.008396311
GOTERM_CC_ALL	GO:0034399~nuclear periphery	1.414427157	0.001092069	0.008825515
GOTERM_CC_ALL	GO:0005886~plasma membrane	27.86421499	0.001210003	0.009620092
GOTERM_CC_ALL	GO:0005815~microtubule organizing center	3.253182461	0.00136273	0.010660279
GOTERM_CC_ALL	GO:0031988~membrane-bounded vesicle	5.799151344	0.001475622	0.011362433
GOTERM_CC_ALL	GO:0031093~platelet alpha granule lumen	1.131541726	0.001625313	0.01213764
GOTERM_CC_ALL	GO:0000792~heterochromatin	1.131541726	0.001625313	0.01213764
GOTERM_CC_ALL	GO:0005819~spindle	2.263083451	0.001624817	0.012316669
GOTERM_CC_ALL	GO:0045120~pronucleus	0.707213579	0.001755847	0.012915388
GOTERM_CC_ALL	GO:0045177~apical part of cell	2.545968883	0.001807808	0.013103968
GOTERM_CC_ALL	GO:0031982~vesicle	6.506364922	0.001971444	0.014080149
GOTERM_CC_ALL	GO:0016328~lateral plasma membrane	0.707213579	0.002375455	0.016707801
GOTERM_CC_ALL	GO:0000785~chromatin	2.687411598	0.002447178	0.016971547
GOTERM_CC_ALL	GO:0042611~MHC protein complex	1.272984441	0.00282315	0.019291659
GOTERM_CC_ALL	GO:0044433~cytoplasmic vesicle part	2.545968883	0.00287021	0.019348097
GOTERM_CC_ALL	GO:0043005~neuron projection	3.818953324	0.003535136	0.023471149
GOTERM_CC_ALL	GO:0005813~centrosome	2.828854314	0.003709696	0.024297918
GOTERM_CC_ALL	GO:0005892~nicotinic acetylcholine-gated receptor-channel complex	0.707213579	0.004033843	0.026058693
GOTERM_CC_ALL	GO:0042995~cell projection	6.364922207	0.00659644	0.041263739
GOTERM_CC_ALL	GO:0016323~basolateral plasma membrane	2.545968883	0.006562148	0.041568224
GOTERM_CC_ALL	GO:0031012~extracellular matrix	3.677510608	0.007612402	0.046910135
GOTERM_CC_ALL	GO:0016023~cytoplasmic membrane-bounded vesicle	5.233380481	0.007912498	0.048134046
GOTERM_MF_ALL	GO:0005515~protein binding	77.22772277	2.88E-50	2.93E-47
GOTERM_MF_ALL	GO:0016563~transcription activator activity	10.04243281	8.19E-23	4.17E-20
GOTERM_MF_ALL	GO:0030528~transcription regulator activity	20.65063649	6.92E-20	2.34E-17
GOTERM_MF_ALL	GO:0005488~binding	90.0990099	2.20E-19	5.59E-17
GOTERM_MF_ALL	GO:0043566~structure-specific DNA binding	5.374823197	1.71E-18	3.48E-16
GOTERM_MF_ALL	GO:0046983~protein dimerization activity	10.46676096	1.06E-17	1.80E-15
GOTERM_MF_ALL	GO:0003677~DNA binding	26.59123055	2.63E-17	3.82E-15
GOTERM_MF_ALL	GO:0005102~receptor binding	13.86138614	5.53E-17	7.02E-15
GOTERM_MF_ALL	GO:0008134~transcription factor binding	9.900990099	9.45E-17	1.02E-14
GOTERM_MF_ALL	GO:0003690~double-stranded DNA binding	4.243281471	8.64E-17	1.13E-14
GOTERM_MF_ALL	GO:0042802~identical protein binding	11.31541726	7.37E-17	1.25E-14
GOTERM_MF_ALL	GO:0003684~damaged DNA binding	3.111739745	6.08E-16	4.71E-14

GOTERM_MF_ALL	GO:0043565~sequence-specific DNA binding	10.60820368	2.13E-15	1.65E-13
GOTERM_MF_ALL	GO:0042803~protein homodimerization activity	6.930693069	6.07E-13	4.41E-11
GOTERM_MF_ALL	GO:0010843~promoter binding	2.828854314	2.23E-12	1.51E-10
GOTERM_MF_ALL	GO:0046982~protein heterodimerization activity	4.95049505	4.50E-11	2.86E-09
GOTERM_MF_ALL	GO:0004714~transmembrane receptor protein tyrosine kinase activity	2.828854314	5.55E-11	3.32E-09
GOTERM_MF_ALL	GO:0046332~SMAD binding	2.404526167	5.92E-11	3.34E-09
GOTERM_MF_ALL	GO:0004672~protein kinase activity	9.193776521	1.09E-10	5.84E-09
GOTERM_MF_ALL	GO:0019899~enzyme binding	8.345120226	1.31E-10	6.66E-09
GOTERM_MF_ALL	GO:0005125~cytokine activity	4.667609618	1.52E-10	7.35E-09
GOTERM_MF_ALL	GO:0008094~DNA-dependent ATPase activity	2.545968883	2.39E-10	1.10E-08
GOTERM_MF_ALL	GO:0060089~molecular transducer activity	22.91371994	4.23E-10	1.87E-08
GOTERM_MF_ALL	GO:0004871~signal transducer activity	22.91371994	4.23E-10	1.87E-08
GOTERM_MF_ALL	GO:0003700~transcription factor activity	12.30551627	7.14E-10	3.03E-08
GOTERM_MF_ALL	GO:0047485~protein N-terminus binding	2.687411598	2.75E-09	1.12E-07
GOTERM_MF_ALL	GO:0003676~nucleic acid binding	29.7029703	3.88E-09	1.52E-07
GOTERM_MF_ALL	GO:0016772~transferase activity, transferring phosphorus-containing groups	11.88118812	4.55E-09	1.71E-07
GOTERM_MF_ALL	GO:0003824~catalytic activity	42.99858557	4.75E-09	1.73E-07
GOTERM_MF_ALL	GO:0008022~protein C-terminus binding	3.536067893	1.45E-08	5.10E-07
GOTERM_MF_ALL	GO:0016773~phosphotransferase activity, alcohol group as acceptor	9.476661952	1.98E-08	6.71E-07
GOTERM_MF_ALL	GO:0016301~kinase activity	10.46676096	2.18E-08	7.16E-07
GOTERM_MF_ALL	GO:0003713~transcription coactivator activity	4.384724187	2.54E-08	8.06E-07
GOTERM_MF_ALL	GO:0003712~transcription cofactor activity	5.940594059	4.43E-08	1.37E-06
GOTERM_MF_ALL	GO:0003697~single-stranded DNA binding	2.121640736	8.68E-08	2.60E-06
GOTERM_MF_ALL	GO:0004713~protein tyrosine kinase activity	3.677510608	9.02E-08	2.62E-06
GOTERM_MF_ALL	GO:0016740~transferase activity	17.53889675	1.48E-07	4.18E-06
GOTERM_MF_ALL	GO:0004520~endodeoxyribonuclease activity	1.414427157	1.97E-07	5.41E-06
GOTERM_MF_ALL	GO:0001883~purine nucleoside binding	16.26591231	2.38E-07	6.37E-06
GOTERM_MF_ALL	GO:0001882~nucleoside binding	16.26591231	3.35E-07	8.73E-06
GOTERM_MF_ALL	GO:0030554~adenyl nucleotide binding	15.98302687	3.61E-07	9.17E-06
GOTERM_MF_ALL	GO:0005524~ATP binding	15.13437058	4.72E-07	1.17E-05
GOTERM_MF_ALL	GO:0032559~adenyl ribonucleotide binding	15.2758133	5.01E-07	1.21E-05
GOTERM_MF_ALL	GO:0000217~DNA secondary structure binding	0.99009901	5.80E-07	1.37E-05
GOTERM_MF_ALL	GO:0003682~chromatin binding	3.253182461	8.45E-07	1.95E-05
GOTERM_MF_ALL	GO:0004536~deoxyribonuclease activity	1.555869873	1.46E-06	3.29E-05

GOTERM_MF_ALL	GO:0016564~transcription repressor activity	4.95049505	1.91E-06	4.23E-05
GOTERM_MF_ALL	GO:0032403~protein complex binding	3.677510608	2.20E-06	4.75E-05
GOTERM_MF_ALL	GO:0017076~purine nucleotide binding	18.10466761	2.25E-06	4.77E-05
GOTERM_MF_ALL	GO:0032555~purine ribonucleotide binding	17.39745403	3.19E-06	6.62E-05
GOTERM_MF_ALL	GO:0032553~ribonucleotide binding	17.39745403	3.19E-06	6.62E-05
GOTERM_MF_ALL	GO:0019838~growth factor binding	2.545968883	3.77E-06	7.67E-05
GOTERM_MF_ALL	GO:0035258~steroid hormone receptor binding	1.555869873	4.46E-06	8.73E-05
GOTERM_MF_ALL	GO:0046906~tetrapyrrole binding	2.828854314	4.40E-06	8.77E-05
GOTERM_MF_ALL	GO:0000166~nucleotide binding	20.22630835	6.11E-06	1.17E-04
GOTERM_MF_ALL	GO:0004518~nuclease activity	3.111739745	7.40E-06	1.34E-04
GOTERM_MF_ALL	GO:0003678~DNA helicase activity	1.555869873	7.38E-06	1.37E-04
GOTERM_MF_ALL	GO:0051427~hormone receptor binding	2.263083451	7.29E-06	1.37E-04
GOTERM_MF_ALL	GO:0004003~ATP-dependent DNA helicase activity	1.272984441	8.32E-06	1.49E-04
GOTERM_MF_ALL	GO:0032404~mismatch repair complex binding	0.848656294	8.74E-06	1.51E-04
GOTERM_MF_ALL	GO:0016705~oxidoreductase activity, acting on paired donors, with incorporation or reduction of molecular oxygen	2.828854314	8.67E-06	1.52E-04
GOTERM_MF_ALL	GO:0008083~growth factor activity	3.111739745	9.95E-06	1.69E-04
GOTERM_MF_ALL	GO:0050681~androgen receptor binding	1.272984441	1.16E-05	1.93E-04
GOTERM_MF_ALL	GO:0031625~ubiquitin protein ligase binding	1.414427157	2.12E-05	3.48E-04
GOTERM_MF_ALL	GO:0005518~collagen binding	1.414427157	2.12E-05	3.48E-04
GOTERM_MF_ALL	GO:0020037~heme binding	2.545968883	2.61E-05	4.22E-04
GOTERM_MF_ALL	GO:0035257~nuclear hormone receptor binding	1.98019802	3.27E-05	5.20E-04
GOTERM_MF_ALL	GO:0004519~endonuclease activity	2.263083451	3.54E-05	5.54E-04
GOTERM_MF_ALL	GO:0004861~cyclin-dependent protein kinase inhibitor activity	0.848656294	3.65E-05	5.62E-04
GOTERM_MF_ALL	GO:0019900~kinase binding	3.111739745	5.00E-05	7.59E-04
GOTERM_MF_ALL	GO:0016538~cyclin-dependent protein kinase regulator activity	0.99009901	6.28E-05	9.38E-04
GOTERM_MF_ALL	GO:0002039~p53 binding	0.99009901	6.28E-05	9.38E-04
GOTERM_MF_ALL	GO:0034061~DNA polymerase activity	1.414427157	7.91E-05	0.001165434
GOTERM_MF_ALL	GO:0004872~receptor activity	16.40735502	9.43E-05	0.001369285
GOTERM_MF_ALL	GO:0004497~monooxygenase activity	2.121640736	9.85E-05	0.001390706
GOTERM_MF_ALL	GO:0016887~ATPase activity	4.526166902	9.80E-05	0.001402573
GOTERM_MF_ALL	GO:0005506~iron ion binding	4.243281471	1.26E-04	0.001755585
GOTERM_MF_ALL	GO:0000287~magnesium ion binding	5.516265912	1.38E-04	0.00189661
GOTERM_MF_ALL	GO:0019904~protein domain specific binding	4.384724187	1.92E-04	0.002594122
GOTERM_MF_ALL	GO:0042623~ATPase activity, coupled	3.818953324	2.19E-04	0.002925768
GOTERM_MF_ALL	GO:0019207~kinase regulator activity	1.98019802	2.40E-04	0.003125672
GOTERM_MF_ALL	GO:0005021~vascular endothelial growth factor receptor activity	0.707213579	2.39E-04	0.003146862

GOTERM_MF_ALL	GO:0003705~RNA polymerase II transcription factor activity, enhancer binding	1.272984441	2.72E-04	0.003498201
GOTERM_MF_ALL	GO:0003707~steroid hormone receptor activity	1.414427157	2.76E-04	0.003499869
GOTERM_MF_ALL	GO:0032137~guanine/thymine mispair binding	0.565770863	3.43E-04	0.004292117
GOTERM_MF_ALL	GO:0000104~succinate dehydrogenase activity	0.565770863	3.43E-04	0.004292117
GOTERM_MF_ALL	GO:0032134~mispaired DNA binding	0.565770863	3.43E-04	0.004292117
GOTERM_MF_ALL	GO:0000405~bubble DNA binding	0.565770863	3.43E-04	0.004292117
GOTERM_MF_ALL	GO:0032138~single base insertion or deletion binding	0.565770863	3.43E-04	0.004292117
GOTERM_MF_ALL	GO:0043138~3'-5' DNA helicase activity	0.565770863	3.43E-04	0.004292117
GOTERM_MF_ALL	GO:0009055~electron carrier activity	3.253182461	3.73E-04	0.004616611
GOTERM_MF_ALL	GO:0003887~DNA-directed DNA polymerase activity	1.131541726	4.34E-04	0.005302547
GOTERM_MF_ALL	GO:0004386~helicase activity	2.404526167	5.14E-04	0.006205588
GOTERM_MF_ALL	GO:0030145~manganese ion binding	2.545968883	5.22E-04	0.006230039
GOTERM_MF_ALL	GO:0019825~oxygen binding	1.272984441	5.48E-04	0.006462302
GOTERM_MF_ALL	GO:0070412~R-SMAD binding	0.707213579	6.66E-04	0.007672262
GOTERM_MF_ALL	GO:0070330~aromatase activity	0.99009901	6.62E-04	0.007706412
GOTERM_MF_ALL	GO:0005160~transforming growth factor beta receptor binding	0.848656294	6.90E-04	0.007860799
GOTERM_MF_ALL	GO:0030291~protein serine/threonine kinase inhibitor activity	0.848656294	6.90E-04	0.007860799
GOTERM_MF_ALL	GO:0032135~DNA insertion or deletion binding	0.565770863	8.28E-04	0.009314736
GOTERM_MF_ALL	GO:0032407~MutSalpha complex binding	0.565770863	8.28E-04	0.009314736
GOTERM_MF_ALL	GO:0016566~specific transcriptional repressor activity	1.131541726	9.21E-04	0.010029836
GOTERM_MF_ALL	GO:0019887~protein kinase regulator activity	1.697312588	9.10E-04	0.010120085
GOTERM_MF_ALL	GO:0004675~transmembrane receptor protein serine/threonine kinase activity	0.848656294	9.21E-04	0.010134967
GOTERM_MF_ALL	GO:0005024~transforming growth factor beta receptor activity	0.848656294	9.21E-04	0.010134967
GOTERM_MF_ALL	GO:0030234~enzyme regulator activity	8.203677511	0.001007464	0.010732591
GOTERM_MF_ALL	GO:0004879~ligand-dependent nuclear receptor activity	1.414427157	1.00E-03	0.010764299
GOTERM_MF_ALL	GO:0001948~glycoprotein binding	1.131541726	0.001093257	0.011521145
GOTERM_MF_ALL	GO:0030983~mismatched DNA binding	0.848656294	0.001204525	0.012556988
GOTERM_MF_ALL	GO:0008757~S-adenosylmethionine-dependent methyltransferase activity	1.697312588	0.001235301	0.012617475
GOTERM_MF_ALL	GO:0043176~amine binding	1.98019802	0.001227019	0.012660445
GOTERM_MF_ALL	GO:0016491~oxidoreductase activity	6.930693069	0.001496466	0.01511506
GOTERM_MF_ALL	GO:0043498~cell surface binding	0.99009901	0.001524231	0.0152423
GOTERM_MF_ALL	GO:0015026~coreceptor activity	0.848656294	0.00154762	0.015324013
GOTERM_MF_ALL	GO:0032405~MutLalpha complex binding	0.565770863	0.001600717	0.015693361

GOTERM_MF_ALL	GO:0004674~protein serine/threonine kinase activity	4.809052334	0.001764781	0.017124448
GOTERM_MF_ALL	GO:0016712~oxidoreductase activity, acting on paired donors, with incorporation or reduction of molecular oxygen, reduced flavin or flavoprotein as one donor, and incorporation of one atom of oxygen	0.99009901	0.001834393	0.017626526
GOTERM_MF_ALL	GO:0004527~exonuclease activity	1.272984441	0.002264178	0.021513138
GOTERM_MF_ALL	GO:0016279~protein-lysine N-methyltransferase activity	0.99009901	0.002595382	0.024397764
GOTERM_MF_ALL	GO:0016278~lysine N-methyltransferase activity	0.99009901	0.002595382	0.024397764
GOTERM_MF_ALL	GO:0018024~histone-lysine N-methyltransferase activity	0.99009901	0.002595382	0.024397764
GOTERM_MF_ALL	GO:0019955~cytokine binding	1.838755304	0.003394156	0.031508916
GOTERM_MF_ALL	GO:0042162~telomeric DNA binding	0.707213579	0.00362232	0.033291811
GOTERM_MF_ALL	GO:0005539~glycosaminoglycan binding	2.121640736	0.003958302	0.036004762
GOTERM_MF_ALL	GO:0046965~retinoid X receptor binding	0.565770863	0.004190436	0.037743395
GOTERM_MF_ALL	GO:0004888~transmembrane receptor activity	11.17397454	0.004662041	0.041544346

Supplementary Table S6: The significantly enriched InterPro annotations in the 724 human protein-coding CPGs

Annotation source	Functional term	Proportion of annotated genes to all 724 coding CPGs	Raw *P*-value	Benjamini-Hochberg adjusted *P*-value
INTERPRO	IPR008266:Tyrosine protein kinase, active site	3.111739745	2.82E-10	3.55E-07
INTERPRO	IPR001245:Tyrosine protein kinase	3.111739745	1.20E-08	7.54E-06
INTERPRO	IPR017441:Protein kinase, ATP binding site	6.364922207	1.16E-07	4.86E-05
INTERPRO	IPR000719:Protein kinase, core	6.364922207	4.11E-07	1.29E-04
INTERPRO	IPR001824:Receptor tyrosine kinase, class III, conserved site	0.848656294	1.24E-05	0.003127369
INTERPRO	IPR012346:p53 and RUNT-type transcription factor, DNA-binding	0.707213579	3.91E-05	0.00490733
INTERPRO	IPR017972:Cytochrome P450, conserved site	1.697312588	3.78E-05	0.00526432
INTERPRO	IPR001357:BRCT	1.131541726	3.51E-05	0.005511348
INTERPRO	IPR013088:Zinc finger, NHR/GATA-type	1.555869873	3.45E-05	0.006189713
INTERPRO	IPR001128:Cytochrome P450	1.697312588	3.22E-05	0.006736429
INTERPRO	IPR016129:Peptidase C14, ICE, catalytic subunit p20, active site	0.848656294	7.06E-05	0.008042128
INTERPRO	IPR002138:Peptidase C14, caspase non-catalytic subunit p10	0.848656294	7.06E-05	0.008042128
INTERPRO	IPR001628:Zinc finger, nuclear hormone receptor-type	1.414427157	8.66E-05	0.008347505
INTERPRO	IPR017973:Cytochrome P450, C-terminal region	1.555869873	8.01E-05	0.008358082

INTERPRO	IPR008946:Nuclear hormone receptor, ligand-binding	1.414427157	1.23E-04	0.010953917
INTERPRO	IPR000536:Nuclear hormone receptor, ligand-binding, core	1.414427157	1.23E-04	0.010953917
INTERPRO	IPR002401:Cytochrome P450, E-class, group I	1.414427157	1.45E-04	0.012068347
INTERPRO	IPR015917:Peptidase C14, caspase precursor p45, core	0.848656294	1.67E-04	0.013019717
INTERPRO	IPR013783:Immunoglobulin-like fold	5.940594059	1.81E-04	0.013315492
INTERPRO	IPR011600:Peptidase C14, caspase catalytic	0.848656294	2.42E-04	0.015871426
INTERPRO	IPR001309:Peptidase C14, ICE, catalytic subunit p20	0.848656294	2.42E-04	0.015871426
INTERPRO	IPR001092:Basic helix-loop-helix dimerisation region bHLH	2.121640736	2.31E-04	0.016005467
INTERPRO	IPR011029:DEATH-like	1.555869873	3.72E-04	0.023135496
INTERPRO	IPR012351:Four-helical cytokine, core	1.272984441	4.98E-04	0.029372503
INTERPRO	IPR001723:Steroid hormone receptor	1.272984441	5.78E-04	0.032542981
INTERPRO	IPR003151:PIK-related kinase, FAT	0.565770863	6.42E-04	0.033109066
INTERPRO	IPR015592:Ras small GTPase, Ras-related	0.565770863	6.42E-04	0.033109066
INTERPRO	IPR009134:Vascular endothelial growth factor receptor, VEGFR, N-terminal	0.565770863	6.42E-04	0.033109066
INTERPRO	IPR018936:Phosphatidylinositol 3- and 4-kinase, conserved site	0.848656294	6.22E-04	0.033476525
INTERPRO	IPR000403:Phosphatidylinositol 3- and 4-kinase, catalytic	0.848656294	6.22E-04	0.033476525
INTERPRO	IPR003006:Immunoglobulin/major histocompatibility complex, conserved site	1.697312588	8.25E-04	0.039139175
INTERPRO	IPR007110:Immunoglobulin-like	5.233380481	7.96E-04	0.03926615
INTERPRO	IPR002477:Peptidoglycan binding-like	0.848656294	0.00105212	0.047863437
INTERPRO	IPR002398:Peptidase C14, caspase precursor p45	0.848656294	0.00105212	0.047863437

Supplementary Table S7: The statistics of CPGs in 17 major cancer types

Cancer type	Number of CPGs	Genes
Breast cancer	142	MLH1, NAT2, ACACA, APC, APEX1, FAS, FASLG, ATM, ATR, BACH1, BARD1, CCND1, BCL2, BRCA1, BRCA2, CASP8, CASP9, CASP10, CDH1, COMT, CTBP1, CTLA4, CYP1A1, CYP1B1, CYP2E1, CYP11A1, CYP17A1, CYP19A1, GADD45A, NQO1, ENO1, ERBB2, ESR1, ESR2, ESRRG, FGFR2, FGFR4, FH, FLT1, XRCC6, GSPT1, GSTM1, GSTP1, GSTT1, HIF1A, HLA-G, HMMR, HSD17B2, IL2, IL13, LTA, MAP3K1, MMP2, MMP7, MRE11A, MTHFR, MTNR1A, MTNR1B, MTR, MTRR, NEK2, NQO2, NOS3, YBX1, OGG1, PDCD1, PGR, ABCB1, PHB, PIK3CA, PMS2, PON1, PTEN, RAD51, RAD51C, RAD51D, RB1, RNASEL, CXCL12, SELE, SHBG, AURKA, STK11, TGFB1, TGFBR1, ICAM5, TLR2, TLR4, TP53, TSG101, TWIST1, TYMS, VDR, VEGFA, XRCC1, XRCC2, XRCC3, PRDM2, PSCA, BRCATA, BCPR, NCOA3, BAP1, RAD54L, PPM1D, IQGAP1, PER2, MBD2, THEMIS2, CLOCK, RB1CC1, RAD50, PPARGC1A, MORF4L1, TOPBP1, PSIP1, CHEK2, AKAP13, FAN1, RRP1B, FBXO10, TOX3, UGT1A1, WRAP53, LAPTM4B, FANCM, ZNF350, BRCA3, NOD2, MCPH1, PALB2, ATG10, BRIP1, FAM175A, CNTROB, PPARGC1B, MIER3, MIR146A, MIR196A2, MIR27A, MIR499A, KLLN
Prostate cancer	108	NAT1, NAT2, APEX1, BIRC5, AR, ZFHX3, BCL2, BMPR1B, BRCA1, BRCA2, CAV1, CD14, CDH1, CDH13, CCR5, KLF6, CYP1A1, CYP1B1, CYP17A1, CYP19A1, ACE, NQO1, EGF, EPHB2, ERCC6, FGFR4, GCNT1, GNB3, GPX1, GSTA1, GSTM1, GSTP1, GSTT1, HIF1A, HIP1, HPN, IGF1, IGFBP3, CYR61, IL6, IL16, IRS1, ITGA2, CD82, KLK2, LGALS3, LHB, MDM2, MLH1, MSMB, MSR1, MTHFR, MXI1, MYC, NBN, NKX3-1, NOS3, OGG1, POU5F1, POU5F1B, PPP2R2A, PRKDC, PTEN, CCL5, STX1A, HNF1B, TGFB1, TNF, TP53, TSG101, UGT2B17, VDR, XRCC3, PCAP, FGF23, CPNE3, GDF15, HPCX, KLK4, MAGEC1, HOXB13, CHEK2, POLI, EHBP1, GNMT, IRX4, PDE11A, BTNL2, ELAC2, RINT1, ESCO1, ARL11, RFX6, FMN1, HPCQTL19, MIR146A, HPC3, HPC4, HPC5, CCR2, HPCX2, HPC6, HPC7, HPC9, HPC10, HPC14, HPC15,RNASEL
Lung cancer	105	NAT2, AGER, APEX1, BIRC5, FAS, FASLG, BAX, CCND1, CASP5, CASP8, CASP9, CD28, TNFSF8, CDK5, CHRNA3, CHRNA5, CHRNB3, CHRNB4, MAP3K8, CREB1, CTLA4, CYP1A1, CYP1B1, CYP2A6, CYP2E1, NQO1, DNMT3B, EGFR, EPHX1, ERBB2, ERCC1, ERCC6, FEN1, GPC5, XRCC6, GSTM1, GSTP1, GSTT1, GTF2H1, IFNG, IL4, IL6, IL10, IL17A, JUN, KRAS, MAD2L1, MMP1, MMP2, MMP3, MPO, ABCC1, MTHFR, MUC1, NME1, OGG1, ABCB1, PIN1, PPP2R1B, PTGIS, PTGS2, REV3L, CCL2, CXCL12, SMARCA2, SULT1A1, TERT, TGFB1, TNF, TP53, TP73, TSG101, VEGFA, XRCC1, XRCC3, CXCR4, SCLC1, PRRC2A, AXIN2, MAD1L1, TP63, TNFRSF10A, MBD4, CHRNA6, ADIPOQ, HDAC9, DLEC1, PDCD6IP, PDCD6, RASSF1, CHEK2, XRN2, RGS17, ICOS, TSG11, SUV39H2, CLPTM1L, HAVCR2, C18orf54, LIN28B, LNCR1, LNCR3, LNCR4, LNCR5, NKX2-1
Colon cancer	103	CYP1A1, CYP1B1, DES, ENO1, GSTM1, GSTT1, CXCL8, MBL2, ODC1, PCK2, PTPN12, PTPRJ, RBBP8, SRC, AXIN2, RAD54B, NAT2, ALOX12, APC, APEX1, BLM, BMP2, BMP4, BMPR1A, BRCA1, BUB1, CASP8, CASP9, CD14, CD80, CDH1, CRP, CSE1L, CTNNB1, CYP1A2, CYP2E1, DNMT3B, EFNA1, EP300, GSTP1, MSH6, IGF2, IL17A, IL18, KCNQ1, RPSA, LBP, EPCAM, SMAD4, SMAD7, MCC, MDM2, MLH1, MMP2, MSH2, MTHFR, MUTYH, MYC, NBN, NFKB1, NRAS, OGG1, ABCB1, PLA2G2A, PLD2, PMS2, POLD1, PTGS2, RPS20, AURKA, STK11, TCN2, TGFB1, TGFBR1, TP53, VEGFA, XRCC2, XRCC3, XRCC4, TNFRSF10A, GREM1, MLH3, PLCE1, CHFR, ABCG8, NSD1, GALNT12, COLCA2, COLCA1, MIR146A, MIR149, MIR196A1, MIR196A2, CRCS6, CRCS7, CRCS2, CRCS5, CRCS8, CRCS11, CRCS9, POLE, BRAF, CD86
Gastric cancer	91	BLM, ADD1, ALDH2, BIRC5, FAS, CCND1, BMPR1A, CASP7, CAV1, RUNX3, CDH1, CTLA4, CYP2E1, DNMT3A, FOLR1, XRCC6, GPX1, GSPT1, GSTM1, MSH6, HLA-DQA1, HLA-DQB1, HMOX1, HRAS, IGFBP3, IL1B, IL1RN, IL6, IL7, CXCL8, IL10, IL15RA, IL17A, LOX, MDM2, MIF, MLH1, MPO, MTHFR, MTRR, MUC1, MUC5AC, MYCL, NOS2, OGG1, PGC, ABCB1, PMS2, PRKAA1, PTGER4, PTGS2, PTPRCAP, SELE, SOD2, SPP1, HSPA13, STK11, TGFB1, TLR2, TLR4, TNFAIP2, TP53, TP53BP2, TSG101, TYMS, VEGFA, XRCC1, XRCC2, XRCC3, PSCA, ADIPOQ, TXNRD2, CD226, ZBTB20, IL37, PLCE1, TLR9, ABCG8, NSD1, HAVCR2, EGLN2, IL23R, DAB2IP, IRGM, MIR146A, MIR184, MIR196A2, MIR200C, MIR181A1, MIR27A, MIR938

Leukemia	76	IRF4, BLM, MSH6, MSH2, ABL1, ABL2, ARNT, BCL3, BCL6, BCR, RUNX1, CBFB, CEBPA, DKC1, ELANE, ETV6, FLT3, KDSR, GATA1, LMO1, LMO2, LPP, LYL1, MEFV, MLF1, KMT2A, MYB, NPM1, NUMA1, PAX5, PBX1, PML, RARA, RMRP, SH3GL1, STAT5B, TAL1, TAL2, TCF3, TCL4, TERC, TERT, ZBTB16, DEK, LALL, NUP214, CLLS2, TCL1A, TAM, PICALM, GMPS, TCL1B, IKZF1, SEPT9, MLLT11, ARHGAP26, ACSL6, ARHGEF12, TINF2, CHIC2, WHSC1L1, WRAP53, NOP10, NHP2, CTC1, PHF6, CLLS1, CLLS3, CLLS4, CLLS5, ALL1, ALL2, MLSM7, RAP1GDS1, MKL1, RBM15
Hepatocellular cancer	64	APC, GPC3, BIRC5, AR, CDH17, CSE1L, CTLA4, EPHB1, ESR1, EZH2, FAH, XRCC6, GBA, HFE, HIF1A, HLA-DQA1, HLA-DQB1, HLA-G, HMBS, IFNG, IGF2, IGF2R, LEPR, SERPINE1, ABCB1, SERPINA1, CCL5, CXCL12, SPARC, STAT1, STAT4, AURKA, TGFB1, TGFBR1, TGFBR2, TGFBR3, TIMP3, TIMP4, TSG101, UROD, VEGFC, XRCC1, XRCC5, AXIN1, ABCB11, EXO1, ADIPOQ, APOBEC3B, SLC25A13, DLC1, HTATIP2, HCP5, RASSF1, UGT1A10, UGT1A7, IFNL3, MIR196A2, MIR378A, MIR499A, CCR2, PTGS2, PRDM2, GNMT, LAPTM4B
Esophageal cancer	44	ASCC1, CTHRC1, BCL2, BRCA1, CP, CTLA4, CYP1A1, CYP1B1, NQO1, FOXF1, GSTM1, GSTP1, HLA-A, HLA-B, ERAS, IL12B, IL12RB1, MMP2, MTHFR, RNF6, SLC11A1, DLEC1, PLCE1, FAT4, RHBDF2, SPINK7, NAT2, ADH1B, ADH1C, ADH7, ALOX12, BAG1, COMT, CYP2E1, EZH2, IGFBP3, PTGS2, SOD2, AURKA, XRCC2, XRCC3, TNFRSF6B, LZTS1, WWOX
Thyroid cancer	40	PRKAR1A, XRCC4, XRCC5, BIRC5, ATM, BCL2, BRAF, CASP9, CDKN1B, FOXE1, GSTP1, HLA-B, HRAS, LIG4, MDM2, NTRK1, PCM1, PTEN, PTPRJ, RET, NKX2-1, TSHR, WRN, XRCC1, PAX8, CCDC6, NCOA4, TRIM24, IL32, STK17B, ATG5, MINPP1, GOLGA5, TCO, NDUFA13, TRIM33, SRGAP1, PTCPRN, NMTC1, PTCSC3
Ovarian cancer	39	MLH1, MSH6, HLA-DQA1, LOX, AGER, APEX1, ATM, BRCA1, CCNE1, CDK2, ERBB2, ESR2, MSH2, RNASEL, TGFBR1, VEGFA, SEPT9, PPARGC1A, TOPBP1, RRAS2, LAPTM4B, MCPH1, PALB2, MTHFR, NFKB1, OPCML, PMS2, RAD51C, RAD51D, CCL2, AURKA, TP73, TSG101, VDR, BRIP1, IL23R, IL27, OVCAS1, BRCA2
Cervical cancer	37	NAT2, ATM, CCND1, CD28, CYP1A1, CYP1B1, DEFB4A, EGF, GSTM1, GSTT1, HLA-DPB1, HLA-DQB1, HLA-DRB1, HLA-G, IFNA17, IFNG, IL1B, IL1RN, IL6, LOX, MTHFR, SERPINE1, SPP1, STAT3, TLR2, TLR3, TLR4, TNF, TSG101, CD83, TLR9, LAPTM4B, MICA, MGMT, ST3, CTLA4, TMC6
Bladder cancer	34	PARP1, AKT1, APEX1, BIRC5, CCND1, CASP8, CTLA4, CYP2E1, NQO1, ERCC6, FGFR3, MTOR, GPX1, GSTM1, GSTP1, GSTT1, HSD3B2, CXCL8, LTA, MDM2, MMP1, MMP2, OGG1, PRKDC, PTEN, PTGS2, SLC14A1, THBS1, TP53, XRCC1, XRCC3, PSCA, ARL6IP5, UGT1A7
Renal cancer	27	HAVCR1, CTLA4, BIRC5, FH, MET, SDHB, TSC1, TSC2, ABCG2, FLCN, CAV1, MTOR, GSTM1, GSTP1, GSTT1, HIF1A, IL4, PRCC, HNF1A, TFE3, BAP1, RNF139, DIRC2, KLLN, TMEM127, PTEN, VHL
Oral cancer	23	BIRC5, ATM, CCND1, ACE, GSTM1, GSTM3, GSTP1, GSTT1, ICAM1, IL18, KRAS, MMP14, MTHFR, CXCL12, SPP1, TNF, TP53, VDR, VEGFA, XRCC1, RECK, CCR2, MICA
Squamous cancer	22	RMRP, COL7A1, BLM, DDB2, DKC1, ERCC2, ERCC3, ERCC4, ERCC5, FANCA, FANCC, FANCG, GJB2, ING1, XPA, XPC, TNFRSF10B, RECQL4, DOCK8, BRIP1, RSPO1, OGG1
Melanoma	21	CDKN2A, CDKN2B, CMM, DDB2, ERCC2, ERCC3, ERCC4, ERCC5, MITF, PTEN, TYR, WRN, XPA, XPC, BAP1, POT1, PALB2, UVM2, CMM4, CMM7, UVM1
Nasopharyngeal cancer	21	GSTM1, GSTT1, BIRC5, CAV1, CCNE1, CR2, HLA-A, HSPA1B, IFNG, IL6, IL10, ITGA9, MMP2, NPC1, TLR3, TP73, CD209, LOC344967, MIR151A, NPCA2, PTGS2

Supplementary Table S8: The common CPGs in multiple cancer types

Gene symbol	Number of involved cancers	Cancer
GSTM1	13	squamous cell lung carcinoma, colorectal cancer, laryngeal cancer, esophageal cancer, arsenic induced cancer, breast cancer, prostatic cancer, oral cancer, gastric cancer, cervical cancer, bladder cancer, renal cell carcinoma, nasopharyngeal cancer
MSH6	11	colorectal cancer, endometrial cancer, gastric cancer, ovarian cancer, urothelial cancers, parotid cancer, laryngeal cancer, brain tumors, hematological malignancy, embryonal tumors, endometrial cancer
TP53	10	bladder cancer, skin cancer, gastric cancer, adrenocortical cancer, lung cancer, breast cancer, brain tumor, colorectal cancer, oral squamous cell carcinoma, prostate cancer
GSTP1	9	thyroid cancer, breast cancer, prostatic cancer, oral cancer, lung cancer, colorectal cancer, renal cell carcinoma, bladder cancer, esophageal cancer
MTHFR	9	ovarian cancer, breast cancer, lung cancer, cervical cancer, gastric cancer, esophageal cancer, prostate cancer, colorectal cancer, oral cancer
BIRC5	9	lung cancer, gastric cancer, bladder cancer, nasopharyngeal carcinoma, hepatocellular carcinoma, renal cell cancer, oral cancer, papillary thyroid carcinoma, prostate cancer
CTLA4	9	lung cancer, breast cancer, esophagus cancer, gastric cancer, pancreatic cancer, bladder cancer, renal adenocarcinoma, hepatocellular carcinoma, cervical squamous cell carcinoma
GSTT1	9	colorectal cancer, breast cancer, lung cancer, prostatic cancer, oral cancer, cervical cancer, bladder cancer, renal cell carcinoma, nasopharyngeal cancer
NAT2	8	prostate cancer, gallbladder cancer, pancreatic cancer, lung cancer, cervical cancer, breast cancer, colorectal cancer, esophageal squamous cell carcinoma
MMP2	8	lung cancer, gallbladder cancer, nasopharyngeal carcinoma, breast cancer, esophageal cancer, laryngeal carcinoma, bladder cancer, colorectal cancer
MLH1	8	endometrialcancer, gastric cancer, ovarian cancer, urothelial cancers, colorectal cancer, lynch syndrome cancer, breast cancer, prostate cancers
PMS2	8	colorectal cancer, endometrial cancer, gastric cancer, ovarian cancer, breast cancer, parotid cancer, laryngeal cancer,
TSG101	8	cervical cancer, gallbladder cancer, gastric cancer, ovarian cancer, breast cancer, hepatocellular carcinoma, prostate carcinoma, lung cancer
PTEN	8	breast cancer, skin cancer, thyroid tumor, bladder cancer, renal cancer, malignant melanoma, glioblastoma, prostate cancer
OGG1	8	breast cancer, gastric cancer, head neck cancer, prostate cancer, squamous cell carcinomas, bladder cancer, lung cancer, colorectal cancer
XRCC1	8	breast cancer, bladder cancer, lung cancer, squamous cell carcinoma of the head and neck, gastric cardia cancer, oral cancer, papillary thyroid carcinoma, hepatocellular carcinoma
AURKA	7	skin tumor, esophageal squamous cell carcinoma, breast cancer, penetrance cancer, colorectal cancer, hepatocellular carcinoma, ovarian cancer
NBN	7	prostate cancer, larynx cancer, colorectal cancer, neuroblastoma, wilms tumor, medulloblastoma, medulloblastoma
PTGS2	7	gastric cancer, lung cancer, colorectal cancer, esophageal squamous cell carcinoma, nasopharyngeal carcinoma, bladder cancer, liver cancer
VEGFA	7	gastric cancer, colorectal cancer, ovarian cancer, breast cancer, oral cancer, gallbladder cancer, lung cancer
DICER1	7	embryonal rhabdomyosarcoma, pleuropulmonary blastoma, cystic nephroma, ovarian Sertoli Leydig tumor, familial multinodular goiter, cystic nephroma, medullo epithelioma
XRCC3	7	breast cancer, prostate cancer, colorectal cancer, lung cancer, esophageal squamous cell carcinoma, gastric cardia adenocarcinoma, bladder cancer
BRCA2	7	breast cancer, ovarian cancer, prostate cancer, pancreatic cancer, myeloid neoplasms, medulloblastoma, Wilms tumor

MSH2	7	brain tumors, hematological malignancy, embryonal tumors, colorectal cancer, endometrial cancer, ovarian cancer, epithelioma
MDM2	7	bladder cancer, neuroblastoma, colorectal cancer, gastric cancer, prostate cancer, salivary gland carcinoma, thyroid carcinoma
MIR146A	6	pancreatic cancer, prostatic cancer, colorectal cancer, breast cancer, gastric cancer, squamous cell carcinoma of the head and neck
IL6	6	pancreatic cancer, gastric cancer, prostate cancer, lung cancer, cervical cancer, nasopharyngeal carcinoma
ABCB1	6	lung cancer, colorectal cancer, breast cancer, endometrial cancer, hepatocellular carcinoma, gastric cancer
CYP2E1	6	gastric cancer, lung cancer, breast cancer, esophageal squamous cell cancer, colorectal cancer, bladder cancer
TGFB1	6	gastric cancer, breast cancer, lung cancer, colorectal cancer, prostate cancer, hepatocellular carcinoma
CYP1A1	6	esophageal cancer, lung cancer, colorectal cancer, prostate cancer, breast cancer, cervical cancer
CCND1	6	cervical cancer, gastric cardiac carcinoma, lung cancer, breast cancer, bladder cancer, oral cancer
ATM	6	breast cancer, ovarian cancer, oral cancer, cervical cancer, thyroid carcinoma, pancreatic ductal adenocarcinoma
BRCA1	6	breast cancer, ovarian cancer, colorectal cancer, esophageal cancer, prostate cancer, myeloid neoplasms
MIR196A2	6	breast cancer, head and neck squamous cell carcinoma, hepatocellular carcinoma, colorectal cancer, liver cancer, gastric cancer
APEX1	6	breast cancer, bladder cancer, lung cancer, colorectal cancer, ovarian cancer, prostate cancer
NF1	6	brain tumors, peripheral nerve tumor, neurofibromas, pilocytic astrocytomas, schwannomas, leukaemia
GPC3	5	wilms tumor, hepatoblastoma, hepatocellular carcinoma, neuroblastoma, gonadoblastoma
HRAS	5	thyroid cancer, gastric cancer, rhabdomyosarcoma, neuroblastoma, transitional cell carcinoma
BAP1	5	renal cell carcinoma, uveal melanoma, cutaneous melanoma, cholangiocarcinoma and breast carcinoma, malignant mesothelioma
MEN1	5	parathyroid, pituitary adenoma, neuroendocrine tumor, carcinoid tumor, adrenocortical carcinoma
VDR	5	ovarian cancer, prostate cancer, oral squamous cell carcinoma, breast cancer, gallbladder adenocarcinoma
CASP9	5	lung cancer, pancreatic cancer, colorectal cancer, papillary thyroid carcinoma, breast cancer
CASP8	5	lung cancer, pancreatic cancer, breast cancer, bladder cancer, colorectal cancer
LAPTM4B	5	liver cancer, cervical cancer, ovarian cancer, breast cancer,
NQO1	5	esophageal cancer, bladder cancer, lung cancer, prostate cancer, breast cancer
HLA-G	5	cervical cancer, transitional cell carcinoma, hepatocellular carcinoma, breast carcinoma, cervical squamous cell carcinoma
CXCL12	5	breast cancer, lung cancer, squamous cell carcinoma of the head and neck, hepatocellular carcinoma, oral cancer
TSC1	4	renal cell cancer, angiomyolipoma, subependymal giant cell astrocytoma, rhabdomyoma
TSC2	4	renal cell cancer, angiomyolipoma, subependymal giant cell astrocytoma, rhabdomyoma
HIF1A	4	prostate cancer, renal cell carcinoma, breast cancer, hepatocellular carcinoma
CAV1	4	prostate cancer, gastric cancer, nasopharyngeal carcinoma, renal cell carcinoma

BRIP1	4	myeloid hematological malignancy, squamous cell carcinoma, breast cancer, ovarian cancer
PALB2	4	melanoma breast cancer, male breast cancer, ovarian cancer, pancreatic cancer
BLM	4	lymphoma, myeloid hematological malignancy, squamous cell carcinoma,colorectal cancer
FAS	4	lung cancer, gastric cancer, laryngeal and hypopharyngeal squamous cell carcinoma, breast cancer
CDH1	4	lobular breast, colorectal cancer, gastric cancer, prostate cancer
XRCC6	4	hepatocellular carcinoma, breast cancer, gastric cancer, lung cancer
TLR2	4	gallbladder cancer, gastric cancer, cervical cancer, breast cancer
PLCE1	4	gallbladder cancer, esophageal cancer, gastric adenocarcinoma, colorectal cancer
TLR4	4	gallbladder cancer, breast cancer, cervical cancer, gastric cancer
XRCC2	4	esophageal squamous cell carcinoma, gastric cardiac adenocarcinoma, breast cancer, colorectal cancer
STK11	4	colorectal cancer, gastric cancer, breast cancer, sex cord stromal tumor
APC	4	colorectal cancer, desmoid tumour, breast cancer, hepatoblastoma
TNF	4	cervical cancer, non small cell lung cancer, oral squamous cell carcinoma, prostate cancer
IFNG	4	cervical cancer, lung cancer, nasopharyngeal carcinoma, hepatocellular carcinoma
BCL2	4	breast cancer, esophageal cancer, prostate cancer, papillary thyroid carcinoma
CYP1B1	4	breast cancer, colorectal cancer, prostate cancer, lung cancer
BUB1B	3	wilms tumor, rhabdomyosarcoma, myeloid hematological malignancy
SUFU	3	sonic hedgehog tumor, medulloblastoma, meningioma
WRN	3	sarcoma, melanoma, thyroid cancer
VHL	3	retinal and CNS haemangioblastomas, pheochromocytomas, renal clear cell carcinomas
GNMT	3	prostate cancer, liver cancer, hepatocellular carcinoma
SDHB	3	paraganglioma, pheochromocytoma, renal cell cancer
SDHA	3	paraganglioma, pheochromocytoma, gastrointestinal stromal tumor
SDHC	3	paraganglioma, pheochromocytoma, gastrointestinal stromal tumor
SDHD	3	paraganglioma, pheochromocytoma, gastrointestinal stromal tumor
SELE	3	pancreatic cancer, breast cancer, gastric cancer
TP73	3	ovarian cancer, nasopharyngeal carcinoma, lung cancer
HLA-DQA1	3	ovarian cancer, gastric cancer, hepatocellular carcinoma
LOX	3	ovarian cancer, gastric cancer, cervical cancer
RAD51C	3	ovarian cancer, breast cancer, head and neck cancer
RECQL4	3	osteosarcoma, basal cell carcinoma, squamous cell carcinoma
CCR2	3	oral cancer,hepatocellular carcinoma,prostate cancer
RMRP	3	non-hodgkin lymphoma, squamous carcinoma, leukemia
ALK	3	neuroblastoma, ganglioneuroblastoma, ganglioneuroma
IL10	3	nasopharyngeal carcinoma,lung cancer, gastric cancer
GBA	3	myeloma, lymphoma, hepatocellular carcinoma
CDKN1B	3	multiple endocrine neoplasia,thyroid cancer, Pituitary adenoma
NF2	3	meningiomas and schwannomas, nervous system tumors, spinal cord glial tumor
CDKN2A	3	melanoma, pancreas cancer, astrocytoma
FASLG	3	lung cancer, pancreatic cancer, breast cancer
ADIPOQ	3	lung cancer, hepatocellular carcinoma, gastric cancer
DNMT3B	3	lung cancer, colorectal cancer, gallbladder cancer
ERBB2	3	lung cancer, breast cancer, ovarian carcinoma

ERCC6	3	lung cancer, bladder cancer, prostate cancer
FH	3	leiomyomatosis, renal cell cancer, breast cancer
AR	3	hepatocellular carcinoma, prostate cancer, prostate cancer
MIR499A	3	gastrointestinal cancer, hepatocellular carcinoma, breast cancer
SPP1	3	gastric cancer,oral squamous cell carcinoma, cervical cancer
IGFBP3	3	gastric cancer, squamous cell esophageal cancer, prostate cancer
HAVCR2	3	gastric cancer, lung cancer, pancreatic cancer
IL17A	3	gastric cancer, colorectal cancer, non small cell lung cancer
HLA-DQB1	3	gastric cancer, cervical cancer, hepatocellular carcinoma
GPX1	3	gastric cancer, bladder cancer, prostate cancer
PSCA	3	gastric cancer, bladder cancer, breast cancer
MEFV	3	familial mediterranean fever, acute myeloid leukemia, myelodysplastic syndrome
MMP7	3	endometrial cancer, gallbladder cancer, breast cancer
CYLD	3	cylindroma, spiroadenocarcinoma, basal cell carcinoma
TNFRSF10A	3	colorectal cancer, lung cancer, gallbladder carcinoma
CXCL8	3	colorectal cancer, gastric cardiac adenocarcinoma, bladder carcinoma
BMPR1A	3	colorectal cancer, gastric cancer, hamartoma
MUTYH	3	colorectal cancer, endometrial cancer, head neck cancer
CD28	3	cervical cancer, lung cancer, cervical cancer
CHEK2	3	breast cancer, prostate cancer, lung cancer
MTRR	3	breast cancer, pancreatic cancer, gastric cancer
MCPH1	3	breast cancer, ovarian cancer,
ESR1	3	breast cancer, endometrial cancer, hepatocellular carcinoma
TGFBR1	3	breast and ovarian cancer, colorectal cancer, hepatocellular carcinoma
DDB2	3	basal cell carcinoma, squamous cell carcinoma, melanoma
ERCC2	3	basal cell carcinoma, squamous cell carcinoma, melanoma
ERCC3	3	basal cell carcinoma, squamous cell carcinoma, melanoma
ERCC4	3	basal cell carcinoma, squamous cell carcinoma, melanoma
ERCC5	3	basal cell carcinoma, squamous cell carcinoma, melanoma
XPA	3	basal cell carcinoma, squamous cell carcinoma, melanoma
XPC	3	basal cell carcinoma, squamous cell carcinoma, melanoma
TERT	3	acute myelogenous leukemia, testicular germ cell cancer, lung cancer
PRKAR1A	2	thyroid cancer, sex cord stromal tumor
XRCC5	2	thyroid cancer, hepatocellular carcinoma
XRCC4	2	thyroid cancer, colorectal cancer
DOCK8	2	squamous cell carcinoma, lymphoma
PTCH1	2	sonic hedgehog tumor, medulloblastomas
IRF4	2	skin cancer, haematological malignancies
SMARCB1	2	rhabdoid tumor, central primitive neuroectodermal tumor
RB1	2	retinoblastoma, breast cancer
TMEM127	2	renal cell carcinomas, pheochromocytoma
FLCN	2	renal cell cancer, oncocytoma
NKX2-1	2	pulmonary carcinoma, thyroid cancer
CCL5	2	prostate cancer, hepatocellular carcinoma
MYC	2	prostate cancer, colorectal cancer
CYP17A1	2	prostate cancer, breast cancer
CYP19A1	2	prostate cancer, breast cancer

KIF1B	2	pheochromocytoma, neuroblastoma
CDC73	2	parathyroid cancer, ossifying fibroma
MAX	2	paraganglioma, pheochromocytoma
SDHAF2	2	paraganglioma, pheochromocytoma
PTPRJ	2	papillary thyroid carcinoma, colorectal cancer
CDKN1A	2	pancreatic cancer, squamous cell carcinoma of the head and neck
IL23R	2	ovarian cancer, gastric cancer
NFKB1	2	ovarian cancer, colorectal cancer
RAD51D	2	ovarian cancer, breast cancer
KRAS	2	oral squamous cell carcinoma, lung tumor
MICA	2	oral squamous cell carcinoma, cervical cancer
ICAM1	2	oral cancer, urothelial cell carcinoma
ACE	2	oral cancer, prostate cancer
RHBDF2	2	oesophageal cancer, tylosis associated cancer
IL4	2	non small cell lung cancer, renal cell carcinoma
CCL2	2	non small cell lung cancer, ovarian cancer
AGER	2	non small cell lung cancer, epithelial ovarian carcinoma
BRAF	2	non medullary thyroid cancer, colorectal carcinogenesis
CCNE1	2	nasopharyngeal carcinoma, ovarian cancer
TLR3	2	nasopharyngeal carcinoma, cervical cancer
FANCA	2	myeloid hematological malignancy, squamous cell carcinoma
FANCC	2	myeloid hematological malignancy, squamous cell carcinoma
FANCG	2	myeloid hematological malignancy, squamous cell carcinoma
CEBPA	2	myeloid hematological malignancy, leukemia
FOXE1	2	multiple self healing squamous epitheliomata, thyroid cancer
RET	2	medullary thyroid cancer, pheochromocytoma
RASSF1	2	lung cancer, hepatocellular carcinoma
NME1	2	lung cancer, endometrial cancer
AXIN2	2	lung cancer, colon cancer
PRDM2	2	liver cancer, breast cancer
SEPT9	2	leukemia, ovarian carcinoma
EZH2	2	hepatocellular carcinoma, esophageal squamous cell carcinoma
HFE	2	hepatocellular carcinoma, cholangiocarcinoma
ABCB11	2	hepatocellular carcinoma, cholangiocarcinoma
SERPINE1	2	hepatocellular carcinoma, cervical cancer
UGT1A7	2	hepatocellular carcinoma, bladder cancer
KCNQ1	2	gastrointestinal cancer, colorectal cancer
MIR27A	2	gastric cancer,breast cancer
MPO	2	gastric cancer, lung cancer
MUC1	2	gastric cancer, lung adenocarcinoma
NSD1	2	gastric cancer, colorectal cancer
GSPT1	2	gastric cancer, breast cancer
TYMS	2	gastric cancer, breast cancer
SOD2	2	esophageal squamous cell carcinoma,gastric cancer
HLA-B	2	esophageal carcinoma, papillary thyroid carcinoma
HLA-A	2	esophageal carcinoma, nasopharyngeal carcinoma
DLEC1	2	esophageal cancer, lung cancer

ESR2	2	epithelial ovarian cancer, breast cancer
MET	2	epithelial cancer, renal cell cancer
CASP7	2	endometrial cancer, gastric adenocarcinoma
CD14	2	colorectal cancer, prostate cancer
IL18	2	colorectal cancer, oral cancer
CSE1L	2	colorectal cancer, hepatocellular carcinoma
IGF2	2	colorectal cancer, hepatocellular carcinoma
ABCG8	2	colorectal cancer, gastric cancer
ALOX12	2	colorectal cancer, esophageal squamous cell carcinoma
POLD1	2	colorectal cancer, endometrial cancer
MLH3	2	colorectal cancer, endometrial cancer
ENO1	2	colon cancer, breast cancer
EGF	2	cervical cancer, prostate cancer
TLR9	2	cervical cancer, gastric carcinoma
IL1B	2	cervical cancer, gastric cancer
IL1RN	2	cervical cancer, gastric cancer
KLLN	2	breast cancer, renal cell carcinoma
FGFR4	2	breast cancer, prostate cancer
NOS3	2	breast cancer, prostate cancer
PPARGC1A	2	breast cancer, ovarian cancer
TOPBP1	2	breast cancer, ovarian cancer
IL13	2	breast cancer, glioma
COMT	2	breast cancer, esophageal squamous cell carcinomal
LTA	2	breast cancer, bladder cancer
WRAP53	2	breast cancer, acute myelogenous leukemia
RNASEL	2	breast and ovarian cancer, prostate cancer
IL12B	2	brain tumor, esophageal cancer
MTOR	2	bladder cancer, renal cell carcinoma
PRKDC	2	bladder cancer, prostate cancer
MMP1	2	bladder cancer, lung cancer
PARP1	2	bladder cancer, gastrointestinal cancer
DKC1	2	acute myeloid leukemia, squamous cell carcinoma
TRIM37	1	wilms tumor
POU6F2	1	wilms tumor
DIS3L2	1	wilms tumor
H19	1	wilms tumor
WT3	1	wilms tumor
WT4	1	wilms tumor
DEL11P13	1	wilms tumor
SLC14A1	1	urinary bladder cancer
FANCM	1	triple negative breast cancer
MFT2	1	trichoepithelioma
PCM1	1	thyroid carcinoma
TSHR	1	thyroid carcinoma
CCDC6	1	thyroid carcinoma
NCOA4	1	thyroid carcinoma
TRIM24	1	thyroid carcinoma

ATG5	1	thyroid carcinoma
MINPP1	1	thyroid carcinoma
GOLGA5	1	thyroid carcinoma
NDUFA13	1	thyroid carcinoma
TRIM33	1	thyroid carcinoma
TCO	1	thyroid carcinoma
PTCPRN	1	thyroid carcinoma
NMTC1	1	thyroid carcinoma
LIG4	1	thyroid cancer
IL32	1	thyroid cancer
KITLG	1	testicular germ cell tumors
SPRY4	1	testicular germ cell tumor
TGCT1	1	testicular germ cell tumor
DMRT1	1	testicular germ cell cancer
ATF7IP	1	testicular germ cell cancer
KRT17	1	steatocystoma
BAG1	1	squamous cell esophageal cancer
CASP3	1	squamous cell carcinoma of the head and neck
IGFBP5	1	squamous cell carcinoma of the head and neck
DEC1	1	squamous cell carcinoma of the head and neck
MIR885	1	squamous cell carcinoma of the head and neck
PTPN13	1	squamous cell carcinoma of head and neck
GJB2	1	squamous cell carcinoma
ING1	1	squamous cell carcinoma
TNFRSF10B	1	squamous cell carcinoma
RSPO1	1	squamous cell carcinoma
COL7A1	1	squamous cell carcinmona
STX1A	1	sporadic prostate cancer
SCLC1	1	small-cell cancer of lung
CDK4	1	skin tumor
MC1R	1	skin cancer
POLH	1	skin cancer
SERPINB4	1	skin cancer
LEF1	1	sebaceous tumor
SSX1	1	sarcoma
SSX2	1	sarcoma
FOXO1	1	rhabdomyosarcoma
PAX3	1	rhabdomyosarcoma
PAX7	1	rhabdomyosarcoma
SOS1	1	rhabdomyosarcoma
SMARCA4	1	rhabdoid tumor
PRCC	1	renal cell carcinoma
HNF1A	1	renal cell carcinoma
TFE3	1	renal cell carcinoma
RNF139	1	renal cell carcinoma
HAVCR1	1	renal cell carcinoma
DIRC2	1	renal cell carcinoma

ABCG2	1	renal cell cancer
HPCQTL19	1	prostate cancer
NAT1	1	prostate cancer
ZFHX3	1	prostate cancer
BMPR1B	1	prostate cancer
CDH13	1	prostate cancer
CCR5	1	prostate cancer
KLF6	1	prostate cancer
EPHB2	1	prostate cancer
GCNT1	1	prostate cancer
GNB3	1	prostate cancer
GSTA1	1	prostate cancer
HIP1	1	prostate cancer
HPN	1	prostate cancer
IGF1	1	prostate cancer
CYR61	1	prostate cancer
IL16	1	prostate cancer
IRS1	1	prostate cancer
ITGA2	1	prostate cancer
CD82	1	prostate cancer
KLK2	1	prostate cancer
LGALS3	1	prostate cancer
LHB	1	prostate cancer
MSMB	1	prostate cancer
MSR1	1	prostate cancer
MXI1	1	prostate cancer
NKX3-1	1	prostate cancer
POU5F1	1	prostate cancer
POU5F1B	1	prostate cancer
PPP2R2A	1	prostate cancer
HNF1B	1	prostate cancer
UGT2B17	1	prostate cancer
FGF23	1	prostate cancer
CPNE3	1	prostate cancer
GDF15	1	prostate cancer
KLK4	1	prostate cancer
HOXB13	1	prostate cancer
POLI	1	prostate cancer
EHBP1	1	prostate cancer
IRX4	1	prostate cancer
PDE11A	1	prostate cancer
BTNL2	1	prostate cancer
ELAC2	1	prostate cancer
RINT1	1	prostate cancer
ESCO1	1	prostate cancer
ARL11	1	prostate cancer
RFX6	1	prostate cancer

FMN1	1	prostate cancer
PCAP	1	prostate cancer
HPCX	1	prostate cancer
HPC3	1	prostate cancer
HPC4	1	prostate cancer
HPC5	1	prostate cancer
HPCX2	1	prostate cancer
HPC6	1	prostate cancer
HPC7	1	prostate cancer
HPC9	1	prostate cancer
HPC10	1	prostate cancer
HPC14	1	prostate cancer
HPC15	1	prostate cancer
RAD54B	1	primary lymphoma and colorectal cancer
PRKCA	1	pituitary tumor
AIP	1	pituitary adenoma
GPR101	1	pituitary adenoma
GNAI2	1	pituitary ACTH secreting adenoma
MDH2	1	pheochromocytoma/paraganglioma
GDNF	1	pheochromocytoma
SRGAP1	1	papillary thyroid carcinoma
PTCSC3	1	papillary thyroid carcinoma
PAX8	1	papillary thyroid cancer
STK17B	1	papillary thyroid cancer
ST11	1	pancreatic endocrine tumors
ACVR1B	1	pancreatic cancer
PRSS1	1	pancreatic cancer
PALLD	1	pancreatic cancer
YTHDC2	1	pancreatic cancer
RRAS2	1	ovarian carcinoma
CDK2	1	ovarian cancer
OPCML	1	ovarian cancer
IL27	1	ovarian cancer
OVCAS1	1	ovarian cancer
LRP5	1	osteoporosis pseudoglioma syndrome
MMP14	1	oral squamous cell carcinoma
GSTM3	1	oral cancer
RECK	1	oral cancer
CP	1	oesophageal cancer
TSG11	1	nonsmall cell lung cancer
EGFR	1	non small cell lung cancer
PTGER4	1	non cardia gastric cancer
ZBTB20	1	non cardia gastric cancer
PTPN11	1	neuroblastoma
PHOX2B	1	neuroblastoma
NBLST4	1	neuroblastoma
NBLST5	1	neuroblastoma

NBLST6	1	neuroblastoma
CR2	1	nasopharyngeal carcinoma
HSPA1B	1	nasopharyngeal carcinoma
ITGA9	1	nasopharyngeal carcinoma
NPC1	1	nasopharyngeal carcinoma
CD209	1	nasopharyngeal carcinoma
MIR151A	1	nasopharyngeal carcinoma
LOC344967	1	nasopharyngeal carcinoma
NPCA2	1	nasopharyngeal carcinoma
DDIT3	1	myxoid liposarcoma
CALR	1	myeloproliferative neoplasms
JAK2	1	myeloproliferative neoplasms
CBFB	1	myeloid leukemia
GATA2	1	myeloid hematological malignancy
SBDS	1	myeloid hematological malignancy
ACSL6	1	myelogenous leukemia
MLSM7	1	myelodysplasia and leukemia syndrome
CRTC1	1	mucoepidermoid salivary gland carcinoma
MAML2	1	mucoepidermoid salivary gland carcinoma
WT1	1	mesothelioma
MN1	1	meningioma
MNRI	1	meningioma
SMARCE1	1	mengingioma
CDKN2B	1	melanoma
MITF	1	melanoma
TYR	1	melanoma
POT1	1	melanoma
CMM	1	melanoma
UVM1	1	melanoma
UVM2	1	melanoma
CMM4	1	melanoma
CMM7	1	melanoma
MKL1	1	megakaryoblastic leukemia
RBM15	1	megakaryoblastic leukemia
NTRK1	1	medullary thyroid carcinoma
MMS	1	malignant mesothelioma
MTAP	1	malignant fibrous histiocytoma (sarcoma)
MFHAS1	1	malignant fibrous histiocytoma
MGCT	1	male germ cell tumor
BCL10	1	male germ cell tumor
KDSR	1	lymphoma/leukemia
IRF1	1	lymphoma
SH2D1A	1	lymphoma
PRF1	1	lymphoma
WAS	1	lymphoma
RAP1GDS1	1	lymphocytic leukemia
BAX	1	lung cancer

CASP5	1	lung cancer
TNFSF8	1	lung cancer
CDK5	1	lung cancer
CHRNA3	1	lung cancer
CHRNA5	1	lung cancer
CHRNB3	1	lung cancer
CHRNB4	1	lung cancer
MAP3K8	1	lung cancer
CREB1	1	lung cancer
CYP2A6	1	lung cancer
EPHX1	1	lung cancer
ERCC1	1	lung cancer
FEN1	1	lung cancer
GPC5	1	lung cancer
GTF2H1	1	lung cancer
JUN	1	lung cancer
MAD2L1	1	lung cancer
MMP3	1	lung cancer
ABCC1	1	lung cancer
PIN1	1	lung cancer
PPP2R1B	1	lung cancer
PTGIS	1	lung cancer
REV3L	1	lung cancer
SMARCA2	1	lung cancer
SULT1A1	1	lung cancer
CXCR4	1	lung cancer
PRRC2A	1	lung cancer
MAD1L1	1	lung cancer
TP63	1	lung cancer
MBD4	1	lung cancer
CHRNA6	1	lung cancer
HDAC9	1	lung cancer
PDCD6IP	1	lung cancer
PDCD6	1	lung cancer
XRN2	1	lung cancer
RGS17	1	lung cancer
ICOS	1	lung cancer
SUV39H2	1	lung cancer
CLPTM1L	1	lung cancer
C18orf54	1	lung cancer
LIN28B	1	lung cancer
LNCR1	1	lung cancer
LNCR3	1	lung cancer
LNCR4	1	lung cancer
LNCR5	1	lung cancer
LPSA	1	liposarcoma
LHCGR	1	leydig cell adenoma

BCL3	1	leukemia/lymphoma
BCL6	1	leukemia/lymphoma
TCL1A	1	leukemia/lymphoma
TCL1B	1	leukemia/lymphoma
TCL4	1	leukemia/lymphoma
ELANE	1	leukemia
ABL1	1	leukemia
ABL2	1	leukemia
ARNT	1	leukemia
BCR	1	leukemia
RUNX1	1	leukemia
ETV6	1	leukemia
FLT3	1	leukemia
LMO1	1	leukemia
LMO2	1	leukemia
LPP	1	leukemia
LYL1	1	leukemia
MLF1	1	leukemia
KMT2A	1	leukemia
MYB	1	leukemia
NPM1	1	leukemia
NUMA1	1	leukemia
PAX5	1	leukemia
PBX1	1	leukemia
PML	1	leukemia
RARA	1	leukemia
SH3GL1	1	leukemia
STAT5B	1	leukemia
TAL1	1	leukemia
TAL2	1	leukemia
TCF3	1	leukemia
ZBTB16	1	leukemia
DEK	1	leukemia
NUP214	1	leukemia
PICALM	1	leukemia
GMPS	1	leukemia
IKZF1	1	leukemia
MLLT11	1	leukemia
ARHGAP26	1	leukemia
ARHGEF12	1	leukemia
CHIC2	1	leukemia
WHSC1L1	1	leukemia
LALL	1	leukemia
CLLS2	1	leukemia
TAM	1	leukemia
CLLS1	1	leukemia
CLLS3	1	leukemia

CLLS4	1	leukemia
CLLS5	1	leukemia
ALL1	1	leukemia
ALL2	1	leukemia
HMGA2	1	leiomyoma
ITS	1	insulinoma
ITK	1	hodgkins lymphoma
KLHDC8B	1	hodgkin lymphoma
MAGEC1	1	hereditary prostate cancer
CDH17	1	hepatocellular carcinoma
EPHB1	1	hepatocellular carcinoma
FAH	1	hepatocellular carcinoma
HMBS	1	hepatocellular carcinoma
IGF2R	1	hepatocellular carcinoma
LEPR	1	hepatocellular carcinoma
SERPINA1	1	hepatocellular carcinoma
SPARC	1	hepatocellular carcinoma
STAT1	1	hepatocellular carcinoma
STAT4	1	hepatocellular carcinoma
TGFBR2	1	hepatocellular carcinoma
TGFBR3	1	hepatocellular carcinoma
TIMP3	1	hepatocellular carcinoma
TIMP4	1	hepatocellular carcinoma
UROD	1	hepatocellular carcinoma
VEGFC	1	hepatocellular carcinoma
AXIN1	1	hepatocellular carcinoma
EXO1	1	hepatocellular carcinoma
APOBEC3B	1	hepatocellular carcinoma
SLC25A13	1	hepatocellular carcinoma
DLC1	1	hepatocellular carcinoma
HTATIP2	1	hepatocellular carcinoma
UGT1A10	1	hepatocellular carcinoma
IFNL3	1	hepatocellular carcinoma
HCP5	1	hepatocellular carcinoma
MIR378A	1	hepatocellular carcinoma
FLT4	1	hemangioma
KDR	1	hemangioma
ANTXR1	1	hemangioma
ATP2A2	1	head and neck squamous cell carcinoma
ATP2A3	1	head and neck squamous cell carcinoma
CYP2D6	1	head and neck cancer
SRY	1	gonadoblastoma
IDH1	1	glioma
GLM4	1	glioma
GLM8	1	glioma
GLM5	1	glioma
GLM6	1	glioma

GLM7	1	glioma
KIT	1	gastro intestinal stromal tumor
PDGFRA	1	gastro intestinal stromal tumor
IL15RA	1	gastric cardiac adenocarcinoma
IL37	1	gastric cardiac adenocarcinoma
ADD1	1	gastric cancer
ALDH2	1	gastric cancer
RUNX3	1	gastric cancer
DNMT3A	1	gastric cancer
FOLR1	1	gastric cancer
HMOX1	1	gastric cancer
IL7	1	gastric cancer
MIF	1	gastric cancer
MUC5AC	1	gastric cancer
MYCL	1	gastric cancer
NOS2	1	gastric cancer
PGC	1	gastric cancer
PRKAA1	1	gastric cancer
PTPRCAP	1	gastric cancer
HSPA13	1	gastric cancer
TNFAIP2	1	gastric cancer
TP53BP2	1	gastric cancer
TXNRD2	1	gastric cancer
CD226	1	gastric cancer
EGLN2	1	gastric cancer
DAB2IP	1	gastric cancer
IRGM	1	gastric cancer
MIR184	1	gastric cancer
MIR200C	1	gastric cancer
MIR181A1	1	gastric cancer
MIR938	1	gastric cancer
ADRB3	1	gallbladder cancer and gallstone disease
CR1	1	gallbladder cancer
DCC	1	gallbladder cancer
MMP9	1	gallbladder cancer
TIMP2	1	gallbladder cancer
NR1H2	1	gallbladder cancer
PEG10	1	gallbladder cancer
NOC3L	1	gallbladder cancer
FL1	1	follicular lymphoma
EWSR1	1	ewing sarcoma
ADH1B	1	esophageal squamous cell carcinoma
ADH1C	1	esophageal squamous cell carcinoma
ADH7	1	esophageal squamous cell carcinoma
TNFRSF6B	1	esophageal squamous cell carcinoma
LZTS1	1	esophageal squamous cell carcinoma
WWOX	1	esophageal squamous cell carcinoma

ERAS	1	esophageal neoplasm
FOXF1	1	esophageal carcinoma
RNF6	1	esophageal carcinoma
IL12RB1	1	esophageal cancer
SLC11A1	1	esophageal cancer
FAT4	1	esophageal cancer
SPINK7	1	esophageal cancer
MSH3	1	endometrial carcinoma
TET2	1	endometrial cancer
TMC8	1	ecrvix cancer
PDGFB	1	dermatofibrosarcoma
POLE	1	colorectal cancer
BMP2	1	colorectal cancer
BMP4	1	colorectal cancer
BUB1	1	colorectal cancer
CD80	1	colorectal cancer
CD86	1	colorectal cancer
CRP	1	colorectal cancer
CTNNB1	1	colorectal cancer
CYP1A2	1	colorectal cancer
EFNA1	1	colorectal cancer
EP300	1	colorectal cancer
RPSA	1	colorectal cancer
LBP	1	colorectal cancer
EPCAM	1	colorectal cancer
SMAD4	1	colorectal cancer
SMAD7	1	colorectal cancer
MBL2	1	colorectal cancer
MCC	1	colorectal cancer
NRAS	1	colorectal cancer
ODC1	1	colorectal cancer
PCK2	1	colorectal cancer
PLA2G2A	1	colorectal cancer
PLD2	1	colorectal cancer
RBBP8	1	colorectal cancer
RPS20	1	colorectal cancer
SRC	1	colorectal cancer
TCN2	1	colorectal cancer
GREM1	1	colorectal cancer
CHFR	1	colorectal cancer
GALNT12	1	colorectal cancer
COLCA2	1	colorectal cancer
COLCA1	1	colorectal cancer
MIR149	1	colorectal cancer
MIR196A1	1	colorectal cancer
CRCS6	1	colorectal cancer
CRCS7	1	colorectal cancer

CRCS2	1	colorectal cancer
CRCS5	1	colorectal cancer
CRCS8	1	colorectal cancer
CRCS11	1	colorectal cancer
CRCS9	1	colorectal cancer
DES	1	colon cancer
PTPN12	1	colon cancer
CHDM	1	chordoma
EXT1	1	chondrosarcoma
EXT2	1	chondrosarcoma
NR4A3	1	chondrosarcoma
TAF15	1	chondrosarcoma
TMC6	1	cervix cancer
MGMT	1	cervical carcinoma
ST3	1	cervical carcinoma
DEFB4A	1	cervical cancer
HLA-DPB1	1	cervical cancer
HLA-DRB1	1	cervical cancer
IFNA17	1	cervical cancer
STAT3	1	cervical cancer
CD83	1	cervical cancer
LZTS2	1	carcinogen induced tumor
PON1	1	breast neoplasms
PDCD1	1	breast carcinoma
PGR	1	breast carcinoma
ACACA	1	breast cancer
ATR	1	breast cancer
BACH1	1	breast cancer
BARD1	1	breast cancer
CASP10	1	breast cancer
CTBP1	1	breast cancer
CYP11A1	1	breast cancer
GADD45A	1	breast cancer
ESRRG	1	breast cancer
FGFR2	1	breast cancer
FLT1	1	breast cancer
HMMR	1	breast cancer
HSD17B2	1	breast cancer
IL2	1	breast cancer
MAP3K1	1	breast cancer
MRE11A	1	breast cancer
MTNR1A	1	breast cancer
MTNR1B	1	breast cancer
MTR	1	breast cancer
NEK2	1	breast cancer
NQO2	1	breast cancer
YBX1	1	breast cancer

PHB	1	breast cancer
PIK3CA	1	breast cancer
RAD51	1	breast cancer
SHBG	1	breast cancer
ICAM5	1	breast cancer
TWIST1	1	breast cancer
NCOA3	1	breast cancer
RAD54L	1	breast cancer
PPM1D	1	breast cancer
IQGAP1	1	breast cancer
PER2	1	breast cancer
MBD2	1	breast cancer
THEMIS2	1	breast cancer
CLOCK	1	breast cancer
RB1CC1	1	breast cancer
RAD50	1	breast cancer
MORF4L1	1	breast cancer
PSIP1	1	breast cancer
AKAP13	1	breast cancer
FAN1	1	breast cancer
RRP1B	1	breast cancer
FBXO10	1	breast cancer
TOX3	1	breast cancer
UGT1A1	1	breast cancer
ZNF350	1	breast cancer
NOD2	1	breast cancer
ATG10	1	breast cancer
FAM175A	1	breast cancer
CNTROB	1	breast cancer
PPARGC1B	1	breast cancer
MIER3	1	breast cancer
BRCATA	1	breast cancer
BCPR	1	breast cancer
BRCA3	1	breast cancer
IL12A	1	brain tumor
AKT1	1	bladder cancer
FGFR3	1	bladder cancer
HSD3B2	1	bladder cancer
THBS1	1	bladder cancer
ARL6IP5	1	bladder cancer
CYP2C19	1	biliary tract cancer
GAB1	1	biliary tract cancer
RASA1	1	basal cell carcinoma
TGM3	1	basal cell carcinoma
PTCH2	1	basal cell carcinoma
RGS22	1	basal cell carcinoma
BCC1	1	basal cell carcinoma

BCC2	1	basal cell carcinoma
BCC3	1	basal cell carcinoma
BCC4	1	basal cell carcinoma
BCC5	1	basal cell carcinoma
BCC6	1	basal cell carcinoma
ASCC1	1	barrett esophagus/esophageal adenocarcinoma
CTHRC1	1	barrett esophagus/esophageal adenocarcinoma
BCL7A	1	B cell non hodgkin lymphoma
ANC	1	anal canal carcinoma
ASPSCR1	1	alveolar soft part sarcoma
GATA1	1	acute myelogenous leukemia
TINF2	1	acute myelogenous leukemia
NOP10	1	acute myelogenous leukemia
NHP2	1	acute myelogenous leukemia
CTC1	1	acute myelogenous leukemia
TERC	1	acute myelogenous leukemia
PHF6	1	acute lymphoblastic leukemia

Supplementary Table S9: The information of 57 human CPGs act as training gene set

Gene ID	Official symbol	Official full name	Number of literature evidences
672	BRCA1	breast cancer 1, early onset	169
675	BRCA2	breast cancer 2, early onset	96
7157	TP53	tumor protein p53	85
11200	CHEK2	checkpoint kinase 2	40
79728	PALB2	partner and localizer of BRCA2	39
4436	MSH2	mutS homolog 2	38
4292	MLH1	mutL homolog 1	37
7515	XRCC1	X-ray repair complementing defective repair in Chinese hamster cells 1	37
1543	CYP1A1	cytochrome P450, family 1, subfamily A, polypeptide 1	35
4763	NF1	neurofibromin 1	33
472	ATM	ATM serine/threonine kinase	29
2944	GSTM1	glutathione S-transferase mu 1	29
5728	PTEN	phosphatase and tensin homolog	27
324	APC	adenomatous polyposis coli	26
999	CDH1	cadherin 1, type 1, E-cadherin (epithelial)	25
2956	MSH6	mutS homolog 6	25
5889	RAD51C	RAD51 paralog C	22
6790	AURKA	aurora kinase A	21
60528	ELAC2	elaC ribonuclease Z 2	21
4524	MTHFR	methylenetetrahydrofolate reductase (NAD(P)H)	21
5395	PMS2	PMS2 postmeiotic segregation increased 2 (S. cerevisiae)	20
2068	ERCC2	excision repair cross-complementation group 2	18
2952	GSTT1	glutathione S-transferase theta 1	18
5743	PTGS2	prostaglandin-endoperoxide synthase 2 (prostaglandin G/H synthase and cyclooxygenase)	17
4221	MEN1	multiple endocrine neoplasia I	16

2950	GSTP1	glutathione S-transferase pi 1	15
5925	RB1	retinoblastoma 1	15
6794	STK11	serine/threonine kinase 11	15
7422	VEGFA	vascular endothelial growth factor A	15
83990	BRIP1	BRCA1 interacting protein C-terminal helicase 1	14
595	CCND1	cyclin D1	14
4595	MUTYH	mutY homolog	14
4683	NBN	nibrin	14
1493	CTLA4	cytotoxic T-lymphocyte-associated protein 4	13
4191	MDH2	malate dehydrogenase 2, NAD (mitochondrial)	13
4771	NF2	neurofibromin 2 (merlin)	13
7040	TGFB1	transforming growth factor, beta 1	13
1029	CDKN2A	cyclin-dependent kinase inhibitor 2A	12
2271	FH	fumarate hydratase	12
4968	OGG1	8-oxoguanine DNA glycosylase	12
7015	TERT	telomerase reverse transcriptase	12
7251	TSG101	tumor susceptibility 101	12
1027	CDKN1B	cyclin-dependent kinase inhibitor 1B (p27, Kip1)	11
2099	ESR1	estrogen receptor 1	11
6041	RNASEL	ribonuclease L (2',5'-oligoisoadenylate synthetase-dependent)	11
6598	SMARCB1	SWI/SNF related, matrix associated, actin dependent regulator of chromatin, subfamily b, member 1	11
7124	TNF	tumor necrosis factor	11
7428	VHL	von Hippel-Lindau tumor suppressor, E3 ubiquitin protein ligase	11
7517	XRCC3	X-ray repair complementing defective repair in Chinese hamster cells 3	11
5243	ABCB1	ATP-binding cassette, sub-family B (MDR/TAP), member 1	10
580	BARD1	BRCA1 associated RING domain 1	10
332	BIRC5	baculoviral IAP repeat containing 5	10
1571	CYP2E1	cytochrome P450, family 2, subfamily E, polypeptide 1	10
2263	FGFR2	fibroblast growth factor receptor 2	10
3091	HIF1A	hypoxia inducible factor 1, alpha subunit (basic helix-loop-helix transcription factor)	10
10	NAT2	N-acetyltransferase 2 (arylamine N-acetyltransferase)	10
5979	RET	ret proto-oncogene	10

Supplementary Table S10: Gene ranking result of the 667 human CPGs

Supplementary Table S11: The significantly enriched pathway annotations in the 100 CPGs

Annotation source	Functional term	Raw *P*-value	Benjamini-Hochberg adjusted *P*-value
Reactome	DNA Repair	9.68E-16	1.57E-12
Reactome	Double-Strand Break Repair	3.98E-11	1.85E-08
Reactome	Homologous Recombination Repair	1.22E-09	4.40E-07
Reactome	Homologous recombination repair of replication-independent double-strand breaks	1.22E-09	4.40E-07
KEGG PATHWAY	Colorectal cancer	1.65E-09	4.76E-07
KEGG PATHWAY	Pathways in cancer	1.81E-09	4.90E-07
KEGG PATHWAY	Prostate cancer	6.56E-09	1.29E-06
KEGG PATHWAY	Pancreatic cancer	2.21E-08	3.34E-06
PANTHER	p53 pathway	2.41E-07	2.41E-05
KEGG PATHWAY	Hepatitis B	4.41E-07	4.10E-05
PANTHER	p53 pathway feedback loops 2	5.62E-07	4.94E-05
Reactome	Cellular responses to stress	1.61E-06	0.000120069
Reactome	Recruitment of repair and signaling proteins to double-strand breaks	3.69E-06	0.000223851
Reactome	Mismatch Repair	3.73E-06	0.000223851
Reactome	Meiotic recombination	3.93E-06	0.000232242
KEGG PATHWAY	Bladder cancer	5.25E-06	0.000299361
BioCarta	cell cycle: g1/s check point	7.07E-06	0.000383554
PID	BARD1 signaling events	7.58E-06	0.000407784
KEGG PATHWAY	Chronic myeloid leukemia	9.14E-06	0.000461027
Reactome	Meiosis	1.00E-05	0.000499114
Reactome	DNA Damage/Telomere Stress Induced Senescence	1.04E-05	0.000513673
KEGG PATHWAY	Endometrial cancer	1.08E-05	0.000526613
Reactome	Resolution of AP sites via the single-nucleotide replacement pathway	1.66E-05	0.000745271
KEGG PATHWAY	FoxO signaling pathway	2.38E-05	0.000984359
PANTHER	Hypoxia response via HIF activation	2.71E-05	0.001101972
KEGG PATHWAY	Cell cycle	3.40E-05	0.001340011
Reactome	Mismatch repair (MMR) directed by MSH2:MSH6 (MutSalpha)	3.96E-05	0.001513961
Reactome	Mismatch repair (MMR) directed by MSH2:MSH3 (MutSbeta)	3.96E-05	0.001513961
KEGG PATHWAY	Central carbon metabolism in cancer	8.50E-05	0.002696239
KEGG PATHWAY	p53 signaling pathway	9.56E-05	0.002948094
Reactome	Displacement of DNA glycosylase by APE1	0.000101127	0.003034384
Reactome	Regulation of gene expression by Hypoxia-inducible Factor	0.000101127	0.003034384
Reactome	Resolution of Abasic Sites (AP sites)	0.000101473	0.003034384
Reactome	Base Excision Repair	0.000101473	0.003034384
Reactome	Base-free sugar-phosphate removal via the single-nucleotide replacement pathway	0.000139317	0.003857014
PID	Regulation of Telomerase	0.000145747	0.004000959
BioCarta	role of brca1 brca2 and atr in cancer susceptibility	0.000153565	0.004133918
KEGG PATHWAY	Adherens junction	0.000167657	0.00441609
KEGG PATHWAY	Homologous recombination	0.000182151	0.004620693

Reactome	Constitutive PI3K/AKT Signaling in Cancer	0.00023219	0.005553777
Reactome	Fanconi Anemia pathway	0.000260815	0.006103821
KEGG PATHWAY	Glioma	0.00028313	0.006440701
KEGG PATHWAY	Fanconi anemia pathway	0.000296422	0.006673076
Reactome	Regulation of Hypoxia-inducible Factor (HIF) by oxygen	0.000307806	0.006765488
Reactome	Cellular response to hypoxia	0.000307806	0.006765488
KEGG PATHWAY	Renal cell carcinoma	0.000315928	0.006897411
Reactome	LRR FLII-interacting protein 1 (LRRFIP1) activates type I IFN production	0.000422601	0.008618936
Reactome	ATM mediated response to DNA double-strand break	0.000422601	0.008618936
Reactome	ATM mediated phosphorylation of repair proteins	0.000422601	0.008618936
KEGG PATHWAY	Mismatch repair	0.000436519	0.008847329
KEGG PATHWAY	HTLV-I infection	0.000453497	0.008940762
KEGG PATHWAY	Melanoma	0.000531282	0.010077327
PID	FOXM1 transcription factor network	0.000559566	0.010521773
Reactome	Pre-NOTCH Transcription and Translation	0.000562313	0.010537648
Reactome	PI-3K cascade	0.000595057	0.010874836
Reactome	PIP3 activates AKT signaling	0.000595057	0.010874836
Reactome	PI3K events in ERBB2 signaling	0.000595057	0.010874836
Reactome	PI3K events in ERBB4 signaling	0.000595057	0.010874836
Reactome	PI3K/AKT Signaling in Cancer	0.000595057	0.010874836
Reactome	Cell Cycle	0.000714472	0.012495586
Reactome	GAB1 signalosome	0.000734973	0.012684012
Reactome	PI3K/AKT activation	0.000734973	0.012684012
Reactome	TGF-beta receptor signaling activates SMADs	0.000736944	0.012684012
Reactome	Removal of DNA patch containing abasic residue	0.000744461	0.012745963
Reactome	Resolution of AP sites via the multiple-nucleotide patch replacement pathway	0.000744461	0.012745963
Reactome	Cellular Senescence	0.000780726	0.013159077
BioCarta	ctcf: first multivalent nuclear factor	0.000784208	0.013183607
Reactome	Homologous DNA pairing and strand exchange	0.000878636	0.014045221
KEGG PATHWAY	HIF-1 signaling pathway	0.000916029	0.014535812
Reactome	Role of LAT2/NTAL/LAB on calcium mobilization	0.001025988	0.015383277
Reactome	Base-Excision Repair, AP Site Formation	0.001190048	0.016979067
BioCarta	tgf beta signaling pathway	0.001371825	0.018829308
BioCyc	glutathione-mediated detoxification	0.001506068	0.020244791
Reactome	Cellular response to heat stress	0.00167556	0.021715518
Reactome	p53-Dependent G1 DNA Damage Response	0.00168443	0.02174101
Reactome	p53-Dependent G1/S DNA damage checkpoint	0.00168443	0.02174101
KEGG PATHWAY	Non-small cell lung cancer	0.001734586	0.022032874
Reactome	G1 Phase	0.001859369	0.022911089
Reactome	Cyclin D associated events in G1	0.001859369	0.022911089
Reactome	G1/S DNA Damage Checkpoints	0.001979447	0.024161877
KEGG PATHWAY	Small cell lung cancer	0.001994363	0.024206849
KEGG PATHWAY	Non-homologous end-joining	0.002487153	0.028639682
KEGG PATHWAY	mTOR signaling pathway	0.00256291	0.029150856
Reactome	Downregulation of TGF-beta receptor signaling	0.002600152	0.029501124
Reactome	Signaling by FGFR in disease	0.002705951	0.030405725

Reactome	Signaling by ERBB4	0.002939003	0.031975173
BioCarta	p53 signaling pathway	0.003168346	0.034071498
Reactome	Pre-NOTCH Expression and Processing	0.003285527	0.034870534
Reactome	Cytosolic sensors of pathogen-associated DNA	0.003563267	0.036797802
Reactome	IRF3-mediated induction of type I IFN	0.003746599	0.038265891
Reactome	AKT phosphorylates targets in the cytosol	0.003746599	0.038265891
BioCarta	atm signaling pathway	0.003958122	0.039617759
BioCarta	pelp1 modulation of estrogen receptor activity	0.00403233	0.040298528
Reactome	Loss of Function of SMAD4 in Cancer	0.004350302	0.041806597
Reactome	SMAD4 MH2 Domain Mutants in Cancer	0.004350302	0.041806597
Reactome	TGFBR1 LBD Mutants in Cancer	0.004350302	0.041806597
Reactome	TGFBR2 MSI Frameshift Mutants in Cancer	0.004350302	0.041806597
Reactome	SMAD2/3 MH2 Domain Mutants in Cancer	0.004350302	0.041806597
Reactome	Loss of Function of SMAD2/3 in Cancer	0.004350302	0.041806597
Reactome	SMAD2/3 Phosphorylation Motif Mutants in Cancer	0.004350302	0.041806597
Reactome	TGFBR1 KD Mutants in Cancer	0.004350302	0.041806597
Reactome	Signaling by TGF-beta Receptor Complex	0.004350302	0.041806597
Reactome	Loss of Function of TGFBR1 in Cancer	0.004350302	0.041806597
Reactome	Loss of Function of TGFBR2 in Cancer	0.004350302	0.041806597
Reactome	Signaling by TGF-beta Receptor Complex in Cancer	0.004350302	0.041806597
Reactome	TGFBR2 Kinase Domain Mutants in Cancer	0.004350302	0.041806597
Reactome	Signaling by ERBB2	0.004938244	0.046178625
BioCarta	hypoxia and p53 in the cardiovascular system	0.004974553	0.046198572
BioCarta	telomeres telomerase cellular aging and immortality	0.005505041	0.049537757

Supplementary Table S12: The significantly enriched disease annotations in the 100 CPGs

Annotation source	Functional term	Raw *P*-value	Benjamini-Hochberg adjusted *P*-value
GAD	Breast cancer	5.75E-24	3.74E-20
GAD	Colorectal cancer	1.95E-18	6.33E-15
GAD	Prostate cancer	7.62E-16	1.57E-12
GAD	Stomach cancer	5.05E-15	6.57E-12
GAD	Lung cancer	3.49E-12	3.24E-09
GAD	Cancer	4.89E-12	3.97E-09
GAD	Bladder cancer	8.97E-12	5.84E-09
GAD	Ovarian cancer	3.70E-11	1.85E-08
GAD	Endometrial cancer	3.04E-09	7.06E-07
GAD	Esophageal cancer	5.41E-09	1.14E-06
GAD	Cytogenetic studies	1.38E-08	2.35E-06
GAD	Benzene toxicity	2.21E-07	2.29E-05
GAD	Endometriosis	2.25E-07	2.29E-05
GAD	Liver cancer	3.36E-07	3.26E-05
GAD	Overall effect	4.59E-07	4.20E-05
GAD	Leukemia	1.82E-06	0.000133201
GAD	Oral cancer	5.01E-06	0.000291292
GAD	Radiotherapy response	5.23E-06	0.000299361
FunDO	Oral cancer	9.61E-06	0.000480921

OMIM	Breast cancer	1.45E-05	0.000671085
GAD	Carcinoma, squamous cell	2.12E-05	0.000889698
NHGRI GWAS Catalog	Bladder cancer	2.41E-05	0.000991635
GAD	Cervical cancer	3.05E-05	0.001223559
GAD	Neoplasms	5.53E-05	0.001960903
GAD	Coke-oven toxicity	5.55E-05	0.001960903
GAD	Kidney cancer	6.13E-05	0.002110697
GAD	Adenocarcinoma	9.05E-05	0.002832213
GAD	Pancreatic cancer	0.00011025	0.003203291
GAD	Glioma	0.000139205	0.003857014
FunDO	Barrett's esophagus	0.000149817	0.00409543
FunDO	Endometriosis	0.000168868	0.00443006
NHGRI GWAS Catalog	Breast cancer	0.000222361	0.005350764
FunDO	Testicular dysfunction	0.000255764	0.006073002
GAD	Head and neck cancer	0.000292162	0.00662302
NHGRI GWAS Catalog	Glioma	0.000344287	0.007272503
NHGRI GWAS Catalog	Melanoma	0.000536977	0.010155725
GAD	Nasopharyngeal cancer	0.00069062	0.012151767
GAD	Multiple myeloma	0.00069062	0.012151767
GAD	Stomach neoplasms	0.000764695	0.013026739
FunDO	Peptic esophagitis	0.000766868	0.013026739
FunDO	Neck cancer	0.000766868	0.013026739
FunDO	Adenovirus infection	0.00088522	0.014081273
GAD	1-hydroxypyrene, urinary	0.000939205	0.014651667
GAD	Upper aerodigestive tract cancer	0.000939205	0.014651667
GAD	Non-hodgkin's lymphoma	0.000939205	0.014651667
GAD	Arsenic metabolism	0.000939205	0.014651667
GAD	Methotrexate toxicity	0.001381078	0.018916408
GAD	Infertility, male	0.001689815	0.02174101
GAD	Pneumonia	0.001934189	0.023653823
NHGRI GWAS Catalog	Basal cell carcinoma	0.002179726	0.025878285
GAD	Preterm delivery	0.002446255	0.0283191
GAD	Migraine disorders	0.002607308	0.029501124
GAD	Prostatic hyperplasia	0.002607308	0.029501124
GAD	Melanoma	0.002786775	0.030939865
GAD	Hepatitis b	0.002899748	0.031734971
GAD	Inflammatory bowel disease	0.003407492	0.035756681
NHGRI GWAS Catalog	Breast cancer (early onset)	0.003476922	0.036426497
FunDO	Hyperparathyroidism	0.003706326	0.038047948
GAD	Brain cancer	0.004344023	0.041806597
GAD	Reproduction	0.004590828	0.043396542
NHGRI GWAS Catalog	Serum prostate-specific antigen levels	0.004595791	0.043396542
FunDO	Fanconi's anemia	0.004940105	0.046178625
FunDO	Aseptic necrosis of bone	0.004975156	0.046198572
GAD	Bone mass	0.00542069	0.048981953
GAD	Gastric cancer	0.00542069	0.048981953

Supplementary Table S13: The significantly enriched Gene Ontology (GO) annotations in the 100 human CPGs

Annotation source	Functional term	Proportion of annotated genes to all 100 CPGs	Raw *P*-value	Benjamini-Hochberg adjusted *P*-value
GOTERM_BP_ALL	GO:0006974~response to DNA damage stimulus	34.34343434	1.13E-28	2.39E-25
GOTERM_BP_ALL	GO:0006281~DNA repair	30.3030303	6.78E-27	7.19E-24
GOTERM_BP_ALL	GO:0033554~cellular response to stress	37.37373737	2.31E-26	1.63E-23
GOTERM_BP_ALL	GO:0010941~regulation of cell death	41.41414141	3.09E-25	8.19E-23
GOTERM_BP_ALL	GO:0006950~response to stress	54.54545455	2.75E-25	8.34E-23
GOTERM_BP_ALL	GO:0051716~cellular response to stimulus	41.41414141	3.89E-25	9.17E-23
GOTERM_BP_ALL	GO:0043067~regulation of programmed cell death	41.41414141	2.69E-25	9.51E-23
GOTERM_BP_ALL	GO:0042981~regulation of apoptosis	41.41414141	1.85E-25	9.82E-23
GOTERM_BP_ALL	GO:0048522~positive regulation of cellular process	56.56565657	2.66E-25	1.13E-22
GOTERM_BP_ALL	GO:0051726~regulation of cell cycle	30.3030303	5.72E-25	1.21E-22
GOTERM_BP_ALL	GO:0006259~DNA metabolic process	34.34343434	2.16E-24	4.17E-22
GOTERM_BP_ALL	GO:0048518~positive regulation of biological process	57.57575758	3.54E-24	6.26E-22
GOTERM_BP_ALL	GO:0048523~negative regulation of cellular process	50.50505051	9.30E-22	1.52E-19
GOTERM_BP_ALL	GO:0042127~regulation of cell proliferation	37.37373737	1.75E-21	2.65E-19
GOTERM_BP_ALL	GO:0022402~cell cycle process	32.32323232	1.22E-20	1.62E-18
GOTERM_BP_ALL	GO:0007049~cell cycle	36.36363636	1.20E-20	1.69E-18
GOTERM_BP_ALL	GO:0048519~negative regulation of biological process	50.50505051	4.16E-20	5.19E-18
GOTERM_BP_ALL	GO:0050896~response to stimulus	66.66666667	1.23E-19	1.45E-17
GOTERM_BP_ALL	GO:0006310~DNA recombination	18.18181818	2.18E-19	2.43E-17
GOTERM_BP_ALL	GO:0042221~response to chemical stimulus	42.42424242	5.88E-19	6.24E-17
GOTERM_BP_ALL	GO:0043065~positive regulation of apoptosis	27.27272727	2.68E-18	2.70E-16
GOTERM_BP_ALL	GO:0043068~positive regulation of programmed cell death	27.27272727	3.18E-18	3.06E-16
GOTERM_BP_ALL	GO:0010942~positive regulation of cell death	27.27272727	3.56E-18	3.28E-16
GOTERM_BP_ALL	GO:0048731~system development	53.53535354	7.30E-18	6.45E-16
GOTERM_BP_ALL	GO:0008285~negative regulation of cell proliferation	25.25252525	7.65E-18	6.49E-16
GOTERM_BP_ALL	GO:0000075~cell cycle checkpoint	16.16161616	2.49E-17	2.03E-15
GOTERM_BP_ALL	GO:0048513~organ development	45.45454545	1.27E-16	8.10E-15
GOTERM_BP_ALL	GO:0065009~regulation of molecular function	35.35353535	1.15E-16	8.44E-15

GOTERM_BP_ALL	GO:0065008~regulation of biological quality	42.42424242	8.02E-17	8.77E-15
GOTERM_BP_ALL	GO:0048856~anatomical structure development	53.53535354	2.56E-16	1.52E-14
GOTERM_BP_ALL	GO:0032502~developmental process	59.5959596	1.71E-16	1.57E-14
GOTERM_BP_ALL	GO:0007275~multicellular organismal development	56.56565657	4.81E-16	2.94E-14
GOTERM_BP_ALL	GO:0051052~regulation of DNA metabolic process	16.16161616	8.30E-16	5.00E-14
GOTERM_BP_ALL	GO:0043066~negative regulation of apoptosis	23.23232323	1.01E-15	6.23E-14
GOTERM_BP_ALL	GO:0002520~immune system development	21.21212121	1.27E-15	7.41E-14
GOTERM_BP_ALL	GO:0060548~negative regulation of cell death	23.23232323	1.39E-15	7.64E-14
GOTERM_BP_ALL	GO:0043069~negative regulation of programmed cell death	23.23232323	1.31E-15	7.85E-14
GOTERM_BP_ALL	GO:0009893~positive regulation of metabolic process	33.33333333	1.82E-15	9.91E-14
GOTERM_BP_ALL	GO:0010604~positive regulation of macromolecule metabolic process	32.32323232	1.90E-15	1.03E-13
GOTERM_BP_ALL	GO:0051173~positive regulation of nitrogen compound metabolic process	28.28282828	5.68E-15	3.00E-13
GOTERM_BP_ALL	GO:0048545~response to steroid hormone stimulus	18.18181818	7.74E-15	4.02E-13
GOTERM_BP_ALL	GO:0045595~regulation of cell differentiation	25.25252525	9.03E-15	4.54E-13
GOTERM_BP_ALL	GO:0010033~response to organic substance	29.29292929	1.07E-14	5.31E-13
GOTERM_BP_ALL	GO:0009314~response to radiation	18.18181818	1.53E-14	7.39E-13
GOTERM_BP_ALL	GO:0044260~cellular macromolecule metabolic process	72.72727273	2.41E-14	1.14E-12
GOTERM_BP_ALL	GO:0031328~positive regulation of cellular biosynthetic process	28.28282828	2.53E-14	1.17E-12
GOTERM_BP_ALL	GO:0009628~response to abiotic stimulus	22.22222222	2.61E-14	1.18E-12
GOTERM_BP_ALL	GO:0016043~cellular component organization	50.50505051	3.14E-14	1.39E-12
GOTERM_BP_ALL	GO:0009891~positive regulation of biosynthetic process	28.28282828	3.59E-14	1.55E-12
GOTERM_BP_ALL	GO:0010628~positive regulation of gene expression	26.26262626	3.95E-14	1.68E-12
GOTERM_BP_ALL	GO:0042493~response to drug	18.18181818	5.47E-14	2.28E-12
GOTERM_BP_ALL	GO:0007346~regulation of mitotic cell cycle	16.16161616	6.74E-14	2.75E-12
GOTERM_BP_ALL	GO:0010557~positive regulation of macromolecule biosynthetic process	27.27272727	6.99E-14	2.80E-12
GOTERM_BP_ALL	GO:0009411~response to UV	12.12121212	1.35E-13	5.29E-12
GOTERM_BP_ALL	GO:0007050~cell cycle arrest	14.14141414	1.41E-13	5.34E-12

GOTERM_BP_ALL	GO:0050793~regulation of developmental process	27.27272727	1.41E-13	5.44E-12
GOTERM_BP_ALL	GO:0042770~DNA damage response, signal transduction	13.13131313	1.53E-13	5.71E-12
GOTERM_BP_ALL	GO:0009719~response to endogenous stimulus	22.22222222	1.71E-13	6.24E-12
GOTERM_BP_ALL	GO:0045941~positive regulation of transcription	25.25252525	1.80E-13	6.49E-12
GOTERM_BP_ALL	GO:0045935~positive regulation of nucleobase, nucleoside, nucleotide and nucleic acid metabolic process	26.26262626	1.98E-13	6.99E-12
GOTERM_BP_ALL	GO:0031325~positive regulation of cellular metabolic process	30.3030303	2.16E-13	7.50E-12
GOTERM_BP_ALL	GO:0006302~double-strand break repair	12.12121212	2.41E-13	8.23E-12
GOTERM_BP_ALL	GO:0022403~cell cycle phase	22.22222222	2.62E-13	8.81E-12
GOTERM_BP_ALL	GO:0009725~response to hormone stimulus	21.21212121	2.78E-13	9.21E-12
GOTERM_BP_ALL	GO:0044237~cellular metabolic process	79.7979798	4.94E-13	1.61E-11
GOTERM_BP_ALL	GO:0043170~macromolecule metabolic process	73.73737374	9.31E-13	2.99E-11
GOTERM_BP_ALL	GO:0009790~embryonic development	24.24242424	1.77E-12	5.61E-11
GOTERM_BP_ALL	GO:0048869~cellular developmental process	39.39393939	2.84E-12	8.84E-11
GOTERM_BP_ALL	GO:0006917~induction of apoptosis	19.19191919	2.94E-12	9.05E-11
GOTERM_BP_ALL	GO:0012502~induction of programmed cell death	19.19191919	3.10E-12	9.40E-11
GOTERM_BP_ALL	GO:0045944~positive regulation of transcription from RNA polymerase II promoter	20.2020202	3.55E-12	1.06E-10
GOTERM_BP_ALL	GO:0050790~regulation of catalytic activity	28.28282828	3.85E-12	1.13E-10
GOTERM_BP_ALL	GO:0045893~positive regulation of transcription, DNA-dependent	22.22222222	3.99E-12	1.16E-10
GOTERM_BP_ALL	GO:0030154~cell differentiation	38.38383838	4.10E-12	1.17E-10
GOTERM_BP_ALL	GO:0032501~multicellular organismal process	62.62626263	4.33E-12	1.22E-10
GOTERM_BP_ALL	GO:0031324~negative regulation of cellular metabolic process	26.26262626	4.72E-12	1.30E-10
GOTERM_BP_ALL	GO:0051254~positive regulation of RNA metabolic process	22.22222222	4.67E-12	1.30E-10
GOTERM_BP_ALL	GO:0060249~anatomical structure homeostasis	13.13131313	4.87E-12	1.32E-10
GOTERM_BP_ALL	GO:0045786~negative regulation of cell cycle	12.12121212	5.18E-12	1.39E-10
GOTERM_BP_ALL	GO:0051174~regulation of phosphorus metabolic process	22.22222222	5.47E-12	1.45E-10
GOTERM_BP_ALL	GO:0019220~regulation of phosphate metabolic process	22.22222222	5.47E-12	1.45E-10

GOTERM_BP_ALL	GO:0008361~regulation of cell size	16.16161616	5.87E-12	1.54E-10
GOTERM_BP_ALL	GO:0008152~metabolic process	83.83838384	6.35E-12	1.64E-10
GOTERM_BP_ALL	GO:0006996~organelle organization	34.34343434	8.33E-12	2.13E-10
GOTERM_BP_ALL	GO:0070482~response to oxygen levels	14.14141414	8.50E-12	2.15E-10
GOTERM_BP_ALL	GO:0031323~regulation of cellular metabolic process	55.55555556	9.11E-12	2.27E-10
GOTERM_BP_ALL	GO:0019222~regulation of metabolic process	56.56565657	1.40E-11	3.45E-10
GOTERM_BP_ALL	GO:0042325~regulation of phosphorylation	21.21212121	2.23E-11	5.43E-10
GOTERM_BP_ALL	GO:0014070~response to organic cyclic substance	13.13131313	2.39E-11	5.75E-10
GOTERM_BP_ALL	GO:0009892~negative regulation of metabolic process	26.26262626	2.68E-11	6.39E-10
GOTERM_BP_ALL	GO:0044238~primary metabolic process	78.78787879	3.65E-11	8.60E-10
GOTERM_BP_ALL	GO:0051276~chromosome organization	21.21212121	4.56E-11	1.06E-09
GOTERM_BP_ALL	GO:0007242~intracellular signaling cascade	32.32323232	4.99E-11	1.15E-09
GOTERM_BP_ALL	GO:0042592~homeostatic process	25.25252525	7.80E-11	1.78E-09
GOTERM_BP_ALL	GO:0006139~nucleobase, nucleoside, nucleotide and nucleic acid metabolic process	53.53535354	8.03E-11	1.79E-09
GOTERM_BP_ALL	GO:0001666~response to hypoxia	13.13131313	8.01E-11	1.81E-09
GOTERM_BP_ALL	GO:0006807~nitrogen compound metabolic process	56.56565657	8.29E-11	1.83E-09
GOTERM_BP_ALL	GO:0051171~regulation of nitrogen compound metabolic process	48.48484848	8.63E-11	1.89E-09
GOTERM_BP_ALL	GO:0043627~response to estrogen stimulus	12.12121212	9.34E-11	2.02E-09
GOTERM_BP_ALL	GO:0034641~cellular nitrogen compound metabolic process	55.55555556	9.89E-11	2.12E-09
GOTERM_BP_ALL	GO:0045859~regulation of protein kinase activity	18.18181818	1.02E-10	2.16E-09
GOTERM_BP_ALL	GO:0009416~response to light stimulus	13.13131313	1.13E-10	2.38E-09
GOTERM_BP_ALL	GO:0045934~negative regulation of nucleobase, nucleoside, nucleotide and nucleic acid metabolic process	21.21212121	1.20E-10	2.49E-09
GOTERM_BP_ALL	GO:0051053~negative regulation of DNA metabolic process	9.090909091	1.48E-10	3.05E-09
GOTERM_BP_ALL	GO:0051172~negative regulation of nitrogen compound metabolic process	21.21212121	1.52E-10	3.11E-09
GOTERM_BP_ALL	GO:0007568~aging	12.12121212	1.56E-10	3.14E-09
GOTERM_BP_ALL	GO:0043549~regulation of kinase activity	18.18181818	1.74E-10	3.47E-09

GOTERM_BP_ALL	GO:0044093~positive regulation of molecular function	22.22222222	1.87E-10	3.71E-09
GOTERM_BP_ALL	GO:0060255~regulation of macromolecule metabolic process	51.51515152	2.19E-10	4.30E-09
GOTERM_BP_ALL	GO:0051239~regulation of multicellular organismal process	27.27272727	2.35E-10	4.57E-09
GOTERM_BP_ALL	GO:0032535~regulation of cellular component size	16.16161616	2.88E-10	5.56E-09
GOTERM_BP_ALL	GO:0050794~regulation of cellular process	76.76767677	2.96E-10	5.66E-09
GOTERM_BP_ALL	GO:0043687~post-translational protein modification	30.3030303	2.99E-10	5.67E-09
GOTERM_BP_ALL	GO:0080090~regulation of primary metabolic process	51.51515152	3.16E-10	5.93E-09
GOTERM_BP_ALL	GO:0045597~positive regulation of cell differentiation	15.15151515	3.25E-10	6.05E-09
GOTERM_BP_ALL	GO:0051338~regulation of transferase activity	18.18181818	3.28E-10	6.05E-09
GOTERM_BP_ALL	GO:0009653~anatomical structure morphogenesis	30.3030303	4.03E-10	7.37E-09
GOTERM_BP_ALL	GO:0051094~positive regulation of developmental process	16.16161616	4.12E-10	7.46E-09
GOTERM_BP_ALL	GO:0009792~embryonic development ending in birth or egg hatching	17.17171717	5.82E-10	1.05E-08
GOTERM_BP_ALL	GO:0022414~reproductive process	24.24242424	6.46E-10	1.15E-08
GOTERM_BP_ALL	GO:0000003~reproduction	24.24242424	7.33E-10	1.30E-08
GOTERM_BP_ALL	GO:0040008~regulation of growth	17.17171717	7.88E-10	1.38E-08
GOTERM_BP_ALL	GO:0000077~DNA damage checkpoint	9.090909091	8.66E-10	1.51E-08
GOTERM_BP_ALL	GO:0045792~negative regulation of cell size	11.11111111	1.01E-09	1.74E-08
GOTERM_BP_ALL	GO:0007399~nervous system development	28.28282828	1.16E-09	1.98E-08
GOTERM_BP_ALL	GO:0048646~anatomical structure formation involved in morphogenesis	17.17171717	1.25E-09	2.12E-08
GOTERM_BP_ALL	GO:0043412~biopolymer modification	33.33333333	1.43E-09	2.41E-08
GOTERM_BP_ALL	GO:0006357~regulation of transcription from RNA polymerase II promoter	23.23232323	1.57E-09	2.62E-08
GOTERM_BP_ALL	GO:0031570~DNA integrity checkpoint	9.090909091	1.69E-09	2.78E-08
GOTERM_BP_ALL	GO:0008630~DNA damage response, signal transduction resulting in induction of apoptosis	8.080808081	1.72E-09	2.78E-08
GOTERM_BP_ALL	GO:0048534~hemopoietic or lymphoid organ development	15.15151515	1.71E-09	2.79E-08
GOTERM_BP_ALL	GO:0008284~positive regulation of cell proliferation	18.18181818	1.68E-09	2.79E-08
GOTERM_BP_ALL	GO:0009888~tissue development	22.22222222	1.86E-09	2.99E-08

GOTERM_BP_ALL	GO:0010605~negative regulation of macromolecule metabolic process	23.23232323	1.87E-09	2.99E-08
GOTERM_BP_ALL	GO:0006468~protein amino acid phosphorylation	22.22222222	1.96E-09	3.10E-08
GOTERM_BP_ALL	GO:0000079~regulation of cyclin-dependent protein kinase activity	9.090909091	2.31E-09	3.63E-08
GOTERM_BP_ALL	GO:0008629~induction of apoptosis by intracellular signals	9.090909091	2.31E-09	3.63E-08
GOTERM_BP_ALL	GO:0040007~growth	13.13131313	2.97E-09	4.64E-08
GOTERM_BP_ALL	GO:0050789~regulation of biological process	76.76767677	3.17E-09	4.91E-08
GOTERM_BP_ALL	GO:0019219~regulation of nucleobase, nucleoside, nucleotide and nucleic acid metabolic process	45.45454545	3.64E-09	5.59E-08
GOTERM_BP_ALL	GO:0010564~regulation of cell cycle process	11.11111111	4.05E-09	6.18E-08
GOTERM_BP_ALL	GO:0046822~regulation of nucleocytoplasmic transport	9.090909091	4.17E-09	6.31E-08
GOTERM_BP_ALL	GO:0043009~chordate embryonic development	16.16161616	4.52E-09	6.81E-08
GOTERM_BP_ALL	GO:0030097~hemopoiesis	14.14141414	5.21E-09	7.78E-08
GOTERM_BP_ALL	GO:0051098~regulation of binding	12.12121212	5.39E-09	7.99E-08
GOTERM_BP_ALL	GO:0001558~regulation of cell growth	13.13131313	5.76E-09	8.49E-08
GOTERM_BP_ALL	GO:0016310~phosphorylation	23.23232323	9.13E-09	1.33E-07
GOTERM_BP_ALL	GO:0007131~reciprocal meiotic recombination	7.070707071	9.07E-09	1.33E-07
GOTERM_BP_ALL	GO:0016444~somatic cell DNA recombination	7.070707071	9.07E-09	1.33E-07
GOTERM_BP_ALL	GO:0002562~somatic diversification of immune receptors via germline recombination within a single locus	7.070707071	9.07E-09	1.33E-07
GOTERM_BP_ALL	GO:0007127~meiosis I	8.080808081	1.03E-08	1.49E-07
GOTERM_BP_ALL	GO:0006298~mismatch repair	7.070707071	1.19E-08	1.70E-07
GOTERM_BP_ALL	GO:0022008~neurogenesis	20.2020202	1.23E-08	1.75E-07
GOTERM_BP_ALL	GO:0000018~regulation of DNA recombination	7.070707071	1.53E-08	2.17E-07
GOTERM_BP_ALL	GO:0065007~biological regulation	77.77777778	1.56E-08	2.19E-07
GOTERM_BP_ALL	GO:0007126~meiosis	10.1010101	1.69E-08	2.35E-07
GOTERM_BP_ALL	GO:0051327~M phase of meiotic cell cycle	10.1010101	1.69E-08	2.35E-07
GOTERM_BP_ALL	GO:0002200~somatic diversification of immune receptors	7.070707071	1.96E-08	2.72E-07
GOTERM_BP_ALL	GO:0051321~meiotic cell cycle	10.1010101	2.02E-08	2.78E-07
GOTERM_BP_ALL	GO:0050678~regulation of epithelial cell proliferation	9.090909091	2.16E-08	2.95E-07
GOTERM_BP_ALL	GO:0032386~regulation of intracellular transport	9.090909091	2.16E-08	2.95E-07
GOTERM_BP_ALL	GO:0009991~response to extracellular stimulus	13.13131313	2.36E-08	3.21E-07

GOTERM_BP_ALL	GO:0000723~telomere maintenance	7.070707071	2.48E-08	3.36E-07
GOTERM_BP_ALL	GO:0051329~interphase of mitotic cell cycle	10.1010101	2.62E-08	3.51E-07
GOTERM_BP_ALL	GO:0032200~telomere organization	7.070707071	3.12E-08	4.16E-07
GOTERM_BP_ALL	GO:0051325~interphase	10.1010101	3.37E-08	4.47E-07
GOTERM_BP_ALL	GO:0006464~protein modification process	30.3030303	3.41E-08	4.49E-07
GOTERM_BP_ALL	GO:0000279~M phase	15.15151515	3.44E-08	4.50E-07
GOTERM_BP_ALL	GO:0010647~positive regulation of cell communication	15.15151515	3.44E-08	4.50E-07
GOTERM_BP_ALL	GO:0045926~negative regulation of growth	10.1010101	4.66E-08	6.07E-07
GOTERM_BP_ALL	GO:0043085~positive regulation of catalytic activity	18.18181818	4.96E-08	6.42E-07
GOTERM_BP_ALL	GO:0007167~enzyme linked receptor protein signaling pathway	15.15151515	5.57E-08	7.12E-07
GOTERM_BP_ALL	GO:0033157~regulation of intracellular protein transport	8.080808081	5.56E-08	7.15E-07
GOTERM_BP_ALL	GO:0051270~regulation of cell motion	12.12121212	6.03E-08	7.66E-07
GOTERM_BP_ALL	GO:0006796~phosphate metabolic process	24.24242424	6.57E-08	8.29E-07
GOTERM_BP_ALL	GO:0006793~phosphorus metabolic process	24.24242424	6.57E-08	8.29E-07
GOTERM_BP_ALL	GO:0031667~response to nutrient levels	12.12121212	7.44E-08	9.34E-07
GOTERM_BP_ALL	GO:0001568~blood vessel development	13.13131313	7.75E-08	9.66E-07
GOTERM_BP_ALL	GO:0000082~G1/S transition of mitotic cell cycle	8.080808081	8.23E-08	1.02E-06
GOTERM_BP_ALL	GO:0010627~regulation of protein kinase cascade	13.13131313	9.25E-08	1.14E-06
GOTERM_BP_ALL	GO:0016447~somatic recombination of immunoglobulin gene segments	6.060606061	9.41E-08	1.15E-06
GOTERM_BP_ALL	GO:0001944~vasculature development	13.13131313	1.01E-07	1.23E-06
GOTERM_BP_ALL	GO:0002376~immune system process	24.24242424	1.04E-07	1.26E-06
GOTERM_BP_ALL	GO:0006469~negative regulation of protein kinase activity	9.090909091	1.09E-07	1.31E-06
GOTERM_BP_ALL	GO:0043193~positive regulation of gene-specific transcription	9.090909091	1.09E-07	1.31E-06
GOTERM_BP_ALL	GO:0010212~response to ionizing radiation	8.080808081	1.34E-07	1.61E-06
GOTERM_BP_ALL	GO:0051093~negative regulation of developmental process	13.13131313	1.42E-07	1.68E-06
GOTERM_BP_ALL	GO:0033673~negative regulation of kinase activity	9.090909091	1.42E-07	1.69E-06

GOTERM_BP_ALL	GO:0048514~blood vessel morphogenesis	12.12121212	1.50E-07	1.76E-06
GOTERM_BP_ALL	GO:0009887~organ morphogenesis	18.18181818	1.60E-07	1.87E-06
GOTERM_BP_ALL	GO:0006275~regulation of DNA replication	8.080808081	1.69E-07	1.96E-06
GOTERM_BP_ALL	GO:0030308~negative regulation of cell growth	9.090909091	1.68E-07	1.96E-06
GOTERM_BP_ALL	GO:0008283~cell proliferation	16.16161616	1.74E-07	2.01E-06
GOTERM_BP_ALL	GO:0045596~negative regulation of cell differentiation	12.12121212	1.90E-07	2.17E-06
GOTERM_BP_ALL	GO:0048468~cell development	19.19191919	2.02E-07	2.30E-06
GOTERM_BP_ALL	GO:0051348~negative regulation of transferase activity	9.090909091	2.35E-07	2.66E-06
GOTERM_BP_ALL	GO:0032583~regulation of gene-specific transcription	10.1010101	2.57E-07	2.90E-06
GOTERM_BP_ALL	GO:0016445~somatic diversification of immunoglobulins	6.060606061	2.83E-07	3.18E-06
GOTERM_BP_ALL	GO:0051345~positive regulation of hydrolase activity	11.11111111	3.01E-07	3.36E-06
GOTERM_BP_ALL	GO:0030155~regulation of cell adhesion	10.1010101	3.10E-07	3.45E-06
GOTERM_BP_ALL	GO:0009987~cellular process	90.90909091	3.27E-07	3.60E-06
GOTERM_BP_ALL	GO:0032880~regulation of protein localization	10.1010101	3.30E-07	3.61E-06
GOTERM_BP_ALL	GO:0051128~regulation of cellular component organization	16.16161616	3.27E-07	3.62E-06
GOTERM_BP_ALL	GO:0051130~positive regulation of cellular component organization	11.11111111	3.34E-07	3.63E-06
GOTERM_BP_ALL	GO:0051336~regulation of hydrolase activity	14.14141414	3.43E-07	3.71E-06
GOTERM_BP_ALL	GO:0042306~regulation of protein import into nucleus	7.070707071	4.27E-07	4.60E-06
GOTERM_BP_ALL	GO:0032879~regulation of localization	18.18181818	4.83E-07	5.17E-06
GOTERM_BP_ALL	GO:0042326~negative regulation of phosphorylation	7.070707071	4.90E-07	5.23E-06
GOTERM_BP_ALL	GO:0051246~regulation of protein metabolic process	17.17171717	5.65E-07	5.99E-06
GOTERM_BP_ALL	GO:0009967~positive regulation of signal transduction	13.13131313	5.75E-07	6.07E-06
GOTERM_BP_ALL	GO:0045736~negative regulation of cyclin-dependent protein kinase activity	5.050505051	5.87E-07	6.17E-06
GOTERM_BP_ALL	GO:0051247~positive regulation of protein metabolic process	12.12121212	6.16E-07	6.43E-06
GOTERM_BP_ALL	GO:0048609~reproductive process in a multicellular organism	16.16161616	7.14E-07	7.42E-06
GOTERM_BP_ALL	GO:0032504~multicellular organism reproduction	16.16161616	7.14E-07	7.42E-06
GOTERM_BP_ALL	GO:0045936~negative regulation of phosphate metabolic process	7.070707071	7.27E-07	7.52E-06

GOTERM_BP_ALL	GO:0010563~negative regulation of phosphorus metabolic process	7.070707071	7.27E-07	7.52E-06
GOTERM_BP_ALL	GO:0007417~central nervous system development	15.15151515	7.87E-07	8.10E-06
GOTERM_BP_ALL	GO:0006284~base-excision repair	6.060606061	8.46E-07	8.66E-06
GOTERM_BP_ALL	GO:0009966~regulation of signal transduction	21.21212121	1.01E-06	1.03E-05
GOTERM_BP_ALL	GO:0006916~anti-apoptosis	11.11111111	1.10E-06	1.11E-05
GOTERM_BP_ALL	GO:0051252~regulation of RNA metabolic process	31.31313131	1.15E-06	1.16E-05
GOTERM_BP_ALL	GO:0001889~liver development	7.070707071	1.32E-06	1.33E-05
GOTERM_BP_ALL	GO:0051101~regulation of DNA binding	9.090909091	1.39E-06	1.39E-05
GOTERM_BP_ALL	GO:0070201~regulation of establishment of protein localization	9.090909091	1.39E-06	1.39E-05
GOTERM_BP_ALL	GO:0002377~immunoglobulin production	6.060606061	1.47E-06	1.46E-05
GOTERM_BP_ALL	GO:0032388~positive regulation of intracellular transport	6.060606061	1.47E-06	1.46E-05
GOTERM_BP_ALL	GO:0002682~regulation of immune system process	14.14141414	1.54E-06	1.53E-05
GOTERM_BP_ALL	GO:0048661~positive regulation of smooth muscle cell proliferation	6.060606061	1.74E-06	1.72E-05
GOTERM_BP_ALL	GO:0002440~production of molecular mediator of immune response	6.060606061	1.74E-06	1.72E-05
GOTERM_BP_ALL	GO:0030334~regulation of cell migration	10.1010101	1.82E-06	1.78E-05
GOTERM_BP_ALL	GO:0045787~positive regulation of cell cycle	7.070707071	2.05E-06	2.00E-05
GOTERM_BP_ALL	GO:0043523~regulation of neuron apoptosis	8.080808081	2.21E-06	2.13E-05
GOTERM_BP_ALL	GO:0001932~regulation of protein amino acid phosphorylation	10.1010101	2.21E-06	2.14E-05
GOTERM_BP_ALL	GO:0051704~multi-organism process	18.18181818	2.20E-06	2.14E-05
GOTERM_BP_ALL	GO:0030518~steroid hormone receptor signaling pathway	7.070707071	2.27E-06	2.18E-05
GOTERM_BP_ALL	GO:0006355~regulation of transcription, DNA-dependent	30.3030303	2.36E-06	2.25E-05
GOTERM_BP_ALL	GO:0001701~in utero embryonic development	10.1010101	2.54E-06	2.42E-05
GOTERM_BP_ALL	GO:0010558~negative regulation of macromolecule biosynthetic process	16.16161616	3.02E-06	2.86E-05
GOTERM_BP_ALL	GO:0001775~cell activation	12.12121212	3.13E-06	2.95E-05
GOTERM_BP_ALL	GO:0010646~regulation of cell communication	22.22222222	3.46E-06	3.25E-05
GOTERM_BP_ALL	GO:0010741~negative regulation of protein kinase cascade	6.060606061	3.77E-06	3.52E-05

GOTERM_BP_ALL	GO:0048699~generation of neurons	16.16161616	3.94E-06	3.67E-05
GOTERM_BP_ALL	GO:0031399~regulation of protein modification process	12.12121212	4.08E-06	3.78E-05
GOTERM_BP_ALL	GO:0031327~negative regulation of cellular biosynthetic process	16.16161616	4.12E-06	3.78E-05
GOTERM_BP_ALL	GO:0043409~negative regulation of MAPKKK cascade	5.050505051	4.11E-06	3.79E-05
GOTERM_BP_ALL	GO:0007584~response to nutrient	9.090909091	4.16E-06	3.80E-05
GOTERM_BP_ALL	GO:0042063~gliogenesis	7.070707071	4.47E-06	4.07E-05
GOTERM_BP_ALL	GO:0031326~regulation of cellular biosynthetic process	40.4040404	4.49E-06	4.07E-05
GOTERM_BP_ALL	GO:0008219~cell death	18.18181818	4.57E-06	4.13E-05
GOTERM_BP_ALL	GO:0016265~death	18.18181818	5.01E-06	4.51E-05
GOTERM_BP_ALL	GO:0040012~regulation of locomotion	10.1010101	5.19E-06	4.64E-05
GOTERM_BP_ALL	GO:0009889~regulation of biosynthetic process	40.4040404	5.29E-06	4.71E-05
GOTERM_BP_ALL	GO:0009890~negative regulation of biosynthetic process	16.16161616	5.32E-06	4.72E-05
GOTERM_BP_ALL	GO:0070271~protein complex biogenesis	15.15151515	5.92E-06	5.23E-05
GOTERM_BP_ALL	GO:0006461~protein complex assembly	15.15151515	5.92E-06	5.23E-05
GOTERM_BP_ALL	GO:0001525~angiogenesis	9.090909091	6.28E-06	5.53E-05
GOTERM_BP_ALL	GO:0007398~ectoderm development	10.1010101	6.94E-06	6.09E-05
GOTERM_BP_ALL	GO:0043408~regulation of MAPKKK cascade	8.080808081	7.94E-06	6.93E-05
GOTERM_BP_ALL	GO:0007093~mitotic cell cycle checkpoint	6.060606061	9.27E-06	8.06E-05
GOTERM_BP_ALL	GO:0051095~regulation of helicase activity	4.04040404	9.82E-06	8.50E-05
GOTERM_BP_ALL	GO:0045910~negative regulation of DNA recombination	4.04040404	9.82E-06	8.50E-05
GOTERM_BP_ALL	GO:0030522~intracellular receptor-mediated signaling pathway	7.070707071	1.03E-05	8.90E-05
GOTERM_BP_ALL	GO:0051223~regulation of protein transport	8.080808081	1.07E-05	9.17E-05
GOTERM_BP_ALL	GO:0012501~programmed cell death	16.16161616	1.15E-05	9.83E-05
GOTERM_BP_ALL	GO:0045930~negative regulation of mitotic cell cycle	5.050505051	1.23E-05	1.05E-04
GOTERM_BP_ALL	GO:0043525~positive regulation of neuron apoptosis	5.050505051	1.23E-05	1.05E-04
GOTERM_BP_ALL	GO:0010556~regulation of macromolecule biosynthetic process	38.38383838	1.25E-05	1.06E-04
GOTERM_BP_ALL	GO:0048660~regulation of smooth muscle cell proliferation	6.060606061	1.30E-05	1.09E-04
GOTERM_BP_ALL	GO:0007507~heart development	10.1010101	1.29E-05	1.09E-04

GOTERM_BP_ALL	GO:0043281~regulation of caspase activity	7.070707071	1.40E-05	1.17E-04
GOTERM_BP_ALL	GO:0033043~regulation of organelle organization	10.1010101	1.39E-05	1.17E-04
GOTERM_BP_ALL	GO:0032268~regulation of cellular protein metabolic process	14.14141414	1.47E-05	1.23E-04
GOTERM_BP_ALL	GO:0022603~regulation of anatomical structure morphogenesis	10.1010101	1.50E-05	1.24E-04
GOTERM_BP_ALL	GO:0043086~negative regulation of catalytic activity	11.11111111	1.52E-05	1.26E-04
GOTERM_BP_ALL	GO:0010468~regulation of gene expression	38.38383838	1.54E-05	1.27E-04
GOTERM_BP_ALL	GO:0052548~regulation of endopeptidase activity	7.070707071	1.73E-05	1.42E-04
GOTERM_BP_ALL	GO:0007169~transmembrane receptor protein tyrosine kinase signaling pathway	10.1010101	1.79E-05	1.46E-04
GOTERM_BP_ALL	GO:0044419~interspecies interaction between organisms	11.11111111	1.83E-05	1.49E-04
GOTERM_BP_ALL	GO:0010638~positive regulation of organelle organization	7.070707071	1.85E-05	1.50E-04
GOTERM_BP_ALL	GO:0010948~negative regulation of cell cycle process	5.050505051	2.10E-05	1.69E-04
GOTERM_BP_ALL	GO:0050680~negative regulation of epithelial cell proliferation	5.050505051	2.10E-05	1.69E-04
GOTERM_BP_ALL	GO:0031960~response to corticosteroid stimulus	7.070707071	2.13E-05	1.71E-04
GOTERM_BP_ALL	GO:0052547~regulation of peptidase activity	7.070707071	2.27E-05	1.82E-04
GOTERM_BP_ALL	GO:0016446~somatic hypermutation of immunoglobulin genes	4.04040404	2.33E-05	1.86E-04
GOTERM_BP_ALL	GO:0002566~somatic diversification of immune receptors via somatic mutation	4.04040404	2.33E-05	1.86E-04
GOTERM_BP_ALL	GO:0032270~positive regulation of cellular protein metabolic process	10.1010101	2.45E-05	1.95E-04
GOTERM_BP_ALL	GO:0031100~organ regeneration	5.050505051	2.46E-05	1.95E-04
GOTERM_BP_ALL	GO:0010001~glial cell differentiation	6.060606061	2.62E-05	2.07E-04
GOTERM_BP_ALL	GO:0002521~leukocyte differentiation	8.080808081	2.64E-05	2.08E-04
GOTERM_BP_ALL	GO:0051253~negative regulation of RNA metabolic process	12.12121212	2.79E-05	2.18E-04
GOTERM_BP_ALL	GO:0001952~regulation of cell-matrix adhesion	5.050505051	2.88E-05	2.24E-04
GOTERM_BP_ALL	GO:0048732~gland development	8.080808081	3.21E-05	2.49E-04
GOTERM_BP_ALL	GO:0045321~leukocyte activation	10.1010101	3.30E-05	2.56E-04
GOTERM_BP_ALL	GO:0006303~double-strand break repair via nonhomologous end joining	4.04040404	3.32E-05	2.56E-04

GOTERM_BP_ALL	GO:0008156~negative regulation of DNA replication	5.050505051	3.34E-05	2.57E-04
GOTERM_BP_ALL	GO:0000278~mitotic cell cycle	12.12121212	3.41E-05	2.61E-04
GOTERM_BP_ALL	GO:0030099~myeloid cell differentiation	7.070707071	3.55E-05	2.70E-04
GOTERM_BP_ALL	GO:0007165~signal transduction	37.37373737	3.54E-05	2.70E-04
GOTERM_BP_ALL	GO:0048598~embryonic morphogenesis	11.11111111	3.68E-05	2.79E-04
GOTERM_BP_ALL	GO:0010552~positive regulation of specific transcription from RNA polymerase II promoter	6.060606061	3.75E-05	2.83E-04
GOTERM_BP_ALL	GO:0006260~DNA replication	9.090909091	3.85E-05	2.90E-04
GOTERM_BP_ALL	GO:0060341~regulation of cellular localization	10.1010101	4.00E-05	3.00E-04
GOTERM_BP_ALL	GO:0006915~apoptosis	15.15151515	4.22E-05	3.15E-04
GOTERM_BP_ALL	GO:0042327~positive regulation of phosphorylation	7.070707071	4.51E-05	3.35E-04
GOTERM_BP_ALL	GO:0051272~positive regulation of cell motion	7.070707071	4.77E-05	3.54E-04
GOTERM_BP_ALL	GO:0007179~transforming growth factor beta receptor signaling pathway	6.060606061	4.81E-05	3.55E-04
GOTERM_BP_ALL	GO:0006308~DNA catabolic process	6.060606061	4.81E-05	3.55E-04
GOTERM_BP_ALL	GO:0008637~apoptotic mitochondrial changes	5.050505051	5.06E-05	3.72E-04
GOTERM_BP_ALL	GO:0001501~skeletal system development	11.11111111	5.09E-05	3.73E-04
GOTERM_BP_ALL	GO:0016481~negative regulation of transcription	13.13131313	5.15E-05	3.77E-04
GOTERM_BP_ALL	GO:0046649~lymphocyte activation	9.090909091	5.35E-05	3.89E-04
GOTERM_BP_ALL	GO:0010562~positive regulation of phosphorus metabolic process	7.070707071	5.35E-05	3.90E-04
GOTERM_BP_ALL	GO:0045937~positive regulation of phosphate metabolic process	7.070707071	5.35E-05	3.90E-04
GOTERM_BP_ALL	GO:0048872~homeostasis of number of cells	7.070707071	5.35E-05	3.90E-04
GOTERM_BP_ALL	GO:0021700~developmental maturation	7.070707071	5.66E-05	4.08E-04
GOTERM_BP_ALL	GO:0022415~viral reproductive process	6.060606061	5.64E-05	4.08E-04
GOTERM_BP_ALL	GO:0048583~regulation of response to stimulus	13.13131313	5.84E-05	4.19E-04
GOTERM_BP_ALL	GO:0002208~somatic diversification of immunoglobulins during immune response	4.04040404	6.02E-05	4.32E-04
GOTERM_BP_ALL	GO:0045190~isotype switching	4.04040404	6.02E-05	4.32E-04
GOTERM_BP_ALL	GO:0002204~somatic recombination of immunoglobulin genes during immune response	4.04040404	6.02E-05	4.32E-04

GOTERM_BP_ALL	GO:0010243~response to organic nitrogen	6.060606061	6.09E-05	4.35E-04
GOTERM_BP_ALL	GO:0051090~regulation of transcription factor activity	7.070707071	6.31E-05	4.49E-04
GOTERM_BP_ALL	GO:0010035~response to inorganic substance	9.090909091	6.60E-05	4.68E-04
GOTERM_BP_ALL	GO:0006928~cell motion	13.13131313	7.16E-05	5.06E-04
GOTERM_BP_ALL	GO:0000060~protein import into nucleus, translocation	5.050505051	7.34E-05	5.17E-04
GOTERM_BP_ALL	GO:0044092~negative regulation of molecular function	11.11111111	7.47E-05	5.24E-04
GOTERM_BP_ALL	GO:0002381~immunoglobulin production during immune response	4.04040404	7.79E-05	5.45E-04
GOTERM_BP_ALL	GO:0010608~posttranscriptional regulation of gene expression	9.090909091	8.07E-05	5.63E-04
GOTERM_BP_ALL	GO:0051222~positive regulation of protein transport	6.060606061	8.20E-05	5.70E-04
GOTERM_BP_ALL	GO:0016477~cell migration	10.1010101	9.13E-05	6.33E-04
GOTERM_BP_ALL	GO:0048565~gut development	5.050505051	9.23E-05	6.35E-04
GOTERM_BP_ALL	GO:0030521~androgen receptor signaling pathway	5.050505051	9.23E-05	6.35E-04
GOTERM_BP_ALL	GO:0044085~cellular component biogenesis	19.19191919	9.22E-05	6.37E-04
GOTERM_BP_ALL	GO:0009605~response to external stimulus	18.18181818	9.88E-05	6.76E-04
GOTERM_BP_ALL	GO:0000726~non-recombinational repair	4.04040404	9.87E-05	6.77E-04
GOTERM_BP_ALL	GO:0019216~regulation of lipid metabolic process	7.070707071	1.01E-04	6.87E-04
GOTERM_BP_ALL	GO:0045637~regulation of myeloid cell differentiation	6.060606061	1.01E-04	6.88E-04
GOTERM_BP_ALL	GO:0043388~positive regulation of DNA binding	6.060606061	1.01E-04	6.88E-04
GOTERM_BP_ALL	GO:0018193~peptidyl-amino acid modification	8.080808081	1.02E-04	6.92E-04
GOTERM_BP_ALL	GO:0043062~extracellular structure organization	8.080808081	1.06E-04	7.16E-04
GOTERM_BP_ALL	GO:0016032~viral reproduction	6.060606061	1.08E-04	7.29E-04
GOTERM_BP_ALL	GO:0006979~response to oxidative stress	8.080808081	1.10E-04	7.40E-04
GOTERM_BP_ALL	GO:0001503~ossification	7.070707071	1.17E-04	7.80E-04
GOTERM_BP_ALL	GO:0010165~response to X-ray	4.04040404	1.23E-04	8.16E-04
GOTERM_BP_ALL	GO:0065003~macromolecular complex assembly	15.15151515	1.22E-04	8.16E-04
GOTERM_BP_ALL	GO:0010629~negative regulation of gene expression	13.13131313	1.25E-04	8.31E-04
GOTERM_BP_ALL	GO:0045892~negative regulation of transcription, DNA-dependent	11.11111111	1.26E-04	8.35E-04
GOTERM_BP_ALL	GO:0007420~brain development	10.1010101	1.30E-04	8.53E-04
GOTERM_BP_ALL	GO:0043570~maintenance of DNA repeat elements	3.03030303	1.31E-04	8.60E-04

GOTERM_BP_ALL	GO:0070141~response to UV-A	3.03030303	1.31E-04	8.60E-04
GOTERM_BP_ALL	GO:0007423~sensory organ development	9.090909091	1.43E-04	9.32E-04
GOTERM_BP_ALL	GO:0051049~regulation of transport	12.12121212	1.45E-04	9.46E-04
GOTERM_BP_ALL	GO:0007090~regulation of S phase of mitotic cell cycle	4.04040404	1.50E-04	9.78E-04
GOTERM_BP_ALL	GO:0051301~cell division	10.1010101	1.51E-04	9.81E-04
GOTERM_BP_ALL	GO:0002761~regulation of myeloid leukocyte differentiation	5.050505051	1.55E-04	9.99E-04
GOTERM_BP_ALL	GO:0060348~bone development	7.070707071	1.69E-04	0.001087073
GOTERM_BP_ALL	GO:0051099~positive regulation of binding	6.060606061	1.69E-04	0.001087107
GOTERM_BP_ALL	GO:0051384~response to glucocorticoid stimulus	6.060606061	1.69E-04	0.001087107
GOTERM_BP_ALL	GO:0007243~protein kinase cascade	11.11111111	1.73E-04	0.00110901
GOTERM_BP_ALL	GO:0034097~response to cytokine stimulus	6.060606061	1.80E-04	0.00114752
GOTERM_BP_ALL	GO:0051896~regulation of protein kinase B signaling cascade	4.04040404	1.82E-04	0.001156982
GOTERM_BP_ALL	GO:0031575~G1/S transition checkpoint	4.04040404	1.82E-04	0.001156982
GOTERM_BP_ALL	GO:0001824~blastocyst development	5.050505051	1.87E-04	0.001183941
GOTERM_BP_ALL	GO:0009057~macromolecule catabolic process	16.16161616	1.89E-04	0.001195999
GOTERM_BP_ALL	GO:0044267~cellular protein metabolic process	31.31313131	2.02E-04	0.001273395
GOTERM_BP_ALL	GO:0051674~localization of cell	10.1010101	2.04E-04	0.001282209
GOTERM_BP_ALL	GO:0048870~cell motility	10.1010101	2.04E-04	0.001282209
GOTERM_BP_ALL	GO:0045449~regulation of transcription	33.33333333	2.17E-04	0.001358415
GOTERM_BP_ALL	GO:0033993~response to lipid	4.04040404	2.17E-04	0.001361135
GOTERM_BP_ALL	GO:0010038~response to metal ion	7.070707071	2.19E-04	0.00136365
GOTERM_BP_ALL	GO:0051495~positive regulation of cytoskeleton organization	5.050505051	2.23E-04	0.001385776
GOTERM_BP_ALL	GO:0008544~epidermis development	8.080808081	2.25E-04	0.001392577
GOTERM_BP_ALL	GO:0022607~cellular component assembly	17.17171717	2.31E-04	0.001428434
GOTERM_BP_ALL	GO:0043933~macromolecular complex subunit organization	15.15151515	2.42E-04	0.001490051
GOTERM_BP_ALL	GO:0010810~regulation of cell-substrate adhesion	5.050505051	2.43E-04	0.001492489
GOTERM_BP_ALL	GO:0010648~negative regulation of cell communication	9.090909091	2.46E-04	0.001504564
GOTERM_BP_ALL	GO:0031401~positive regulation of protein modification process	8.080808081	2.48E-04	0.001515191
GOTERM_BP_ALL	GO:0070723~response to cholesterol	3.03030303	2.61E-04	0.0015895

GOTERM_BP_ALL	GO:0032909~regulation of transforming growth factor-beta2 production	3.03030303	2.61E-04	0.0015895
GOTERM_BP_ALL	GO:0000019~regulation of mitotic recombination	3.03030303	2.61E-04	0.0015895
GOTERM_BP_ALL	GO:0051051~negative regulation of transport	7.070707071	2.80E-04	0.001699052
GOTERM_BP_ALL	GO:0048589~developmental growth	6.060606061	2.83E-04	0.001711068
GOTERM_BP_ALL	GO:0051493~regulation of cytoskeleton organization	7.070707071	2.91E-04	0.001757925
GOTERM_BP_ALL	GO:0033261~regulation of S phase	4.04040404	3.00E-04	0.001808081
GOTERM_BP_ALL	GO:0051385~response to mineralocorticoid stimulus	4.04040404	3.00E-04	0.001808081
GOTERM_BP_ALL	GO:0009896~positive regulation of catabolic process	5.050505051	3.11E-04	0.001864973
GOTERM_BP_ALL	GO:0030335~positive regulation of cell migration	6.060606061	3.14E-04	0.001880518
GOTERM_BP_ALL	GO:0001934~positive regulation of protein amino acid phosphorylation	6.060606061	3.14E-04	0.001880518
GOTERM_BP_ALL	GO:0007140~male meiosis	4.04040404	3.49E-04	0.002081299
GOTERM_BP_ALL	GO:0045884~regulation of survival gene product expression	4.04040404	3.49E-04	0.002081299
GOTERM_BP_ALL	GO:0003006~reproductive developmental process	9.090909091	3.55E-04	0.002115834
GOTERM_BP_ALL	GO:0044087~regulation of cellular component biogenesis	7.070707071	3.67E-04	0.002179132
GOTERM_BP_ALL	GO:0010551~regulation of specific transcription from RNA polymerase II promoter	6.060606061	4.05E-04	0.002395153
GOTERM_BP_ALL	GO:0051096~positive regulation of helicase activity	3.03030303	4.33E-04	0.002548738
GOTERM_BP_ALL	GO:0060284~regulation of cell development	8.080808081	4.33E-04	0.002554215
GOTERM_BP_ALL	GO:0009894~regulation of catabolic process	6.060606061	4.46E-04	0.002617244
GOTERM_BP_ALL	GO:0032355~response to estradiol stimulus	5.050505051	4.52E-04	0.002645617
GOTERM_BP_ALL	GO:0006289~nucleotide-excision repair	5.050505051	4.85E-04	0.002830642
GOTERM_BP_ALL	GO:0040017~positive regulation of locomotion	6.060606061	4.90E-04	0.002853644
GOTERM_BP_ALL	GO:0048771~tissue remodeling	5.050505051	5.20E-04	0.003016112
GOTERM_BP_ALL	GO:0048585~negative regulation of response to stimulus	6.060606061	5.38E-04	0.003113316
GOTERM_BP_ALL	GO:0032768~regulation of monooxygenase activity	4.04040404	5.91E-04	0.00341318
GOTERM_BP_ALL	GO:0007178~transmembrane receptor protein serine/threonine kinase signaling pathway	6.060606061	6.16E-04	0.003543803
GOTERM_BP_ALL	GO:0043280~positive regulation of caspase activity	5.050505051	6.34E-04	0.003640145

GOTERM_BP_ALL	GO:0010952~positive regulation of peptidase activity	5.050505051	6.34E-04	0.003640145
GOTERM_BP_ALL	GO:0007406~negative regulation of neuroblast proliferation	3.03030303	6.47E-04	0.003701894
GOTERM_BP_ALL	GO:0060391~positive regulation of SMAD protein nuclear translocation	3.03030303	6.47E-04	0.003701894
GOTERM_BP_ALL	GO:0060390~regulation of SMAD protein nuclear translocation	3.03030303	6.47E-04	0.003701894
GOTERM_BP_ALL	GO:0035295~tube development	8.080808081	6.59E-04	0.003763125
GOTERM_BP_ALL	GO:0030330~DNA damage response, signal transduction by p53 class mediator	4.04040404	6.65E-04	0.003787648
GOTERM_BP_ALL	GO:0051091~positive regulation of transcription factor activity	5.050505051	6.76E-04	0.00383852
GOTERM_BP_ALL	GO:0045785~positive regulation of cell adhesion	5.050505051	6.76E-04	0.00383852
GOTERM_BP_ALL	GO:0045860~positive regulation of protein kinase activity	8.080808081	7.14E-04	0.004043432
GOTERM_BP_ALL	GO:0016049~cell growth	5.050505051	7.20E-04	0.004064952
GOTERM_BP_ALL	GO:0048610~reproductive cellular process	7.070707071	7.38E-04	0.004153702
GOTERM_BP_ALL	GO:0045732~positive regulation of protein catabolic process	4.04040404	7.45E-04	0.00418372
GOTERM_BP_ALL	GO:0042130~negative regulation of T cell proliferation	4.04040404	7.45E-04	0.00418372
GOTERM_BP_ALL	GO:0060429~epithelium development	8.080808081	7.93E-04	0.004441361
GOTERM_BP_ALL	GO:0051271~negative regulation of cell motion	5.050505051	8.13E-04	0.004543751
GOTERM_BP_ALL	GO:0001655~urogenital system development	6.060606061	8.30E-04	0.004624091
GOTERM_BP_ALL	GO:0010740~positive regulation of protein kinase cascade	7.070707071	8.64E-04	0.004802853
GOTERM_BP_ALL	GO:0033674~positive regulation of kinase activity	8.080808081	8.79E-04	0.004868082
GOTERM_BP_ALL	GO:0033160~positive regulation of protein import into nucleus, translocation	3.03030303	9.02E-04	0.004969515
GOTERM_BP_ALL	GO:0010718~positive regulation of epithelial to mesenchymal transition	3.03030303	9.02E-04	0.004969515
GOTERM_BP_ALL	GO:0010770~positive regulation of cell morphogenesis involved in differentiation	3.03030303	9.02E-04	0.004969515
GOTERM_BP_ALL	GO:0008406~gonad development	6.060606061	9.00E-04	0.004975914
GOTERM_BP_ALL	GO:0019538~protein metabolic process	33.33333333	9.08E-04	0.0049928
GOTERM_BP_ALL	GO:0030856~regulation of epithelial cell differentiation	4.04040404	9.22E-04	0.005053888
GOTERM_BP_ALL	GO:0033273~response to vitamin	5.050505051	9.69E-04	0.005301236

GOTERM_BP_ALL	GO:0042990~regulation of transcription factor import into nucleus	4.04040404	0.001019152	0.005558523
GOTERM_BP_ALL	GO:0051347~positive regulation of transferase activity	8.080808081	0.00109769	0.005970482
GOTERM_BP_ALL	GO:0044248~cellular catabolic process	17.17171717	0.001113822	0.006042523
GOTERM_BP_ALL	GO:0031099~regeneration	5.050505051	0.001145234	0.006196661
GOTERM_BP_ALL	GO:0010720~positive regulation of cell development	5.050505051	0.001145234	0.006196661
GOTERM_BP_ALL	GO:0007183~SMAD protein complex assembly	3.03030303	0.001196857	0.006458779
GOTERM_BP_ALL	GO:0006285~base-excision repair, AP site formation	3.03030303	0.001196857	0.006458779
GOTERM_BP_ALL	GO:0045737~positive regulation of cyclin-dependent protein kinase activity	3.03030303	0.001196857	0.006458779
GOTERM_BP_ALL	GO:0007369~gastrulation	5.050505051	0.001208511	0.006504963
GOTERM_BP_ALL	GO:0032945~negative regulation of mononuclear cell proliferation	4.04040404	0.001233149	0.006620432
GOTERM_BP_ALL	GO:0048863~stem cell differentiation	4.04040404	0.001233149	0.006620432
GOTERM_BP_ALL	GO:0060021~palate development	4.04040404	0.001233149	0.006620432
GOTERM_BP_ALL	GO:0070664~negative regulation of leukocyte proliferation	4.04040404	0.001233149	0.006620432
GOTERM_BP_ALL	GO:0001936~regulation of endothelial cell proliferation	4.04040404	0.001233149	0.006620432
GOTERM_BP_ALL	GO:0001933~negative regulation of protein amino acid phosphorylation	4.04040404	0.001233149	0.006620432
GOTERM_BP_ALL	GO:0050672~negative regulation of lymphocyte proliferation	4.04040404	0.001233149	0.006620432
GOTERM_BP_ALL	GO:0007276~gamete generation	10.1010101	0.001253906	0.006714577
GOTERM_BP_ALL	GO:0048729~tissue morphogenesis	7.070707071	0.001273533	0.006802226
GOTERM_BP_ALL	GO:0007569~cell aging	4.04040404	0.001349956	0.007191126
GOTERM_BP_ALL	GO:0009056~catabolic process	19.19191919	0.001389028	0.007380108
GOTERM_BP_ALL	GO:0000165~MAPKKK cascade	7.070707071	0.001425205	0.007552825
GOTERM_BP_ALL	GO:0002573~myeloid leukocyte differentiation	4.04040404	0.001473496	0.007788485
GOTERM_BP_ALL	GO:0001763~morphogenesis of a branching structure	5.050505051	0.001486203	0.007835927
GOTERM_BP_ALL	GO:0048608~reproductive structure development	6.060606061	0.001523816	0.008013683
GOTERM_BP_ALL	GO:0010717~regulation of epithelial to mesenchymal transition	3.03030303	0.001532148	0.008037448
GOTERM_BP_ALL	GO:0033158~regulation of protein import into nucleus, translocation	3.03030303	0.001532148	0.008037448
GOTERM_BP_ALL	GO:0048469~cell maturation	5.050505051	0.001562016	0.008173409
GOTERM_BP_ALL	GO:0035239~tube morphogenesis	6.060606061	0.001578034	0.008236639

GOTERM_BP_ALL	GO:0045137~development of primary sexual characteristics	6.060606061	0.001578034	0.008236639
GOTERM_BP_ALL	GO:0045216~cell-cell junction organization	4.04040404	0.001603908	0.008350703
GOTERM_BP_ALL	GO:0042113~B cell activation	5.050505051	0.001640481	0.008519563
GOTERM_BP_ALL	GO:0046700~heterocycle catabolic process	5.050505051	0.001721647	0.008917745
GOTERM_BP_ALL	GO:0002250~adaptive immune response	5.050505051	0.001721647	0.008917745
GOTERM_BP_ALL	GO:0002460~adaptive immune response based on somatic recombination of immune receptors built from immunoglobulin superfamily domains	5.050505051	0.001721647	0.008917745
GOTERM_BP_ALL	GO:0030278~regulation of ossification	5.050505051	0.001805562	0.009328003
GOTERM_BP_ALL	GO:0046685~response to arsenic	3.03030303	0.001906892	0.009825505
GOTERM_BP_ALL	GO:0001953~negative regulation of cell-matrix adhesion	3.03030303	0.001906892	0.009825505
GOTERM_BP_ALL	GO:0000122~negative regulation of transcription from RNA polymerase II promoter	8.080808081	0.001978878	0.010170215
GOTERM_BP_ALL	GO:0002252~immune effector process	6.060606061	0.001998191	0.010244263
GOTERM_BP_ALL	GO:0007389~pattern specification process	8.080808081	0.002021299	0.010337272
GOTERM_BP_ALL	GO:0042552~myelination	4.04040404	0.002037735	0.010395939
GOTERM_BP_ALL	GO:0009059~macromolecule biosynthetic process	32.32323232	0.002174976	0.011066377
GOTERM_BP_ALL	GO:0051781~positive regulation of cell division	4.04040404	0.002196974	0.011151081
GOTERM_BP_ALL	GO:0017015~regulation of transforming growth factor beta receptor signaling pathway	4.04040404	0.002196974	0.011151081
GOTERM_BP_ALL	GO:0050670~regulation of lymphocyte proliferation	5.050505051	0.002268048	0.011482712
GOTERM_BP_ALL	GO:0040011~locomotion	10.1010101	0.002278603	0.01150846
GOTERM_BP_ALL	GO:0051897~positive regulation of protein kinase B signaling cascade	3.03030303	0.002320558	0.011691551
GOTERM_BP_ALL	GO:0007004~telomere maintenance via telomerase	3.03030303	0.002320558	0.011691551
GOTERM_BP_ALL	GO:0010812~negative regulation of cell-substrate adhesion	3.03030303	0.002320558	0.011691551
GOTERM_BP_ALL	GO:0001832~blastocyst growth	3.03030303	0.002320558	0.011691551
GOTERM_BP_ALL	GO:0045165~cell fate commitment	6.060606061	0.002344727	0.011784782
GOTERM_BP_ALL	GO:0051341~regulation of oxidoreductase activity	4.04040404	0.002363735	0.01185181
GOTERM_BP_ALL	GO:0070663~regulation of leukocyte proliferation	5.050505051	0.00236946	0.011852397
GOTERM_BP_ALL	GO:0032944~regulation of mononuclear cell proliferation	5.050505051	0.00236946	0.011852397

GOTERM_BP_ALL	GO:0009952~anterior/posterior pattern formation	6.060606061	0.002418967	0.012070401
GOTERM_BP_ALL	GO:0046777~protein amino acid autophosphorylation	5.050505051	0.002473954	0.012314485
GOTERM_BP_ALL	GO:0050679~positive regulation of epithelial cell proliferation	4.04040404	0.002538137	0.012602812
GOTERM_BP_ALL	GO:0006606~protein import into nucleus	5.050505051	0.002581575	0.012787491
GOTERM_BP_ALL	GO:0031647~regulation of protein stability	4.04040404	0.002720299	0.013439587
GOTERM_BP_ALL	GO:0022405~hair cycle process	4.04040404	0.002720299	0.013439587
GOTERM_BP_ALL	GO:0050868~negative regulation of T cell activation	4.04040404	0.002720299	0.013439587
GOTERM_BP_ALL	GO:0001942~hair follicle development	4.04040404	0.002720299	0.013439587
GOTERM_BP_ALL	GO:0022404~molting cycle process	4.04040404	0.002720299	0.013439587
GOTERM_BP_ALL	GO:0070507~regulation of microtubule cytoskeleton organization	4.04040404	0.002720299	0.013439587
GOTERM_BP_ALL	GO:0008366~axon ensheathment	4.04040404	0.002720299	0.013439587
GOTERM_BP_ALL	GO:0007272~ensheathment of neurons	4.04040404	0.002720299	0.013439587
GOTERM_BP_ALL	GO:0031577~spindle checkpoint	3.03030303	0.002772622	0.013664886
GOTERM_BP_ALL	GO:0060389~pathway-restricted SMAD protein phosphorylation	3.03030303	0.002772622	0.013664886
GOTERM_BP_ALL	GO:0010259~multicellular organismal aging	3.03030303	0.002772622	0.013664886
GOTERM_BP_ALL	GO:0010833~telomere maintenance via telomere lengthening	3.03030303	0.002772622	0.013664886
GOTERM_BP_ALL	GO:0051496~positive regulation of stress fiber formation	3.03030303	0.002772622	0.013664886
GOTERM_BP_ALL	GO:0045885~positive regulation of survival gene product expression	3.03030303	0.002772622	0.013664886
GOTERM_BP_ALL	GO:0045931~positive regulation of mitotic cell cycle	3.03030303	0.002772622	0.013664886
GOTERM_BP_ALL	GO:0051170~nuclear import	5.050505051	0.002806388	0.013798363
GOTERM_BP_ALL	GO:0007162~negative regulation of cell adhesion	4.04040404	0.002910331	0.014273462
GOTERM_BP_ALL	GO:0045667~regulation of osteoblast differentiation	4.04040404	0.002910331	0.014273462
GOTERM_BP_ALL	GO:0042303~molting cycle	4.04040404	0.002910331	0.014273462
GOTERM_BP_ALL	GO:0019058~viral infectious cycle	4.04040404	0.002910331	0.014273462
GOTERM_BP_ALL	GO:0042633~hair cycle	4.04040404	0.002910331	0.014273462
GOTERM_BP_ALL	GO:0000226~microtubule cytoskeleton organization	6.060606061	0.002987424	0.014615597
GOTERM_BP_ALL	GO:0044265~cellular macromolecule catabolic process	13.13131313	0.003068728	0.014976489
GOTERM_BP_ALL	GO:0051249~regulation of lymphocyte activation	6.060606061	0.003075872	0.014976734
GOTERM_BP_ALL	GO:0032770~positive regulation of monooxygenase activity	3.03030303	0.003262566	0.015843691

GOTERM_BP_ALL	GO:0048820~hair follicle maturation	3.03030303	0.003262566	0.015843691
GOTERM_BP_ALL	GO:0042993~positive regulation of transcription factor import into nucleus	3.03030303	0.003262566	0.015843691
GOTERM_BP_ALL	GO:0006351~transcription, DNA-dependent	8.080808081	0.003331061	0.016137281
GOTERM_BP_ALL	GO:0007548~sex differentiation	6.060606061	0.00335257	0.016203859
GOTERM_BP_ALL	GO:0019953~sexual reproduction	10.1010101	0.003417591	0.016478567
GOTERM_BP_ALL	GO:0030900~forebrain development	6.060606061	0.003448664	0.016589749
GOTERM_BP_ALL	GO:0050795~regulation of behavior	4.04040404	0.003528739	0.016934004
GOTERM_BP_ALL	GO:0009968~negative regulation of signal transduction	7.070707071	0.003567229	0.017040486
GOTERM_BP_ALL	GO:0034504~protein localization in nucleus	5.050505051	0.003560683	0.017047757
GOTERM_BP_ALL	GO:0032774~RNA biosynthetic process	8.080808081	0.003589894	0.017109553
GOTERM_BP_ALL	GO:0051050~positive regulation of transport	7.070707071	0.003728091	0.017723802
GOTERM_BP_ALL	GO:0051302~regulation of cell division	4.04040404	0.003751323	0.017793657
GOTERM_BP_ALL	GO:0009746~response to hexose stimulus	4.04040404	0.003751323	0.017793657
GOTERM_BP_ALL	GO:0034284~response to monosaccharide stimulus	4.04040404	0.003751323	0.017793657
GOTERM_BP_ALL	GO:0010862~positive regulation of pathway-restricted SMAD protein phosphorylation	3.03030303	0.003789877	0.017935192
GOTERM_BP_ALL	GO:0009264~deoxyribonucleotide catabolic process	3.03030303	0.003789877	0.017935192
GOTERM_BP_ALL	GO:0032233~positive regulation of actin filament bundle formation	3.03030303	0.003789877	0.017935192
GOTERM_BP_ALL	GO:0001822~kidney development	5.050505051	0.003839935	0.018130003
GOTERM_BP_ALL	GO:0034645~cellular macromolecule biosynthetic process	31.31313131	0.003947112	0.01859099
GOTERM_BP_ALL	GO:0032101~regulation of response to external stimulus	6.060606061	0.004177691	0.019625058
GOTERM_BP_ALL	GO:0032886~regulation of microtubule-based process	4.04040404	0.004221753	0.019786683
GOTERM_BP_ALL	GO:0019217~regulation of fatty acid metabolic process	4.04040404	0.004221753	0.019786683
GOTERM_BP_ALL	GO:0030324~lung development	5.050505051	0.004286105	0.020041699
GOTERM_BP_ALL	GO:0001938~positive regulation of endothelial cell proliferation	3.03030303	0.004354045	0.020312142
GOTERM_BP_ALL	GO:0045580~regulation of T cell differentiation	4.04040404	0.004726478	0.021986175
GOTERM_BP_ALL	GO:0048762~mesenchymal cell differentiation	4.04040404	0.004726478	0.021986175
GOTERM_BP_ALL	GO:0014031~mesenchymal cell development	4.04040404	0.004726478	0.021986175

GOTERM_BP_ALL	GO:0009166~nucleotide catabolic process	4.04040404	0.004726478	0.021986175
GOTERM_BP_ALL	GO:0030323~respiratory tube development	5.050505051	0.004766058	0.022120276
GOTERM_BP_ALL	GO:0080135~regulation of cellular response to stress	5.050505051	0.004933746	0.022841643
GOTERM_BP_ALL	GO:0030098~lymphocyte differentiation	5.050505051	0.004933746	0.022841643
GOTERM_BP_ALL	GO:0043535~regulation of blood vessel endothelial cell migration	3.03030303	0.004954567	0.02288733
GOTERM_BP_ALL	GO:0022612~gland morphogenesis	3.03030303	0.004954567	0.02288733
GOTERM_BP_ALL	GO:0050999~regulation of nitric-oxide synthase activity	3.03030303	0.004954567	0.02288733
GOTERM_BP_ALL	GO:0051412~response to corticosterone stimulus	3.03030303	0.004954567	0.02288733
GOTERM_BP_ALL	GO:0042176~regulation of protein catabolic process	4.04040404	0.004991921	0.023008325
GOTERM_BP_ALL	GO:0060485~mesenchyme development	4.04040404	0.004991921	0.023008325
GOTERM_BP_ALL	GO:0050767~regulation of neurogenesis	6.060606061	0.005010876	0.023044943
GOTERM_BP_ALL	GO:0002694~regulation of leukocyte activation	6.060606061	0.005010876	0.023044943
GOTERM_BP_ALL	GO:0030198~extracellular matrix organization	5.050505051	0.005105356	0.023424766
GOTERM_BP_ALL	GO:0051250~negative regulation of lymphocyte activation	4.04040404	0.005549381	0.02538683
GOTERM_BP_ALL	GO:0006919~activation of caspase activity	4.04040404	0.005549381	0.02538683
GOTERM_BP_ALL	GO:0016064~immunoglobulin mediated immune response	4.04040404	0.005549381	0.02538683
GOTERM_BP_ALL	GO:0019228~regulation of action potential in neuron	4.04040404	0.005549381	0.02538683
GOTERM_BP_ALL	GO:0060393~regulation of pathway-restricted SMAD protein phosphorylation	3.03030303	0.005590945	0.025520164
GOTERM_BP_ALL	GO:0006278~RNA-dependent DNA replication	3.03030303	0.005590945	0.025520164
GOTERM_BP_ALL	GO:0042307~positive regulation of protein import into nucleus	3.03030303	0.005590945	0.025520164
GOTERM_BP_ALL	GO:0060070~Wnt receptor signaling pathway through beta-catenin	3.03030303	0.005590945	0.025520164
GOTERM_BP_ALL	GO:0043010~camera-type eye development	5.050505051	0.005644123	0.025705267
GOTERM_BP_ALL	GO:0051240~positive regulation of multicellular organismal process	7.070707071	0.005759931	0.026171208
GOTERM_BP_ALL	GO:0060541~respiratory system development	5.050505051	0.005831824	0.026437984
GOTERM_BP_ALL	GO:0009058~biosynthetic process	36.36363636	0.005942359	0.026876489
GOTERM_BP_ALL	GO:0006955~immune response	12.12121212	0.006049115	0.027296101
GOTERM_BP_ALL	GO:0051259~protein oligomerization	6.060606061	0.006100425	0.02746687

GOTERM_BP_ALL	GO:0006338~chromatin remodeling	4.04040404	0.006142794	0.027597155
GOTERM_BP_ALL	GO:0051054~positive regulation of DNA metabolic process	4.04040404	0.006142794	0.027597155
GOTERM_BP_ALL	GO:0019724~B cell mediated immunity	4.04040404	0.006142794	0.027597155
GOTERM_BP_ALL	GO:0007565~female pregnancy	5.050505051	0.006219633	0.027879704
GOTERM_BP_ALL	GO:0050865~regulation of cell activation	6.060606061	0.006247447	0.027944137
GOTERM_BP_ALL	GO:0060491~regulation of cell projection assembly	3.03030303	0.006262684	0.02795278
GOTERM_BP_ALL	GO:0018107~peptidyl-threonine phosphorylation	3.03030303	0.006262684	0.02795278
GOTERM_BP_ALL	GO:0001837~epithelial to mesenchymal transition	3.03030303	0.006262684	0.02795278
GOTERM_BP_ALL	GO:0007611~learning or memory	5.050505051	0.006419819	0.028586282
GOTERM_BP_ALL	GO:0010565~regulation of cellular ketone metabolic process	4.04040404	0.006453167	0.028673099
GOTERM_BP_ALL	GO:0002695~negative regulation of leukocyte activation	4.04040404	0.006453167	0.028673099
GOTERM_BP_ALL	GO:0030336~negative regulation of cell migration	4.04040404	0.006453167	0.028673099
GOTERM_BP_ALL	GO:0034330~cell junction organization	4.04040404	0.006453167	0.028673099
GOTERM_BP_ALL	GO:0034656~nucleobase, nucleoside and nucleotide catabolic process	4.04040404	0.006772744	0.030013638
GOTERM_BP_ALL	GO:0034655~nucleobase, nucleoside, nucleotide and nucleic acid catabolic process	4.04040404	0.006772744	0.030013638
GOTERM_BP_ALL	GO:0007017~microtubule-based process	7.070707071	0.006841708	0.030252631
GOTERM_BP_ALL	GO:0044249~cellular biosynthetic process	35.35353535	0.006927918	0.030565757
GOTERM_BP_ALL	GO:0000724~double-strand break repair via homologous recombination	3.03030303	0.006969295	0.030682515
GOTERM_BP_ALL	GO:0048008~platelet-derived growth factor receptor signaling pathway	3.03030303	0.006969295	0.030682515
GOTERM_BP_ALL	GO:0000725~recombinational repair	3.03030303	0.006969295	0.030682515
GOTERM_BP_ALL	GO:0048483~autonomic nervous system development	3.03030303	0.006969295	0.030682515
GOTERM_BP_ALL	GO:0007098~centrosome cycle	3.03030303	0.006969295	0.030682515
GOTERM_BP_ALL	GO:0051492~regulation of stress fiber formation	3.03030303	0.006969295	0.030682515
GOTERM_BP_ALL	GO:0009743~response to carbohydrate stimulus	4.04040404	0.007439775	0.032659703
GOTERM_BP_ALL	GO:0050863~regulation of T cell activation	5.050505051	0.007711254	0.033696752
GOTERM_BP_ALL	GO:0015074~DNA integration	3.03030303	0.007710295	0.033761901

GOTERM_BP_ALL	GO:0032570~response to progesterone stimulus	3.03030303	0.007710295	0.033761901
GOTERM_BP_ALL	GO:0050866~negative regulation of cell activation	4.04040404	0.007787354	0.033955186
GOTERM_BP_ALL	GO:0040013~negative regulation of locomotion	4.04040404	0.007787354	0.033955186
GOTERM_BP_ALL	GO:0031329~regulation of cellular catabolic process	4.04040404	0.007787354	0.033955186
GOTERM_BP_ALL	GO:0009636~response to toxin	4.04040404	0.007787354	0.033955186
GOTERM_BP_ALL	GO:0042129~regulation of T cell proliferation	4.04040404	0.00814439	0.035417821
GOTERM_BP_ALL	GO:0031400~negative regulation of protein modification process	5.050505051	0.008177023	0.035485308
GOTERM_BP_ALL	GO:0016567~protein ubiquitination	5.050505051	0.008177023	0.035485308
GOTERM_BP_ALL	GO:0045429~positive regulation of nitric oxide biosynthetic process	3.03030303	0.008485205	0.03672862
GOTERM_BP_ALL	GO:0000086~G2/M transition of mitotic cell cycle	3.03030303	0.008485205	0.03672862
GOTERM_BP_ALL	GO:0009394~2'-deoxyribonucleotide metabolic process	3.03030303	0.008485205	0.03672862
GOTERM_BP_ALL	GO:0001836~release of cytochrome c from mitochondria	3.03030303	0.008485205	0.03672862
GOTERM_BP_ALL	GO:0032231~regulation of actin filament bundle formation	3.03030303	0.008485205	0.03672862
GOTERM_BP_ALL	GO:0018210~peptidyl-threonine modification	3.03030303	0.008485205	0.03672862
GOTERM_BP_ALL	GO:0001569~patterning of blood vessels	3.03030303	0.008485205	0.03672862
GOTERM_BP_ALL	GO:0045619~regulation of lymphocyte differentiation	4.04040404	0.008510937	0.0367637
GOTERM_BP_ALL	GO:0001894~tissue homeostasis	4.04040404	0.008510937	0.0367637
GOTERM_BP_ALL	GO:0030182~neuron differentiation	9.090909091	0.008761772	0.037754625
GOTERM_BP_ALL	GO:0045471~response to ethanol	4.04040404	0.008887051	0.038209105
GOTERM_BP_ALL	GO:0031668~cellular response to extracellular stimulus	4.04040404	0.008887051	0.038209105
GOTERM_BP_ALL	GO:0042060~wound healing	6.060606061	0.008951302	0.038403703
GOTERM_BP_ALL	GO:0051960~regulation of nervous system development	6.060606061	0.009143326	0.039136131
GOTERM_BP_ALL	GO:0007292~female gamete generation	4.04040404	0.009272784	0.039602123
GOTERM_BP_ALL	GO:0030217~T cell differentiation	4.04040404	0.009272784	0.039602123
GOTERM_BP_ALL	GO:0048754~branching morphogenesis of a tube	4.04040404	0.009272784	0.039602123
GOTERM_BP_ALL	GO:0046824~positive regulation of nucleocytoplasmic transport	3.03030303	0.009293549	0.039610055
GOTERM_BP_ALL	GO:0008633~activation of pro-apoptotic gene products	3.03030303	0.009293549	0.039610055
GOTERM_BP_ALL	GO:0045670~regulation of osteoclast differentiation	3.03030303	0.009293549	0.039610055

GOTERM_BP_ALL	GO:0022407~regulation of cell-cell adhesion	3.03030303	0.009293549	0.039610055
GOTERM_BP_ALL	GO:0048538~thymus development	3.03030303	0.009293549	0.039610055
GOTERM_BP_ALL	GO:0080134~regulation of response to stress	7.070707071	0.00992989	0.042193099
GOTERM_BP_ALL	GO:0042110~T cell activation	5.050505051	0.009951688	0.042200086
GOTERM_BP_ALL	GO:0042698~ovulation cycle	4.04040404	0.0100733	0.04262242
GOTERM_BP_ALL	GO:0044270~nitrogen compound catabolic process	4.04040404	0.0100733	0.04262242
GOTERM_BP_ALL	GO:0003002~regionalization	6.060606061	0.010146324	0.042756543
GOTERM_BP_ALL	GO:0043271~negative regulation of ion transport	3.03030303	0.010134857	0.042793605
GOTERM_BP_ALL	GO:0035264~multicellular organism growth	3.03030303	0.010134857	0.042793605
GOTERM_BP_ALL	GO:0010332~response to gamma radiation	3.03030303	0.010134857	0.042793605
GOTERM_BP_ALL	GO:0048511~rhythmic process	5.050505051	0.01050111	0.044051804
GOTERM_BP_ALL	GO:0001508~regulation of action potential	4.04040404	0.010488176	0.044085202
GOTERM_BP_ALL	GO:0001101~response to acid	3.03030303	0.011008665	0.046052167
GOTERM_BP_ALL	GO:0017038~protein import	5.050505051	0.011361504	0.047315393
GOTERM_BP_ALL	GO:0002449~lymphocyte mediated immunity	4.04040404	0.011347378	0.047350077
GOTERM_BP_ALL	GO:0001654~eye development	5.050505051	0.011658092	0.048432702
GOTERM_BP_ALL	GO:0032446~protein modification by small protein conjugation	5.050505051	0.011658092	0.048432702
GOTERM_BP_ALL	GO:0010594~regulation of endothelial cell migration	3.03030303	0.011914513	0.049381348
GOTERM_BP_ALL	GO:0045862~positive regulation of proteolysis	3.03030303	0.011914513	0.049381348
GOTERM_BP_ALL	GO:0032273~positive regulation of protein polymerization	3.03030303	0.011914513	0.049381348
GOTERM_CC_ALL	GO:0044428~nuclear part	51.51515152	6.60E-23	1.90E-20
GOTERM_CC_ALL	GO:0005654~nucleoplasm	35.35353535	2.56E-19	3.68E-17
GOTERM_CC_ALL	GO:0005634~nucleus	73.73737374	1.43E-18	1.37E-16
GOTERM_CC_ALL	GO:0043233~organelle lumen	46.46464646	1.96E-18	1.41E-16
GOTERM_CC_ALL	GO:0031974~membrane-enclosed lumen	46.46464646	4.20E-18	2.42E-16
GOTERM_CC_ALL	GO:0070013~intracellular organelle lumen	45.45454545	5.74E-18	2.75E-16
GOTERM_CC_ALL	GO:0031981~nuclear lumen	41.41414141	8.86E-18	3.65E-16
GOTERM_CC_ALL	GO:0044446~intracellular organelle part	62.62626263	1.45E-14	5.24E-13
GOTERM_CC_ALL	GO:0044422~organelle part	62.62626263	1.97E-14	6.32E-13
GOTERM_CC_ALL	GO:0005694~chromosome	22.22222222	2.60E-13	7.50E-12
GOTERM_CC_ALL	GO:0043227~membrane-bounded organelle	82.82828283	7.25E-13	1.74E-11
GOTERM_CC_ALL	GO:0043231~intracellular membrane-bounded organelle	82.82828283	6.84E-13	1.79E-11
GOTERM_CC_ALL	GO:0044427~chromosomal part	19.19191919	1.16E-11	2.56E-10
GOTERM_CC_ALL	GO:0043234~protein complex	44.44444444	2.76E-11	5.68E-10

GOTERM_CC_ALL	GO:0044424~intracellular part	91.91919192	3.95E-11	7.58E-10
GOTERM_CC_ALL	GO:0032300~mismatch repair complex	6.060606061	1.42E-10	2.56E-09
GOTERM_CC_ALL	GO:0000228~nuclear chromosome	13.13131313	2.18E-10	3.69E-09
GOTERM_CC_ALL	GO:0032991~macromolecular complex	47.47474747	3.39E-10	5.42E-09
GOTERM_CC_ALL	GO:0005622~intracellular	91.91919192	6.18E-10	9.37E-09
GOTERM_CC_ALL	GO:0043229~intracellular organelle	82.82828283	1.57E-09	2.26E-08
GOTERM_CC_ALL	GO:0043226~organelle	82.82828283	1.71E-09	2.35E-08
GOTERM_CC_ALL	GO:0005667~transcription factor complex	13.13131313	4.28E-09	5.60E-08
GOTERM_CC_ALL	GO:0043232~intracellular non-membrane-bounded organelle	39.39393939	2.84E-08	3.55E-07
GOTERM_CC_ALL	GO:0043228~non-membrane-bounded organelle	39.39393939	2.84E-08	3.55E-07
GOTERM_CC_ALL	GO:0044454~nuclear chromosome part	10.1010101	4.60E-08	5.52E-07
GOTERM_CC_ALL	GO:0000781~chromosome, telomeric region	6.060606061	7.24E-07	8.34E-06
GOTERM_CC_ALL	GO:0044451~nucleoplasm part	16.16161616	9.60E-07	1.06E-05
GOTERM_CC_ALL	GO:0000784~nuclear chromosome, telomeric region	5.050505051	6.61E-06	7.05E-05
GOTERM_CC_ALL	GO:0005737~cytoplasm	66.66666667	8.10E-06	8.33E-05
GOTERM_CC_ALL	GO:0005829~cytosol	23.23232323	8.81E-06	8.75E-05
GOTERM_CC_ALL	GO:0044444~cytoplasmic part	48.48484848	1.02E-04	9.48E-04
GOTERM_CC_ALL	GO:0015630~microtubule cytoskeleton	13.13131313	1.02E-04	9.77E-04
GOTERM_CC_ALL	GO:0005815~microtubule organizing center	9.090909091	1.35E-04	0.001215817
GOTERM_CC_ALL	GO:0032389~MutLalpha complex	3.03030303	2.10E-04	0.001832033
GOTERM_CC_ALL	GO:0005813~centrosome	8.080808081	3.86E-04	0.00326914
GOTERM_CC_ALL	GO:0005730~nucleolus	13.13131313	8.99E-04	0.007170081
GOTERM_CC_ALL	GO:0042995~cell projection	13.13131313	8.88E-04	0.007283294
GOTERM_CC_ALL	GO:0016605~PML body	4.04040404	9.89E-04	0.007668914
GOTERM_CC_ALL	GO:0000793~condensed chromosome	6.060606061	0.001049235	0.007924704
GOTERM_CC_ALL	GO:0000785~chromatin	7.070707071	0.001258984	0.009029385
GOTERM_CC_ALL	GO:0000782~telomere cap complex	3.03030303	0.001236212	0.009093002
GOTERM_CC_ALL	GO:0000783~nuclear telomere cap complex	3.03030303	0.001236212	0.009093002
GOTERM_CC_ALL	GO:0045121~membrane raft	6.060606061	0.001657984	0.011588326
GOTERM_CC_ALL	GO:0000307~cyclin-dependent protein kinase holoenzyme complex	3.03030303	0.001874011	0.012780049
GOTERM_CC_ALL	GO:0016328~lateral plasma membrane	3.03030303	0.003064679	0.020347871
GOTERM_CC_ALL	GO:0016514~SWI/SNF complex	3.03030303	0.003522458	0.022832104
GOTERM_CC_ALL	GO:0070603~SWI/SNF-type complex	3.03030303	0.003522458	0.022832104

GOTERM_CC_ALL	GO:0043005~neuron projection	8.080808081	0.004379747	0.027701036
GOTERM_CC_ALL	GO:0016323~basolateral plasma membrane	6.060606061	0.007384526	0.045344851
GOTERM_MF_ALL	GO:0003690~double-stranded DNA binding	16.16161616	2.44E-17	4.81E-15
GOTERM_MF_ALL	GO:0043566~structure-specific DNA binding	18.18181818	1.99E-17	7.83E-15
GOTERM_MF_ALL	GO:0005515~protein binding	87.87878788	1.70E-15	2.19E-13
GOTERM_MF_ALL	GO:0005524~ATP binding	38.38383838	1.99E-14	1.97E-12
GOTERM_MF_ALL	GO:0032559~adenyl ribonucleotide binding	38.38383838	3.05E-14	2.41E-12
GOTERM_MF_ALL	GO:0003677~DNA binding	46.46464646	1.09E-13	7.17E-12
GOTERM_MF_ALL	GO:0030554~adenyl nucleotide binding	38.38383838	1.55E-13	8.71E-12
GOTERM_MF_ALL	GO:0001883~purine nucleoside binding	38.38383838	2.47E-13	1.22E-11
GOTERM_MF_ALL	GO:0001882~nucleoside binding	38.38383838	3.05E-13	1.34E-11
GOTERM_MF_ALL	GO:0032553~ribonucleotide binding	38.38383838	1.58E-11	6.22E-10
GOTERM_MF_ALL	GO:0032555~purine ribonucleotide binding	38.38383838	1.58E-11	6.22E-10
GOTERM_MF_ALL	GO:0017076~purine nucleotide binding	38.38383838	5.75E-11	2.06E-09
GOTERM_MF_ALL	GO:0008094~DNA-dependent ATPase activity	10.1010101	6.52E-11	2.14E-09
GOTERM_MF_ALL	GO:0003824~catalytic activity	64.64646465	9.38E-11	2.84E-09
GOTERM_MF_ALL	GO:0003676~nucleic acid binding	49.49494949	2.66E-10	7.48E-09
GOTERM_MF_ALL	GO:0032404~mismatch repair complex binding	6.060606061	4.57E-10	1.20E-08
GOTERM_MF_ALL	GO:0016563~transcription activator activity	18.18181818	5.07E-10	1.25E-08
GOTERM_MF_ALL	GO:0003684~damaged DNA binding	9.090909091	7.06E-10	1.64E-08
GOTERM_MF_ALL	GO:0016301~kinase activity	24.24242424	9.68E-10	2.12E-08
GOTERM_MF_ALL	GO:0016740~transferase activity	34.34343434	1.84E-09	3.82E-08
GOTERM_MF_ALL	GO:0016772~transferase activity, transferring phosphorus-containing groups	25.25252525	2.97E-09	5.58E-08
GOTERM_MF_ALL	GO:0019899~enzyme binding	19.19191919	2.89E-09	5.69E-08
GOTERM_MF_ALL	GO:0004672~protein kinase activity	20.2020202	4.56E-09	8.17E-08
GOTERM_MF_ALL	GO:0000166~nucleotide binding	38.38383838	5.18E-09	8.88E-08
GOTERM_MF_ALL	GO:0004519~endonuclease activity	10.1010101	1.11E-08	1.75E-07
GOTERM_MF_ALL	GO:0042802~identical protein binding	20.2020202	1.11E-08	1.82E-07
GOTERM_MF_ALL	GO:0046332~SMAD binding	8.080808081	1.24E-08	1.89E-07
GOTERM_MF_ALL	GO:0016773~phosphotransferase activity, alcohol group as acceptor	21.21212121	1.34E-08	1.95E-07
GOTERM_MF_ALL	GO:0008134~transcription factor binding	18.18181818	1.48E-08	2.08E-07

GOTERM_MF_ALL	GO:0008022~protein C-terminus binding	11.11111111	1.65E-08	2.24E-07
GOTERM_MF_ALL	GO:0047485~protein N-terminus binding	9.090909091	1.75E-08	2.30E-07
GOTERM_MF_ALL	GO:0046983~protein dimerization activity	18.18181818	3.31E-08	4.20E-07
GOTERM_MF_ALL	GO:0004518~nuclease activity	11.11111111	4.90E-08	6.03E-07
GOTERM_MF_ALL	GO:0010843~promoter binding	8.080808081	5.81E-08	6.94E-07
GOTERM_MF_ALL	GO:0030983~mismatched DNA binding	6.060606061	8.99E-08	1.04E-06
GOTERM_MF_ALL	GO:0016887~ATPase activity	14.14141414	1.40E-07	1.57E-06
GOTERM_MF_ALL	GO:0032403~protein complex binding	11.11111111	3.69E-07	4.04E-06
GOTERM_MF_ALL	GO:0004003~ATP-dependent DNA helicase activity	6.060606061	3.99E-07	4.25E-06
GOTERM_MF_ALL	GO:0032137~guanine/thymine mispair binding	4.04040404	9.22E-07	9.56E-06
GOTERM_MF_ALL	GO:0032138~single base insertion or deletion binding	4.04040404	9.22E-07	9.56E-06
GOTERM_MF_ALL	GO:0032134~mispaired DNA binding	4.04040404	9.22E-07	9.56E-06
GOTERM_MF_ALL	GO:0003697~single-stranded DNA binding	7.070707071	1.10E-06	1.12E-05
GOTERM_MF_ALL	GO:0032407~MutSalpha complex binding	4.04040404	2.30E-06	2.26E-05
GOTERM_MF_ALL	GO:0032135~DNA insertion or deletion binding	4.04040404	2.30E-06	2.26E-05
GOTERM_MF_ALL	GO:0043565~sequence-specific DNA binding	16.16161616	4.60E-06	4.22E-05
GOTERM_MF_ALL	GO:0003678~DNA helicase activity	6.060606061	4.59E-06	4.30E-05
GOTERM_MF_ALL	GO:0032405~MutLalpha complex binding	4.04040404	4.57E-06	4.39E-05
GOTERM_MF_ALL	GO:0042623~ATPase activity, coupled	11.11111111	7.11E-06	6.36E-05
GOTERM_MF_ALL	GO:0004520~endodeoxyribonuclease activity	5.050505051	9.35E-06	8.19E-05
GOTERM_MF_ALL	GO:0030528~transcription regulator activity	25.25252525	1.19E-05	1.02E-04
GOTERM_MF_ALL	GO:0004674~protein serine/threonine kinase activity	13.13131313	1.37E-05	1.15E-04
GOTERM_MF_ALL	GO:0000217~DNA secondary structure binding	4.04040404	1.89E-05	1.55E-04
GOTERM_MF_ALL	GO:0070412~R-SMAD binding	4.04040404	2.69E-05	2.17E-04
GOTERM_MF_ALL	GO:0046982~protein heterodimerization activity	9.090909091	4.48E-05	3.53E-04
GOTERM_MF_ALL	GO:0003713~transcription coactivator activity	9.090909091	5.48E-05	4.23E-04
GOTERM_MF_ALL	GO:0004536~deoxyribonuclease activity	5.050505051	5.60E-05	4.24E-04
GOTERM_MF_ALL	GO:0031625~ubiquitin protein ligase binding	5.050505051	7.04E-05	5.24E-04

GOTERM_MF_ALL	GO:0032357~oxidized purine DNA binding	3.03030303	1.14E-04	8.31E-04
GOTERM_MF_ALL	GO:0000400~four-way junction DNA binding	3.03030303	1.14E-04	8.31E-04
GOTERM_MF_ALL	GO:0032142~single guanine insertion binding	3.03030303	1.14E-04	8.31E-04
GOTERM_MF_ALL	GO:0032356~oxidized DNA binding	3.03030303	1.14E-04	8.31E-04
GOTERM_MF_ALL	GO:0019900~kinase binding	8.080808081	1.24E-04	8.85E-04
GOTERM_MF_ALL	GO:0005488~binding	91.91919192	1.29E-04	9.10E-04
GOTERM_MF_ALL	GO:0016538~cyclin-dependent protein kinase regulator activity	4.04040404	1.48E-04	0.001021794
GOTERM_MF_ALL	GO:0005160~transforming growth factor beta receptor binding	4.04040404	1.48E-04	0.001021794
GOTERM_MF_ALL	GO:0042803~protein homodimerization activity	10.1010101	2.27E-04	0.001543842
GOTERM_MF_ALL	GO:0004386~helicase activity	7.070707071	2.34E-04	0.001563738
GOTERM_MF_ALL	GO:0005102~receptor binding	16.16161616	3.46E-04	0.002272656
GOTERM_MF_ALL	GO:0070035~purine NTP-dependent helicase activity	6.060606061	3.57E-04	0.002301667
GOTERM_MF_ALL	GO:0008026~ATP-dependent helicase activity	6.060606061	3.57E-04	0.002301667
GOTERM_MF_ALL	GO:0019237~centromeric DNA binding	3.03030303	3.77E-04	0.002391092
GOTERM_MF_ALL	GO:0030235~nitric-oxide synthase regulator activity	3.03030303	3.77E-04	0.002391092
GOTERM_MF_ALL	GO:0050681~androgen receptor binding	4.04040404	5.43E-04	0.0033383
GOTERM_MF_ALL	GO:0017111~nucleoside-triphosphatase activity	14.14141414	5.43E-04	0.003388938
GOTERM_MF_ALL	GO:0008408~3'-5' exonuclease activity	4.04040404	6.78E-04	0.004103215
GOTERM_MF_ALL	GO:0004714~transmembrane receptor protein tyrosine kinase activity	5.050505051	7.91E-04	0.004645346
GOTERM_MF_ALL	GO:0016462~pyrophosphatase activity	14.14141414	7.81E-04	0.004655193
GOTERM_MF_ALL	GO:0016818~hydrolase activity, acting on acid anhydrides, in phosphorus-containing anhydrides	14.14141414	8.10E-04	0.004686565
GOTERM_MF_ALL	GO:0016817~hydrolase activity, acting on acid anhydrides	14.14141414	8.51E-04	0.004847582
GOTERM_MF_ALL	GO:0019887~protein kinase regulator activity	5.050505051	0.001608212	0.009018314
GOTERM_MF_ALL	GO:0035258~steroid hormone receptor binding	4.04040404	0.001669572	0.009102225
GOTERM_MF_ALL	GO:0004861~cyclin-dependent protein kinase inhibitor activity	3.03030303	0.001660919	0.009182176
GOTERM_MF_ALL	GO:0019903~protein phosphatase binding	4.04040404	0.001800622	0.009680031
GOTERM_MF_ALL	GO:0003712~transcription cofactor activity	9.090909091	0.001871707	0.009925331

GOTERM_MF_ALL	GO:0019104~DNA N-glycosylase activity	3.03030303	0.002021819	0.010575719
GOTERM_MF_ALL	GO:0005072~transforming growth factor beta receptor, cytoplasmic mediator activity	3.03030303	0.002021819	0.010575719
GOTERM_MF_ALL	GO:0003682~chromatin binding	6.060606061	0.00241392	0.012451231
GOTERM_MF_ALL	GO:0019902~phosphatase binding	4.04040404	0.00255169	0.012988298
GOTERM_MF_ALL	GO:0019207~kinase regulator activity	5.050505051	0.002667316	0.013400767
GOTERM_MF_ALL	GO:0015631~tubulin binding	5.050505051	0.003466319	0.017168659
GOTERM_MF_ALL	GO:0004713~protein tyrosine kinase activity	6.060606061	0.003732806	0.018249883
GOTERM_MF_ALL	GO:0016788~hydrolase activity, acting on ester bonds	12.12121212	0.003836474	0.01829973
GOTERM_MF_ALL	GO:0042162~telomeric DNA binding	3.03030303	0.003797972	0.018339015
GOTERM_MF_ALL	GO:0004527~exonuclease activity	4.04040404	0.004335629	0.020414666
GOTERM_MF_ALL	GO:0002039~p53 binding	3.03030303	0.004879753	0.022683171
GOTERM_MF_ALL	GO:0030291~protein serine/threonine kinase inhibitor activity	3.03030303	0.004879753	0.022683171
GOTERM_MF_ALL	GO:0016787~hydrolase activity	25.25252525	0.005576856	0.02558961
GOTERM_MF_ALL	GO:0004364~glutathione transferase activity	3.03030303	0.006735399	0.030487501
GOTERM_MF_ALL	GO:0016799~hydrolase activity, hydrolyzing N-glycosyl compounds	3.03030303	0.006735399	0.030487501
GOTERM_MF_ALL	GO:0000287~magnesium ion binding	9.090909091	0.007023751	0.03141685
GOTERM_MF_ALL	GO:0008080~N-acetyltransferase activity	4.04040404	0.009030478	0.039801822

Supplementary Table S14: The significantly enriched InterPro annotations in the 100 CPGs

Annotation source	Functional term	Proportion of annotated genes to all 100 CPGs	Raw *P*-value	Benjamini-Hochberg adjusted *P*-value
INTERPRO	IPR000719:Protein kinase, core	14.14141414	3.34E-06	0.001042387
INTERPRO	IPR017441:Protein kinase, ATP binding site	13.13131313	1.17E-05	0.001818709
INTERPRO	IPR007695:DNA mismatch repair protein MutS-like, N-terminal	3.03030303	9.82E-05	0.010163554
INTERPRO	IPR000403:Phosphatidylinositol 3- and 4-kinase, catalytic	4.04040404	1.42E-04	0.01102483
INTERPRO	IPR018936:Phosphatidylinositol 3- and 4-kinase, conserved site	4.04040404	1.42E-04	0.01102483
INTERPRO	IPR013507:DNA mismatch repair protein, C-terminal	3.03030303	1.96E-04	0.012138885
INTERPRO	IPR007860:DNA mismatch repair protein MutS, connector	3.03030303	1.96E-04	0.012138885
INTERPRO	IPR008266:Tyrosine protein kinase, active site	6.060606061	2.43E-04	0.012539275
INTERPRO	IPR001357:BRCT	4.04040404	3.44E-04	0.013320256
INTERPRO	IPR003265:HhH-GPD domain	3.03030303	3.25E-04	0.014381348
INTERPRO	IPR003151:PIK-related kinase, FAT	3.03030303	3.25E-04	0.014381348
INTERPRO	IPR013632:Rad51, C-terminal	3.03030303	3.25E-04	0.014381348
INTERPRO	IPR007696:DNA mismatch repair protein MutS, core	3.03030303	3.25E-04	0.014381348
INTERPRO	IPR014009:PIK-related kinase	3.03030303	4.86E-04	0.016696944
INTERPRO	IPR000432:DNA mismatch repair protein MutS, C-terminal	3.03030303	4.86E-04	0.016696944
INTERPRO	IPR016467:DNA repair and recombination, RecA-like	3.03030303	4.86E-04	0.016696944
INTERPRO	IPR014762:DNA mismatch repair, conserved site	3.03030303	4.86E-04	0.016696944
INTERPRO	IPR003152:PIK-related kinase, FATC	3.03030303	4.86E-04	0.016696944
INTERPRO	IPR001245:Tyrosine protein kinase	6.060606061	5.99E-04	0.018521817
INTERPRO	IPR001553:RecA bacterial DNA recombination	3.03030303	9.00E-04	0.025205506
INTERPRO	IPR002099:DNA mismatch repair protein	3.03030303	0.001747343	0.044452365
